# Rh(I)/(III)‐N‐Heterocyclic Carbene Complexes: Effect of Steric Confinement Upon Immobilization on Regio‐ and Stereoselectivity in the Hydrosilylation of Alkynes

**DOI:** 10.1002/chem.202103099

**Published:** 2021-11-08

**Authors:** Pradeep K. R. Panyam, Boshra Atwi, Felix Ziegler, Wolfgang Frey, Michal Nowakowski, Matthias Bauer, Michael R. Buchmeiser

**Affiliations:** ^1^ Institute of Polymer Chemistry University of Stuttgart Pfaffenwaldring 55 70569 Stuttgart Germany; ^2^ Institute of Organic Chemistry University of Stuttgart Pfaffenwaldring 55 70569 Stuttgart Germany; ^3^ German Institutes of Textile and Fiber Research Körschtalstr. 26 73770 Denkendorf Germany; ^4^ Chemistry Department Paderborn University Warburger Str. 100 33098 Paderborn Germany

**Keywords:** alkynes, hydrosilylation, N-Heterocyclic carbenes, rhodium, steric confinement

## Abstract

Rh(I) NHC and Rh(III) Cp* NHC complexes (Cp*=pentamethylcyclopentadienyl, NHC=N‐heterocyclic carbene=pyrid‐2‐ylimidazol‐2‐ylidene (Py−Im), thiophen‐2‐ylimidazol‐2‐ylidene) are presented. Selected catalysts were selectively immobilized inside the mesopores of SBA‐15 with average pore diameters of 5.0 and 6.2 nm. Together with their homogenous progenitors, the immobilized catalysts were used in the hydrosilylation of terminal alkynes. For aromatic alkynes, both the neutral and cationic Rh(I) complexes showed excellent reactivity with exclusive formation of the β(*E*)‐isomer. For aliphatic alkynes, however, selectivity of the Rh(I) complexes was low. By contrast, the neutral and cationic Rh(III) Cp* NHC complexes proved to be highly regio‐ and stereoselective catalysts, allowing for the formation of the thermodynamically less stable β‐(*Z*)‐vinylsilane isomers at room temperature. Notably, the SBA‐15 immobilized Rh(I) catalysts, in which the pore walls provide an additional confinement, showed excellent β‐(*Z*)‐selectivity in the hydrosilylation of aliphatic alkynes, too. Also, in the case of 4‐aminophenylacetylene, selective formation of the β(*Z*)‐isomer was observed with a neutral SBA‐15 supported Rh(III) Cp* NHC complex but not with its homogenous counterpart. These are the first examples of high β(*Z*)‐selectivity in the hydrosilylation of alkynes by confinement generated upon immobilization inside mesoporous silica.

## Graphical abstract

**Novel Rh(III)** pentamethylcyclopentadienyl and Rh(I) complexes containing chelating N‐heterocyclic carbenes have been prepared and used in the hydrosilylation of 1‐alkynes. Selected catalysts were immobilized within the mesopores of SBA‐15. The confinement effects either provided by the ligands or the mesoporous support have been studied and allow for hydrosilylation with β(*Z*)‐selectivity of up to 100 % with both Rh(I) and Rh(III) complexes.

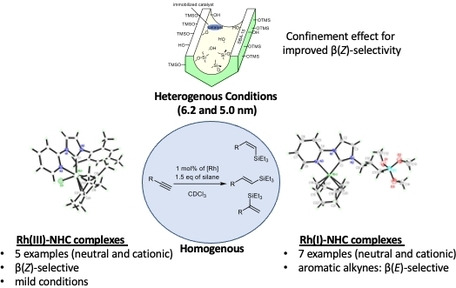

## Introduction

Owing to the versatility, ease of handling, low toxicity, and reasonable stability relative to other vinyl‐metal species, vinylsilanes are highly valuable building blocks in organic synthesis, polymer chemistry, and materials science.^[1]^ The most straightforward and atom‐economic approach to their synthesis is the transition metal‐catalysed hydrosilylation of alkynes.^[2]^ Predominantly Pt‐^[3]^ and Rh‐based catalysts^[4]^ including nanoparticulate supported systems^[5]^ have been used; however, alternative catalytic systems, for example based on ruthenium,^[6]^ cobalt,^[7]^ iron^[8]^ or nickel^[9]^ have been developed more recently.

**Figure 1:**
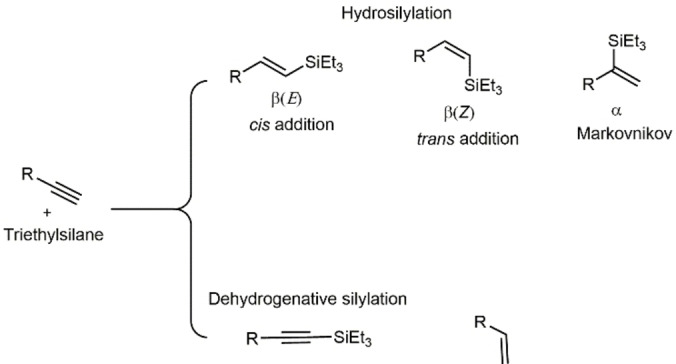
Possible products of the hydrosilylation of terminal alkynes.

Generally, the hydrosilylation of terminal alkynes can yield different isomers (Figure 1). Thus, the reaction may proceed via *anti*‐Markovnikov addition to afford the β(*E*)‐ and β‐(*Z*)‐vinylsilane stereoisomers (resulting from a *syn*‐ and *anti*‐addition, respectively). Markovnikov addition results in the formation of the α‐vinylsilane isomer. Additionally, the formation of the competitive dehydrogenative silylation product, namely, alkynylsilane and the corresponding alkene, has been frequently observed for some catalysts. Consequently, the control of the regio‐ and stereoselectivity along the H−Si addition process is a major issue.

Metal N‐heterocyclic carbene (NHC) complexes have gained increasing importance in the hydrosilylation of alkynes. Co NHC complexes[^7a^, ^7b^, ^10^] usually provide *E/Z*‐mixtures of the *anti*‐Markovnikov product, though in selected cases, high *E‐*selectivity was observed.^[11]^ In contrast, tailored Co bis(NHC) complexes and Co pincer complexes were reported to display high Markovnikov selectivity.^[12]^ Detailed studies on Co(III) bis(NHC) complexes also revealed that bulky NHCs favour the formation of the *anti*‐Markovnikov products in alkene hydrosilylation.^[12a]^ Rh(I) NHC complexes provide high *anti*‐Markovnikov selectivity in the hydrosilylation of alkenes.^[13]^

Despite the vast number of catalysts including Rh NHC complexes,^[14]^ there is still need for the development of catalysts that allow for high stereo‐ *and* regioselectivity.[^4^, ^6b^, ^15^] Also, while substantial efforts have been dedicated to the immobilization of organometallic catalysts over the last decades,[^2b^, ^16^] still few examples of Rh‐catalysed alkyne hydrosilylations by supported catalysts with high regio‐ and stereoselectivity exist.[^15a^, ^17^] In fact, reports on the hydrosilylation of alkynes with high β‐(*Z*)‐selectivity are scarce and so far required the use of cyclometalated Rh(III) Cp* NHC complexes or complex heterogeneous hybrid analogues.^[18]^

We were interested in the question whether a specific environment provided by spatial confinement^[19]^ inside porous silica supports allows for high β‐(*Z*)‐selectivity in the hydrosilylation of both aromatic and aliphatic 1‐alkynes. To address this issue, both Rh(I) and Rh(III) catalysts containing *N*‐ or *S*‐chelating NHCs were prepared and selectively immobilized inside the pores of mesoporous SBA‐15. We used chelating NHCs since these usually display enhanced stability, the more if the bidentate ligand forms a five‐membered metallacycle.

## Results and Discussion

The precursors 1‐(pyrid‐2‐yl)‐1*H*‐imidazole and 1‐(thienyl)‐1*H*‐imidazole were synthesized using a modified Ullman‐type coupling reaction of imidazole with 2‐bromopyridine and 2‐bromo thiazole, respectively, employing dimethyl sulfoxide (DMSO) as solvent and K_2_CO_3_ as base.^[20]^ The imidazolium salts **L1** and **L3** were obtained by reaction of 3‐iodopropyltrimethoxysilane with 1‐pyridyl‐1*H*‐imidazole and 1‐(thienyl)‐1*H*‐imidazole, respectively. The ligand **L2** was prepared according to the literature.^[21]^

### Rh(I)‐NHC complexes

**Scheme 1:**
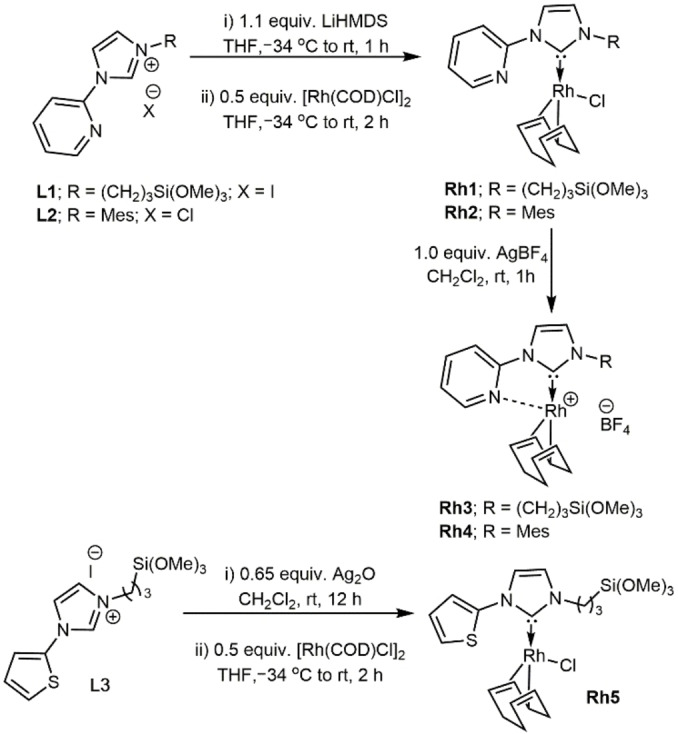
Synthesis of Rh(I)−NHC complexes **Rh1**–**Rh5**.

The rhodium complexes **Rh1** and **Rh2** were synthesized by deprotonating the imidazolium salts **L1** and **L2** with LiHMDS, followed by the addition of half an equivalent of the rhodium dimer [Rh(COD)Cl]_2_ (Scheme 1).

**Figure 2:**
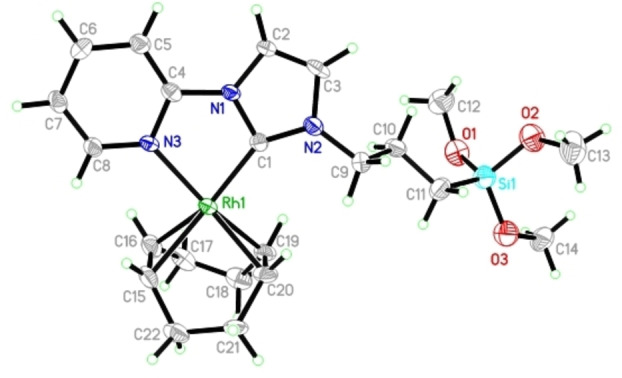
Single crystal X‐ray structure of **Rh3**. Selected bond lengths [pm] and bond angles [°]: Rh1−C1 202.5(7), Rh1−N3 211.0(5), Rh1−C15 223.2(7), Rh1−C16 217.5(7), Rh1−C19 214.9(5), Rh1−C20 214.6(7); C1−Rh1−N3 78.6, C1−Rh1−C15 169.8(2), C1−Rh1−C16 152.4(3), C1−Rh1−C19 97.8(2), C1−Rh1−C20 99.4(3), N3−Rh1−C15 97.7(2), N3−Rh1−C16 92.7(2), N3−Rh1−C19 160.8(2), N3−Rh1−C20 161.2(2). Thermal ellipsoids are set at a 50 % probability level. The BF_4_^−^ anion and the co‐crystallized solvent molecules were omitted for clarity.

Under these reaction conditions we were unable to prepare the Rh(I) analogue of ligand **L3** containing a thiophene instead of a pyridine donor group. The imidazolium salt **L3** was therefore reacted with Ag_2_O in CH_2_Cl_2_ at room temperature followed by the addition of [Rh(COD)Cl]_2_ to yield **Rh5**. Reaction of complexes **Rh1** and **Rh2** with 1 equiv. of AgBF_4_ in CH_2_Cl_2_ at room temperature resulted in the precipitation of AgCl and the formation of yellow to orange solutions from which the cationic complexes **Rh3** and **Rh4** were isolated in 67 and 74 % yield, respectively. Complexes **Rh1**–**Rh5** were stable as solids under atmospheric conditions and were obtained as yellow to orange‐coloured solids after recrystallization from CH_2_Cl_2_/diethyl ether. The ^13^C NMR spectra displayed one doublet signal for the NHC's C_2_‐carbon in the range of 173–184 ppm (*J*_*Rh‐C*_=52 Hz) for complexes **Rh1**–**Rh7**, which is in the usual range for Rh(I)−NHC complexes.^[14c]^ In **Rh3** and **Rh4**, the pyridine fragment of the hemilabile NHCs coordinates to the metal. This chelation of the rhodium center in **Rh3** by the pyridine fragment was confirmed by single‐crystal X‐ray analysis (Figure 2).

Complex **Rh3** (Figure 2) crystallizes in the triclinic space group *P‐1*. The Rh centre experiences a slightly distorted square planar geometry defined by the coordination of the metal to the two olefinic bonds of the cyclooctadiene ligand, the carbon atom of the NHC ligand and the nitrogen lone pair of pyridine fragment. The bite angle of the pyridyl‐NHC ligand (C1−Rh−N3) is ca. 78.6°. The C(NHC)‐Rh and N−Rh bond lengths are 202.5 and 211.0 pm, respectively, and are inconspicuous.^[22]^ The Rh−C(COD) distances (Rh1−C15=223.2(7) pm and Rh1−C16=217.5(7) pm) *trans* to the NHC C_2_‐carbon are slightly longer than the corresponding distances (Rh1−C19=214.9(5) pm and Rh1−C20=214.6(7) pm) *cis* to the NHC, reflecting the *trans* influence of the NHC.

**Scheme 2:**
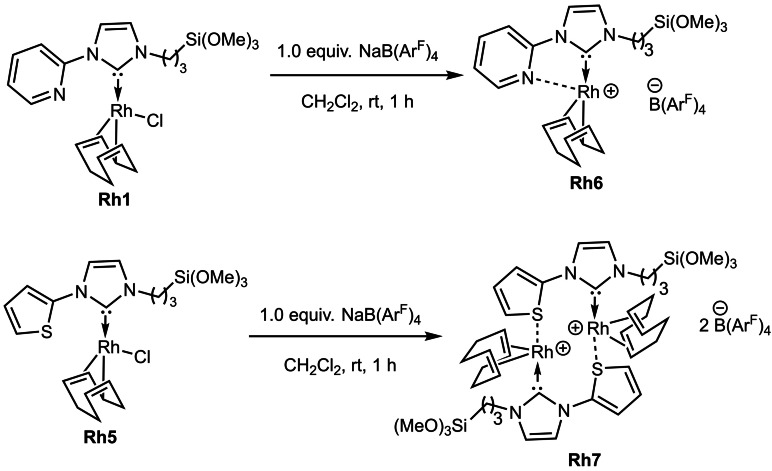
Synthesis of the Rh(I) NHC B(Ar^F^)_4_ complexes **Rh6** and **Rh7**.

**Figure 3:**
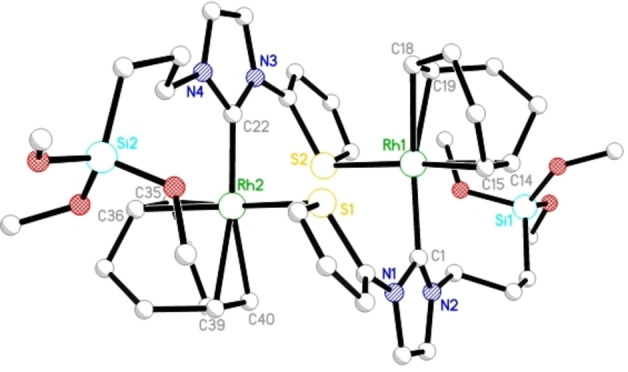
Preliminary single crystal X‐ray structure of **Rh7**. Selected bond lengths [pm] and bond angles [°]: Rh1−C1 209.6(9), Rh1−S2 241.5(2), Rh1−C14 216.0(1), Rh1−C15 215.0(9), Rh1−C18 219.0(1), Rh1−C19 224.7(8); Rh2−C22 200.7(8), Rh2−S1 239.5(3), Rh2−C35 216.0(1), Rh2−C36 217.0(1), Rh2−C39 228.0(1), Rh2−C40 225.0(1); S2−Rh1−C1 92.4(3), S2−Rh1−C14 154.2(3), S2−Rh1−C15 167.2(3), S2−Rh1−C18 100.3(2), S2−Rh1−C19 92.2(3), S1−Rh2−C22 85.7(3), S1−Rh2−C35 166.1(3), S1−Rh2−C36 155.1(3), S1−Rh2−C39 90.8(3), S1−Rh2−C40 94.8(4). Thermal ellipsoids are set at a 50 % probability level. The B(Ar^F^)_4_^−^ anions, and hydrogen atoms were omitted for clarity.

While the reaction of **Rh5** with AgBF_4_ did not result in the clean formation of the cationic BF_4_^−^ complex, the reaction of both **Rh1** and **Rh5** with NaB(Ar^F^)_4_ yielded the cationic analogues **Rh6** and **Rh7** (Scheme 2), which were isolated as microcrystalline, orange‐coloured solids in 72 % and 78 % yield respectively. The solid‐state structure of **Rh7** displays a dimeric structure with the Rh metal of one unit coordinated by a thiophene moiety of the second unit. Complex **Rh7** (Figure 3) crystallizes in the triclinic space group *P*‐1 with both the Rh atoms having a distorted square planar geometry. The metal to carbene distances C22‐Rh2 and C1‐Rh1 are 200.7 and 209.6 pm, respectively, and are in good agreement with similar reported complexes. A *trans* influence of the NHC on one of the double bonds of cyclooctadiene was observed in case of **Rh7**; the propyltrimetoxysilyl side arm was strongly disordered.

### Rh(III)−NHC complexes

**Scheme 3:**
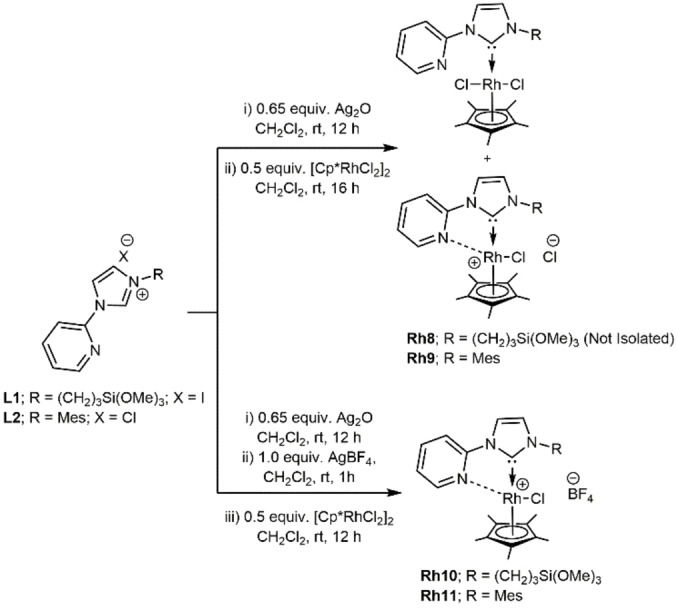
Synthesis of the Rh(III) (Cp*) NHC complexes **Rh8**–**Rh11**.

**Scheme 4:**
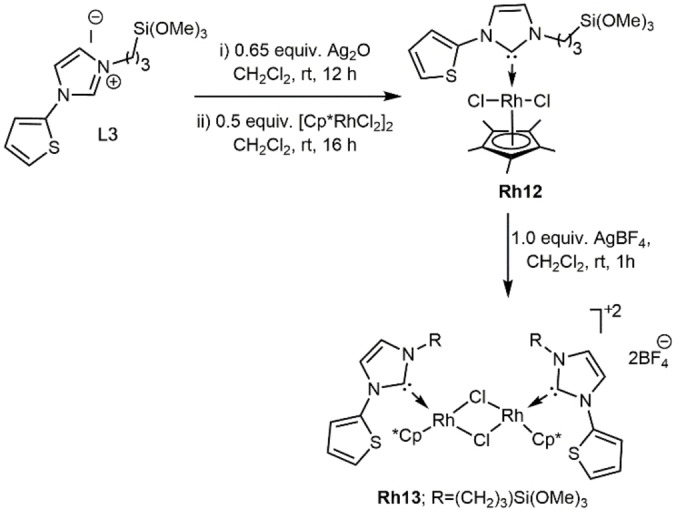
Synthesis of the Rh(III) (Cp*) NHC complexes **Rh12‐Rh13**.

As depicted in Scheme 3 and Scheme 4, reaction of the imidazolium salts **L1**–**L3** with Ag_2_O at room temperature yielded the desired Ag−NHC intermediates, which upon in situ transmetalation with half an equivalent of [Rh(Cp*)Cl_2_]_2_ yielded complexes **Rh8**, **Rh9** and **Rh12**. In case of the 1‐mesityl‐2‐ylimidazolium‐pyridine salt, reaction with [Rh(Cp*)Cl_2_]_2_ produced a mixture of monodentate and bidentate complexes. **Rh9** was obtained in pure form after purification by column chromatography. However, isolation of **Rh8** proved to be extremely difficult and all attempts to synthesize the cationic analogue **Rh10** in situ was also not fruitful. Hence, complexes **Rh10** and **Rh11** were synthesized alternatively from the silver intermediates as shown in Scheme 3. Moreover, as depicted in Scheme 4, **Rh12** was also prepared via transmetalation from **L3** and [Rh(Cp*)Cl_2_]_2_ in good yields (68 %). Notably, we were also able to synthesize the cationic analogue **Rh13** by reacting **Rh12** with AgBF_4_. Complexes **Rh9**–**Rh13** were obtained as yellow‐orange solids after recrystallization from a mixture of CH_2_Cl_2_ and diethyl ether. The carbon atoms bound to the Rh metal centres in **Rh9**–**Rh13**, that is, the carbene and Cp* carbon atoms, exhibit a Rh−C coupling with coupling constants in the range of 51–58 and 7 Hz, respectively.

**Figure 4:**
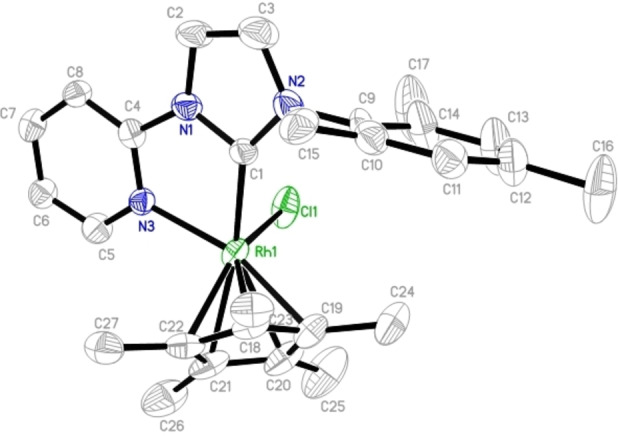
Single crystal X‐ray structure of **Rh9** (synthesized from the ligand containing a bromide anion). Selected bond lengths [pm] and bond angles [°]: Rh1−Cl1 241.1(2), Rh1−C1 204.0(8), Rh1−N3 211.8(6), Rh1−C18 217.0(7), Rh1−C19 214.9(9), Rh1−C20 223(1), Rh1−C21 218.7(9), Rh1−C22 213.8(7); Cl1−Rh1−C1 89.8(2), Cl1−Rh1−N3 85.9(2), C1−Rh1−N3 77.3(3). Thermal ellipsoids are set at a 50 % probability level. The anions, hydrogen atoms and co‐crystallized solvent molecules have been omitted for clarity.

The molecular structure of **Rh9** was confirmed by single‐crystal X‐ray analyses (Figure 4). **Rh9** synthesized from ligand **L2** was clean but, unfortunately, we could not obtain crystals suitable for X‐ray analysis. However, when ligand **L2** containing a chloride anion was replaced by one containing a bromide anion, the complex could be crystallized; nonetheless, the molecular structure featured a mixed set of halo ligands, with ∼25 % of the chloro positions occupied by bromo ligands. In the solid state, the rhodium is coordinated both to the NHC's C_2_ carbon and the pyridine nitrogen. It adopts a three‐legged piano‐stool‐type geometry as observed for other half‐sandwich complexes.^[18a]^ Complex **Rh9** (Figure 3) crystallizes in the triclinic space group *P‐1* with distorted octahedral geometry at the metal centre. The bite angle of the pyridyl‐NHC ligand is ca. 77.3° (C1−Rh−N3). The C1(NHC)−Rh and N−Rh bond lengths are 204.0 and 211.8 pm, respectively, which is in line with other complexes.^[18a]^

**Figure 5:**
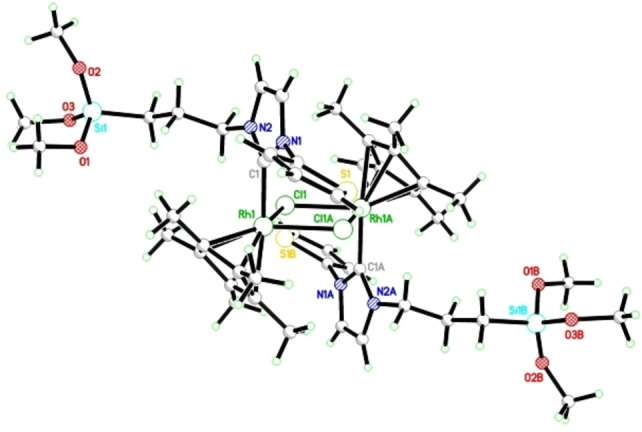
Single crystal X‐ray structure of **Rh13**. Selected bond lengths [pm] and bond angles [°]: Rh1−Cl1 245.5(2), Rh1−C1 206.0(5), Rh1−Cl2 244.1(2); Cl1−Rh1−C1 90.5(2), Cl1−Rh1−C1 80.0(2), C1−Rh1−Cl1 89.8(2), Rh1−Cl1−Rh1 100.0(6). Thermal ellipsoids are set at a 50 % probability level. The BF_4_^−^ anion and the co‐crystallized solvent molecules have been omitted for clarity.

The molecular structure of **Rh13** (Figure 5) displays a typical three‐legged piano stool geometry with the rhodium metal centre coordinated by the cyclopentadienyl, monodentate NHC and bridging chlorine atom. The Rh−C_carbene_ distances (206.0 pm) again lie in the typical range. As shown in the Figure 5, the two rhodium centers are connected by the bridging chloro ligands. Complex **Rh13** crystallizes in a triclinic crystal system in the space group *P‐1* with both the metal centres adopting the distorted octahedral geometry. The BF_4_ anions and the propyl trimethoxysilyl sidearm were strongly disordered.

### Hydrosilylation of terminal alkynes

**Scheme 5:**
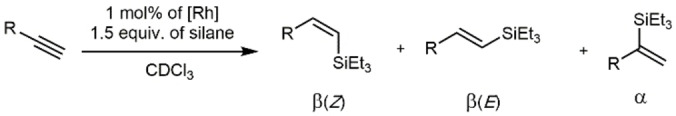
Hydrosilylation of terminal alkynes.

First, the Rh(I) NHC complexes **Rh1**–**Rh5** were used as catalysts in the hydrosilylation of phenylacetylene and 1‐octyne using HSiMe_2_Ph and triethylsilane as silanes (Scheme 5, Figure S1, Table S1, Supporting Information). Reactions were conducted in CDCl_3_ at 60 °C with a standard catalyst loading of 1 mol % and monitored by ^1^H NMR and GC‐MS using *t*‐butylbenzene and *n*‐dodecane, respectively, as internal standards. With all complexes, conversions ranged from 82–100 %. Using phenylacetylene, a clear preference for the thermodynamically more stable β(*E*)‐isomer in the range of 40–100 % was observed (Figure S1, Supporting Information). In addition, particularly the use of **Rh2**–**Rh5** resulted in the formation of significant amounts of the β(*E*) and α‐isomer. Using 1‐octyne and HSiMe_2_Ph, β(*E*) selectivity was reduced, too, yielding substantial amounts of the β(*Z*), along with minor amounts (≤5 %) of the *anti*‐Markovnikov product (α‐isomer) owing to the increased bulkiness of the silane partner. Follow‐up of the reaction of phenyl acetylene and HSiMe_2_Ph in CDCl_3_ by ^1^H NMR displayed only the formation of the β(*E*)‐isomer during the reaction, which rules out the formation of β(*Z*)‐isomer as an intermediate. Notably, for all complexes except for **Rh4**, no polymerization or dehydrogenative silylation was observed. However, hydrosilylation of 1‐octyne with HSiMe_2_Ph catalysed by **Rh6** and **Rh7** (Table 1, entries 9 and 10) displayed a similar selectivity in line with **Rh3** but reaction times were quite extended (5 h and 8 h respectively). Moreover, terminal alkynes bearing hydroxy, chloro, methyl and methoxy groups were tolerated (Table 1, entries 14, 15, 18 and 20) when the reactions were performed with **Rh1**.

**Table 1:** Hydrosilylation of terminal alkynes catalysed by the Rh(I) NHC complexes **Rh1‐Rh7**.

#	Substrate	Catalyst, time^[a]^	Conv. [%]	β(*Z*) [%]	β(*E*) [%]	α [%]
1	1‐Hexyne	**Rh1**, 2 h	100	45	55	–
2		**Rh3**, 2 h	100	43	52	5
3		**Rh5**, 3 h	96	67	27	6
4	1‐Octyne	**Rh1**, 2 h	100	28	72	–
5		**Rh2**, 2 h	100	63	36	1
6		**Rh3**, 2 h	100	38	58	4
7		**Rh4**, 2 h	100	57	39	4
8		**Rh5**, 3 h	90	67	28	5
9		**Rh6**, 5 h	100	58	22	20
10		**Rh7**, 8 h	86	53	29	18
11	1‐Nonyne	**Rh1**, 2 h	100	43	57	–
12		**Rh3**, 2 h	100	60	31	9
13		**Rh5**, 3 h	82	62	32	6
14	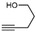	**Rh1**, 2 h	47	60	32	8
15	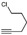	**Rh1**, 2 h	>99	65	35	–
16^[b]^	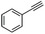	**Rh1**, 3 h	100	1	98	1
17		**Rh5**, 8 h	89	9	71	20
18^[b]^	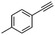	**Rh1**, 3 h	100	1	98	1
19		**Rh5**, 6 h	100	10	68	22
20^[b]^	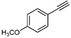	**Rh1**, 3 h	100	1	98	1
21		**Rh5**, 6 h	>99	12	68	20

[a] Unless noted otherwise, all the reactions were performed employing 1.0 equiv. of alkynes, 1.5 equiv. of dimethylphenylsilane, 1 mol % of Rh catalyst, 0.5 mL of CDCl_3_ at 60 °C. [b] Triethylsilane was used at 60 °C.

**Scheme 6:**
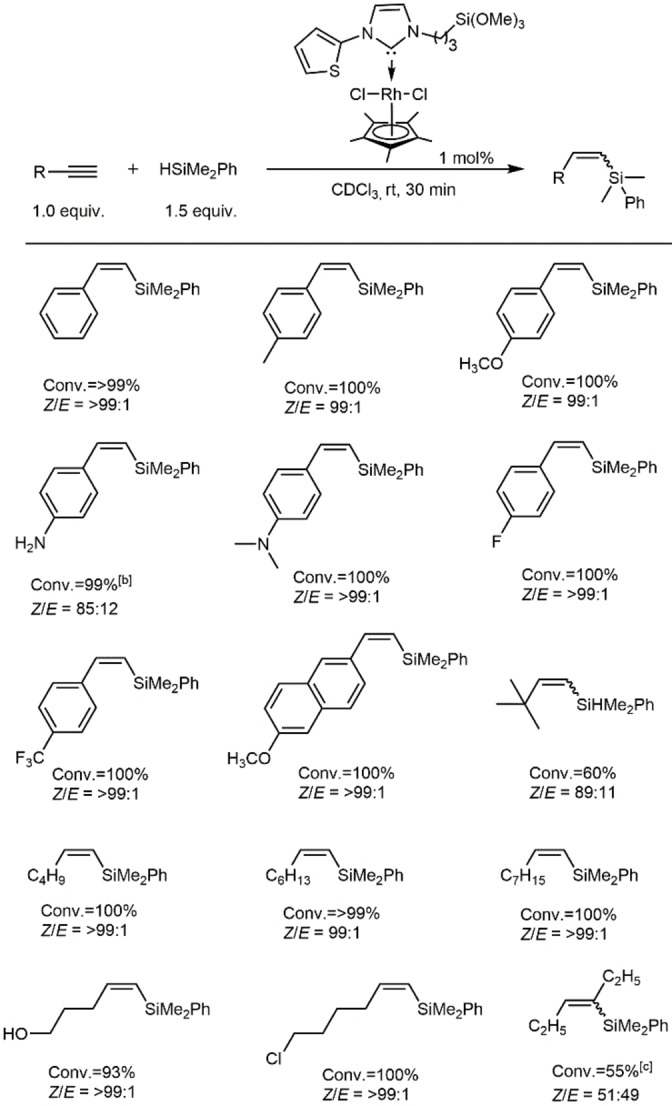
Substrate scope for alkyne hydrosilylation. [a] Unless noted otherwise, all the reactions were performed employing 1.0 equiv. of alkyne, 1.5 equiv. of dimethylphenylsilane, 1 mol % of Rh catalyst, 0.5 mL of CDCl_3_ at 25 °C. *Z*/*E* ratios were determined by ^1^H NMR spectroscopy; [b] reaction was performed for 3 h; [c] reaction was done at 60 °C for 3 h.

In contrast to the Rh(I) NHC complexes, the hydrosilylation reactions with Rh(III) Cp* NHC complexes could be performed at room temperature. Catalysts **Rh9**–**Rh13** (Table 2) were highly active yielding exclusively the corresponding β(*Z*)‐isomers as observed for other Rh(III) complexes^[18b]^ with full conversion to the desired products. However, **Rh9** and **Rh12** were found to be the slightly superior among the series. **Rh12** was therefore also tested for other substrates. The set of aromatic alkynes that undergo Rh‐catalysed *Z*‐selective hydrosilylation is shown in Scheme 6. In general, a wide range of ethynylarenes containing electronically and sterically different aryl groups reacted smoothly in the presence of 1 mol % of **Rh12** and HSiMe_2_Ph at room temperature, affording the corresponding *Z*‐vinylsilanes with complete conversion and excellent stereoselectivities (*Z*/*E*=89 : 11 to >99 : 1). This *Z*‐selective hydrosilylation showed good functional group tolerance for a wide range of reactive groups including ether, trifluoromethyl, halogen, unprotected hydroxyl, unprotected primary aniline and NMe_2_ moieties, which were all are compatible with the reaction conditions. The conversion vs. time profiles for the hydrosilylation of the aromatic alkynes were all similar except for 4‐aminophenylacetylene, which reacted much slower, yet reaching 99 % conversion after 3 h (Figure S7, Supporting Information).

**Table 2:** Hydrosilylation of terminal alkynes catalysed by the Rh(III) Cp* NHC complexes **Rh9**–**Rh13**.

#	Alkyne	Catalyst, time^[a]^	Conv. [%]	β(Z) [%]	β(*E*) [%]	α [%]
1	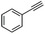	**Rh9**, 30 min	98	>99	–	<1
2^[b]^		**Rh10**, 2 h	100	99	1	–
3^[b]^		**Rh11**, 2 h	92	95	5	
4		**Rh12**, 10 min	>99	100	–	–
5		**Rh13**, 40 min	>99	99	1	–

[a] Unless noted otherwise, all the reactions were performed employing 1.0 equiv. of alkyne, 1.5 equiv. of dimethylphenylsilane, 1 mol % of Rh catalyst, 0.5 mL of CDCl_3_ at 25 °C. [b] reactions were performed at 60 °C.

In addition, bulky *t*‐butylacetylene was also tested for the hydrosilylation; the reaction yielded 89 % of the β(*Z*)‐isomer. An attempt to reduce the catalyst loading to 0.1 mol % led to increased reaction times of up to 2 h to reach full conversion, yet without any effect on the selectivity.

Notably, the current catalytic system was also successfully applied to the hydrosilylation of internal alkynes. Thus, hydrosilylation of 3‐hexyne with HSiMe_2_Ph in the presence of **Rh12** furnished a 1 : 1 mixture of the *E* and *Z* isomer.

While **Rh13** is dimeric in the solid state, the active catalyst must be expected to be monomeric to fulfil the 18‐electron rule. With **Rh9**, similar conversions and selectivities were observed in case triethylsilane instead of dimethylphenylsilane was used in hydrosilylation (Figure S8–S11, Supporting Information).

### Immobilization of Rh(I) NHC and Rh(III) Cp* NHC catalysts on mesoporous SBA‐15

**Scheme 7:**
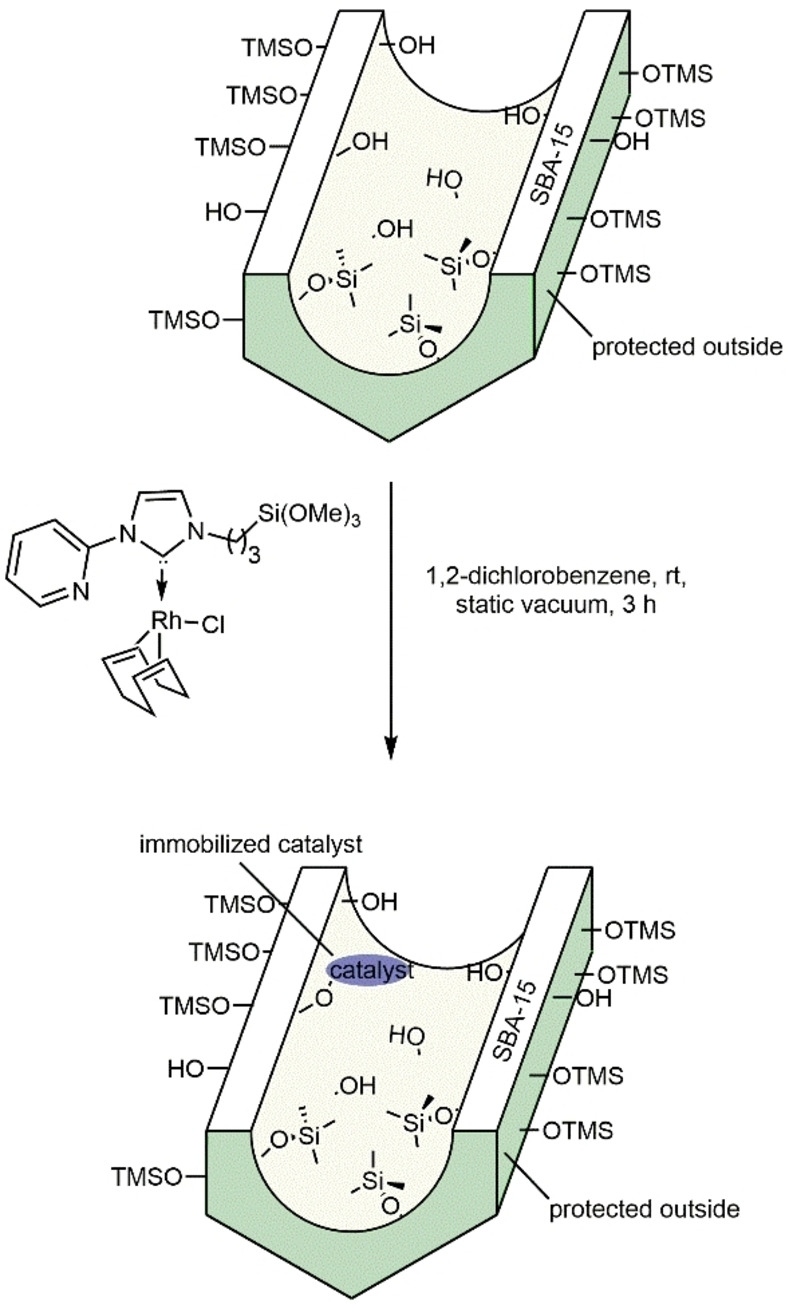
Immobilization of Rh‐catalysts inside mesoporous SBA‐15 exemplified for **Rh1**.

Two different SBA‐15 materials with defined average pore diameters of 5.0 and 6.2 nm, respectively, referred to as **SBA‐15_5.0 nm_** and **SBA‐15_6.2 nm_** were used for the immobilization of **Rh1**, **Rh3**, **Rh5**, **Rh12** and **Rh13** using the trimethoxysilyl moiety (Scheme 7).

**Figure 6:**
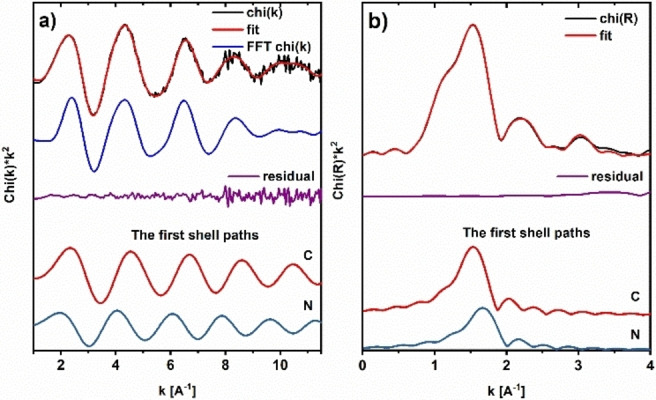
EXAFS fitting results for Rh3@SBA‐15*_6.2 nm_. a) Fitted k^2^‐weighted EXAFS signal (red) along with Fourier‐filtered signal (blue), residual signal (data‐fit, violet) and first shell paths (bottom). b) Fourier‐transformed k^2^‐weighted EXAFS with fit (red), residual function (violet) and first shell paths.

A recently published synthetic protocol that allows for the selective immobilization inside but not outside the pores was used.^[19]^ The Rh content, determined by ICP‐OES, was 9.7, 18.6, 2.9 and 16.4 μmol/g for **Rh1@SBA‐15_5.0 nm_**, **Rh1@SBA‐15_6.2 nm_** and **Rh3@SBA‐15_5.0 nm_** and **Rh3@SBA‐15_6.2 nm_** respectively. For **Rh5@SBA‐15_6.2 nm_**, **Rh12@SBA‐15_6.2 nm_** and **Rh13@SBA‐15_6.2 nm_** a loading of 3.6, 8.9 and 5.6 μmol/g was determined. For comparison, we also immobilized **Rh1** on unmodified **SBA‐15_6.2 nm_**, labelled as Rh1@SBA‐15*_6.2 nm_. ICP‐OES measurements revealed a high Rh‐loading of 204.1 μmol/g, due to the immobilization of **Rh1** both inside and outside the mesopores of silica. Rh K‐edge EXAFS analysis of Rh3@SBA‐15*_6.2 nm_ was conducted to confirm that the catalyst remains intact upon immobilization on SBA‐15. The experimental spectra and the fit results are shown in Figure 6; structural parameters are summarized in Table 3. Six atoms are found in the first coordination shell, three at a distance of 2.06 Å and three at 2.20 Å. Both values are in the range of Rh−C and Rh−N bond distances,^[23]^ and in agreement with the crystal structure. As EXAFS cannot distinguish between light ligands, the type of atoms remains unknown, while the description of scattering paths in Table 3 refers to the parent catalyst **Rh3**. The differences in bond lengths in the first coordination shell did not exceed 2 %, while the maximal difference in the probed vicinity of the Rh atom was 18 %, indicating structural distortion of the complex induced by the support. In conclusion, the structure of the complex remains intact, but structural effects of the confinement are reflected by changes in the bond lengths. Back scatterers of the support could not be detected in the EXAFS analysis.

**Table 3:** EXAFS fitting results for Rh3@SBA‐15*_6.2 nm_, including refined coordination numbers (N), Debye‐Waller factors (σ^2^), crystal structure bond lengths (R_cryst_ ), refined bond lengths (R+ΔR) and relative change of bond lengths upon EXAFS refinement.

Path	N	σ^2^/Å^2^	R_cryst_/Å	R+ΔR/Å	(R+ΔR)/R_cryst_/%
Rh−C	3.1(1)	0.0041(12)	2.025	2.061(9)	102 %
Rh−N	3.4(1)	0.0044(13)	2.110	2.198(10)	102 %
Rh−C	0.8(2)	0.0055(19)	2.157	2.491(26)	118 %
Rh−N	1.0(4)	0.0074(26)	2.861	2.780(32)	97 %
Rh−C	1.5(3)	0.0062(18)	3.003	2.917(26)	97 %
Rh−C	1.8(4)	0.0064(19)	3.119	3.074(32)	99 %
Rh−C‐N	3.9(8)	0.0083(27)	3.312	3.760(23)	114 %
Rh−N	8.4(1.6)	0.0198(64)	4.258	4.590(29)	108 %

### Hydrosilylation reactions and confinement effects

After the selective immobilization^[24]^ of both Rh(I) and Rh(III) NHC complexes inside the mesopores of silica, the supported catalysts were used in the hydrosilylation of terminal alkynes; results were compared to those obtained with the corresponding homogenous catalysts. In contrast to their homogeneous counterparts, the heterogenous catalysts **Rh1@SBA‐15_6.2 nm_**, **Rh3@SBA‐15_6.2 nm_** and **Rh5@SBA‐15_6.2 nm_** yielded predominantly the β(*Z*)‐isomer (up to 97 %, Table 4). Notably, the rhodium catalysts **Rh1**, **Rh3** and **Rh5** supported on modified SBA‐15_6.2 nm_ material showed much better β(*Z*) selectivity (Figure S12, Supporting Information) in the hydrosilylation reaction than the non‐modified silica‐supported analogue Rh1@SBA‐15*_6.2 nm_ (Table 4, entries 2, 7 and 13). This clearly points to a (steric) confinement effect of the mesopores onto the Rh‐catalysts immobilized therein: the exclusive binding of both **Rh1** and **Rh3** inside the mesopores of SBA‐15_6.2 nm_ allows for high β(*Z*)‐selectivity while catalysts supported outside the pores lack the necessary steric environment and cannot provide the same selectivity. Similarly, **Rh5** immobilized on modified SBA‐15_6.2 nm_ showed high β(*Z*) selectivity with aliphatic alkynes, too (Table 4, entries 9 and 15). However, diffusion constraints of the substrates inside the SBA‐15 pores limit activity, leading to longer reaction times.

**Table 4:** Hydrosilylation of terminal alkynes catalysed by the SBA‐15 supported Rh(I) NHC and Rh(III) Cp* NHC complexes **Rh1@SBA‐15_6.2nm_**, Rh1@SBA‐15*_6.2nm__,_ **Rh3@SBA‐15_6.2 nm_**, **Rh5@SBA‐15_6.2 nm_**, **Rh12@SBA‐15_6.2 nm_**, **Rh13@SBA‐15_6.2 nm_**.

#	Substrate	Rh@SBA‐15_6.2 nm_, time^[a]^	Conv. [%]	β(*Z*) [%]	β(*E*) [%]	α [%]
1	1‐Hexyne	**Rh1@SBA‐15**, 40 h	95	95	3	2
2		Rh1@SBA‐15*, 12 h	90	72	28	–
3^[b]^		**Rh1@SBA‐15**, 48 h	94	95	2	3
4		**Rh3@SBA‐15**, 32 h	95	93	7	–
5^[b]^		**Rh3@SBA‐15, 48 h**	90	92	6	2
6	1‐Octyne	**Rh1@SBA‐15**, 40 h	95	92	5	3
7		Rh1@SBA‐15*, 12 h	90	77	20	3
8		**Rh3@SBA‐15**, 32 h	100	93	5	2
9		**Rh5@SBA‐15**, 26 h	63	90	9	1
10		**Rh12@SBA‐15**, 3 h	97	99	1	–
11		**Rh13@SBA‐15**, 4 h	47	99	1	–
12	1‐Nonyne	**Rh1@SBA‐15**, 40 h	95	97	3	–
13		Rh1@SBA‐15*, 12 h	90	76	24	–
14		**Rh3@SBA‐15**, 32 h	95	92	6	2
15		**Rh5@SBA‐15**, 26 h	90	87	10	3
16	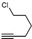	**Rh1@SBA‐15**, 24 h	97	90	10	–
17	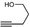	**Rh1@SBA‐15**, 24 h	35	77	23	–
18	3‐Hexyne	**Rh12@SBA‐15**, 4 h	>99	2	98	–
19^[c]^	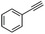	**Rh1@SBA‐15**, 48 h	60	18	73	9
20		**Rh12@SBA‐15**, 3 h	94	100	–	–
21		**Rh13@SBA‐15**, 4 h	69	100	–	–
22^[c]^	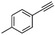	**Rh1@SBA‐15**, 48 h	54	14	75	11
23		**Rh12@SBA‐15**, 3 h	>99	99	1	–
24		**Rh13@SBA‐15**, 4 h	60	99	1	–
25^[c]^	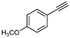	**Rh1@SBA‐15**, 48 h	57	9	85	6
26		**Rh12@SBA‐15**, 3 h	>99	99	1	–
27		**Rh13@SBA‐15**, 3 h	78	100	1	–
28	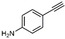	**Rh12@SBA‐15**, 4 h	93	100	–	–
29		**Rh13@SBA‐15**, 4 h	58	100	–	–

[a] Unless noted otherwise, all the reactions were performed employing 1.0 equiv. of alkyne, 1.5 equiv. of dimethylphenylsilane, 0.5 mol % of Rh catalyst, 0.5 mL of CDCl_3_ at 60 °C. SBA‐15_6.2 nm_ was used throughout. [b] SBA‐15_5.0 nm_ was employed. [c] 1.5 equiv. of triethylsilane was employed.

On the expected lines, **Rh1@SBA‐15_5.0 nm_** and **Rh3@SBA‐15_5.0 nm_** (Table 4, entries 3 and 5) also showed excellent β(*Z*)‐selectivity up to 95 % with aliphatic but not for aromatic alkynes (Table 4, entries 19, 22 and 25). Notably, the observed confinement effect was also observed in presence of functional groups such as chlorine and hydroxyl groups (Table 4, entries 16 and 17). In addition, a confinement effect could even be observed in the hydrosilylation of 4‐aminophenylacetylene under the action of a supported Rh(III) Cp* NHC catalyst. While homogeneous **Rh12** delivered only 85 % β(*Z*) selectivity (Scheme 2), the heterogenous catalyst **Rh12@SBA‐15_6.2 nm_** again yielded exclusively the β(*Z*) isomer (Table 4, entry 29). Overall, β(*Z*) selectivity exceeded the one previously reported supported [Rh_4_(CO)_12_] clusters by far.^[25]]^

### Time‐dependent selectivity

Notably, neither **Rh12**, nor **Rh12@SBA‐15_6.2nm_**_,_ showed any β(*Z*) to β(*E*) isomerization, neither for phenylacetylene nor for 1‐octyne within reasonable times after the complete consumption of the alkyne. The monitoring of hydrosilylation of phenylacetylene with HSiMe_2_Ph at 298 K showed that the reaction was completed within 10 min and 40 min for **Rh12** and **Rh12@SBA‐15_6.2nm_**, respectively, yielding exclusively the β(*Z*) isomer (Figure S13, Supporting Information). Similar was observed for aliphatic alkynes; again, no noticeable β(*Z*) to β(*E*) isomerization was observed as shown for for 1‐octyne (Figure S14, Supporting Information). By contrast, in the reaction of 4‐ethynylanisole with HSiMe_2_Ph, β(*Z*) to β(*E*) isomerization was observed, though only after prolonged rection times (Figure S15–S17, Supporting Information).

### Mechanism

**Scheme 8:**
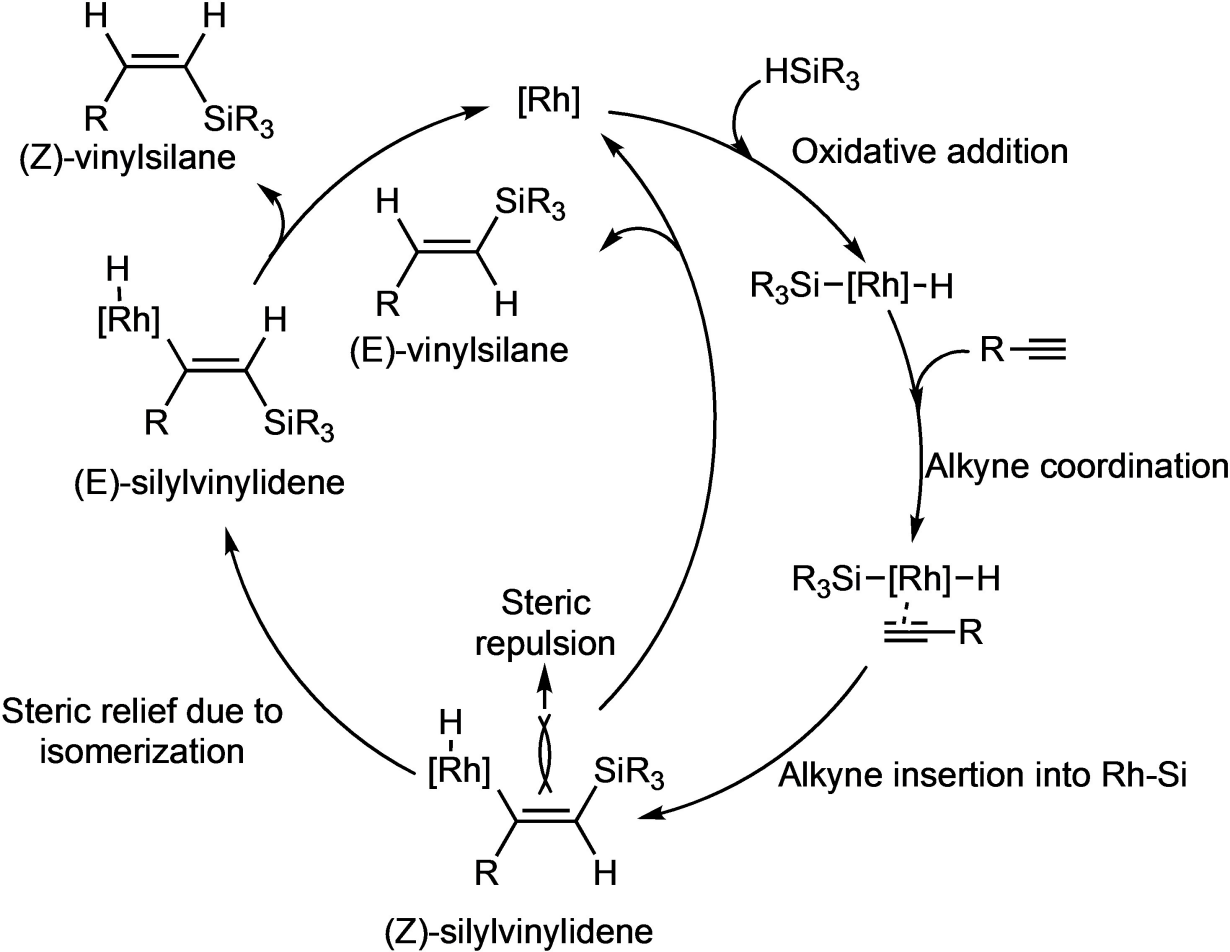
Modified Chalk‐Harrod mechanism[^8a^, ^14g^] for alkyne hydrosilylation.

Based on the above‐observed results and earlier reports on the modified Chalk‐Harrod mechanism,^[8a]^ a plausible mechanism for regioselectivity that accounts for the observed formation of all the three isomers catalysed by Rh(I) NHC complexes is depicted in Scheme 8. Initial rhodium hydride formation is followed by alkyne coordination. Insertion of the alkyne into the Rh−Si bond yields the (*Z*)‐silylvinylidene intermediate and, subsequently, the *E*‐vinylsilane. However, the (*Z*)‐silylvinylidene intermediate can isomerize to the thermodynamically favourable (*E*)‐silylvinylidene owing to the steric repulsion between the substituents at the rhodium centre and the silyl group. At this point, the steric confinement, whether provided by the Cp* and NHC ligand in the Rh(III) complexes or by the pore wall in case of the supported Rh(I) complexes, becomes effective. Particularly, the increased β(*Z*)‐selectivity of the supported catalysts in comparison to their homogenous counterparts is attributed to an enhanced steric congestion inside the mesopores in which the pore wall exerts additional steric stress on the metal centre.

### Recyclability and leaching

To demonstrate the sustainability of the catalytic system, five consecutive hydrosilylation reactions of 1‐octyne with dimethylphenylsilane were carried out using the same batch of an immobilized Rh‐NHC catalyst, i. e., **Rh12@SBA‐15_6.2 nm_**. At the end of each run, the silica‐bound catalyst was recovered by simple filtration, washed repeatedly with chloroform, dried *in vacuo*, and reused in the next reaction. Gratifyingly, the catalytic performance of **Rh12@SBA‐15_6.2 nm_** remained constant over the course of the experiments, with no detrimental effect on selectivity and reactivity (Figure S18, Supporting Information). Noteworthy, recycling of the catalyst neither required purification nor any reactivation steps. Leaching of the catalyst was also tested by a hot filtration test (80 °C) under similar reaction conditions. The reaction mixture was filtered through celite, and the filtrate was analysed by ICP‐OES. ICP analysis demonstrated no rhodium leaching.

## Conclusions

β(*Z*)‐Selectivity in the hydrosilylation of 1‐alkynes is strongly governed by steric effects at the metal centre. Such confinement can to some extent be generated with Rh(I) complexes bearing a chelating N‐heterocyclic carbene (NHC) in combination with bulky silanes. However, only Rh(III) pentamethylcyclopentadienyl complexes containing a chelating NHC provide sufficient steric confinement and allow for high β(*Z*)‐selectivity, both with aromatic and aliphatic alkynes. Alternatively, artificial confinement generated by the immobilization of Rh(I) NHC complexes in mesoporous supports allows for high β(*Z*)‐selectivity, too. In selected cases, the high β(*Z*)‐selectivity of Rh(III) Cp* NHC complexes can also be further increased by immobilization inside mesoporous SBA‐15.

## Experimental Section

Deposition Number(s) 2082933 (**Rh3**), 2103708 (**Rh7**) 2082934 (**Rh9**) and 2086994 (**Rh13**) contain(s) the supplementary crystallographic data for this paper. These data are provided free of charge by the joint Cambridge Crystallographic Data Centre and Fachinformationszentrum Karlsruhe Access Structures service.

Experimental procedures and spectral data for all the new complexes are available in the Supporting Information.

## Conflict of interest

The authors declare no conflict of interest.

## Supporting information

As a service to our authors and readers, this journal provides supporting information supplied by the authors. Such materials are peer reviewed and may be re‐organized for online delivery, but are not copy‐edited or typeset. Technical support issues arising from supporting information (other than missing files) should be addressed to the authors.

Supporting Information
