# Supplementary material for: Rh(I)/(III)‐N‐Heterocyclic Carbene Complexes: Effect of Steric Confinement Upon Immobilization on Regio‐ and Stereoselectivity in the Hydrosilylation of Alkynes
Source: Chemistry. 2021 Nov 8;27(68):17220–9. doi: 10.1002/chem.202103099 (PMC9299010; doi:10.1002/chem.202103099)
Supplement: Supplementary file 1 — Supporting Information [file CHEM-27-17220-s001.pdf]

Chemistry–A European Journal

Supporting Information

Rh(I)/(III)-N-Heterocyclic Carbene Complexes: Effect of Steric Confinement Upon Immobilization on Regio- and Stereoselectivity in the Hydrosilylation of Alkynes

Chemistry–A European Journal

Supporting Information

Rh(I)/(III)-N-Heterocyclic Carbene Complexes: Effect of Steric Confinement Upon Immobilization on Regio- and Stereoselectivity in the Hydrosilylation of Alkynes

Contents

Experimental Section	S2
Kinetic Studies	S13
NMR Spectrum of Complexes	S23
High-Resolution Mass Spectrum of Complexes	S40
NMR Spectrum of Hydrosilylated Products	S54
Single-Crystal X-ray Data	S70

Experimental Section

General: Unless noted otherwise, all reactions were performed under the exclusion of air and moisture by standard Schlenk techniques. Reactions involving metal complexes were performed in a nitrogen-filled glove box (MBraun Labmaster 130). Glassware was stored at 120 °C overnight and cooled in an evacuated antechamber. ^{1}H, ^{13}C, ^{19}F NMR spectra were recorded on a Bruker Avance III 400 spectrometer at 400, 101 and 376 MHz, respectively. Chemical shifts are reported in ppm from tetramethylsilane with the solvent resonance resulting from residual solvent protons ($CDCl_3$: 7.26 ppm, CD_2Cl_2 5.32 ppm) as reference. Data are reported as follows: chemical shift (δ), multiplicity (s = singlet, d = doublet, t = triplet, q = quartet, br = broad, m = multiplet, dd = doublet of doublets, dt = doublet of triplets, td = triplet of doublets, tt = triplet of triplets, qq = quartet of quartets, ddd = doublet of doublet of doublets), coupling constants (Hz) and integration. Single-crystal X-ray analysis was performed with a Bruker Kappa APEXII Duo diffractometer with Mo Kα irradiation at the Institute of Organic Chemistry, University of Stuttgart, Germany. High-resolution mass spectra were recorded at the Institute of Organic Chemistry, University of Stuttgart, Germany. CH_2Cl_2, diethyl ether, toluene, tetrahydrofuran (THF) and pentane were dried by using an MBraun SPS-800 solvent purification system with alumina drying columns and stored over 4 Å Linde type molecular sieves. Deuterated solvents were filtered over activated alumina and stored over 4 Å Linde type molecular sieves inside the glove box. CCDC 2082933 (**Rh3**), 2103708 (**Rh7**) 2082934 (**Rh9**) and 2086994 (**Rh13**) contain the supplementary crystallographic data for this paper. These data can be obtained free of charge from The Cambridge Crystallographic Data Centre by The Cambridge Crystallographic Data Centre.

1-(Pyrid-2-yl)-3-(3-(trimethoxysilyl)propyl)-1*H*-imidazol-3-ium iodide, L1: A mixture of 2-(1*H*-imidazol-1-yl)pyridine (250 mg, 1.72 mmol, 1.0 equiv), 3-iodopropyltrimethoxysilane (1.5 g, 5.16 mmol, 3.0 equiv) and 5 mL of acetonitrile were placed in a pressure tube, which was sealed and heated to 100 °C for 36 h. The reaction mixture was then cooled to room temperature and all the volatiles were removed under reduced pressure. The residue was washed repeatedly with pentane (3 x 10 mL) and diethyl ether (3 x 10 mL); then, all solids were filtered off and the solids were dried under

vacuum. The product was isolated as white coloured solid. Yield: 730 mg (97.3%). **^{1}H NMR** (400 MHz, $CDCl_3$) δ 10.77 (s, 1H), 8.31-8.29 (ddd, J_1 = 4.8, J_2 = 1.9, J_3 = 0.9 Hz, 1H), 8.20-8.18 (dt, J_1 = 8.2, J_2 = 0.9 Hz, 1H), 8.14-8.13 (m, 1H), 7.83-7.79 (ddd, J_1 = 8.3, J_2 = 7.5, J_3 = 1.8 Hz, 1H), 7.56 (dd, J_1 = 2.2, J_2 = 1.6 Hz, 1H), 7.26 (ddd, J_1 = 7.5, J_2 = 4.8, J_3 = 0.8 Hz, 1H), 4.40-4.37 (t, J = 7.1 Hz, 2H), 3.33 (s, 9H), 1.93-1.89 (m, 2H), 0.51-0.47 (m, 2H). **^{13}C NMR** (101 MHz, $CDCl_3$) δ 149.2, 145.8, 140.5, 134.5, 125.3, 123.2, 119.1, 114.9, 52.3, 50.9 24.2, 5.9. Elemental analysis calcd. for $C_{14}H_{22}IN_3O_3Si$: C 38.63, H 5.09, N 9.65. Found: C 38.56, H 5.20, N 9.54. **HRMS** calculated for $C_{14}H_{22}N_3O_3Si$: 308.1425, found: 308.1421.

1-(Thienyl)-3-(3-(trimethoxysilyl)propyl)-1*H*-imidazol-3-ium iodide L3: 1-(Thienyl)-1*H*-imidazole (250 mg, 1.66 mmol, 1.0 equiv), 3-iodopropyltrimethoxysilane (966 mg, 3.32 mmol, 2.0 equiv) and 4 mL of acetonitrile were heated in a sealed tube for 24 h. All volatiles were removed under reduced pressure and the residue was repeatedly washed with diethyl ether and dried under vacuum to obtain a white-coloured solid. Yield: 697 mg (95%). **^{1}H NMR** (400 MHz, CD_2Cl_2) δ 10.53 (s, 1H), 7.67 (dd, J_1 = 3.8, J_2 = 1.5 Hz, 1H), 7.64 (dd, J_1 = 2.1, J_2 = 1.6 Hz, 1H), 7.60 (t, J = 1.9 Hz, 1H), 7.41 (dd, J_1 = 5.5, J_2 = 1.4 Hz, 1H), 7.12 (dd, J_1 = 5.5, J_2 = 3.9 Hz, 1H), 4.55 (t, J = 7.2 Hz, 2H), 3.56 (s, 9H), 2.13-2.05 (m, 2H), 0.73-0.69 (t, J = 7.2 Hz, 2H). **^{13}C NMR** (101 MHz, CD_2Cl_2) δ 136.3, 134.7, 127.1, 125.2, 123.4, 123.2, 122.4, 52.3, 50.7, 24.1, 5.9. Elemental analysis calcd. for $C_{13}H_{21}IN_2O_3SSi$: C 35.46, H 4.81, N 6.36. Found: C 35.50, H 4.927, N 6.10. **HRMS** calculated for $C_{13}H_{21}N_2O_3SSi$: 313.1037, found: 313.1036.

[Chloro(1-{pyrid-2-yl}-3-{3-{trimethoxysilyl}propyl}-imidazol-2-ylidene)(η^4-1,5-cyclooctadiene)rhodium(I)], Rh1: A chilled suspension of LiHMDS (42.3 mg. 0.25 mmol, 1.1 equiv) in 1 mL THF was added to a chilled (-34 °C) suspension of 1-(pyrid-2-yl)-3-(3-(trimethoxysilyl)propyl)-1*H*-imidazol-3-ium iodide (100 mg, 0.23 mmol, 1.0 equiv) in 1 mL THF. The resulting mixture was stirred at room temperature for 1 h and cooled to -34 °C. $[Rh(COD)Cl]_2$ (56.7 mg; 0.12 mmol, 0.5 equiv) suspended in 2 mL of THF was then added at -34 °C. The resulting reaction mixture was stirred at room temperature for 2h, then concentrated under reduced pressure and extracted with CH_2Cl_2. The CH_2Cl_2 solution was filtered through celite and evaporated to dryness. The residue was then

washed with pentane (5 mL), diethyl ether (2 x 5 mL) and dried to obtain an orange-coloured solid. Yield: 77% (96 mg). **^{1}H NMR** (400 MHz, CD_2Cl_2) δ 8.62-8.60 (m, 1H), 8.18 (dd, J_1 = 5.4, J_2 = 1.7 Hz, 1H), 7.99 (ddd, J_1 = 8.3, J_2 =7.4, J_3 =1.7 Hz, 1H), 7.82 (d, J = 2.2 Hz, 1H), 7.33 (ddd, J_1 = 7.4, J_2 =5.3, J_3 =1.1 Hz, 1H), 7.07 (d, J = 2.2 Hz, 1H), 5.00 (br, 2H), 4.12-4.09 (m, 2H), 3.57 (s, 11H), 2.37 (br, 4H), 2.06-1.97 (br, 6H), 0.74-0.70 (m, 2H). **^{13}C NMR** (101 MHz, CD_2Cl_2) δ 181.2 (d, $J_{Rh\text{-}C}$ = 49.5 Hz), 151.4, 147.7, 138.5, 122.3, 122.1, 117.5, 113.9, 94.2, 68.6, 52.7, 50.6, 32.7, 29.0, 23.8, 6.4. Despite numerous efforts satisfactory elemental analytical data could not be achieved. **HRMS** calculated for $[M]^+$ $C_{22}H_{33}N_3O_3RhSi$: 518.1341, found: 518.1341.

Chloro[((1-(pyrid-2-yl)-3-mesityl)-imidazol-2-ylidene))(η^4-1,5-cyclooctadiene)rhodium(I)], Rh2: 1.1 equivalents of LiHMDS (53.54 mg; 0.32 mmol, 1.1 equiv) were added at -34 °C to a suspension of **L2** (100 mg; 0.29 mmol, 1 equiv) in 2 mL THF, and the mixture was stirred at room temperature for 1 h. Then the reaction was cooled to -34 °C and a prechilled solution of 0.5 equiv of $[Rh(COD)Cl]_2$ (71.50 mg, 0.145 mmol, 0.5 equiv) in 2 mL THF was added and the resultant reaction suspension was stirred at room temperature for a further 2 h. All volatiles were removed under vacuum, the residue was dissolved in CH_2Cl_2, and 3 mL of diethyl ether were added. The mixture was filtered through celite, and the filtrate was kept at -34 °C overnight to obtain **Rh2** as yellow-coloured solid. Yield: 60 % (76 mg). **^{1}H NMR** (400 MHz, CD_2Cl_2) δ 9.18 (d, J = 2.2 Hz, 1H), 8.94-8.92 (m, 1H), 8.24-8.20 (ddd, J_1 = 8.4, J_2 =7.5, J_3 =1.6 Hz, 1H), 7.84 (d, J = 5.7 Hz, 1H), 7.47-7.43 (ddd, J_1 = 7.6, J_2 =5.6, J_3 =1.2 Hz, 1H), 7.00 (d, J = 0.7 Hz, 2H), 6.88 (d, J = 2.2 Hz, 1H), 4.98 (br, 2H), 3.89 (br, 2H), 2.34 (s, 3H), 2.29 (br, 4H), 2.12 (s, 6H), 2.01 (br, 4H). **^{13}C NMR** (101 MHz, CD_2Cl_2) δ 174.8 (d, $J_{Rh\text{-}C}$ = 54.54 Hz), 152.9, 147.4, 142.9, 140.5, 134.4, 133.8, 129.6, 124.6, 123.5, 119.4, 114.2, 97.9, 77.7, 32.3, 28.3, 21.0, 17.5. Elemental analysis calcd. for $C_{25}H_{29}ClN_3Rh$: C 58.89 H 5.73, N 8.24. Found: C 58.89, H 5.83, N 8.33. **HRMS** calculated for $[M]^+$ $C_{25}H_{29}N_3Rh$: 474.1411, found: 474.1411.

[(1-{Pyrid-2-yl}-3-{3-{trimethoxysilyl}propyl}-imidazol-2-ylidene)(η^4-1,5-cyclooctadiene)rhodium(I) tetrafluoroborate], Rh3: Rh1 (50 mg, 0.09 mmol, 1.0 equiv) was dissolved in 3 mL of CH_2Cl_2 and the solution was chilled to -34 °C. Next, a

suspension of $AgBF_4$ (17.6 mg, 0.09 mmol, 1.0 equiv) was slowly added at -34 °C, the mixture was stirred for 60 min, then filtered through celite and concentrated under reduced pressure. The residue was washed with pentane and dried under vacuum to obtain an orange-coloured foam. Yield: 67.6 % (37.2 mg). **^{1}H NMR** (400 MHz, CD_2Cl_2) δ 8.18-8.14 (ddd, J_1 = 8.4, J_2 =7.5, J_3 =1.6 Hz, 1H), 7.83-7.81 (m, 3H), 7.46-7.43 (m, 1H), 7.14 (d, J = 2.3 Hz, 1H), 5.03 (d, J = 5.7 Hz, 2H), 4.93-4.91 (dd, J_1 = 5.2, J_2 =2.5 Hz, 2H), 3.73-3.69 (m, 2H), 3.56 (s, 9H), 2.52-2.43 (m, 4H), 2.27-2.18 (m, 4H), 1.97-1.89 (tt, J_1 = 7.9, J_2 = 6.9 Hz, 2H), 0.68-0.64 (m, 2H). **^{13}C NMR** (101 MHz, $CDCl_3$) δ 173.6 (d, J_{Rh-C} = 54.5 Hz) 152.8, 147.6, 147.5, 143.2, 143.1, 123.6, 116.7, 112.2, 97.7, 97.6, 77.8, 77.7, 50.53, 50.5, 32.6, 28.5, 25.2, 6.1. **^{19}F NMR** (376 MHz, CD_2Cl_2) δ -152.68. Elemental analysis calcd. for $C_{22}H_{33}BF_4N_3O_3RhSi$.0.5 CH_2Cl_2: C 41.72 H 5.29, N 6.49. Found: C 41.83, H 5.07, N 6.89. **HRMS** calculated for $[M]^+$ $C_{22}H_{33}N_3O_3RhSi$: 518.1341, found: 518.1344. Crystals suitable for single X-ray structure analysis were obtained by layering diethyl ether over a saturated solution of **Rh3** in CH_2Cl_2.

[1-(Pyrid-2-yl)-3-mesityl)-imidazol-2-ylidene))(η^4-1,5-cyclooctadiene)rhodium(I) tetrafluoroborate], Rh4: Rh2 (50 mg, 0.10 mmol, 1.0 equiv) was dissolved in 3 mL of CH_2Cl_2, the solution was chilled to -34 °C, a suspension of $AgBF_4$ (19.5 mg, 0.1 mmol, 1.0 equiv) in 2 mL of CH_2Cl_2 was added at -34 °C and the mixture was stirred for 60 min. It was then filtered through celite and concentrated under reduced pressure. The residue was washed with pentane and dried to obtain an orange-coloured foam. Yield: 74% (40.4 mg). **^{1}H NMR** (400 MHz, CD_2Cl_2) δ 8.23-8.19 (ddd, J_1 = 8.3, J_2 =7.5, J_3 =1.7 Hz, 1H), 8.05 (d, J = 2.2 Hz, 1H), 7.98-7.96 (m, 1H), 7.84 (d, J = 5.7 Hz, 1H), 7.49-7.46 (ddd, J_1 = 7.6, J_2 =5.6, J_3 = 1.1 Hz, 1H), 7.01 (d, J = 0.7 Hz, 2H), 6.90 (d, J = 2.2 Hz, 1H), 5.03-5.01 (dd, J_1 =5.0, J_2 = 2.6 Hz, 2H), 3.94-3.92 (dd, J_1 = 5.2, J_2 =2.5 Hz, 2H), 2.34-2.26 (m, 7H), 2.12 (s, 6H), 2.02 (m, 4H). **^{13}C NMR** (101 MHz, CD_2Cl_2) δ 175.1 (d, J_{Rh-C} = 55.55 Hz), 152.6, 147.8, 143.2, 140.7, 134.3, 133.6, 129.7, 125.0, 123.9, 117.3, 112.5, 98.6, 98.5, 78.9, 78.8, 32.3, 28.3, 21.0, 20.9, 17.4, 17.3. **^{19}F NMR** (376 MHz, CD_2Cl_2) δ -152.68. Elemental analysis calcd. for $C_{25}H_{29}BF_4N_3Rh$: C 53.50, H 5.21, N 7.49. Found: C 53.41, H 5.18, N 7.45. **HRMS** calculated for $[M]^+$ $C_{25}H_{29}N_3Rh$: 474.1411, found: 474.1414.

Chloro[((1-(thienyl)-3-(3-(trimethoxysilyl)propyl)-1*H*-imidazol-2-ylidene))(η^4-1,5-cyclooctadiene)rhodium(I)], Rh5: 0.65 equivalents of Ag_2O (33 mg; 0.14 mmol, 0.65 equiv) were added at room temperature to a solution of **L3** (100 mg; 0.23 mmol, 1.0 equiv) in 2 mL CH_2Cl_2, along with molecular sieves and the mixture was stirred at room temperature under exclusion of light for 12 h. It was then filtered through celite and cooled to -34 °C for 20 min. A prechilled solution of 0.5 equivalents of $[Rh(COD)Cl]_2$ (56.7 mg, 0.12 mmol, 0.5 equiv) dissolved in 2 mL CH_2Cl_2 was then added and the resultant reaction suspension was stirred at room temperature for a further 2 h. The reaction mixture was then filtered through celite, the filtrate was evaporated under reduced pressure, the residue was washed twice with pentane and the remaining solid was dried under vacuum to yield **Rh5** as yellow-coloured solid. Yield: 71% (90 mg). **^{1}H NMR** (400 MHz, CD_2Cl_2) δ 7.44-7.43 (dd, J_1 = 3.7, J_2 =1.5 Hz, 1H), 7.24-7.22 (m, 2H), 7.05-7.02 (m, 2H), 5.11-5.06 (td, J_1 = 7.9, J_2 =4.1 Hz, 1H), 4.98-4.93 (m, 1H), 4.71-4.64 (ddd, J_1 = 13.3, J_2 =9.4, J_3 = 6.0 Hz, 1H), 4.58-4.51 (ddd, J_1 =13.3, J_2 =9.4, J_3 =6.1 Hz, 1H), 3.58 (s, 9H), 3.29-3.24 (m, 1H), 2.93-2.87 (tt, J_1 = 7.2, J_2 =6.5, 1H), 2.40-2.30 (m, 4H), 2.03-1.85 (m, 6H), 0.81-0.76 (m, 2H). **^{13}C NMR** (101 MHz, CD_2Cl_2) δ 184.7 (d, $J_{Rh\text{-}C}$ = 51.4 Hz), 142.2, 125.1, 121.9, 121.8, 121.2, 119.7, 98.6, 98.5, 97.9, 97.8, 78.7-78.5, 68.6-68.3, 50.5-50.4, 33.1, 32.1, 30.9, 29.0, 28.4, 24.0, 6.6. Elemental analysis calcd. for $C_{21}H_{32}ClN_2O_3RhSSi$: C 45.12, H 5.77, N 5.01. Found: C 44.99, H 5.68, N 4.95 **HRMS** calculated for $[M]^+$ $C_{21}H_{32}N_2O_3RhSSi$:523.0952, found: 523.0966.

[(1-{Pyrid-2-yl}-3-{3-{trimethoxysilyl}propyl}-imidazol-2-ylidene) (η^4-1,5-cyclooctadiene)rhodium(I) tetrakis[3,5-bis(trifluoromethyl)phenyl]borate], Rh6: The synthetic procedure was the same as outlined for **Rh3**, except for the use of sodium tetrakis[3,5-bis(trifluoromethyl)phenyl]borate (79.8 mg, 0.09 mmol, 1.0 equiv) instead of $AgBF_4$. The compound was obtained as orange-coloured foam. Yield: 72% (89.8 mg). **^{1}H NMR** (400 MHz, CD_2Cl_2) δ 8.06-8.01 (ddd, J_1 = 8.4, J_2 =7.6, J_3 =1.6 Hz, 1H), 7.82 (d, J = 5.7 Hz, 1H), 7.74-7.72 (m, 8H), 7.57 (s, 4H), 7.43-7.38 (m, 3H), 7.03 (d, J = 2.2 Hz, 1H), 5.04-5.02 (m, 2H), 4.93-4.91 (m, 2H), 3.72-3.68 (m, 2H), 3.55 (s, 9H), 2.49-2.41 (m, 4H), 2.23-2.17 (m, 4H), 1.96-1.91 (m, 2H), 0.71-0.67 (m, 2H). **^{13}C NMR** (101 MHz, CD_2Cl_2) δ 174.4 (d, $J_{Rh\text{-}C}$ = 54.5 Hz), 162.5 (q, $J_{C\text{-}B}$ = 50.5 Hz), 152.4, 147.9, 142.7, 134.8, 129.3 (qq, $^2J_{C\text{-}F}$ = 27.3, $^3J_{C\text{-}B}$ = 3.0 Hz), 125.9 (q, $^1J_{C\text{-}F}$ = 273.7 Hz), 123.7, 123.4, 120.5, 117.6

(septet*, $^3J_{C\text{-}F}$ = 4.0 Hz), 115.6, 111.2, 98.4, 98.3, 78.1, 78.0, 51.3, 50.7, 50.6, 50.6, 32.5, 28.4, 25.0, 5.9. *septet unresolved. **^{19}F NMR** (376 MHz, CD_2Cl_2) δ -62.80 (24F). Elemental analysis calcd. for $C_{54}H_{45}BF_{24}N_3O_3RhSi$: C 46.94, H 3.28, N 3.04. Found: C 46.98, H 3.327, N 2.94. **HRMS** calculated for $[M]^+$ $C_{22}H_{33}N_3O_3RhSi$: 518.1341, found: 518.1344.

[((1-(Thienyl)-3-(3-(trimethoxysilyl)propyl)-1*H*-imidazol-2-ylidene))(η^4-1,5-cyclooctadiene)rhodium(I) tetrakis[3,5-bis(trifluoromethyl)phenyl]borate], Rh7: The synthetic procedure was the same as outlined for **Rh3**, except for the use of sodium tetrakis[3,5-bis(trifluoromethyl)phenyl]borate instead of $AgBF_4$. The compound was obtained as yellow-coloured solids. Yield: 78.2 % (97 mg) ^{1}H NMR (400 MHz, CD_2Cl_2) δ 7.75-7.72 (m, 16H), 7.58-7.57 (m, 8H), 7.41 (d, *J* = 2.1 Hz, 2H), 7.34-7.32 (dd, J_1 = 5.2, J_2 = 1.7 Hz, 2H), 7.14-7.10 (m, 4H), 7.06 (d, *J* = 2.0 Hz, 2H), 4.95 (br, 4H), 4.73-4.70 (m, 4H), 3.50 (s, 18H), 3.38-3.37 (m, 4H), 2.48-2.30 (m, 8H), 2.17-2.14 (m, 4H), 1.97-1.84 (m, 8H), 0.95-0.92 (m, 4H). **^{13}C NMR** (101 MHz, CD_2Cl_2) δ 176.4 (d, $J_{Rh\text{-}C}$ = 51.51 Hz), 162.5 (q, $J_{C\text{-}B}$ = 50.5 Hz), 140.6, 134.78, 129.4-128.3 (m), 126.8, 125.9 (q, $^1J_{C\text{-}F}$ = 273.7 Hz), 123.0, 122.5, 121.8, 120.5, 118.6, 117.5 (septet*, $^3J_{C\text{-}F}$ = 4.0 Hz), 100.6, 100.5, 70.2, 70.1, 32.1, 27.8, 23.8, 6.3. *septet unresolved. **^{19}F NMR** (376 MHz, CD_2Cl_2) δ -62.84. Elemental analysis calcd. for $C_{53}H_{44}BF_{24}N_2O_3RhSSi$: C 45.90, H 3.20, N 2.02. Found: C 45.99, H 3.28, N 2.08. **HRMS** calculated for $[M]^+$ $C_{21}H_{32}N_2O_3RhSSi$:523.0952, found: 523.0954. Crystals suitable for single X-ray analysis were obtained by layering n-pentane over saturated solution of **Rh7** in dichloromethane at -34 °C

[Chloro(Cp*)((1-(pyrid-2-yl)-3-mesityl)-imidazol-2-ylidene))rhodium(III) chloride], Rh9: The synthetic procedure was the same as for the synthesis of **Rh5**, except for the use of 0.5 equivalents of $[Cp^*RhCl_2]_2$. Yield: 85 % (68 mg). ^{1}H NMR (400 MHz, CD_2Cl_2) δ 9.75 (d, $J_{Rh\text{-}C}$ = 2.3 Hz, 1H), 9.31-9.29 (dt, J_1 = 8.4, J_2 = 1.0 Hz, 1H), 8.54 (dd, J_1 = 5.7, J_2=1.6 Hz, 1H), 8.26-8.22 (ddd, J_1 = 8.4, J_2 = 7.5, J_3 = 1.6 Hz, 1H), 7.56-7.52 (ddd, J_1 = 7.5, J_2 =C5.7, J_3 = 1.2 Hz, 1H), 7.14 (d, *J* = 2.2 Hz, 1H), 7.11 (ddd, J_1 = 9.1, J_2 = 1.4, J_3 = 0.7 Hz, 1H), 2.39 (s, 3H), 2.26 (s, 3H), 2.19 (s, 3H), 1.44 (s, 15H). **^{13}C NMR** (101 MHz, CD_2Cl_2) δ 178.0 (d, *J* = 54.6 Hz), 151.4, 149.8, 142.0, 140.8, 137.0, 135.3, 134.1, 130.1, 129.4, 127.3, 124.4, 119.3, 114.5, 99.2, 99.1, 20.9, 18.9, 18.4, 9.2, Elemental analysis

calcd. for $C_{27}H_{32}Cl_2N_3Rh$. 0.5 CH_2Cl_2: C 53.72, H 5.41, N 6.83. Found: C 54.12, H 5.441, N 6.83. **HRMS** calculated for $[M]^+$ $C_{27}H_{32}Cl_1N_3Rh$: 536.1334, found: 536.1337. Crystal suitable for the single X-ray analysis were obtained from a saturated solution of **Rh9** in CH_2Cl_2 at -34 °C.

[Chloro(Cp*)((1-{pyrid-2-yl}-3-{3-{trimethoxysilyl}propyl}-)-imidazol-2-ylidene)rhodium(III) tetrafluoroborate], Rh10: To a stirred solution of **L1** (100 mg; 0.23 mmol, 1.0 equiv) in 2 mL CH_2Cl_2, silver oxide (35.0 mg; 0.15 mmol, 0.65 equiv) was added at room temperature to a solution, along with molecular sieves and the mixture was stirred at room temperature under exclusion of light for 12 h. It was then filtered through celite and silver tetrafluoroborate (44.8 mg, 0.23 mmol, 1.0 equiv) was added and stirred for 1 h in darkness. The reaction mixture was then filtered through celite and $[Cp^*RhCl_2]_2$ (74 mg, 0.12 mmol, 0.5 equiv) was added and the reaction mass was stirred for 12 h. The reaction mixture was then filtered through celite, the filtrate was evaporated under reduced pressure, the residue was washed twice with diethyl ether and the remaining solid was dried under vacuum to yield **Rh10** as orange-coloured solid. Yield: 35 % (51 mg). **^{1}H NMR** (400 MHz, CD_2Cl_2) δ 8.65 (d, J = 2.3 Hz, 1H), 8.55-8.54 (m, 1H), 8.45-8.43 (m, 1H), 8.21 (ddd, J_1 = 8.3, J_2 = 7.5, J_3 = 1.6 Hz, 1H), 7.53-7.49 (ddd, J_1 =7.5, J_2 = 5.7, J_3 = 1.2 Hz, 1H), 7.45 (d, J = 2.3 Hz, 1H), 4.37-4.20 (m, 2H), 3.54 (s, 9H), 2.06-1.98 (m, 2H), 1.74 (s, 15H), 0.74-0.57 (m, 2H). **^{19}F NMR** (376 MHz, CD_2Cl_2) δ -152.3 **^{13}C NMR** (101 MHz, CD_2Cl_2) δ 179.7 (d, J = 51.51 Hz), 151.5, 150.0, 142.3, 124.5, 123.9, 119.2, 114.0, 99.2, 65.7, 50.5, 24.3, 9.6, 6.3. **HRMS** calculated for $[M]^+$ $C_{24}H_{36}ClN_3O_3RhSi$: 580.1264, found: 580.1270.

[Chloro(Cp*)((1-(pyrid-2-yl)-3-mesityl)-imidazol-2-ylidene))rhodium(III) tetrafluoroborate], Rh11: The synthetic procedure was the same as for the synthesis of **Rh10.** Yield: 78 % (81 mg). **^{1}H NMR** (400 MHz, CD_2Cl_2) δ 9.29 (d, J = 2.3 Hz, 1H), 8.92 (d, J = 8.4 Hz, 1H), 8.54 (dd, J_1 = 5.7, J_2 =1.5 Hz, 1H), 8.22-8.18 (ddd, J_1 = 8.4, J_2 =7.5, J_3 =1.6 Hz, 1H), 7.57-7.53 (ddd, J_1 = 7.5, J_2 = 5.7, J_3 = 1.2 Hz, 1H), 7.13 (d, J = 2.2 Hz, 1H), 7.11 (ddd, J_1 = 10.6, J_2 = 1.4, J_3 = 0.7 Hz, 1H), 2.38 (s, 3H), 2.24 (s, 3H), 2.17 (s, 3H), 1.43 (s, 15H). **^{19}F NMR** (376 MHz, CD_2Cl_2) δ -150.1. **^{13}C NMR** (101 MHz, CD_2Cl_2) δ 177.7 (d, J_{Rh-C} = 54.5 Hz), 151.5, 149.7, 142.1, 140.8, 137.0, 135.3, 134.2, 130.1, 129.3,

127.2, 124.3, 119.7, 114.8, 99.2, 99.1, 65.7, 20.9, 18.9, 18.3, 9.1. Elemental analysis calcd. for $C_{24}H_{36}BClF_4N_3O_3RhSi$: C 43.17, H 5.43, N 6.29. Found: C 42.73, H 5.47, N 6.11. **HRMS** calculated for $[M]^+$ $C_{27}H_{32}Cl_1N_3Rh$: 536.1334, found: 536.1328.

[Chloro(Cp*)((1-(thienyl)-3-(3-(trimethoxysilyl)propyl)-1*H*-imidazol-2-ylidene)rhodium(III) chloride], Rh12: The synthetic procedure was the same as for the synthesis of **Rh5**, except for the use of 0.5 equivalents of $[Cp^*RhCl_2]_2$. Yield: 61 % (42.7 mg). **^{1}H NMR** (400 MHz, CD_2Cl_2) δ 7.69 (d, J = 3.7 Hz, 1H), 7.27 (dd, J_1 = 5.6, J_2 =1.4 Hz, 1H), 7.20 (d, J = 2.1 Hz, 1H), 7.18 (d, J = 2.0 Hz, 1H), 6.96 (dd, J_1 = 5.6, J_2 =3.8 Hz, 1H), 4.80 (br, 1H), 4.02 (br, 1H), 3.58 (s, 9H), 2.04-1.97 (br, 2H), 1.41 (s, 15H), 0.76 (br, 2H). **^{13}C NMR** (101 MHz, CD_2Cl_2) δ 174.7 (d, $J_{Rh\text{-}C}$ = 58.58 Hz), 140.4, 128.8, 128.6, 125.0, 124.2, 121.7, 96.4, 50.5, 25.3, 9.0, 6.2. **HRMS** calculated for [M-H] $C_{23}H_{35}N_2O_3RhSSi$: 549.1109, found: 549.1109.

[Chloro(Cp*)((1-(thienyl)-3-(3-(trimethoxysilyl)propyl)-1*H*-imidazol-2-ylidene)rhodium(III) tetrafluoroborate], Rh13: **Rh12** (50 mg, 0.08 mmol, 1.0 equiv) was dissolved in 3 mL of CH_2Cl_2 and the solution was chilled to -34 °C. Next, a suspension of $AgBF_4$ (15.6 mg, 0.08 mmol, 1.0 equiv) was slowly added at -34 °C, the mixture was stirred for 60 min, then filtered through celite and concentrated under reduced pressure. The residue was washed with pentane and dried under vacuum to obtain an orange-coloured foam. Yield: 66% (35.4 mg). **^{1}H NMR** (400 MHz, CD_2Cl_2) δ 7.67 (d, J = 2.1 Hz, 1H), 7.45-7.41 (m, 2H), 7.30-7.28 (dd, J_1 = 3.8, J_2 =1.1 Hz, 1H), 7.20-7.18 (dd, J_1 = 5.6, J_2 =1.1 Hz, 1H), 4.16-4.01 (m, 2H), 3.57 (s, 9H), 2.05-1.95 (m, 2H), 1.57 (s, 15H), 0.74-0.66 (m, 2H). **^{19}F NMR** (376 MHz, CD_2Cl_2) δ -152.7. **^{13}C NMR** (101 MHz, $CDCl_3$) δ 172.8 (d, $J_{Rh\text{-}C}$ = 56.6 Hz), 124.2, 123.1, 120.3, 100.5, 100.4, 96.3, 96.2, 50.8, 25.5, 9.5, 6.4. Compound decomposes during measurement. **HRMS** calculated for [M-H] $C_{23}H_{35}N_2O_3RhSSi$: 549.1109, found: 549.1110. Crystal suitable for the single X-ray analysis were obtained by layering diethyl ether over a saturated solution of **Rh13** in tetrahydrofuran at -34 °C.

Typical procedure for the immobilization of the catalyst: SBA-15 selectively protected outside the mesopores[24] was added to a solution of the rhodium catalyst in 1,2-dichlorobenzene. For the removal of nitrogen in the pores, vacuum was applied. The

suspension was stirred for three hours under vacuum at room temperature. Then, the suspension was filtered and the resulting silica containing the immobilized catalyst was washed with 1,2-dichlorobenzene and n-pentane, dried under vacuum at room temperature for three hours and stored under inert atmosphere at -35 °C.

Typical procedure for the Rh-catalysed hydrosilylation of alkynes: Inside a vial, the catalyst (1 mol%) and the 1-alkyne (1.0 equiv) were dissolved in 0.5 mL of $CDCl_3$. The reaction mixture was stirred at room temperature for 5 min and silane (1.5 equiv.) was added dropwise. The reaction mixture was stirred at the prescribed temperature for the time indicated during which both conversion and the ratio of isomers was monitored by ^{1}H NMR spectroscopy using *t*-butylbenzene as internal standard. The reaction products were characterized based on the $^3J_{H–H}$ coupling constants of the vinylic protons in the ^{1}H NMR spectra and subsequent comparison to literature values. Values for *J* ranged from 17 to 19 Hz for β-(*E*), 13 to 16 Hz for β-(*Z*), and 1 to 3 Hz for α-vinylsilanes.

Recyclability of the catalyst: A hydrosilylation reaction was performed employing 1-octyne and dimethylphenylsilane as the coupling partners. The reaction was run for 30 h; then, the reaction mixture was filtered, the solid catalyst was washed repeatedly with chloroform, dried under reduced pressure for 8 h and used for subsequent reactions.

Determination of rhodium leaching: A potential Rh leaching into the reaction mixture was analysed by ICP-OES. After reaction completion, the reaction mixture was filtered through celite, the solvent was evaporated under vacuum and the residue was redissolved in aqua regia and analysed by ICP-OES. Rh was measured at 249.077 nm, the background was measured from 248.950 nm - 248.995 nm and from 249.126 nm - 249.150 nm. Calibration was accomplished using Rh standards in 1M nitric acid containing 0.1 - 10 mg Rh/L. No rhodium was detected in the reaction solution.

X-ray absorption measurements. Samples were measured at the P65 beamline, Petra III, DESY.[26] [Measurements were carried out using a four-channel Silicon Drift Detector with a resolution of 135 eV. Energy selection was performed using the Si(311) Double Crystal Monochromator (DCM); higher harmonic rejection was obtained using the Pt-coated mirrors. The DCM resolving power was between $1\text{-}2\cdot10^4$, which translated into an

averaged experimental resolution of 1.7 eV at the Rh K-edge. Calibration was performed on the first inflection point of the Rh foil K-edge XANES spectrum (23220 eV). All measurements were conducted up to 1000 eV above the Rh K edge at room temperature with continuous scan mode parameters of 180 s/spectrum and 0.1 s per point. Samples were measured in the form of pellets. To avoid radiation damage, each spectrum was collected at different points of the sample. Analysis was performed with the aid of the Demeter software package.[27] Initial rebinning, background reduction and normalization of the experimental spectra were made in the Athena software. An EXAFS fitting was performed using the Artemis program and Multiple Scattering Theory.

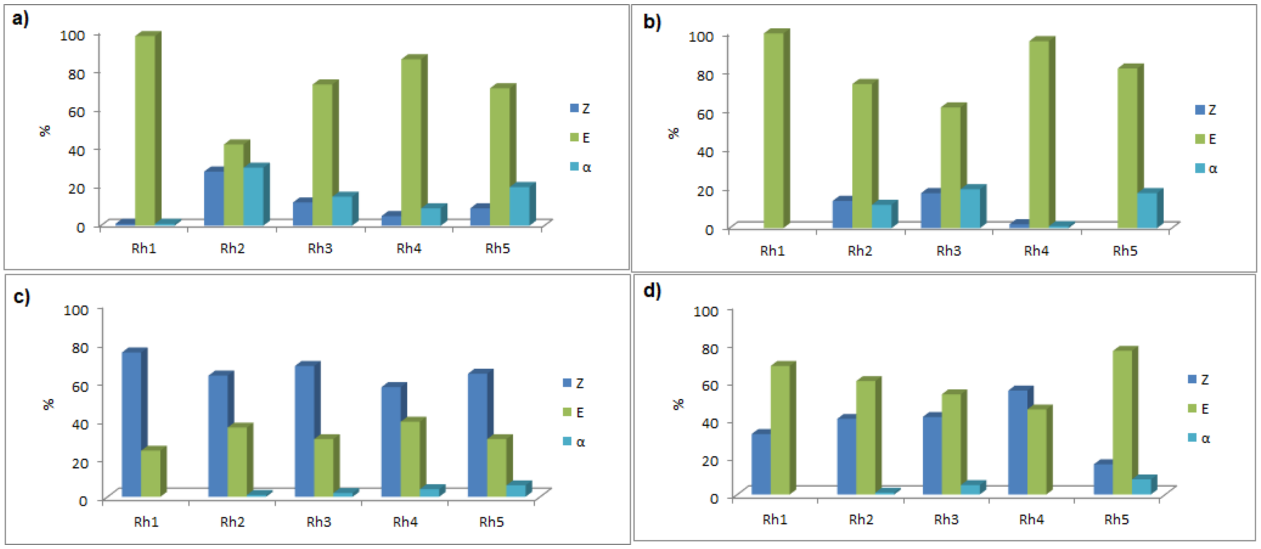

Figure S1. Isomer distribution in alkyne hydrosilylation catalyzed by the Rh(I) complexes **Rh1** – **Rh5**. a) Reaction profile of phenyl acetylene and $HSiMe_2Ph$, b) phenyl acetylene and $HSiEt_3$, c) 1-octyne and $HSiMe_2Ph$, and d) 1-octyne and $HSiEt_3$.

Table S1: Comparison of Rh catalysts immobilized inside different pore diameters.

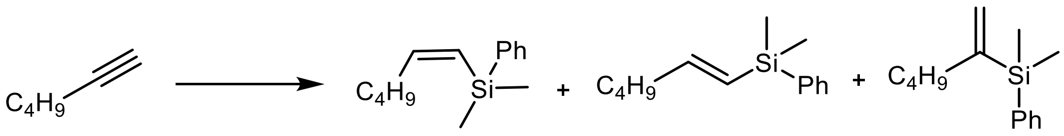

Entry	Substrate	Catalyst, time [a]	Conversion	β(*Z*)	β(*E*)	α
1	1-Hexyne	**Rh1**, 2h	100	45	55	-
2		**Rh1@SBA-15**$_{6.2\ nm}$, 40h	95	95	3	2
3		**Rh1@SBA-15**$_{5.0\ nm}$, 48h	94	95	2	3
4		**Rh3**, 2h	100	43	52	5
5		**Rh3@SBA-15**$_{6.2\ nm}$, 32h	95	93	7	-
6		**Rh3@SBA-15**$_{5.0\ nm}$, 48h	90	92	6	2

[a] Unless noted otherwise, all the reactions were performed at 60 °C employing 1.0 equiv. of alkyne, 1.5 equiv. of dimethylphenylsilane, 1 mol% of Rh catalyst, 0.5 mL of $CDCl_3$.

Table S2: Reactivity profile of **Rh10** in the room temperature hydrosilylation.

**Entry[a]**	**Alkyne**	**Time [h]**	**Conversion [%]**	**β(*Z*)**	**β(*E*)**	**α**
1	1-Octyne	2	3	98	2	-
2	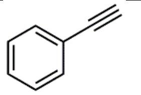	1	9	98	-	2
3	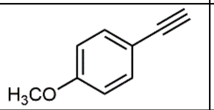	2	23	100	-	-
4	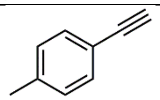	2	17	100	-	-
5	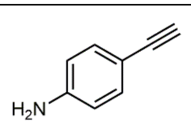	2	34	100	-	-

[a] Unless noted otherwise, all reactions were performed at 298K employing 1.0 equiv. of alkyne, 1.5 equiv. of dimethylphenylsilane, 1 mol% of Rh catalyst, 0.5 mL of $CDCl_3$.

Kinetic Studies

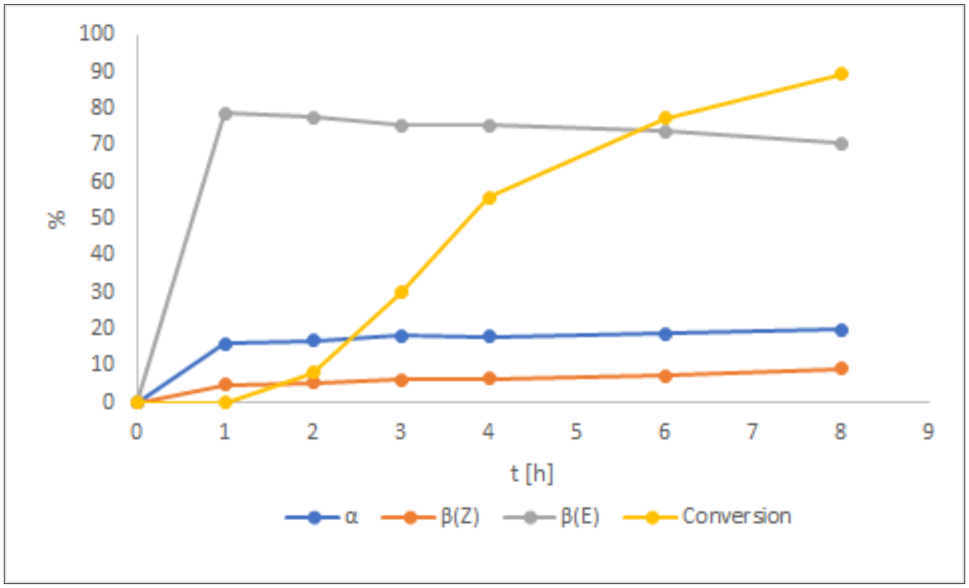

Figure S2: Time-dependent reaction profile of the reaction of phenylacetylene with $HSiMe_2Ph$ employing **Rh5**.

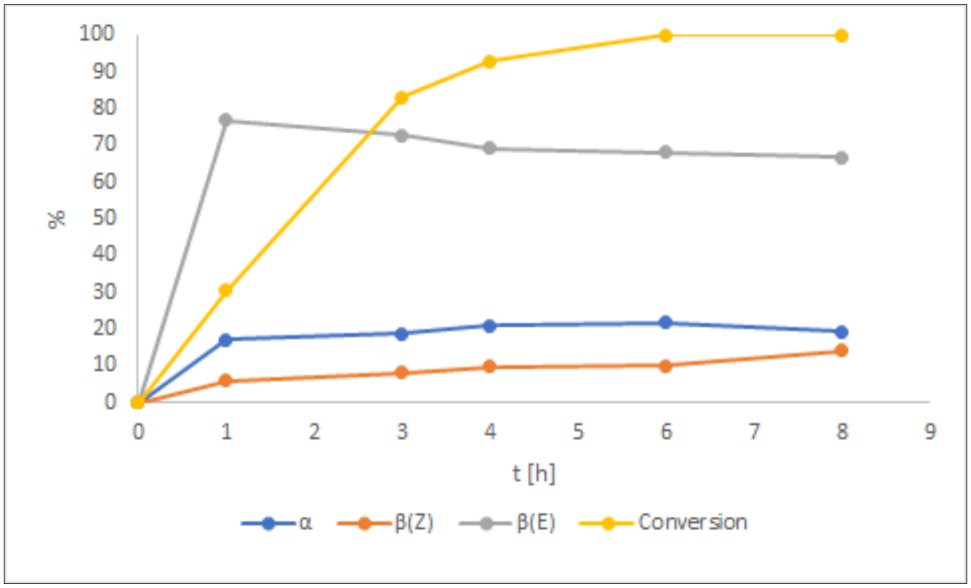

Figure S3: Time-dependent reaction profile of the reaction of 4-ethynyltoluene with $HSiMe_2Ph$ employing **Rh5**.

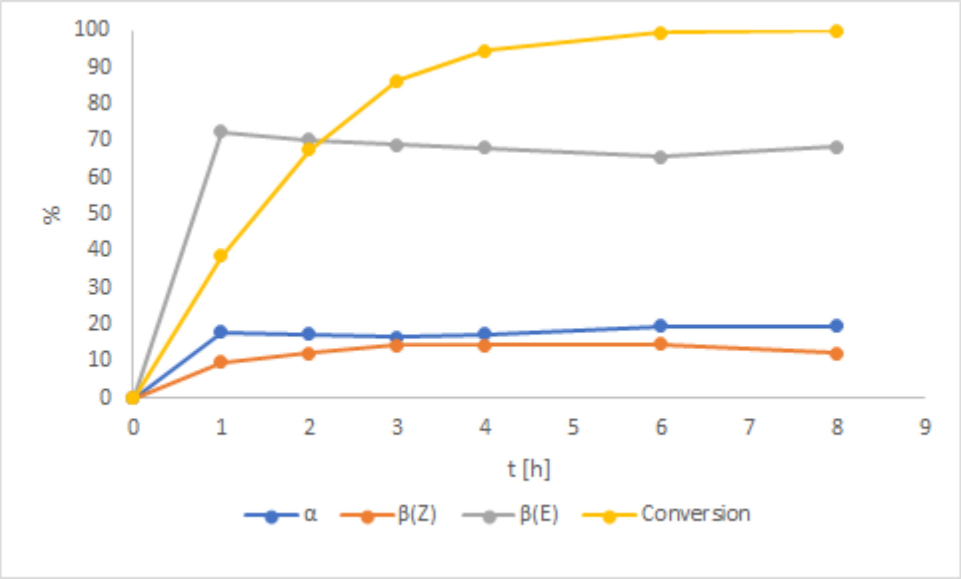

Figure S4: Time-dependent reaction profile of the reaction of 4-ethynylanisole with $HSiMe_2Ph$ employing **Rh5**.

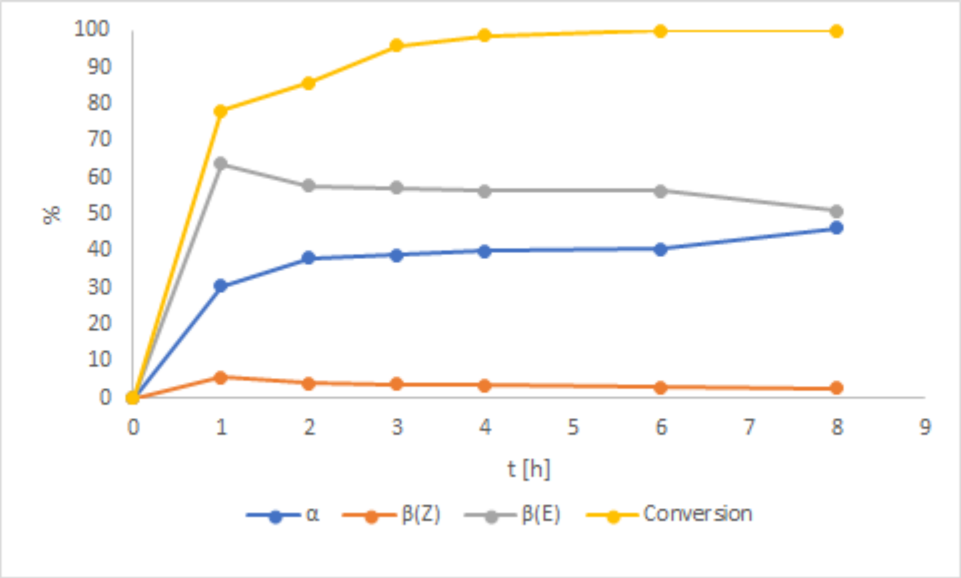

Figure S5: Time-dependent reaction profile of the reaction of 4-ethynylaniline with $HSiMe_2Ph$ employing **Rh5**.

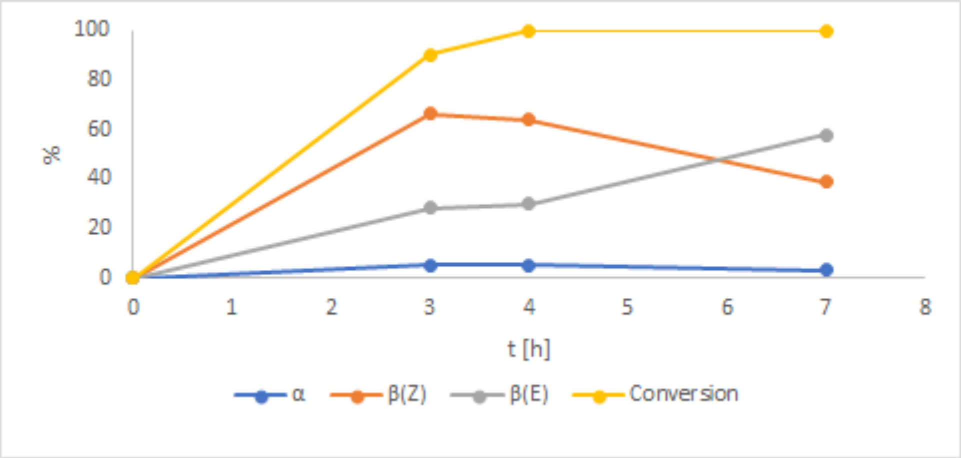

Figure S6: Time-dependent reaction profile of the reaction of 1-octyne with $HSiMe_2Ph$ employing **Rh5**.

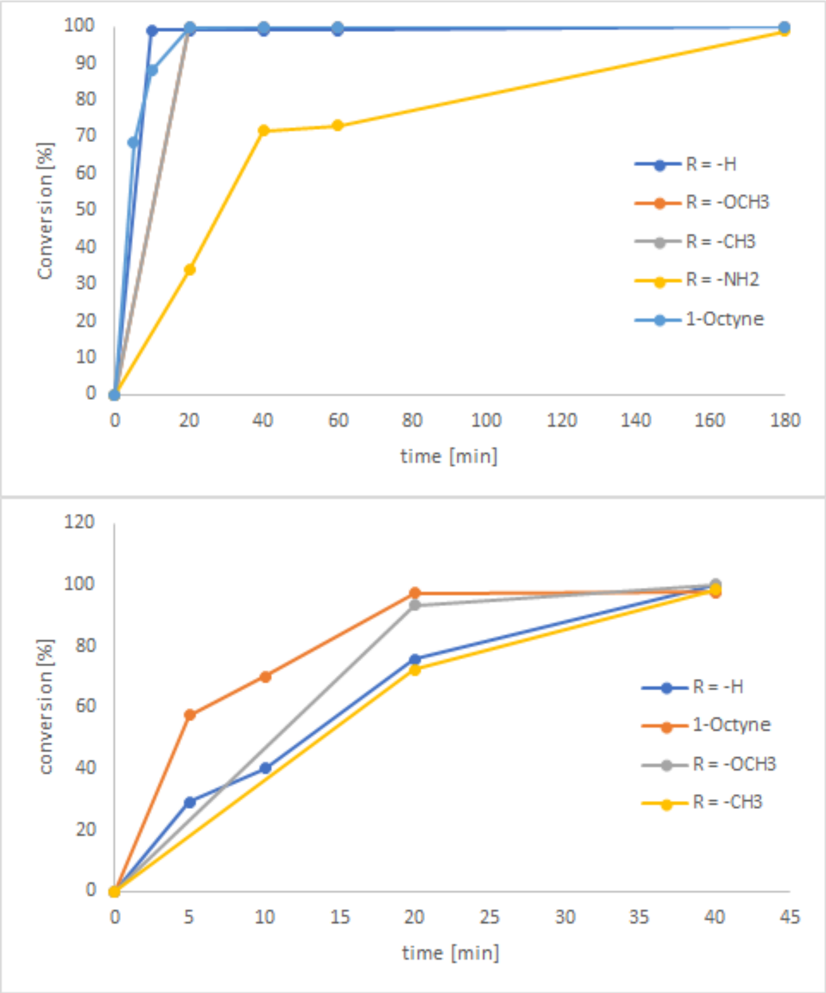

Figure S7. Reaction profiles of conversion *vs* time for the hydrosilylation of alkynes with $HSiMe_2Ph$, catalyzed by **Rh12** (top) and **Rh13** (bottom) in $CDCl_3$ at 298K.

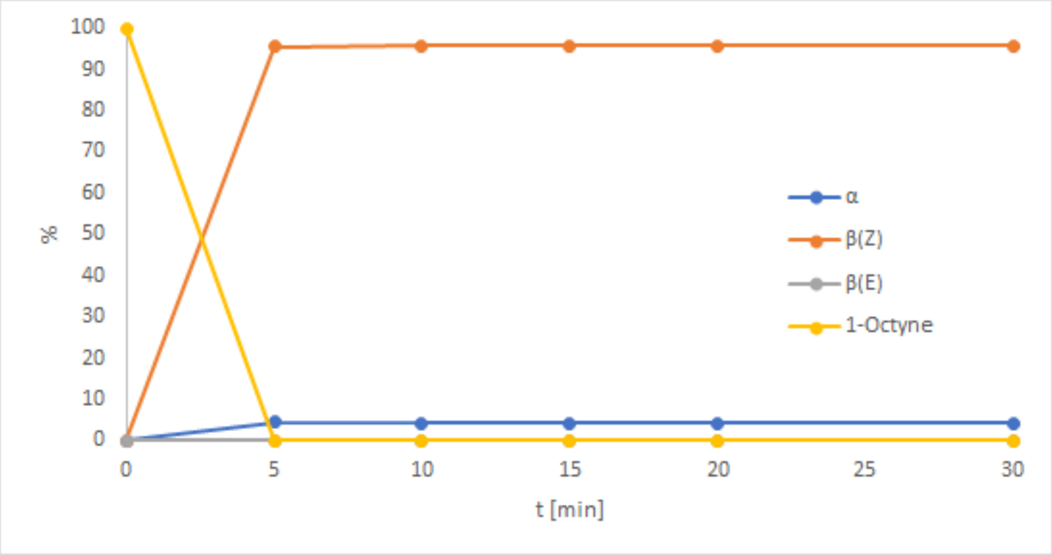

Figure S8: Time-dependent reaction profile of the reaction of 1-octyne with $HSiEt_3$ employing **Rh9**.

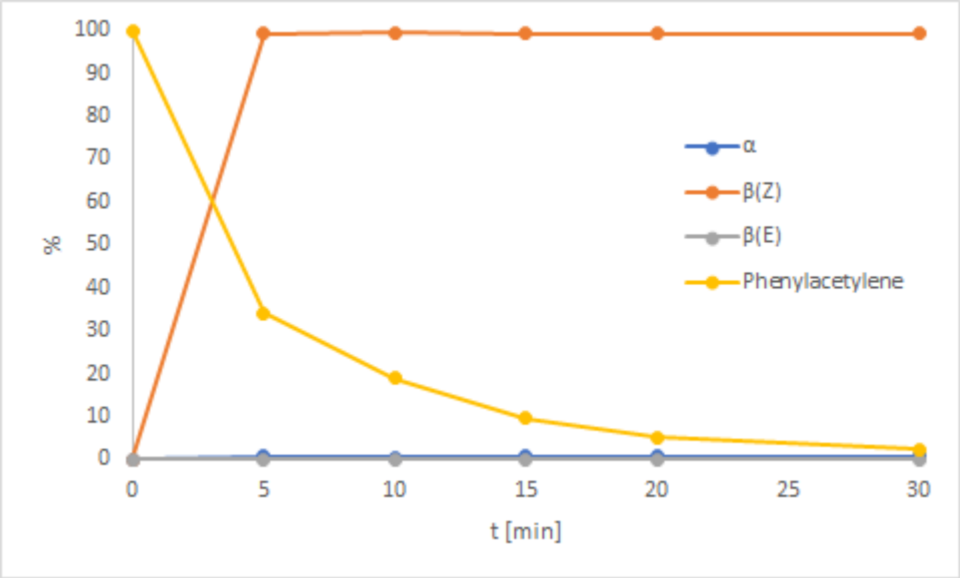

Figure S9: Time-dependent reaction profile of the reaction of phenylacetylene with $HSiEt_3$ employing **Rh9**.

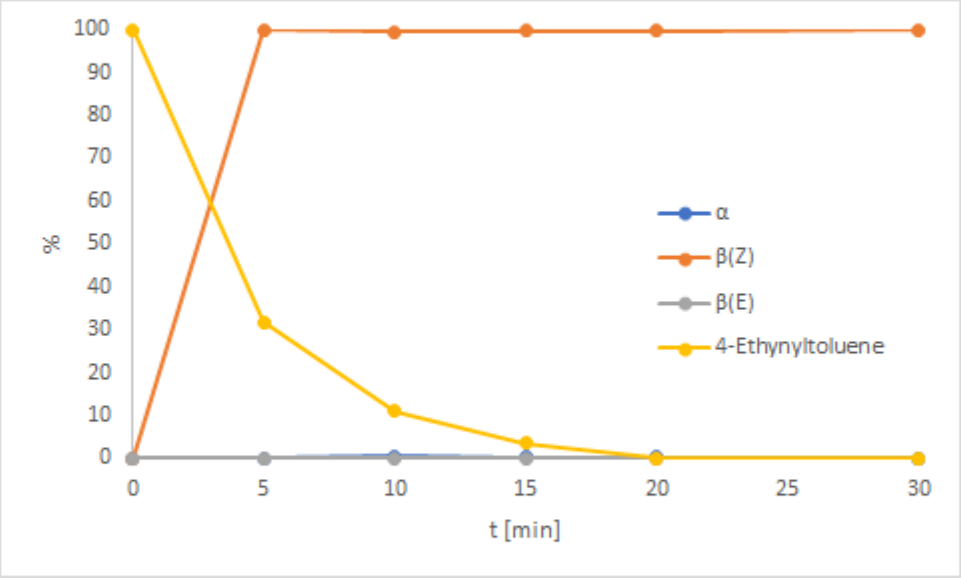

Figure S10: Time-dependent reaction profile of the reaction of 4-ethynyltoluene with $HSiEt_3$ employing **Rh9**.

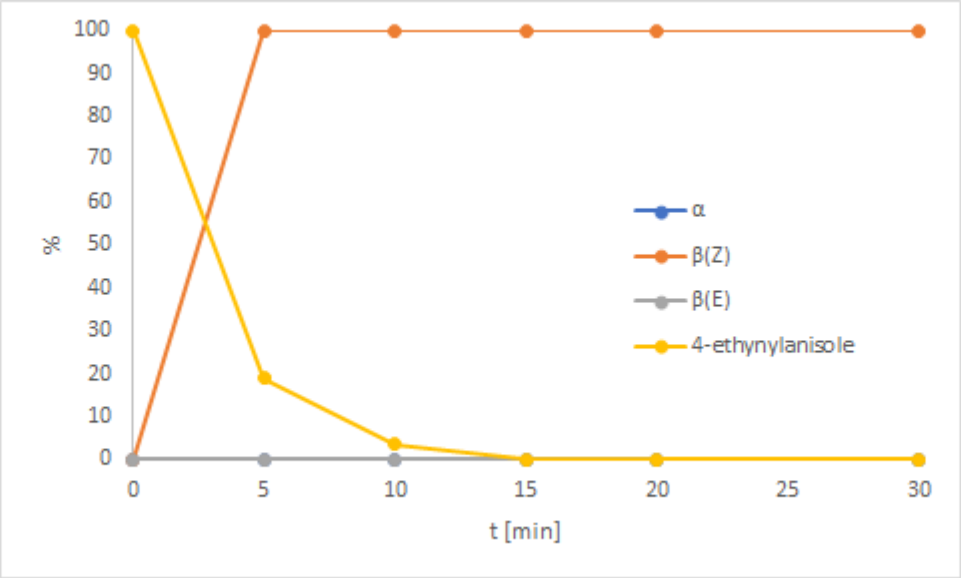

Figure S11: Time-dependent reaction profile of the reaction of 4-ethynylanisole with $HSiEt_3$ employing **Rh9**.

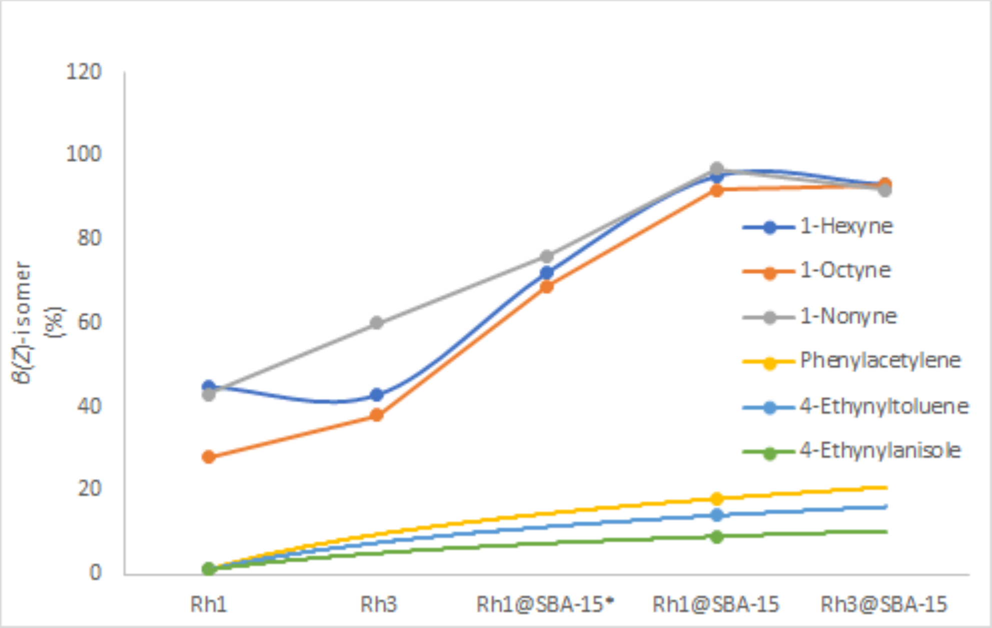

Figure S12. Comparative ratio of β(*Z*)-isomer for hydrosilylation of aliphatic alkynes with homogenous and heterogenous Rh(I)-NHC complexes.

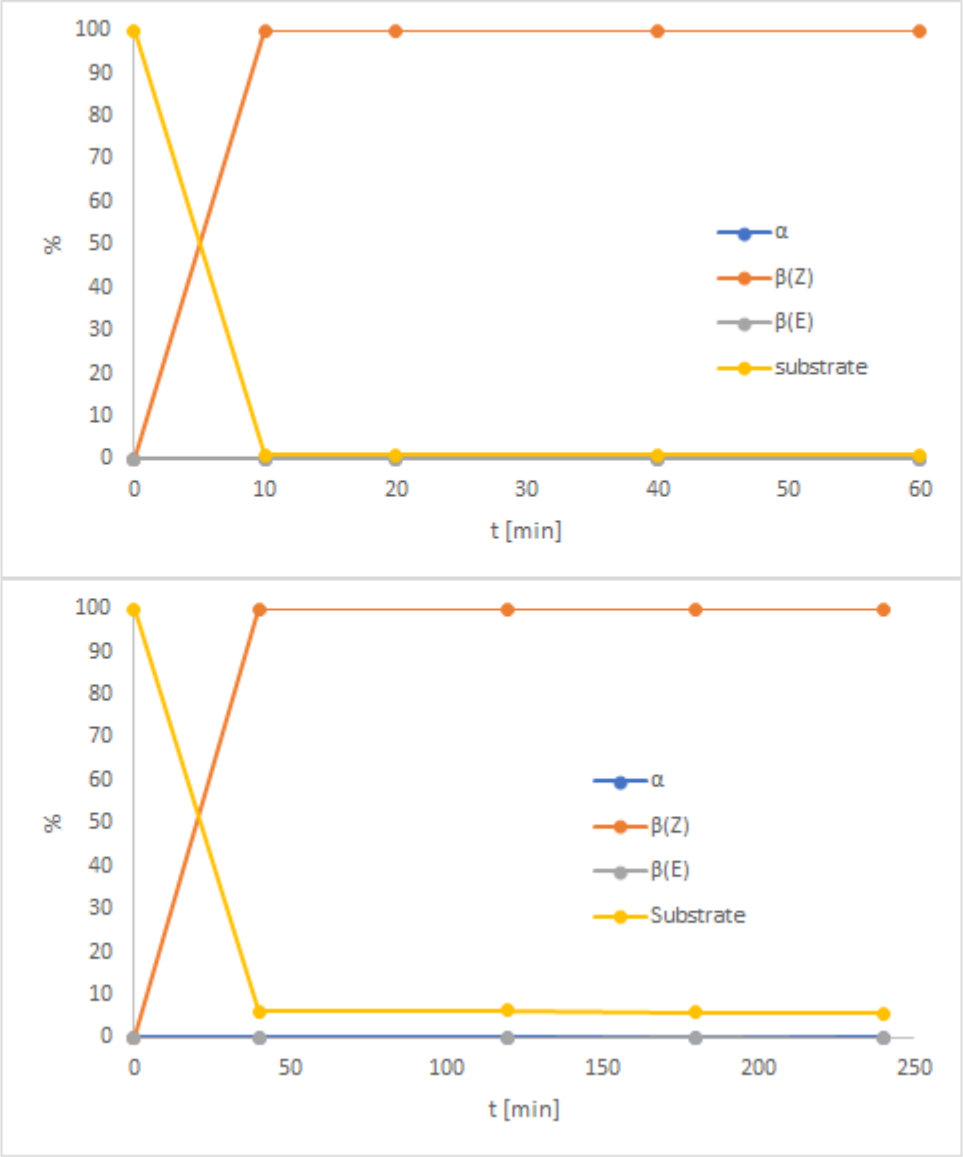

Figure S13. Time dependent reaction profiles of the reaction of phenylacetylene and $HSiMe_2Ph$ employing catalysts **Rh12** (top) and **Rh12@SBA-15$_{6.2nm}$** (bottom).

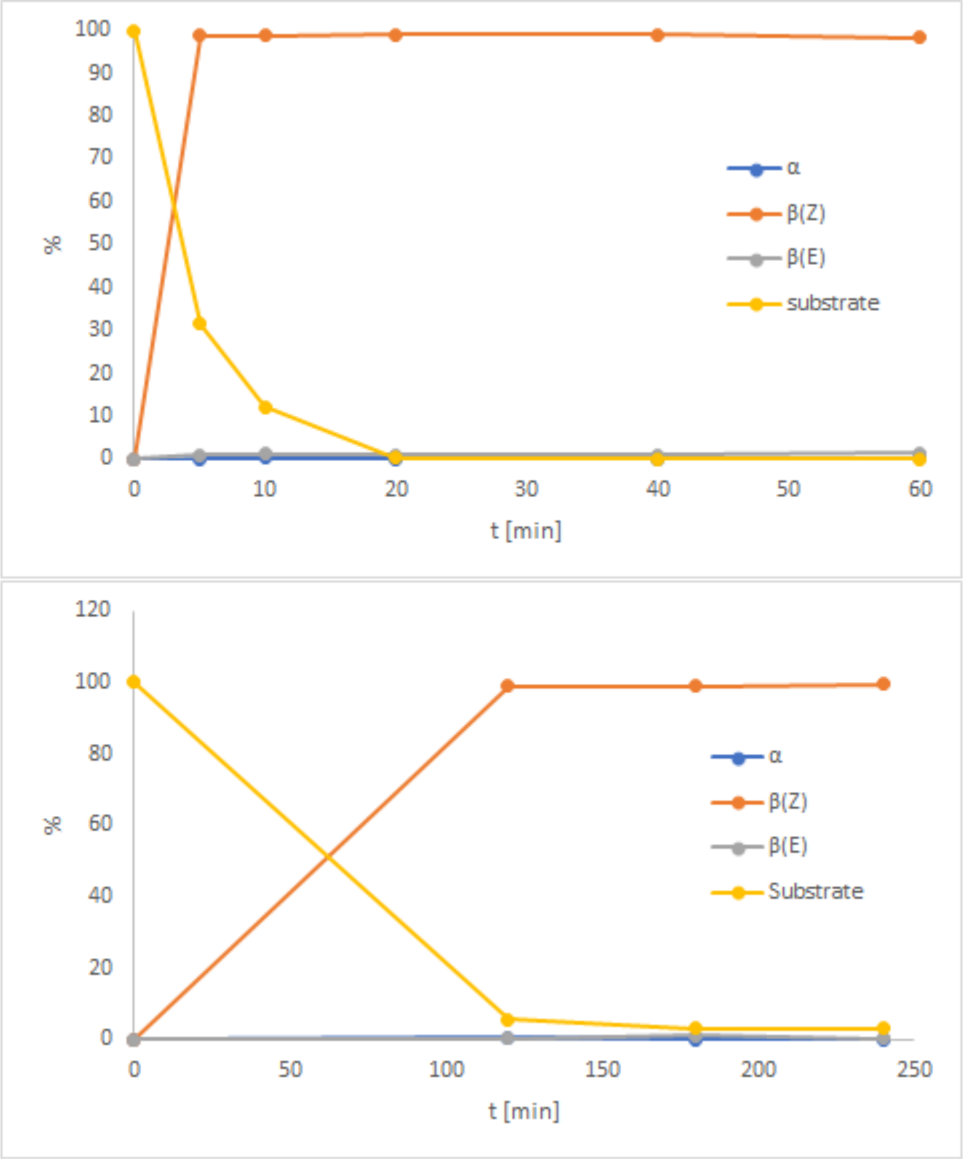

Figure S14. Time dependent reaction profiles of the reaction of 1-octyne and $HSiMe_2Ph$ employing catalysts **Rh12** (top) and **Rh12@SBA-15$_{6.2nm}$** (bottom).

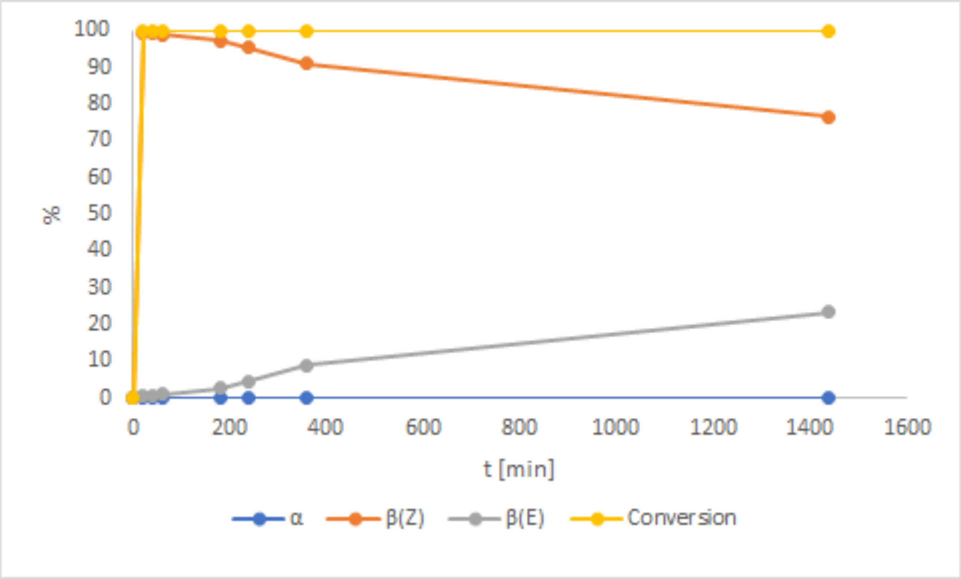

Figure S15: Time-dependent reaction profile of the reaction of 4-ethynyltoluene with $HSiMe_2Ph$ employing **Rh12**.

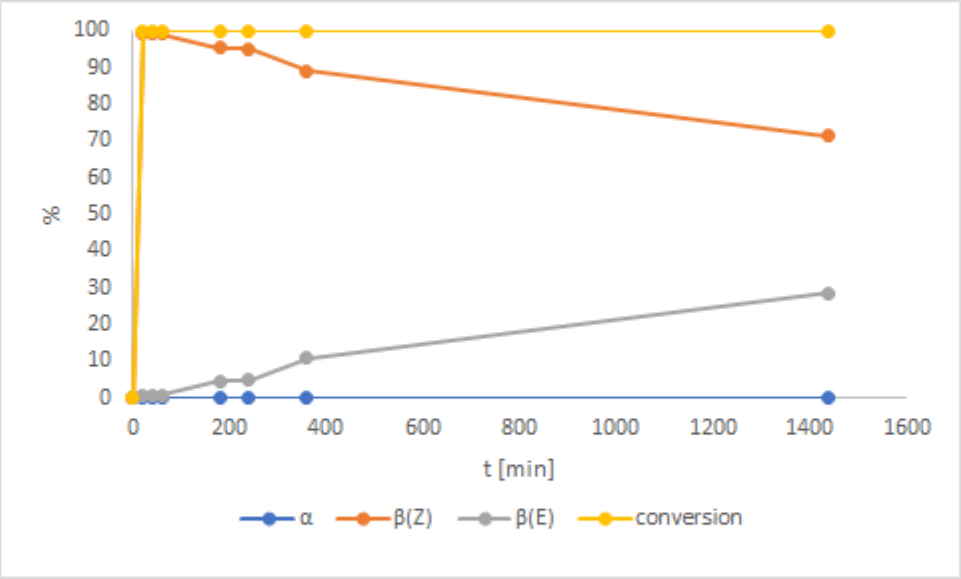

Figure S16: Time-dependent reaction profile of the reaction of 4-ehynylanisole with $HSiMe_2Ph$ employing **Rh12**.

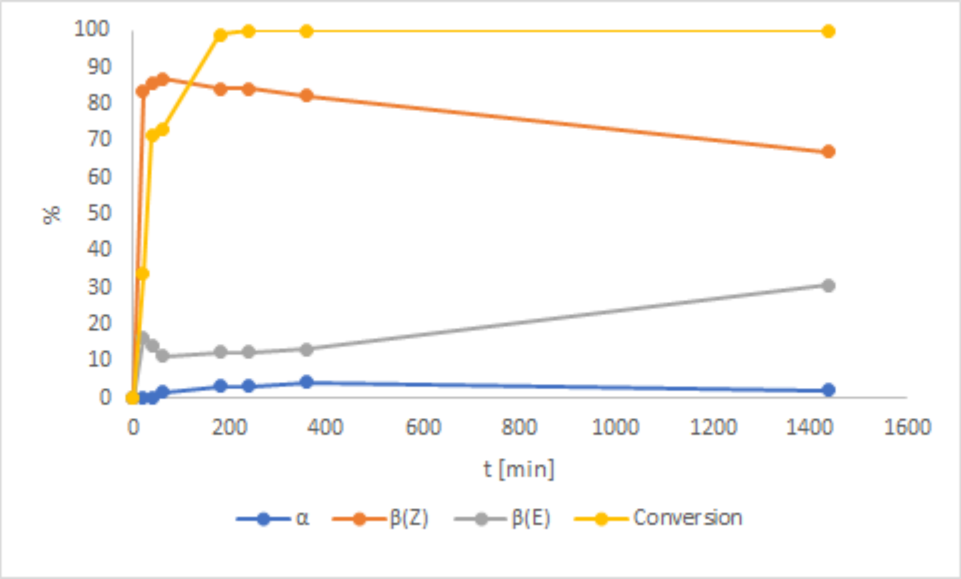

Figure S17: Time-dependent reaction profile of the reaction of 4-ethynylaniline with $HSiMe_2Ph$ employing **Rh12**.

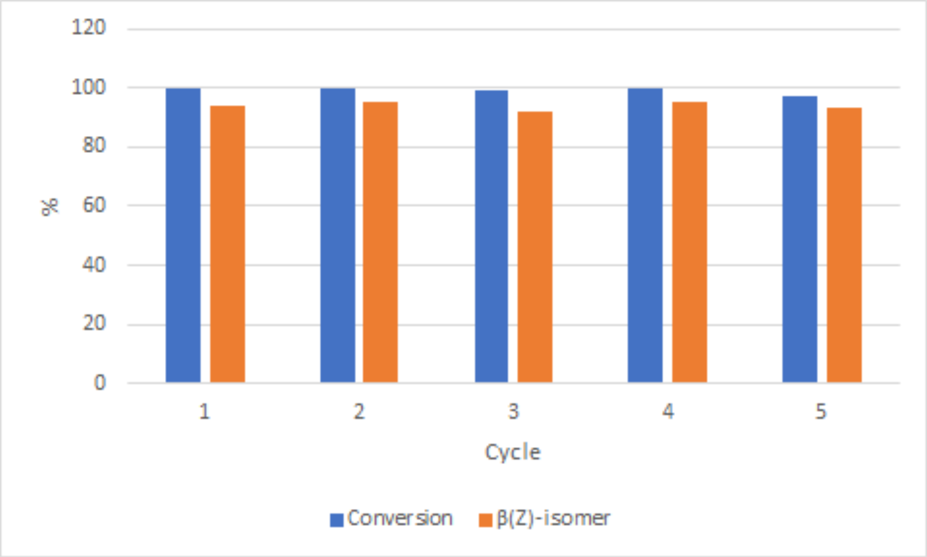

Figure S18: Recyclability studies for **Rh3@SBA-15** using 1-octyne and $HSiMe_2Ph$.

NMR Spectra

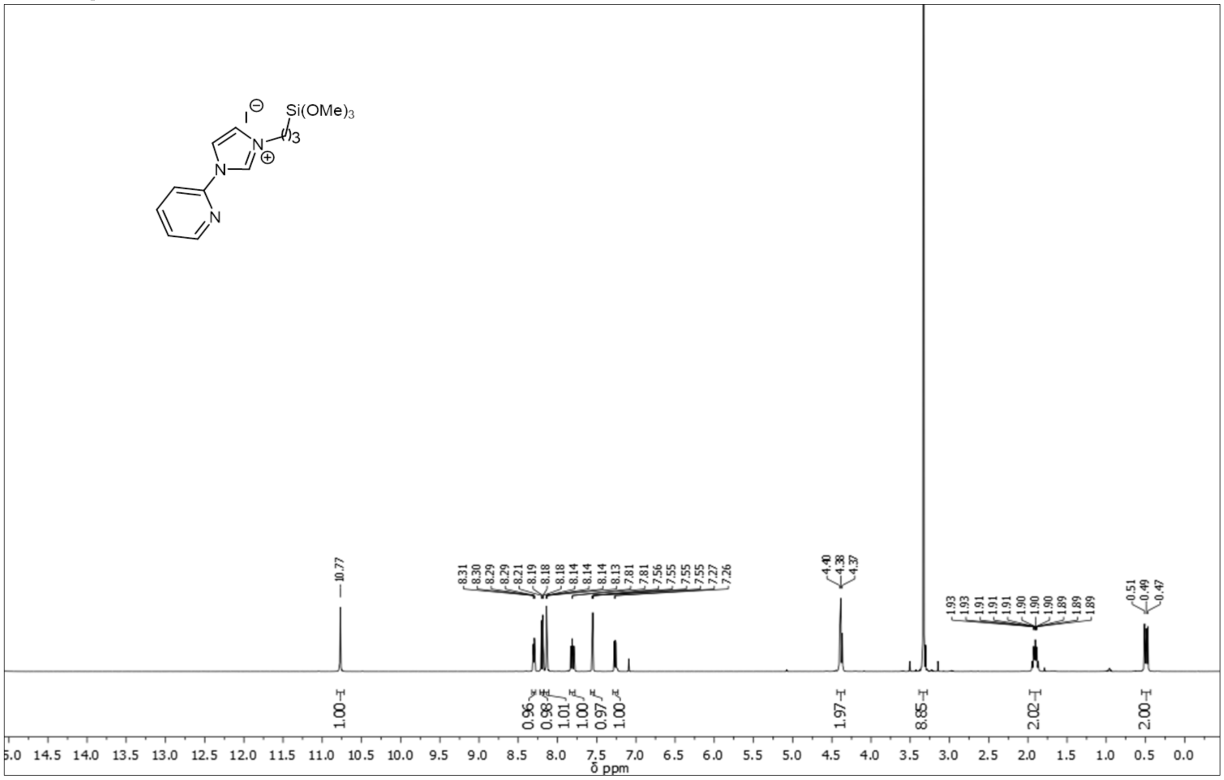

Figure S19. ^{1}H NMR spectrum of **L1** in $CDCl_3$.

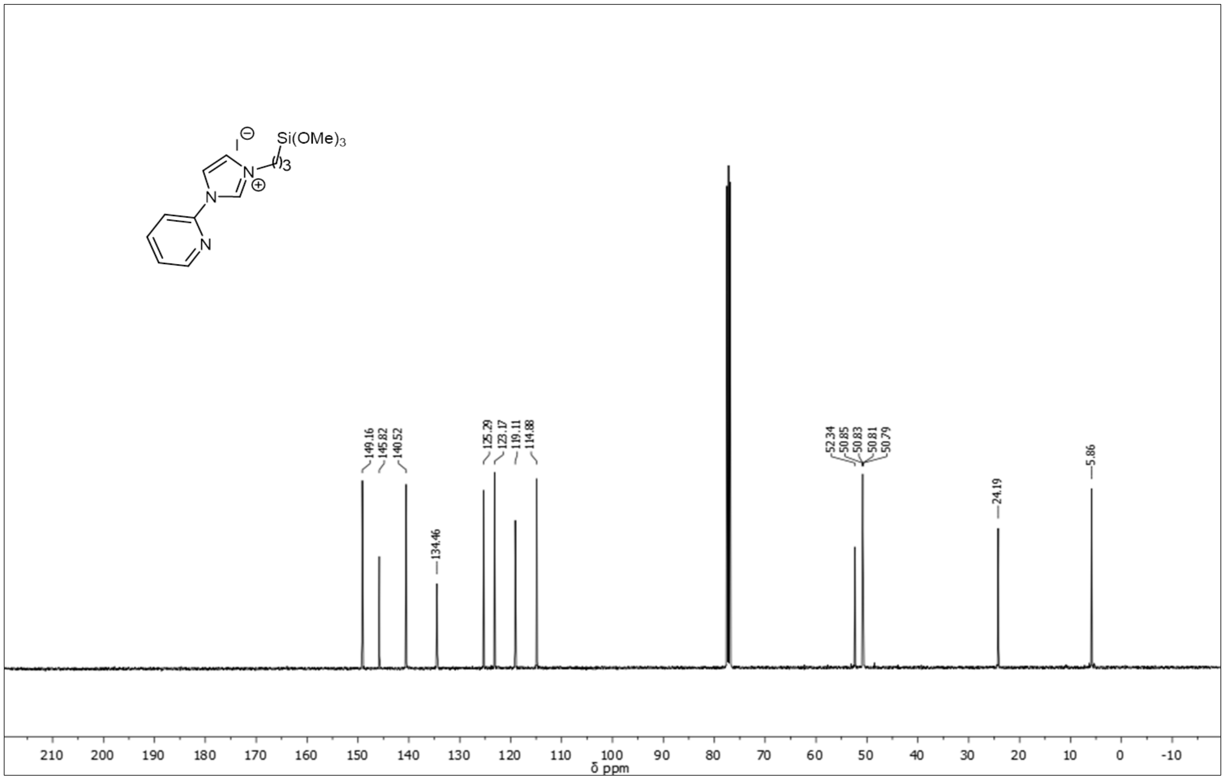

Figure S20. ^{13}C NMR spectrum of **L1** in $CDCl_3$.

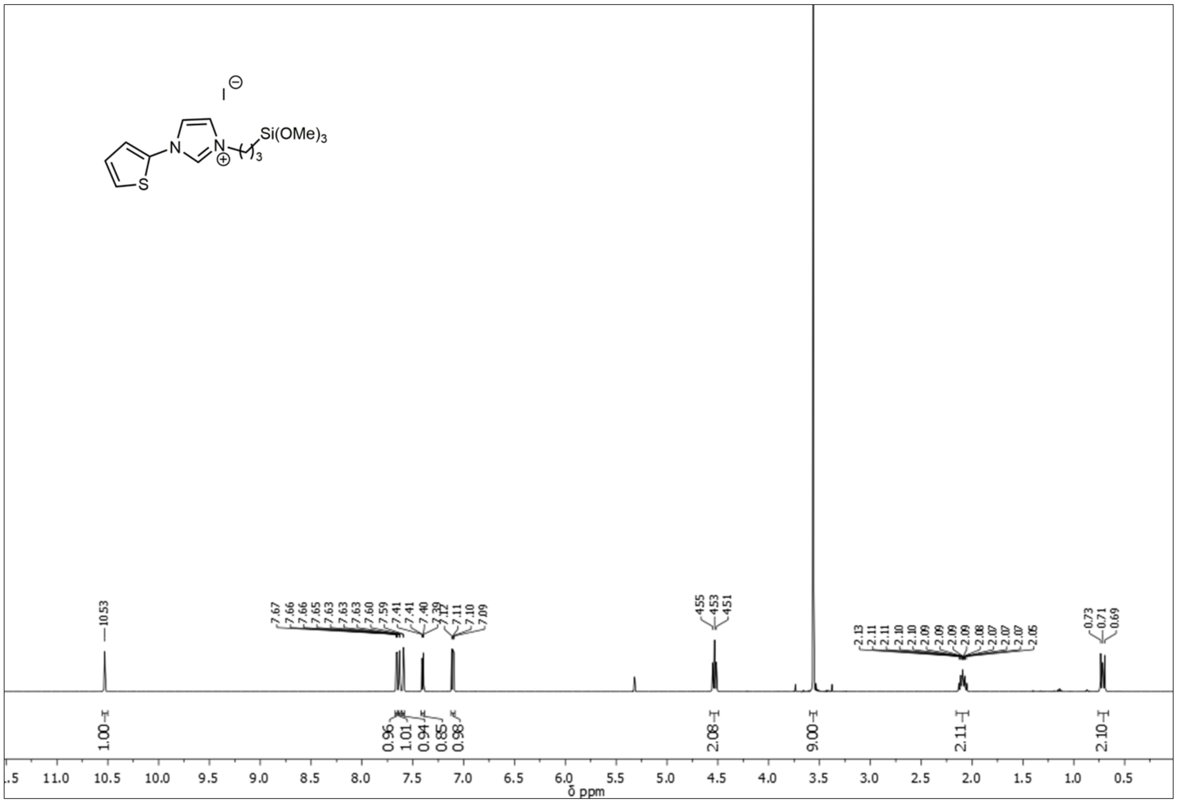

Figure S21. ^{1}H NMR spectrum of **L3** in CD_2Cl_2.

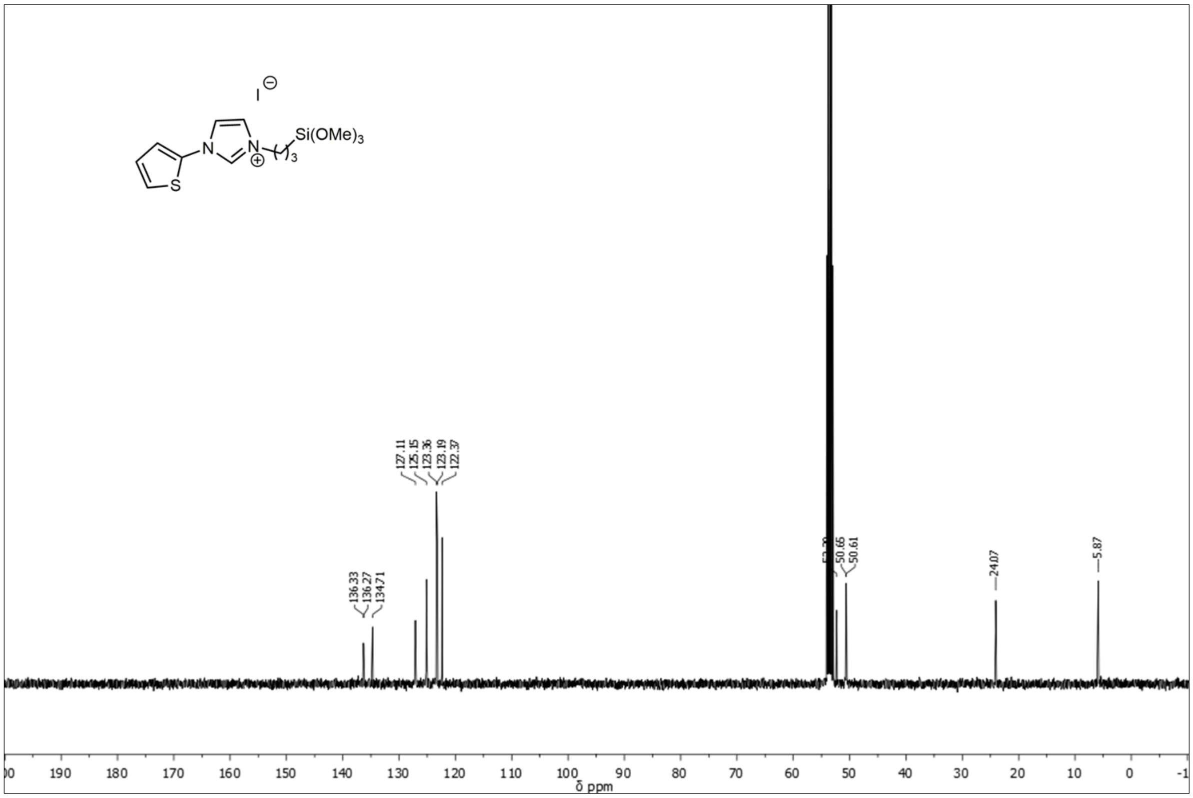

Figure S22. ^{13}C NMR spectrum of **L3** in CD_2Cl_2.

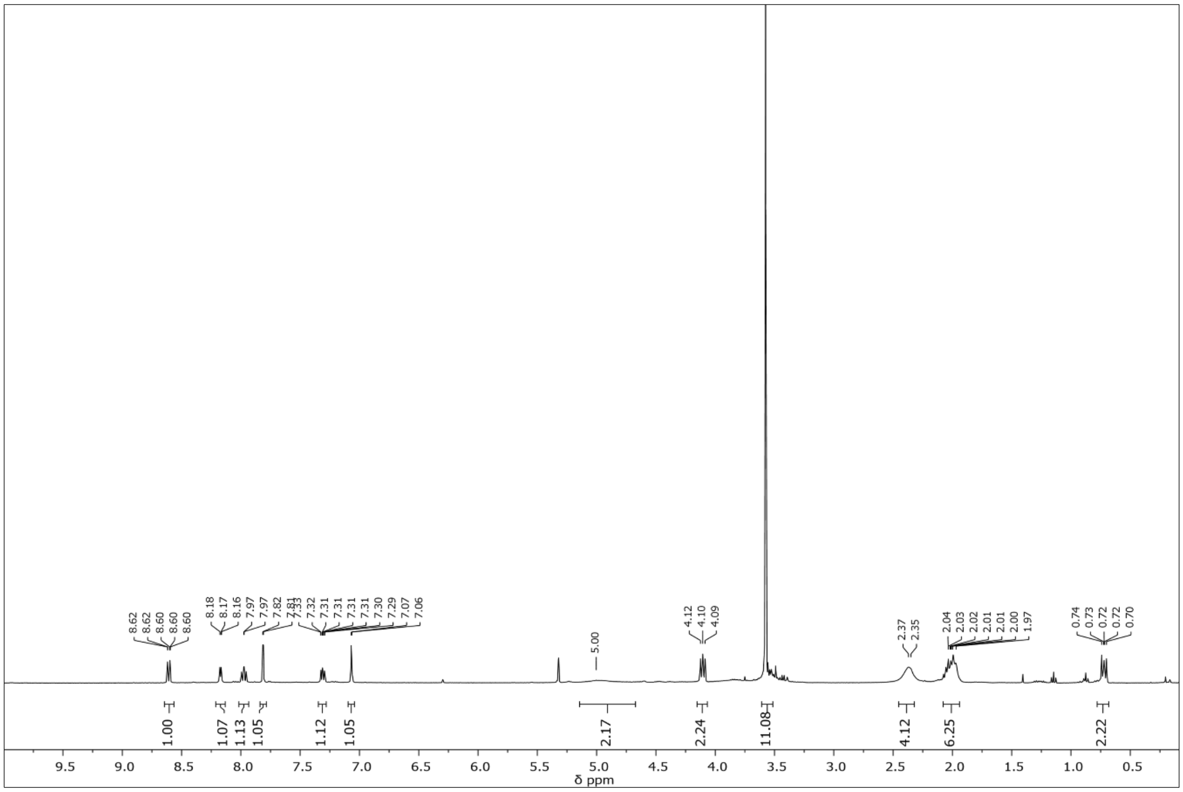

Figure S23. ^{1}H NMR spectrum of **Rh1** in CD_2Cl_2.

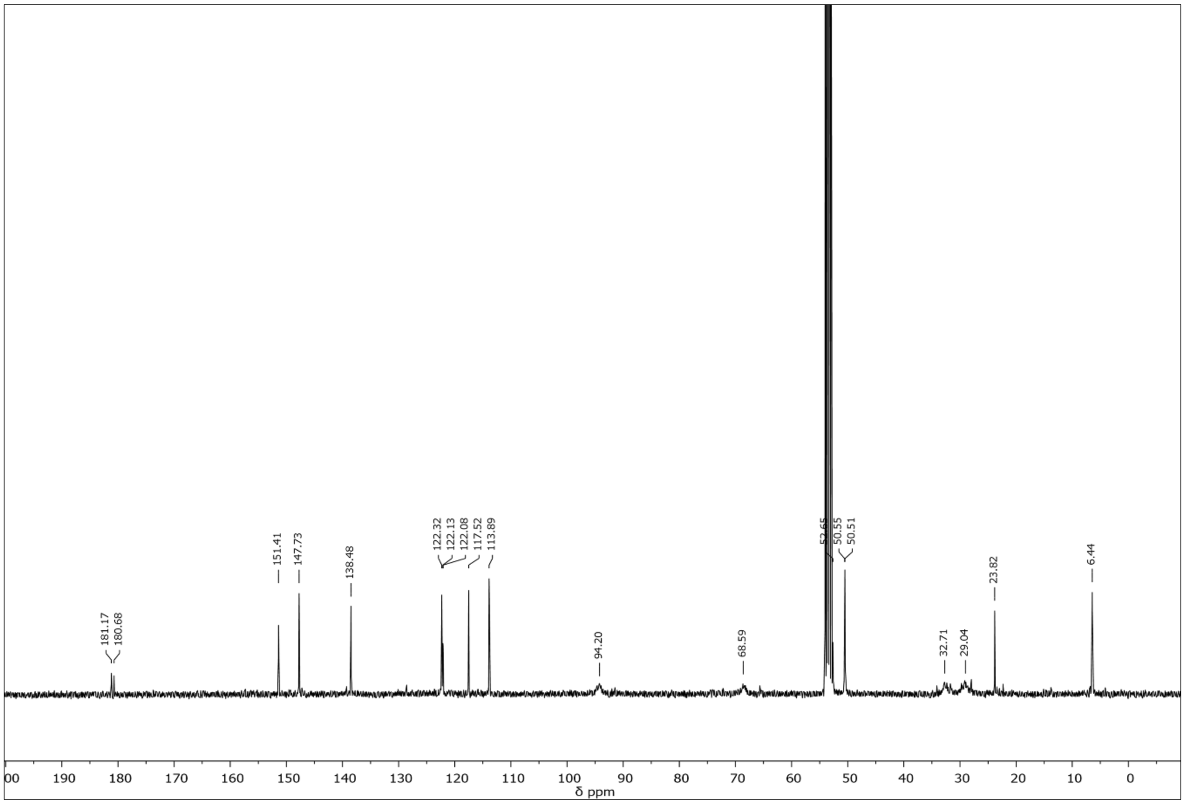

Figure S24. ^{13}C NMR spectrum of **Rh1** in CD_2Cl_2.

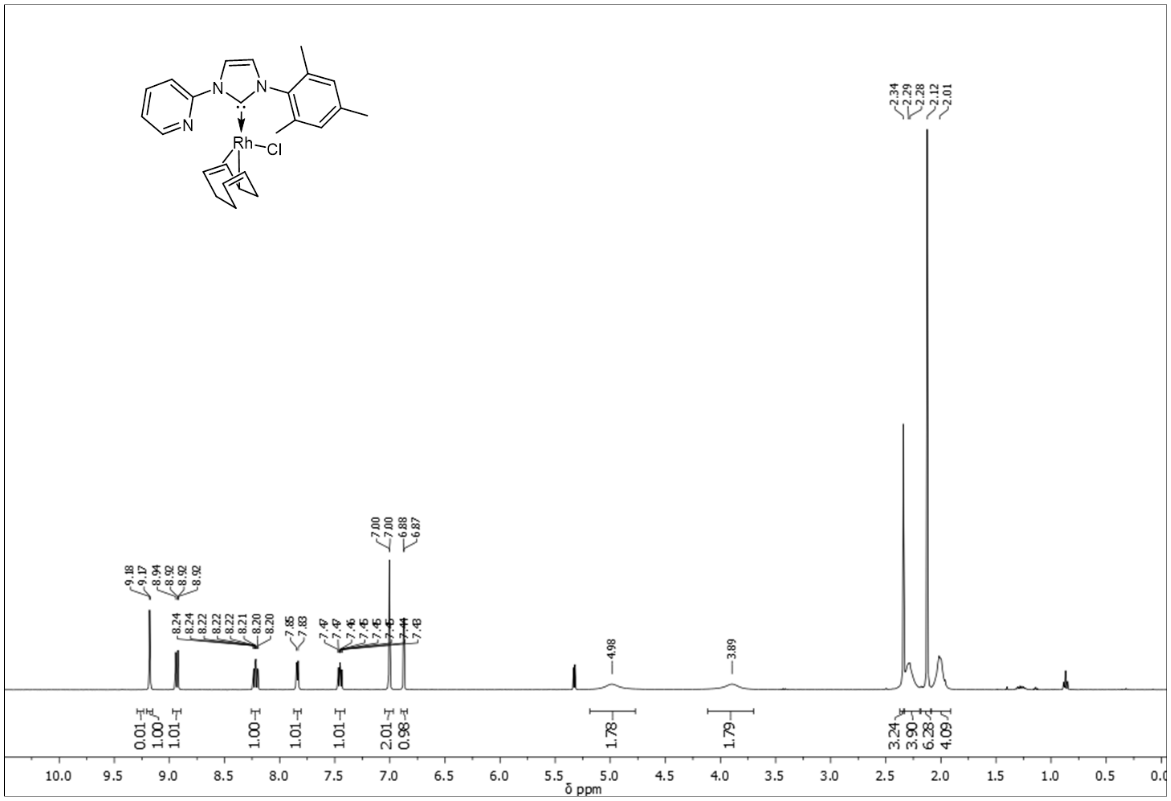

Figure S25. ^{1}H NMR spectrum of **Rh2** in CD_2Cl_2.

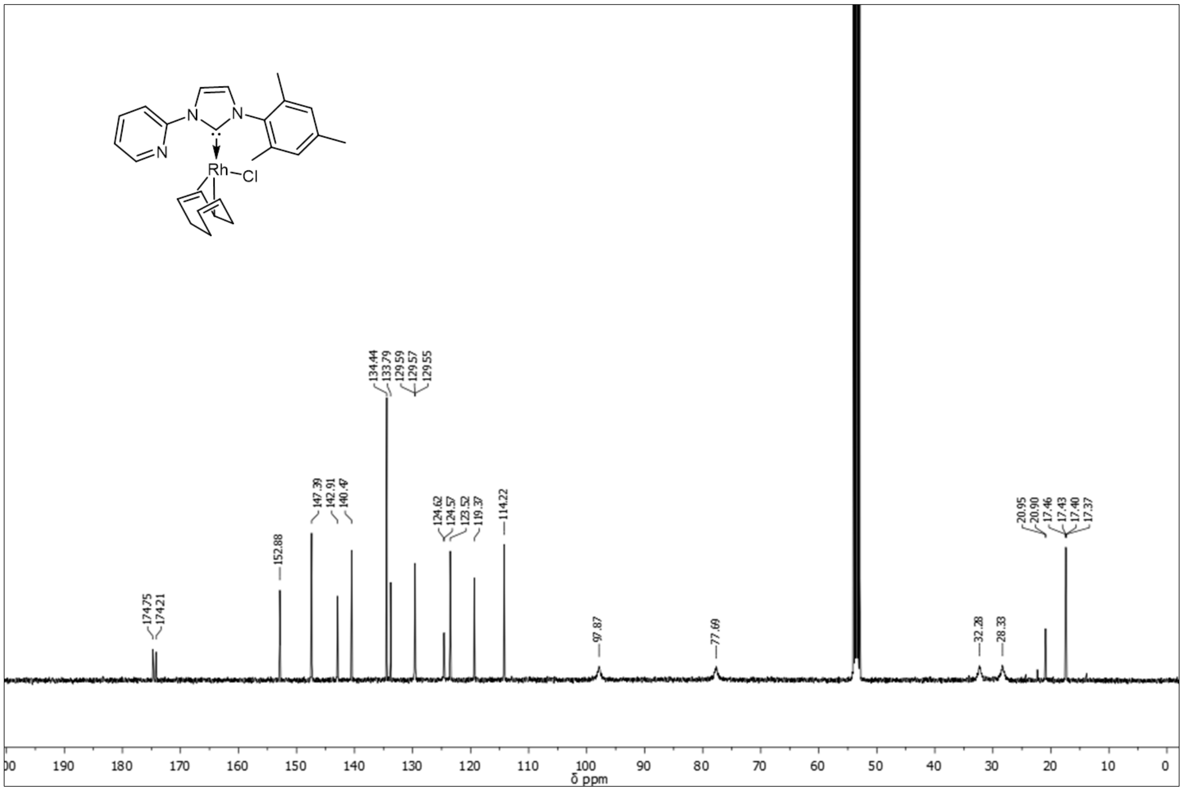

Figure S26. ^{13}C NMR spectrum of **Rh2** in CD_2Cl_2.

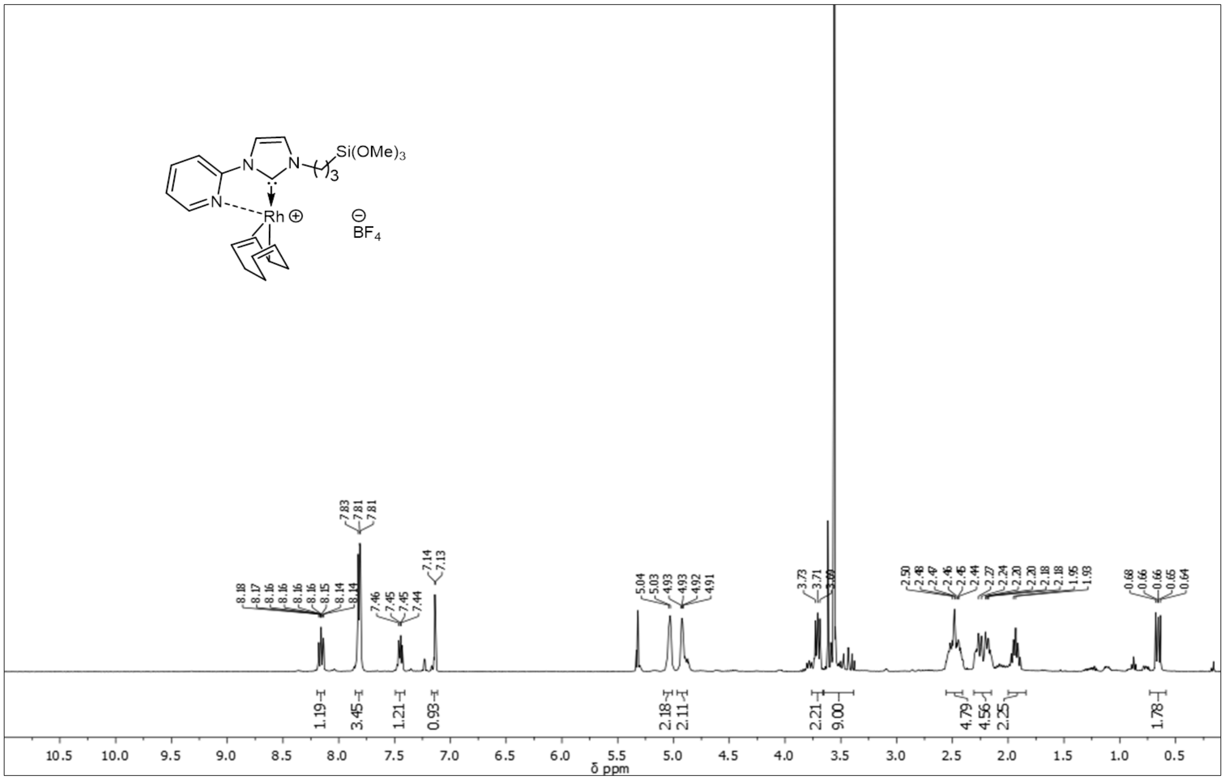

Figure S27. ^{1}H NMR spectrum of **Rh3** in CD_2Cl_2.

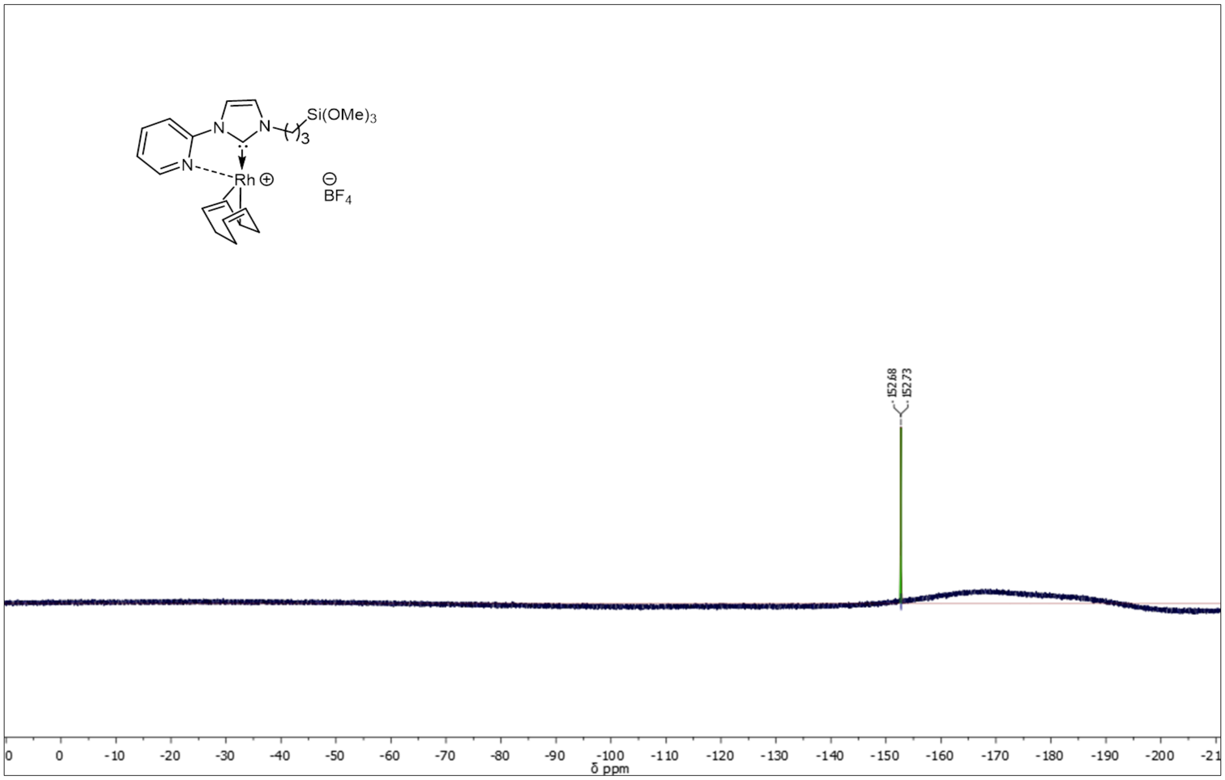

Figure S28. ^{19}F NMR spectrum of **Rh3** in CD_2Cl_2.

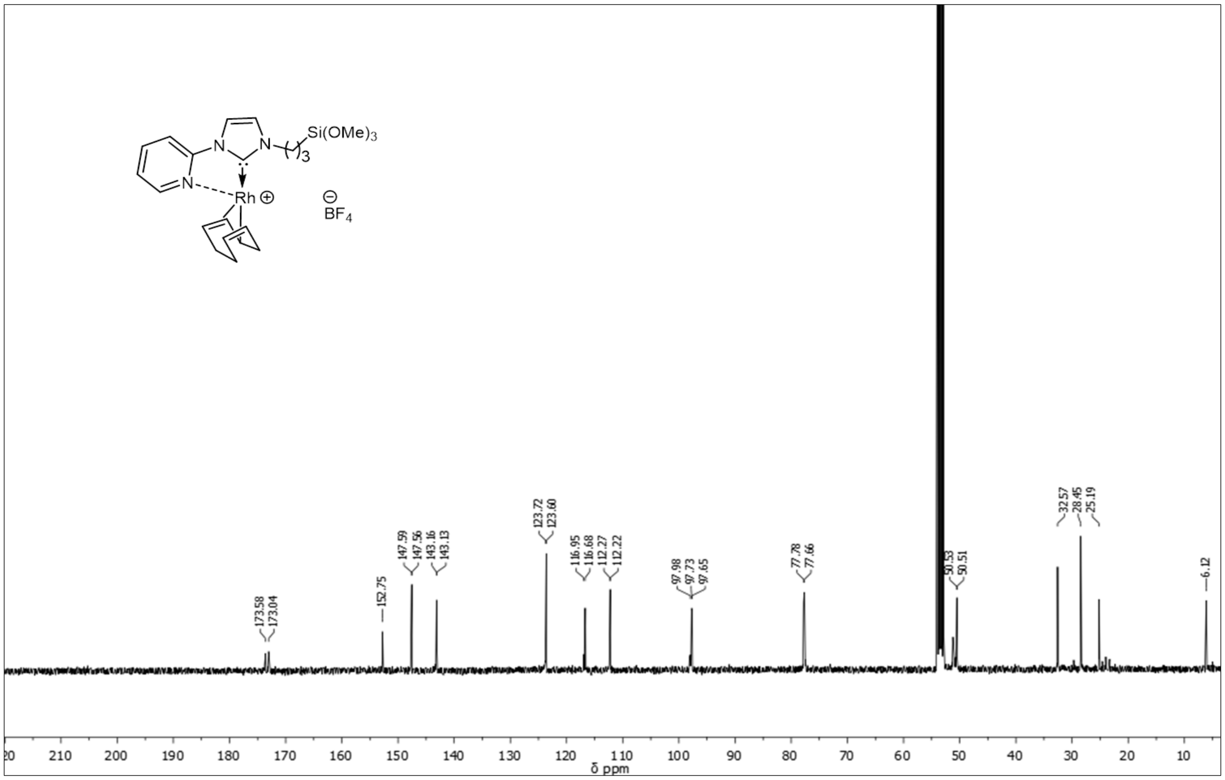

Figure S29. ^{13}C NMR spectrum of **Rh3** in CD_2Cl_2.

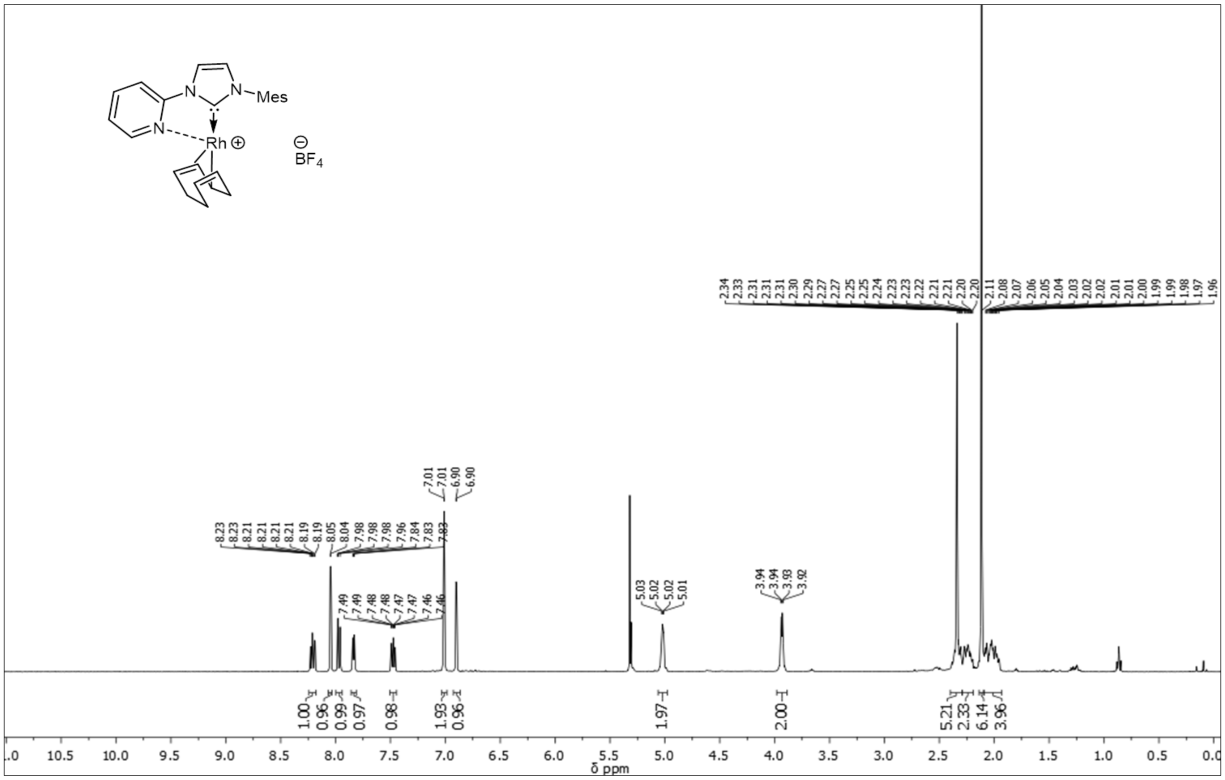

Figure S30. ^{1}H NMR spectrum of **Rh4** in CD_2Cl_2.

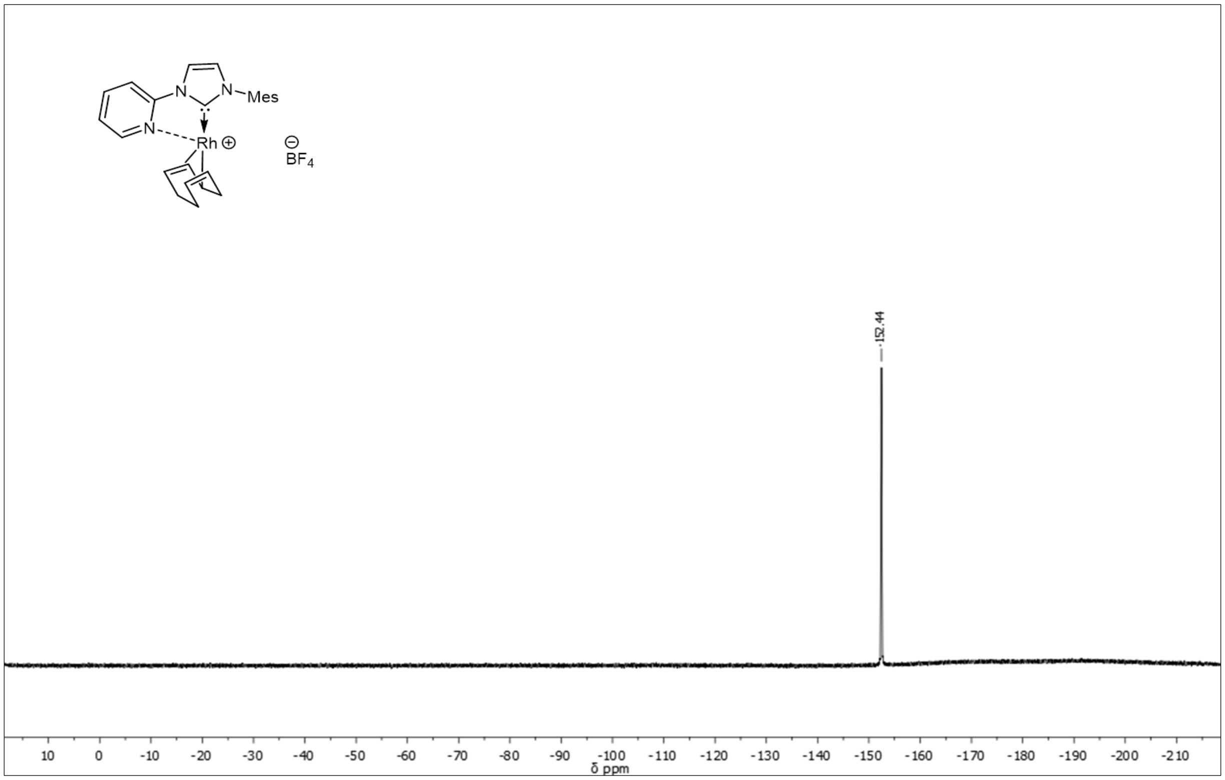

Figure S31. ^{19}F NMR spectrum of **Rh4** in CD_2Cl_2.

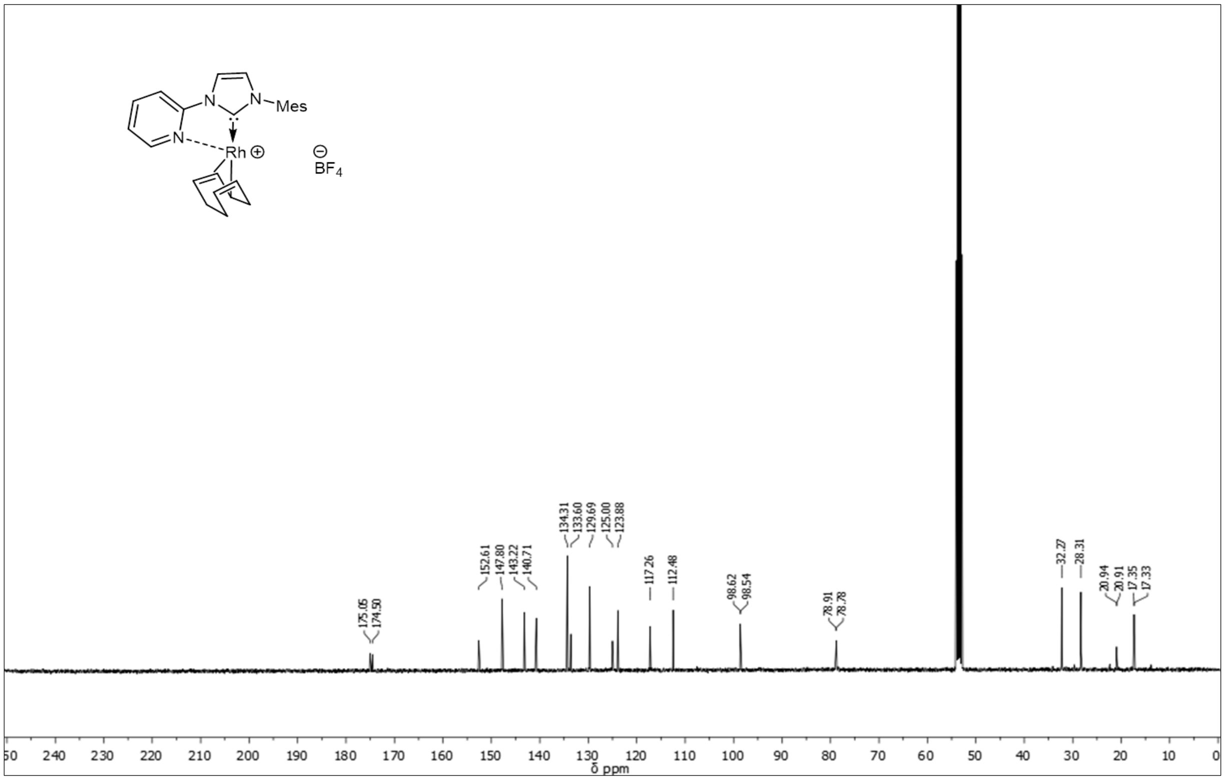

Figure S32. ^{13}C NMR spectrum of **Rh4** in CD_2Cl_2.

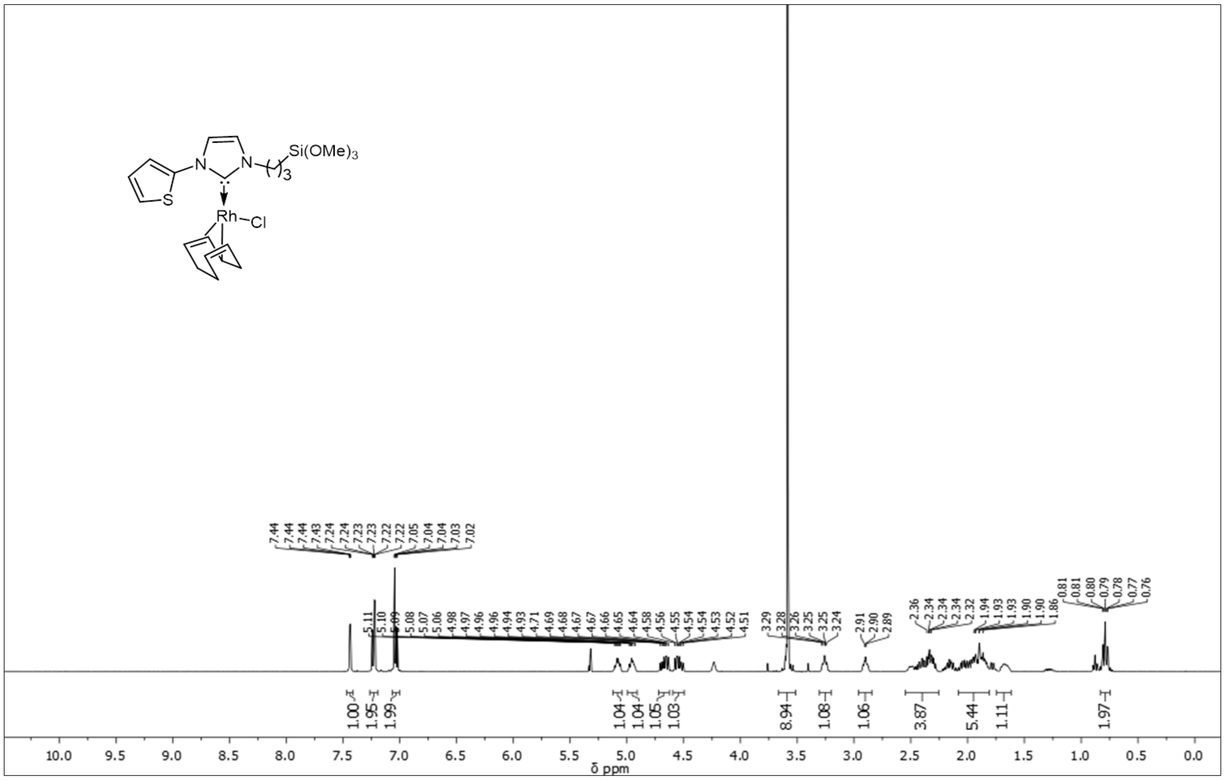

Figure S33. ^{1}H NMR spectrum of **Rh5** in CD_2Cl_2.

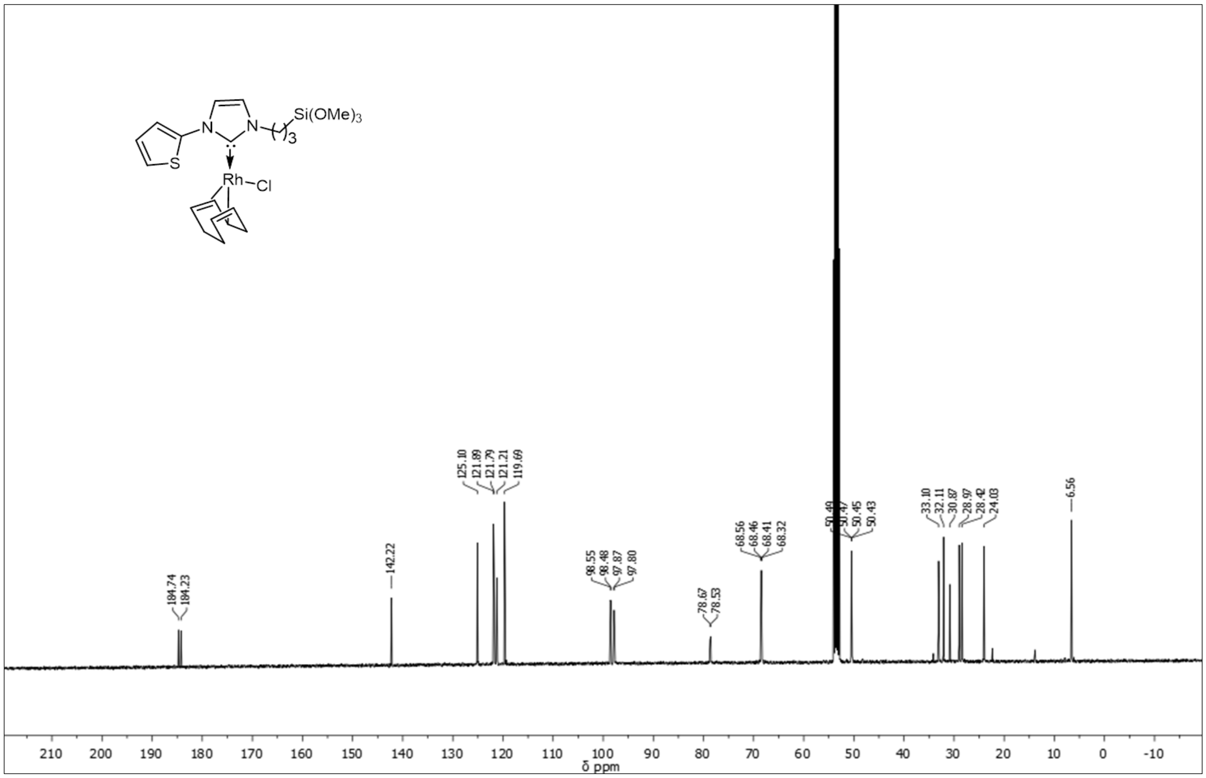

Figure S34. ^{13}C NMR spectrum of **Rh5** in CD_2Cl_2.

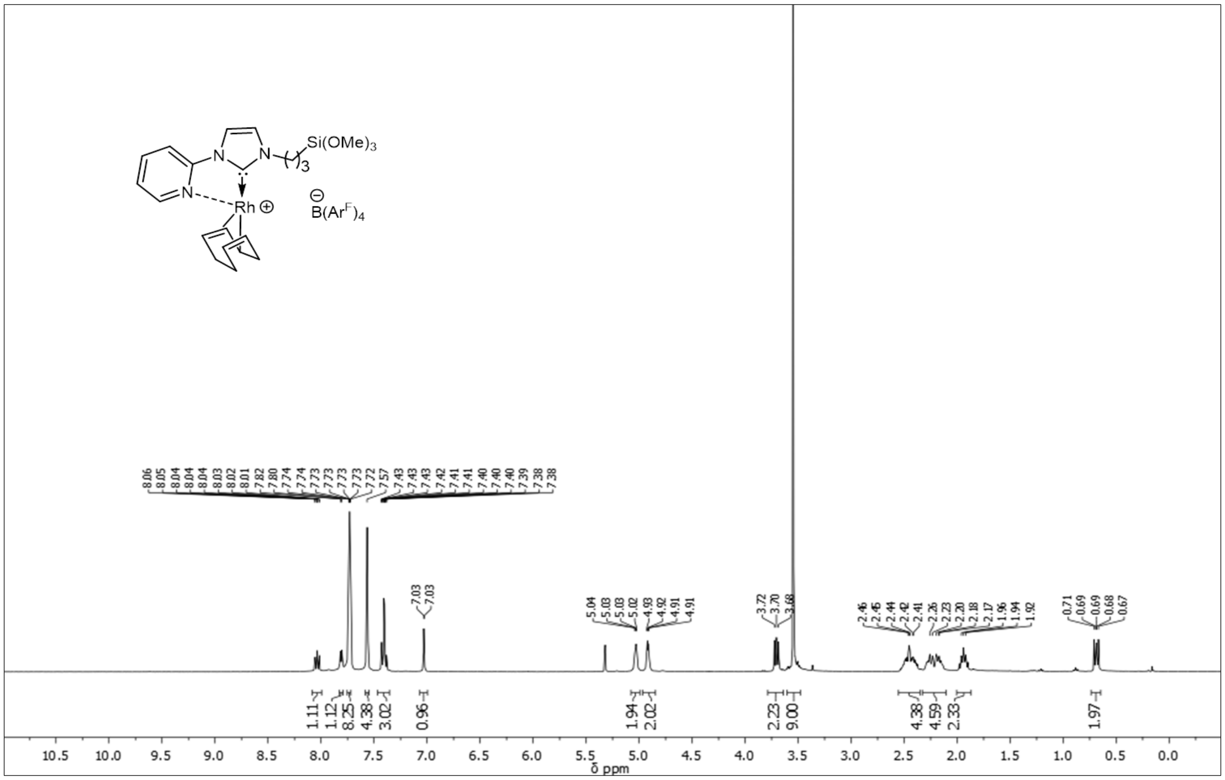

Figure S35. ^{1}H NMR spectrum of **Rh6** in CD_2Cl_2.

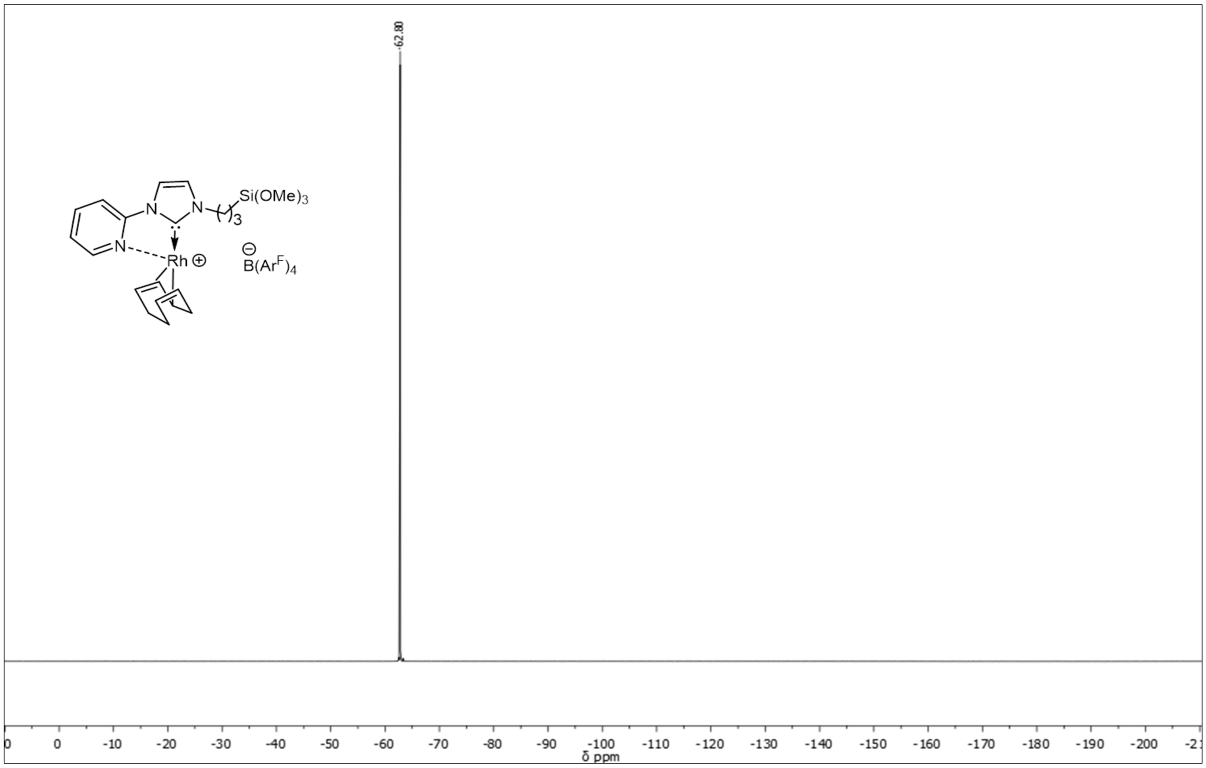

Figure S36. ^{19}F NMR spectrum of **Rh6** in CD_2Cl_2.

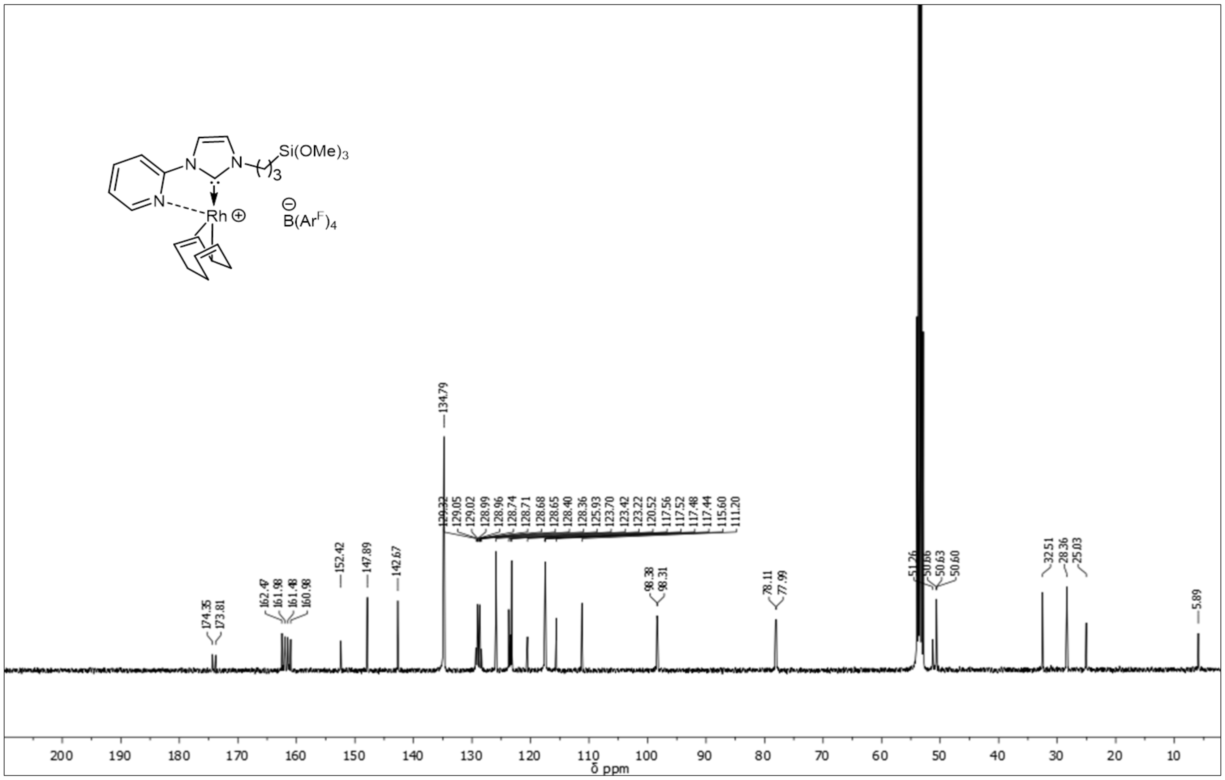

Figure S37. ^{13}C NMR spectrum of **Rh6** in CD_2Cl_2.

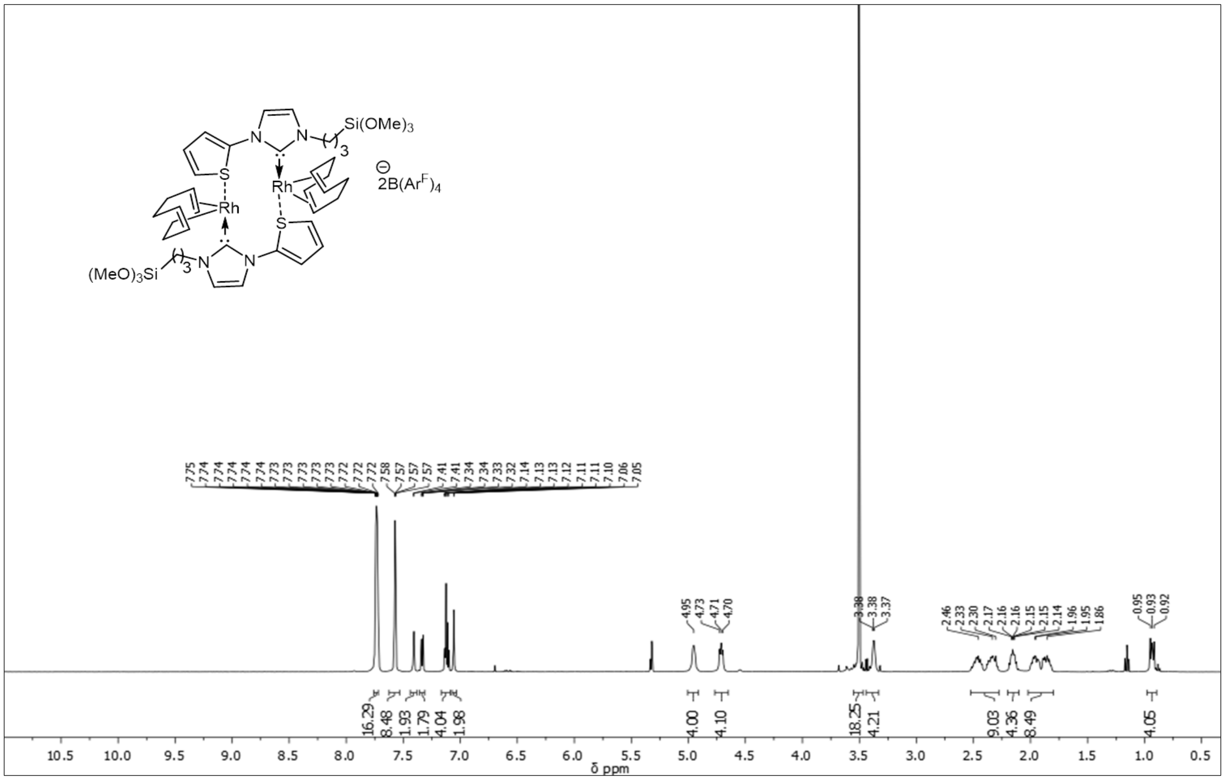

Figure S38. ^{1}H NMR spectrum of **Rh7** in CD_2Cl_2.

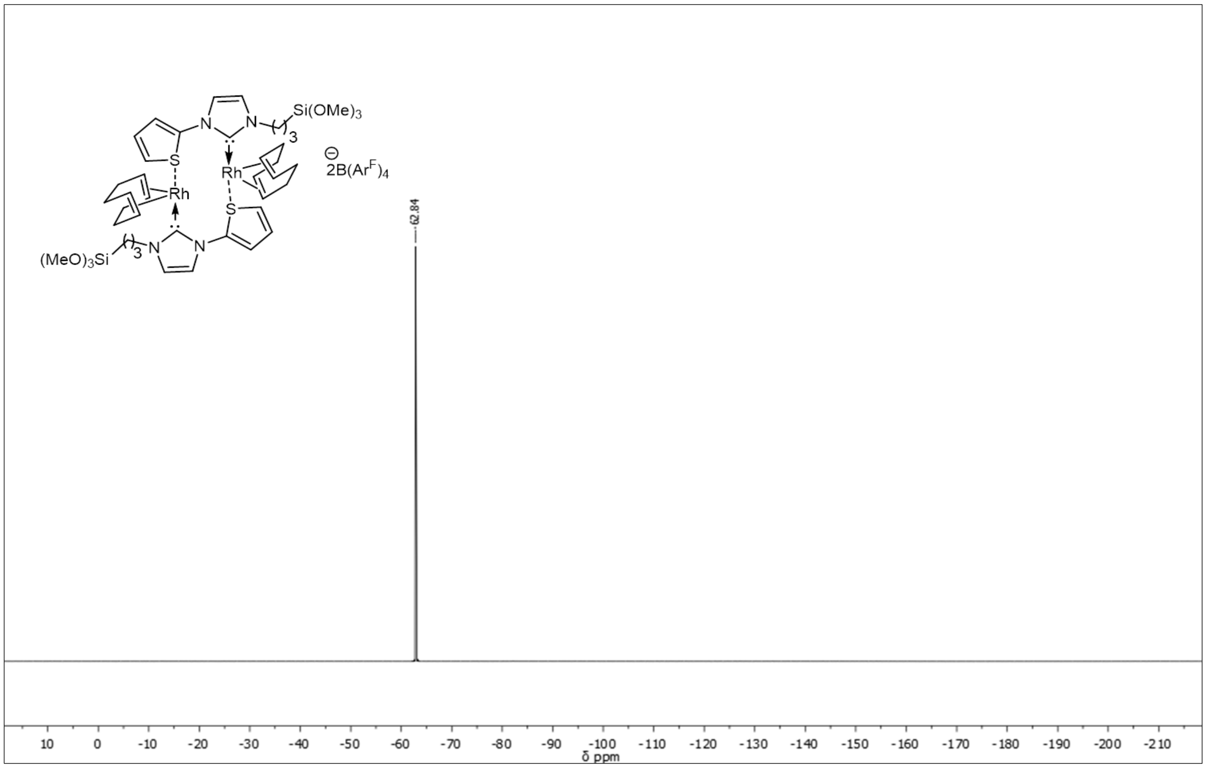

Figure S39. ^{19}F NMR spectrum of **Rh7** in CD_2Cl_2.

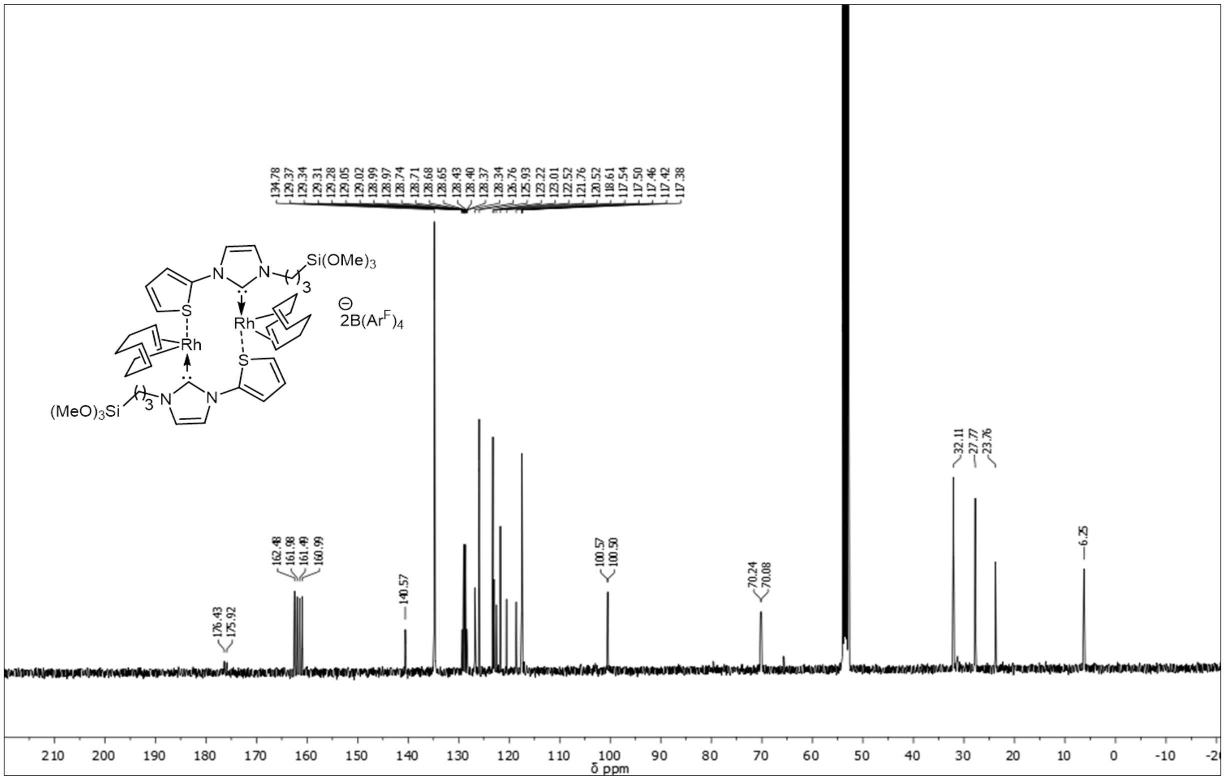

Figure S40. ^{13}C NMR spectrum of **Rh7** in CD_2Cl_2.

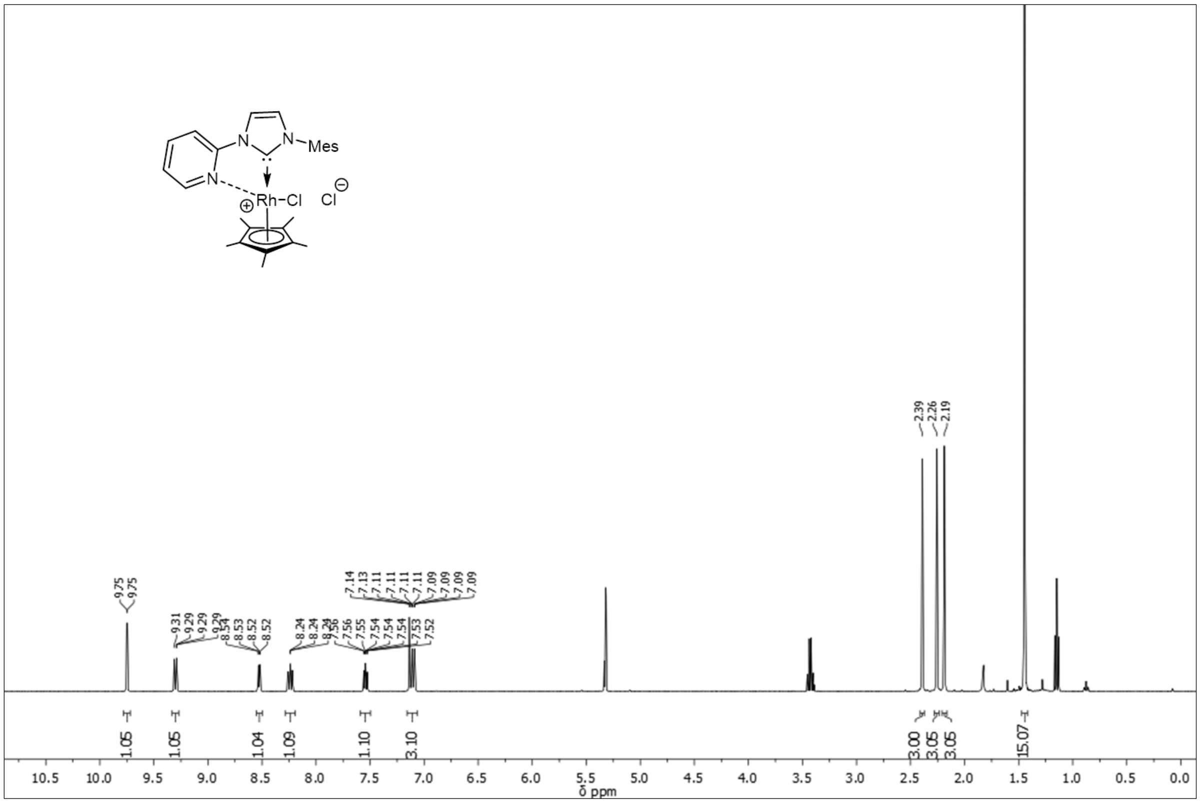

Figure S41. ^{1}H NMR spectrum of **Rh9** in CD_2Cl_2.

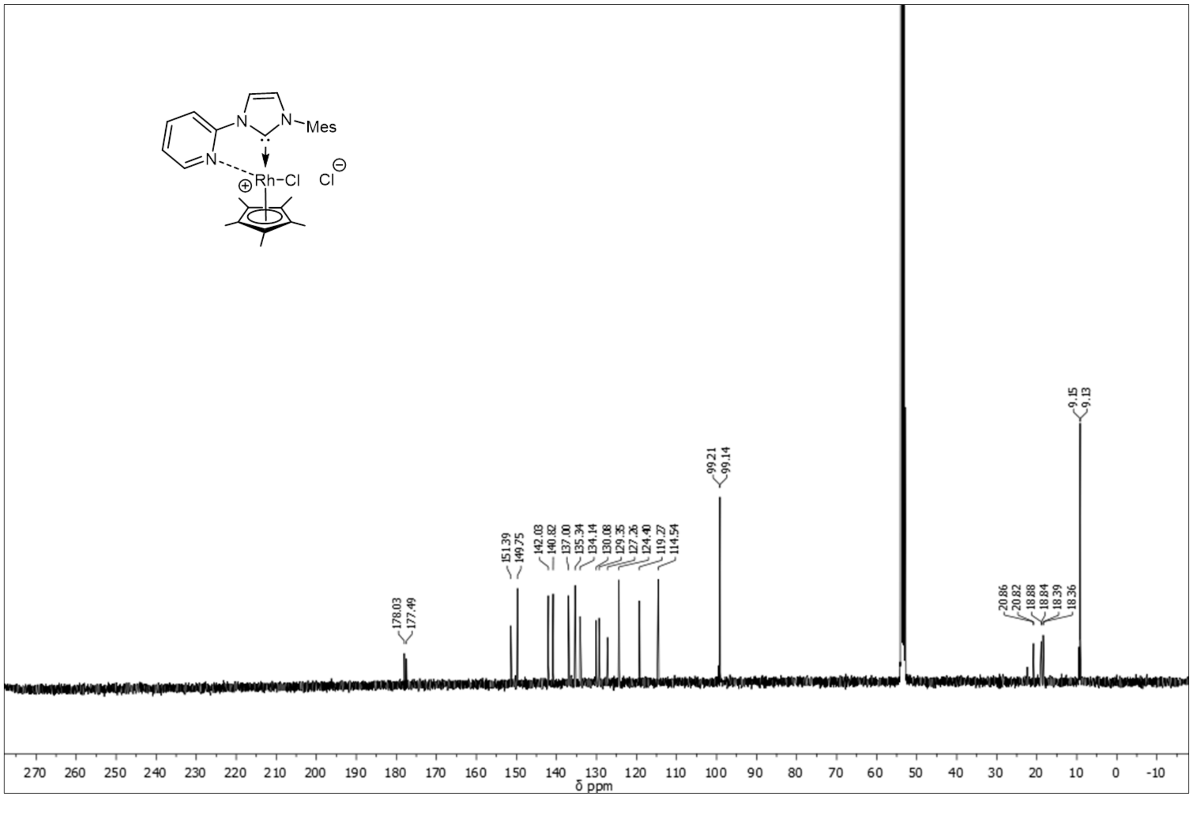

Figure S42. ^{13}C NMR spectrum of **Rh9** in CD_2Cl_2.

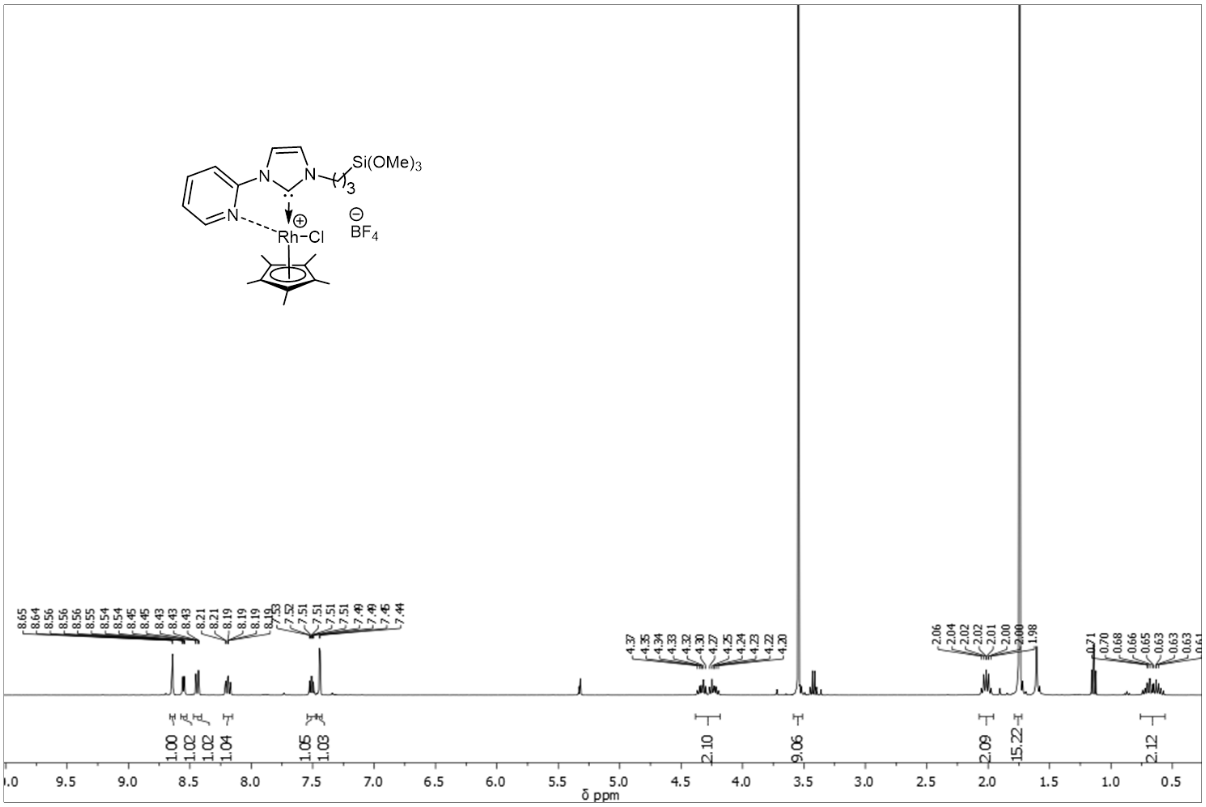

Figure S43. ^{1}H NMR spectrum of **Rh10** in CD_2Cl_2.

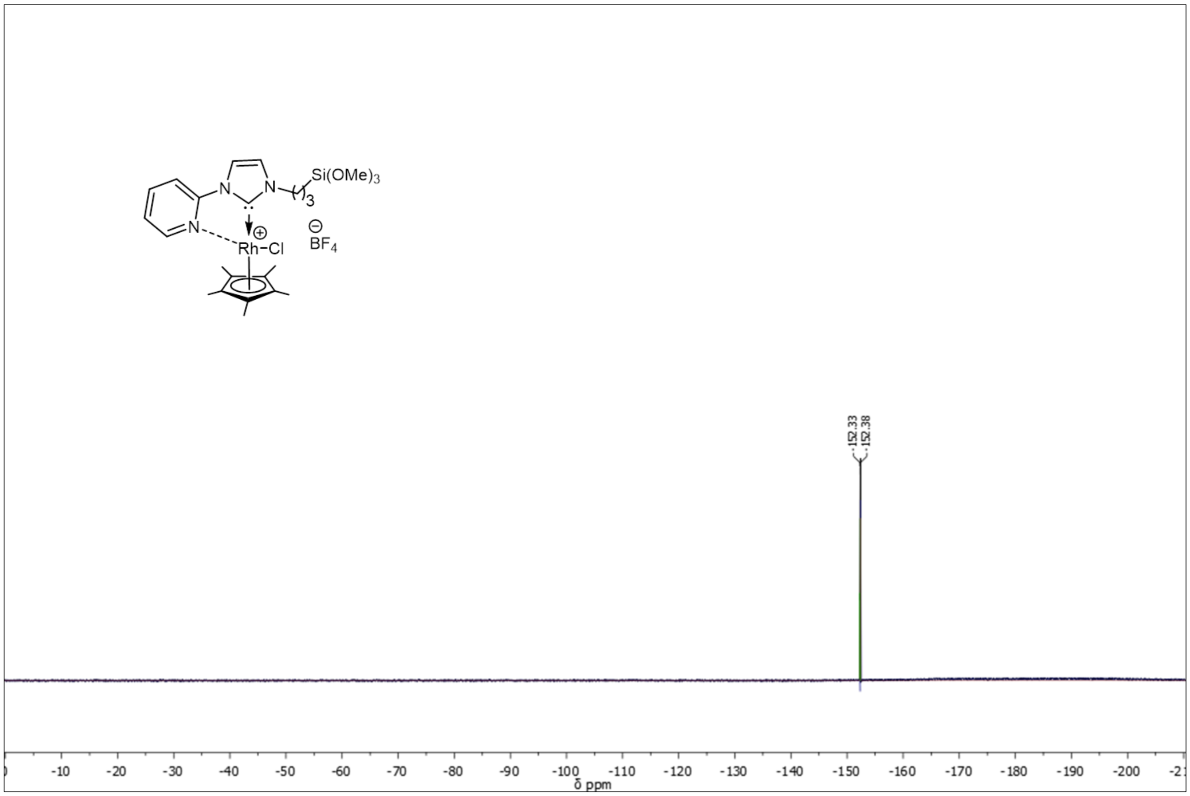

Figure S44. ^{19}F NMR spectrum of **Rh10** in CD_2Cl_2.

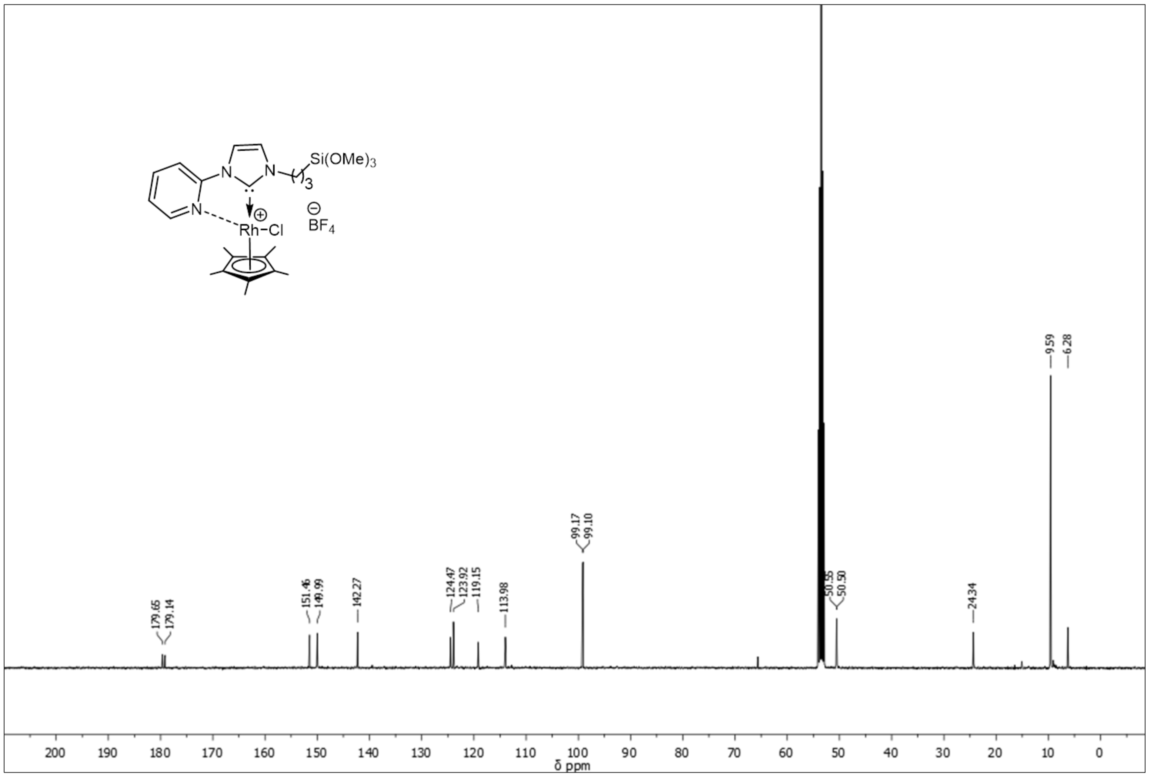

Figure S45. ^{13}C NMR spectrum of **Rh10** in CD_2Cl_2.

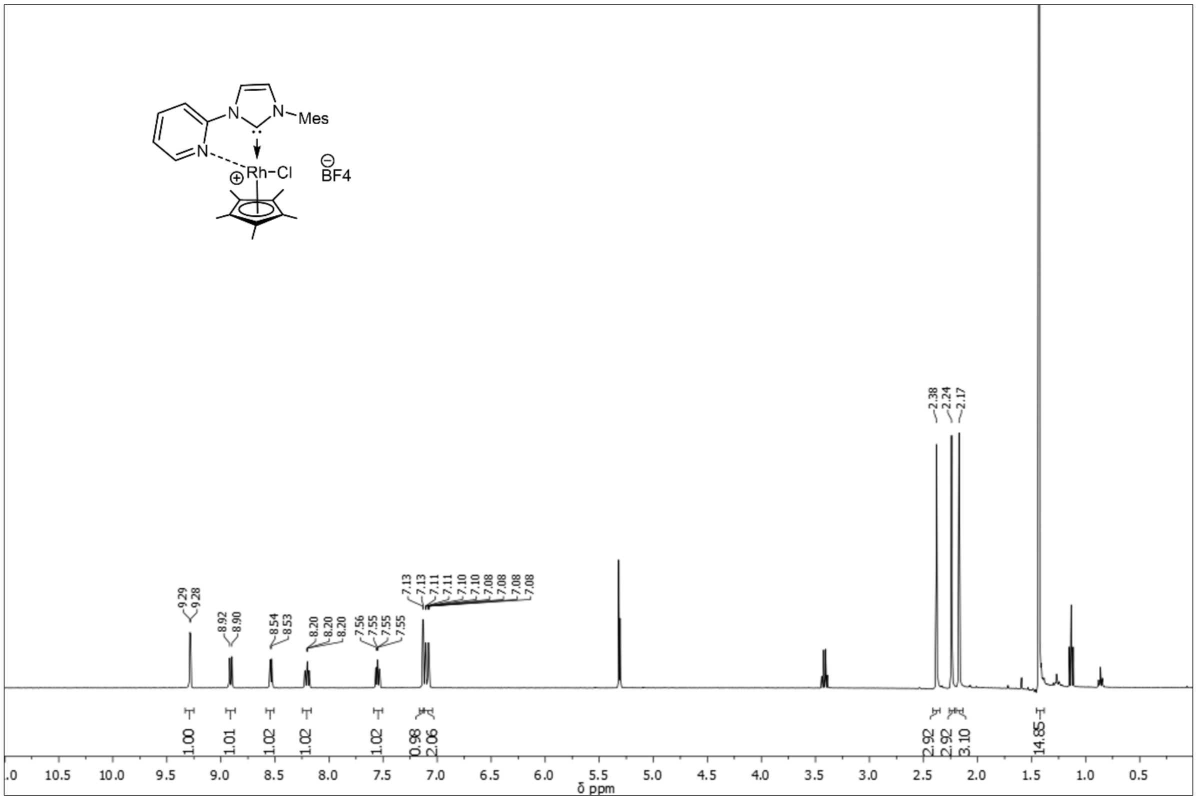

Figure S46. ^{1}H NMR spectrum of **Rh11** in CD_2Cl_2.

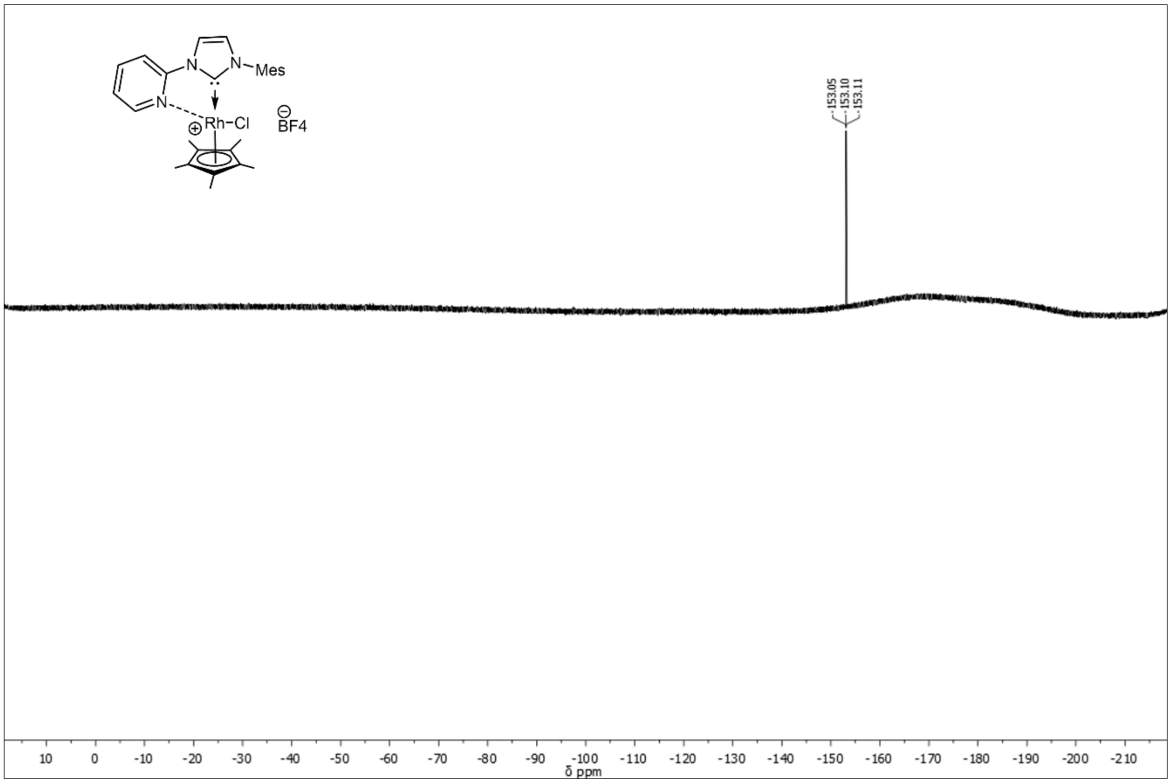

Figure S47. ^{19}F NMR spectrum of **Rh11** in CD_2Cl_2.

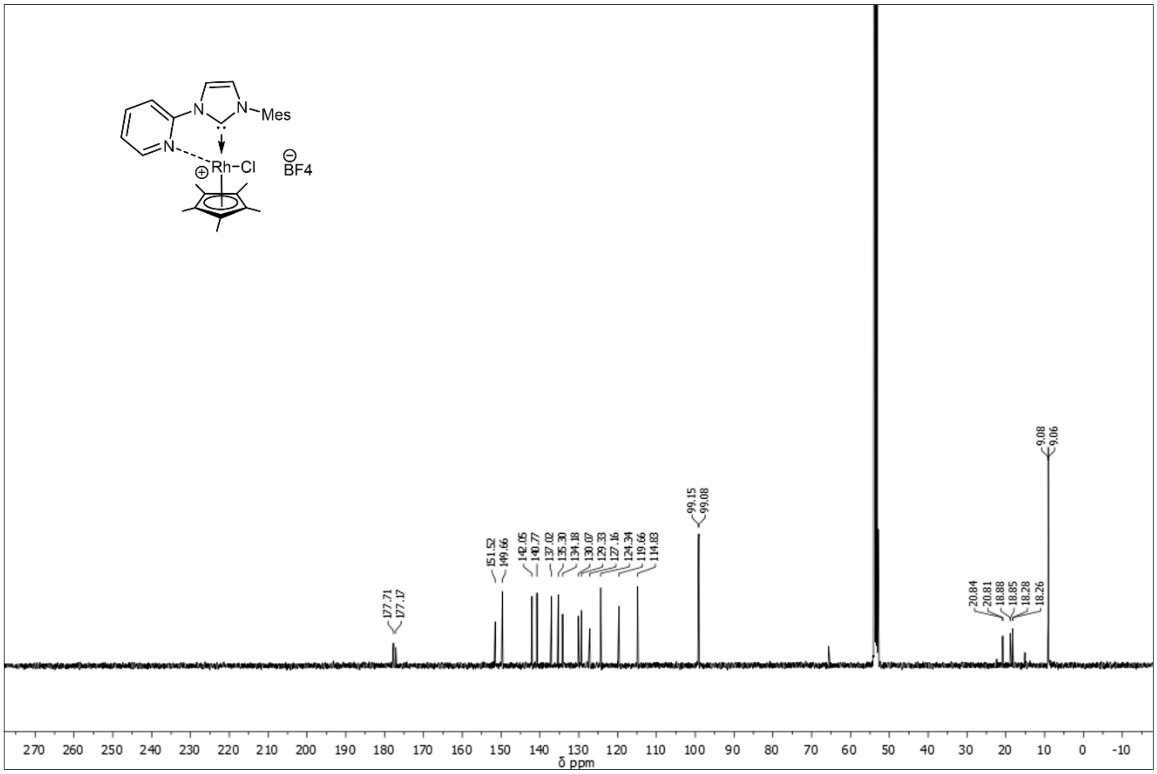

Figure S48. ^{13}C NMR spectrum of **Rh11** in CD_2Cl_2.

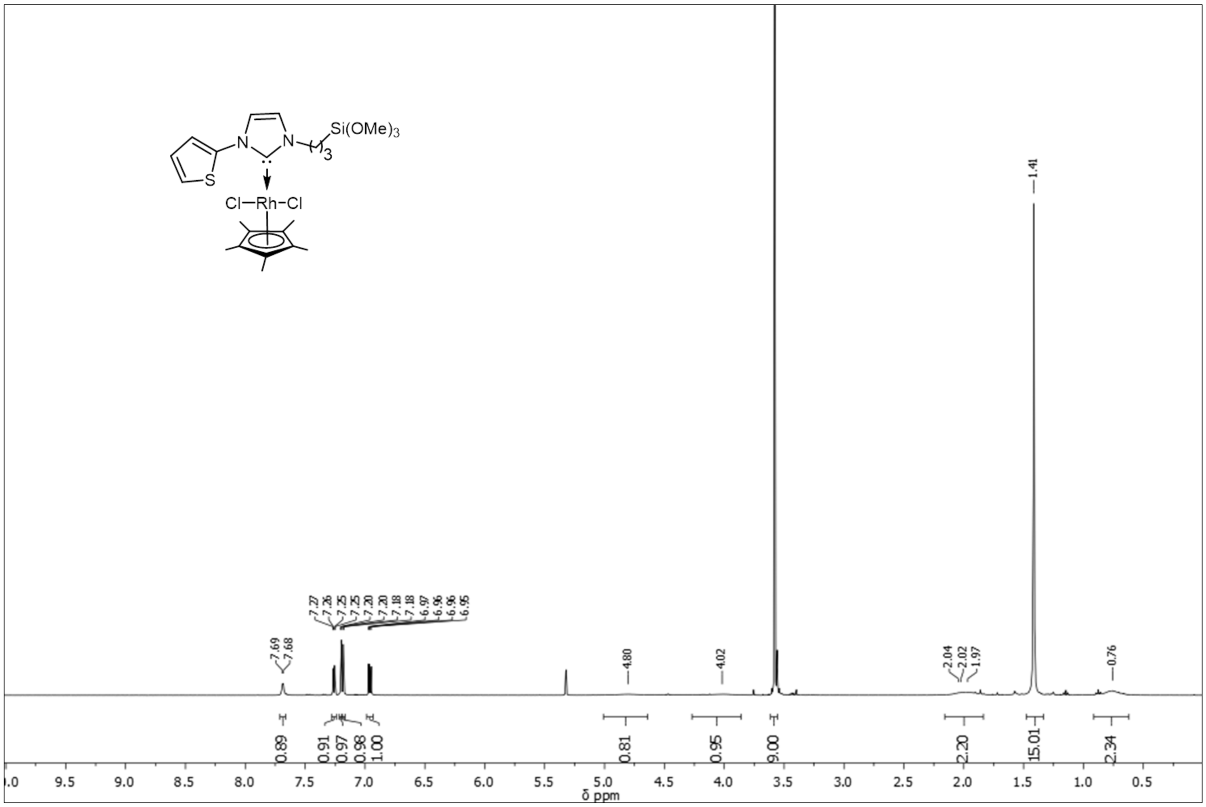

Figure S49. ^{1}H NMR spectrum of **Rh12** in CD_2Cl_2.

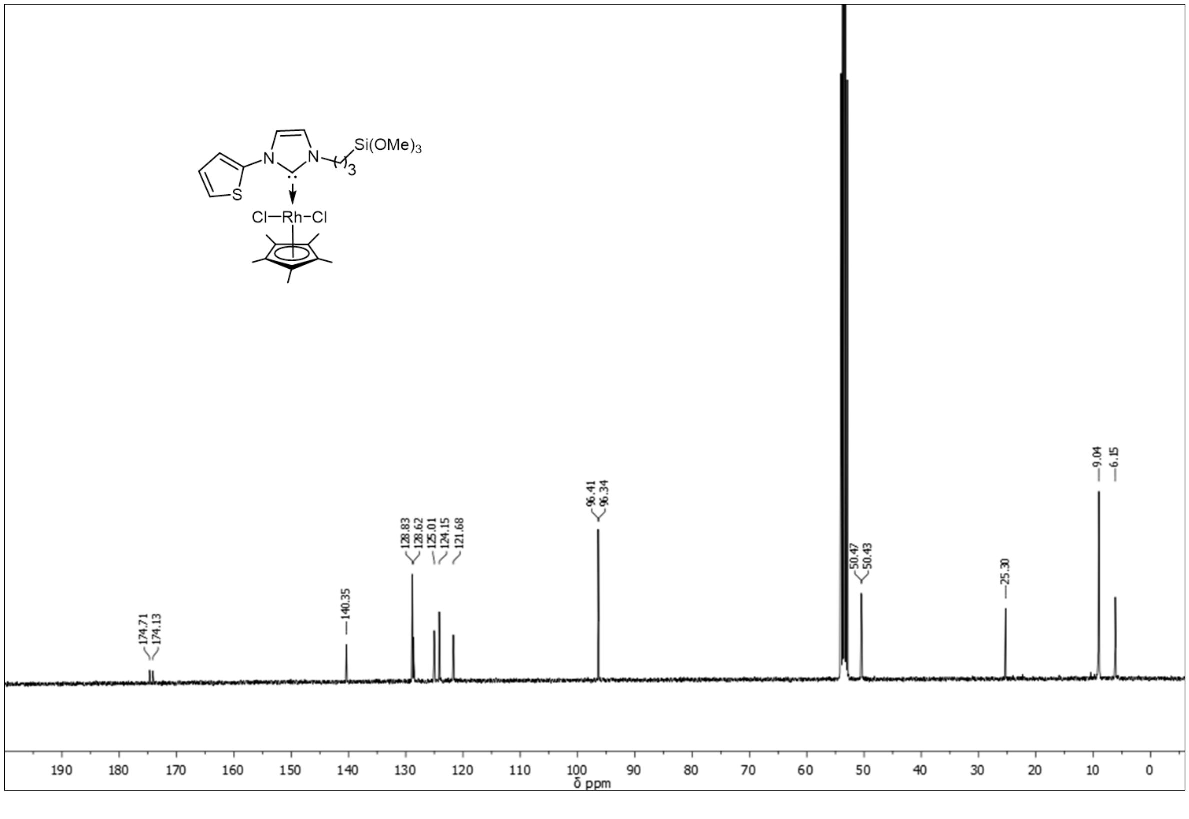

Figure S50. ^{13}C NMR spectrum of **Rh12** in $CDCl_3$.

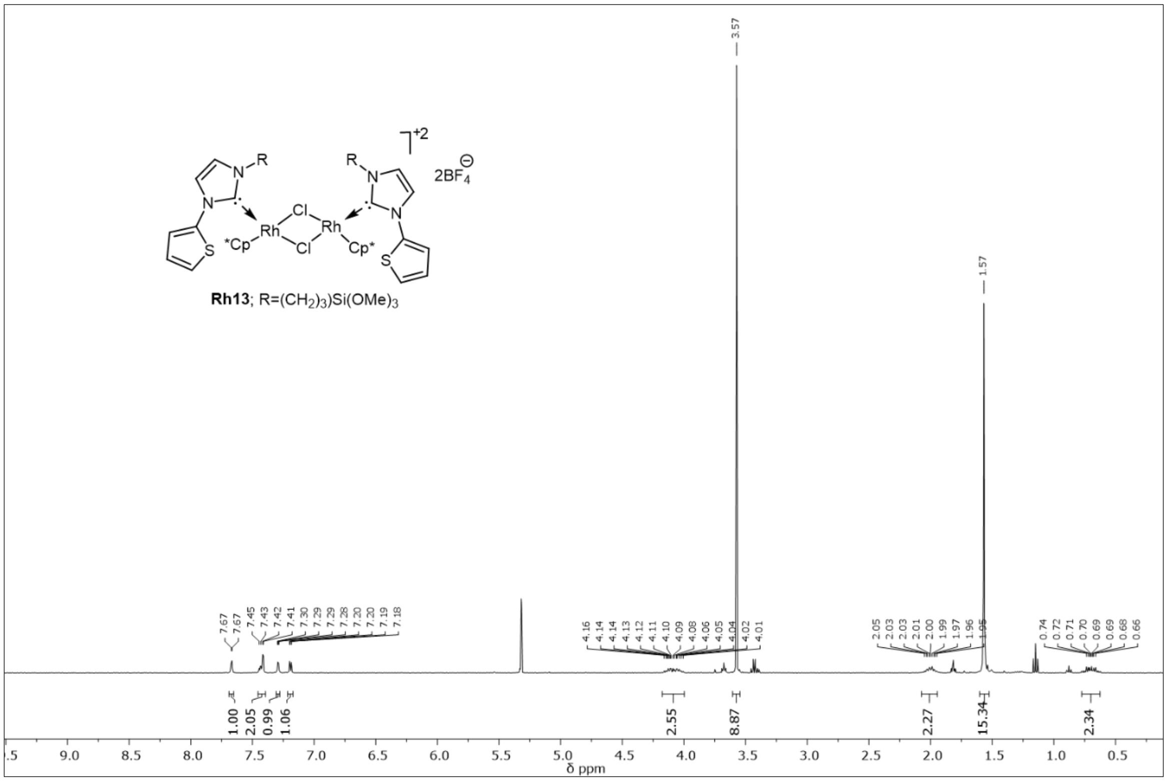

Figure S51. ^{1}H NMR spectrum of **Rh13** in CD_2Cl_2.

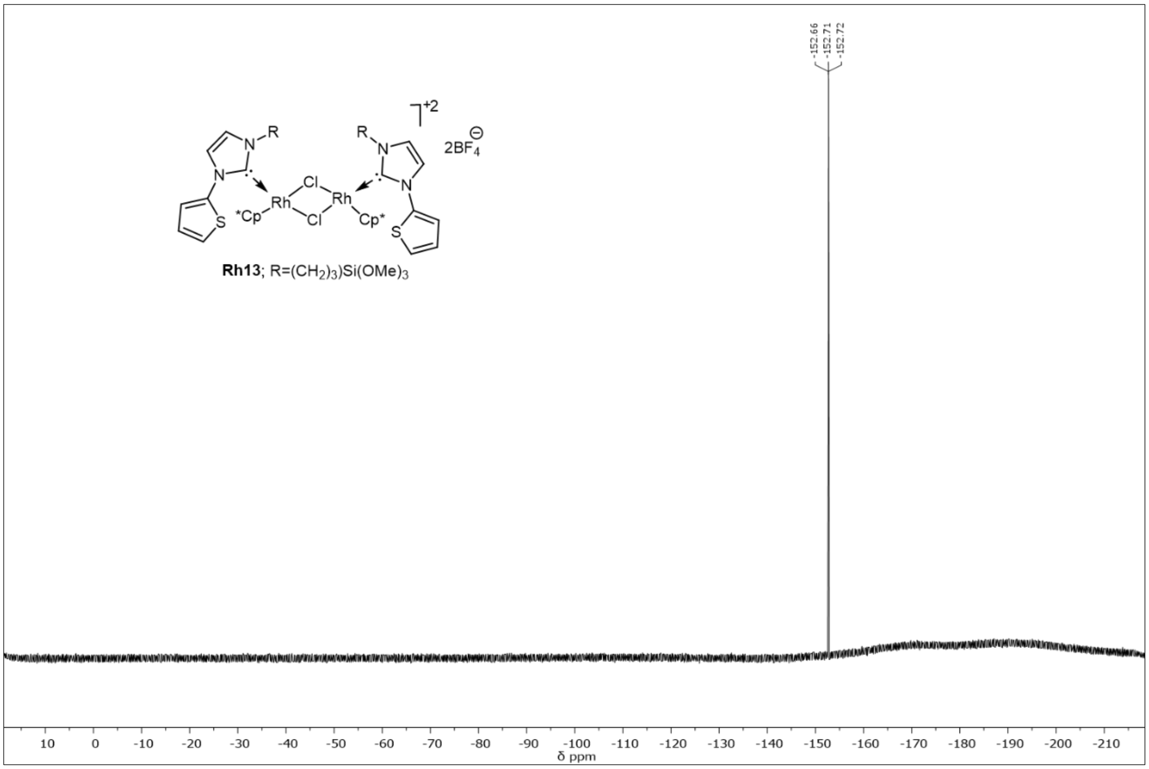

Figure S52. ^{19}F NMR spectrum of **Rh13** in CD_2Cl_2.

High-Resolution Mass Spectra

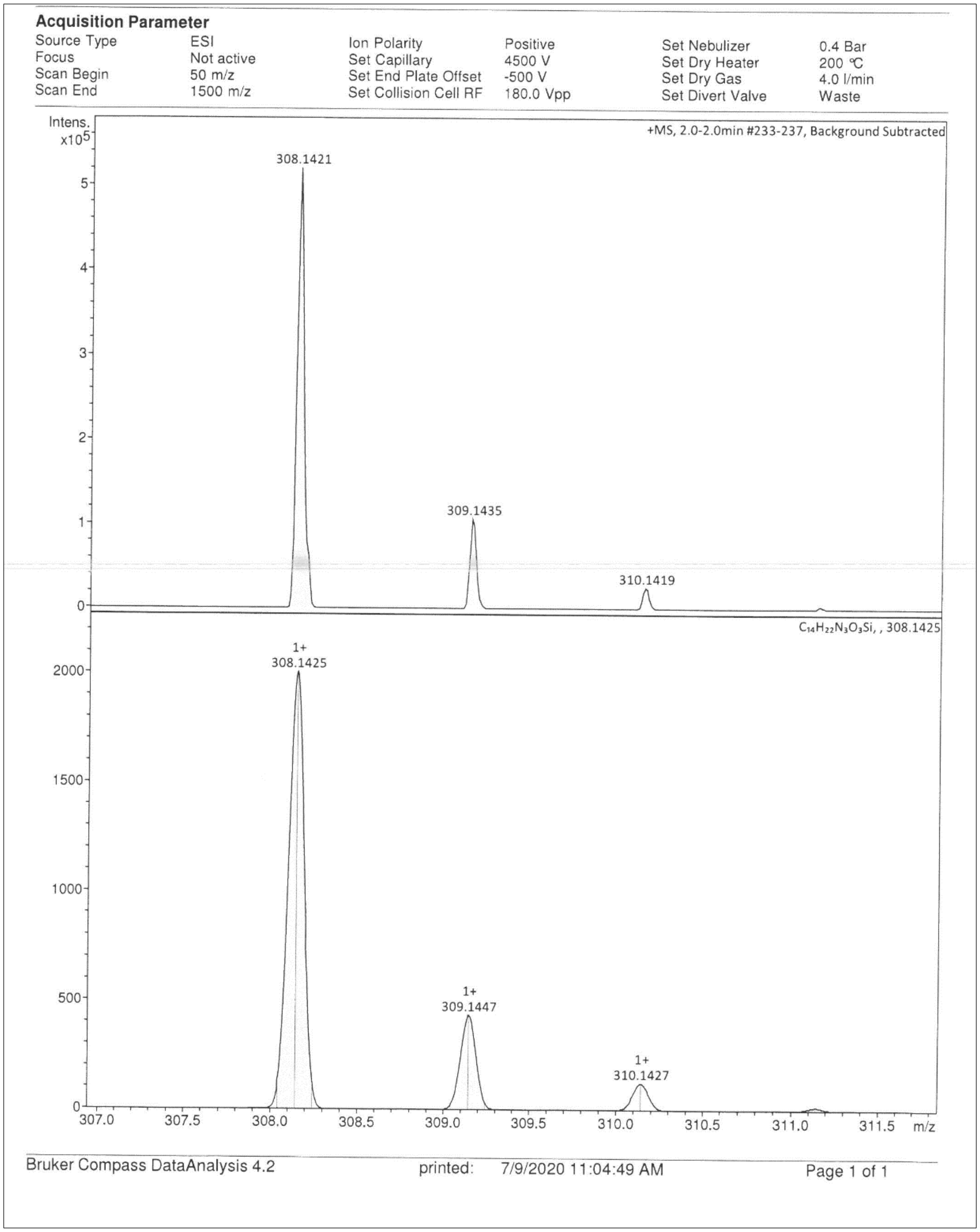

Figure S53. High-resolution mass spectrum of **L1**. Found (top) and calculated (bottom) isotope distribution of the molecular ion peak.

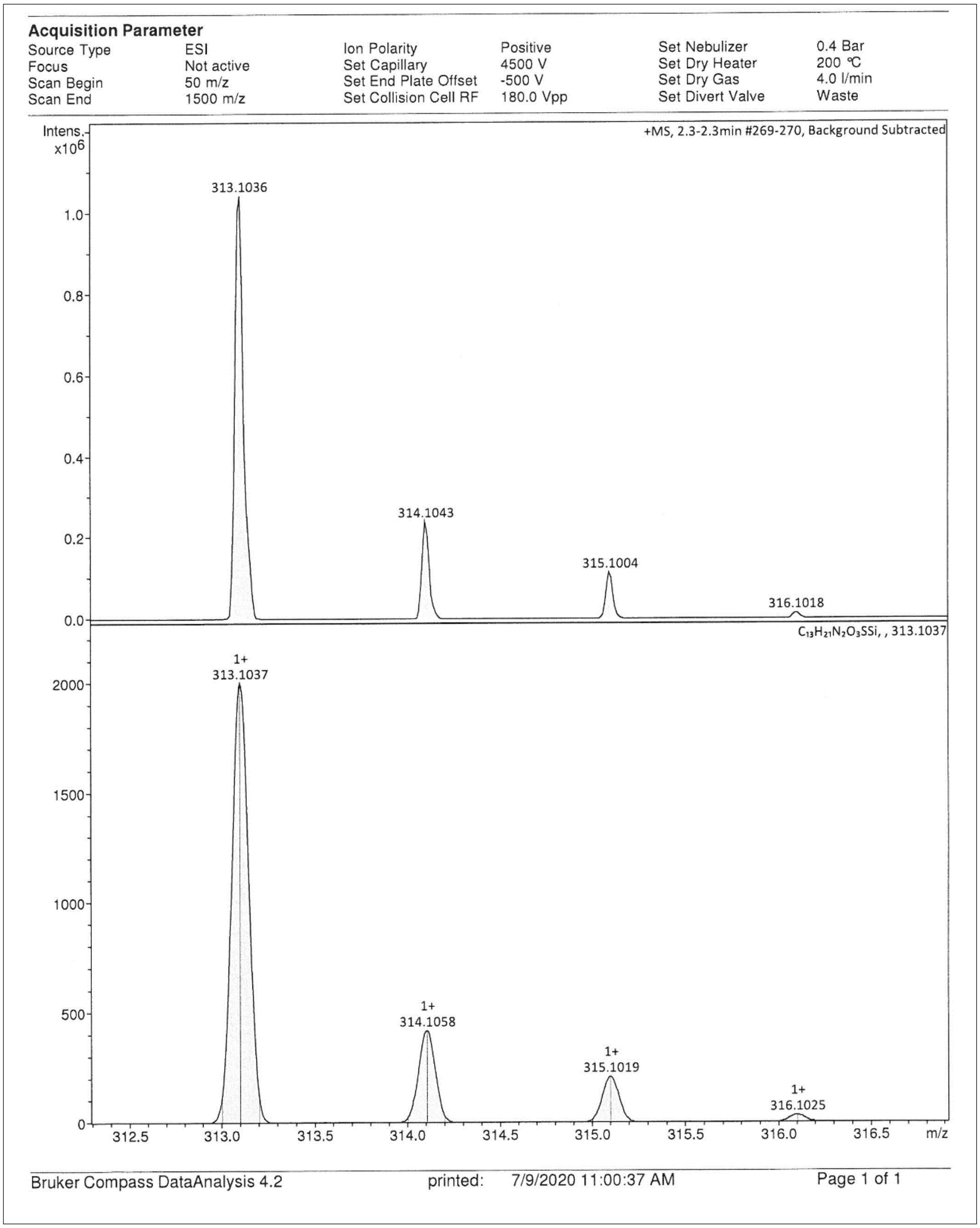

Figure S54. High-resolution mass spectrum of **L2**. Found (top) and calculated (bottom) isotope distribution of the molecular ion peak.

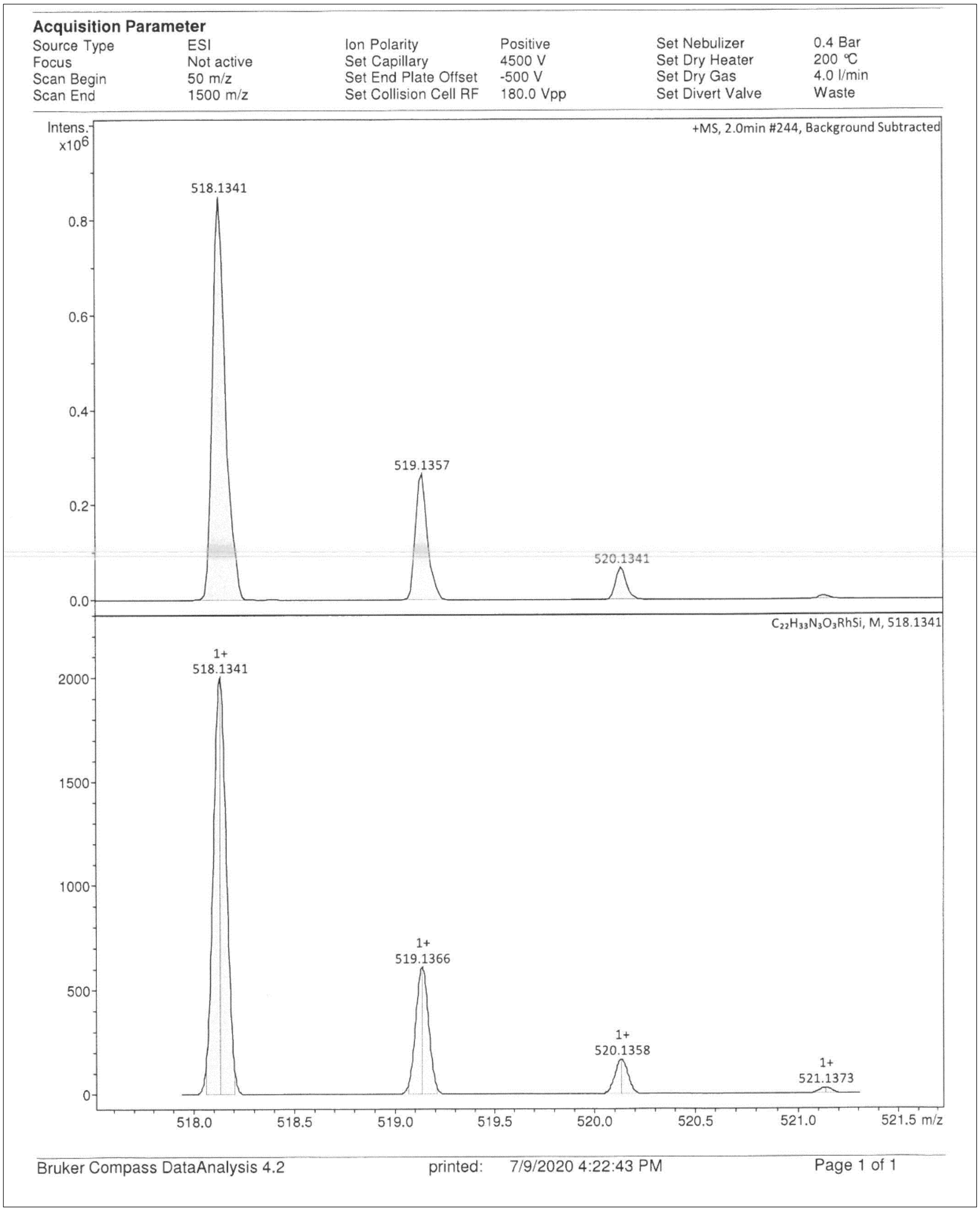

Figure S55. High-resolution mass spectrum of **Rh1**. Found (top) and calculated (bottom) isotope distribution of the molecular ion peak.

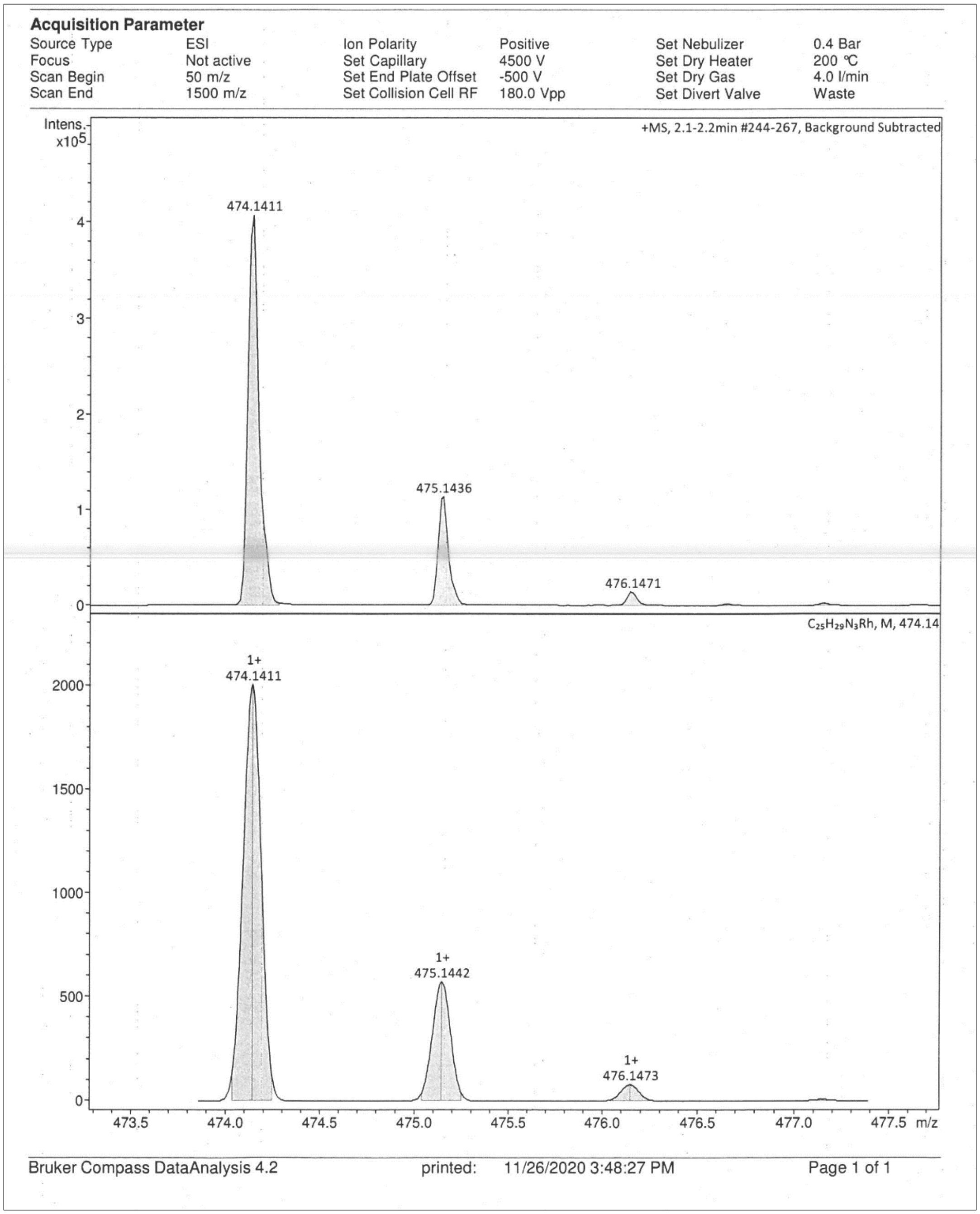

Figure S56. High-resolution mass spectrum of **Rh2**. Found (top) and calculated (bottom) isotope distribution of the molecular ion peak.

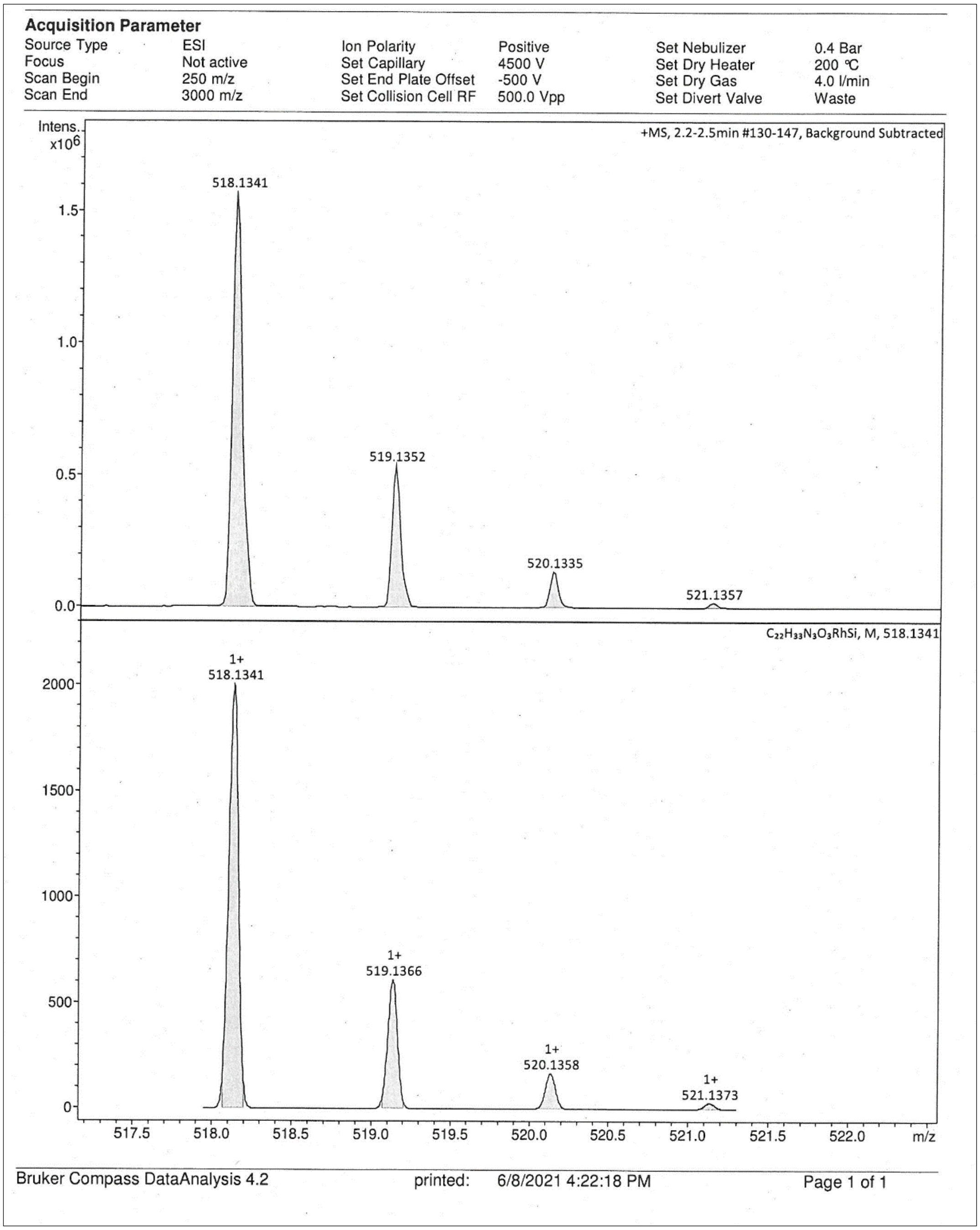

Figure S57. High-resolution mass spectrum of **Rh3**. Found (top) and calculated (bottom) isotope distribution of the molecular ion peak.

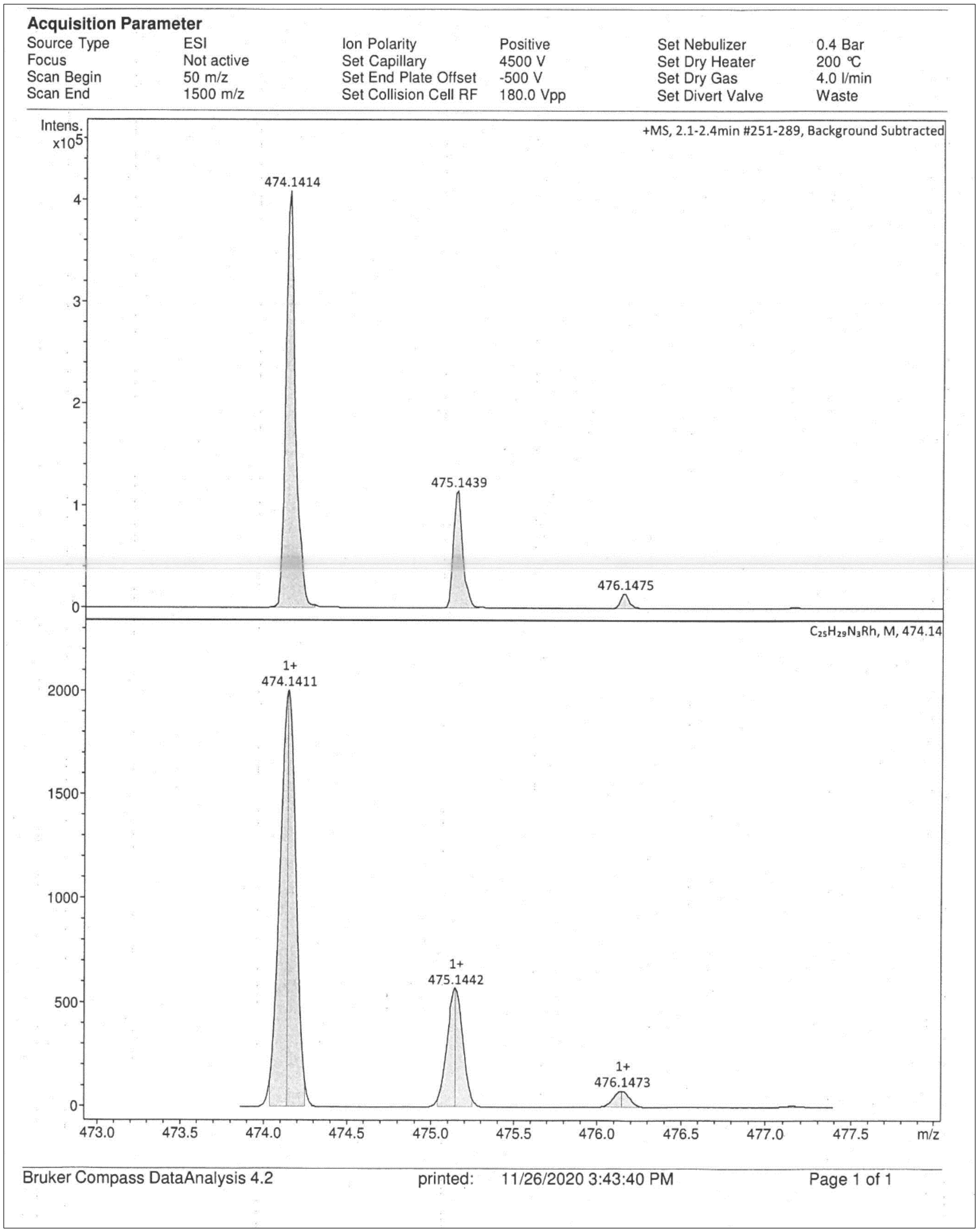

Figure S58. High-resolution mass spectrum of **Rh4**. Found (top) and calculated (bottom) isotope distribution of the molecular ion peak.

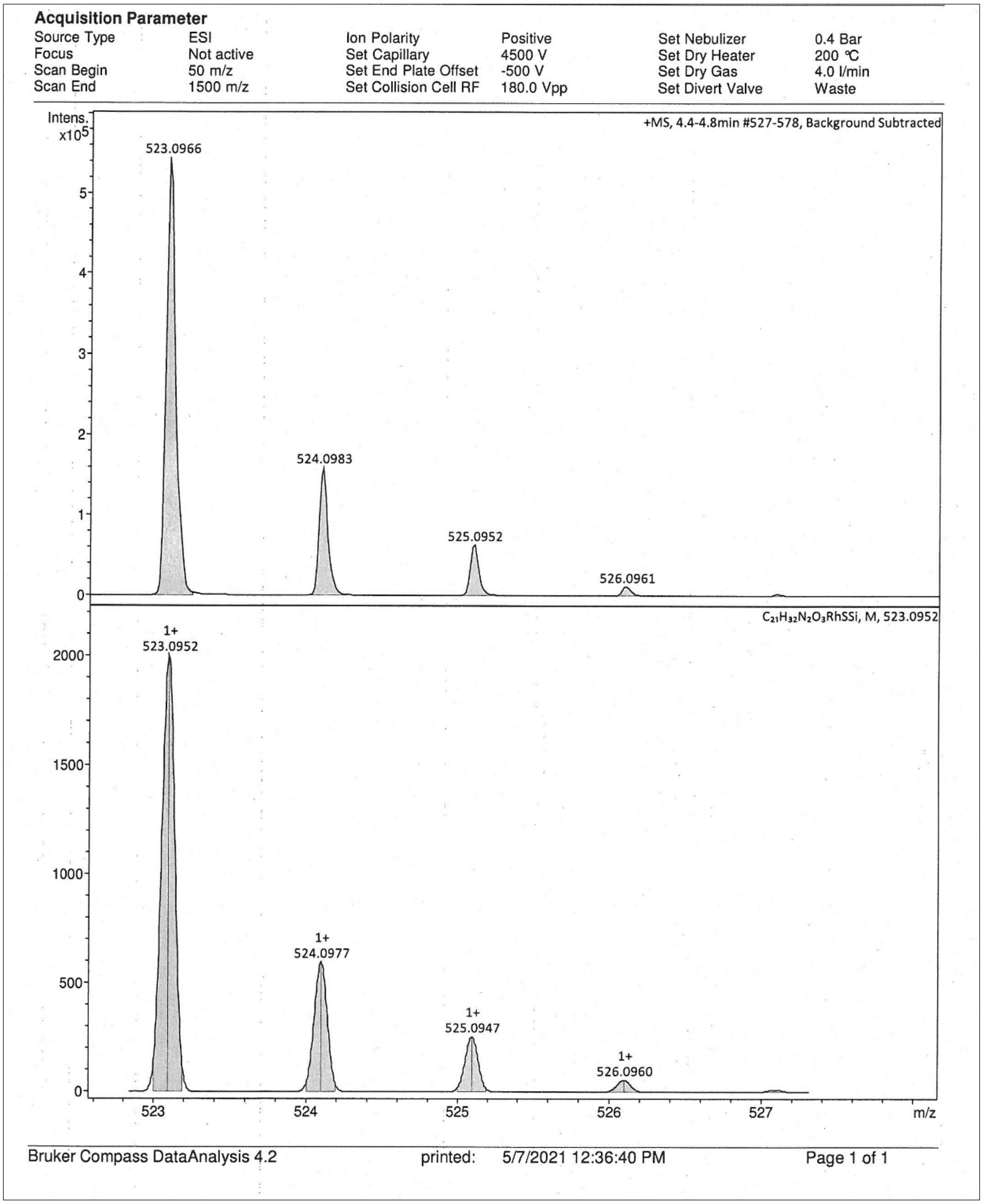

Figure S59. High-resolution mass spectrum of **Rh5**. Found (top) and calculated (bottom) isotope distribution of the molecular ion peak.

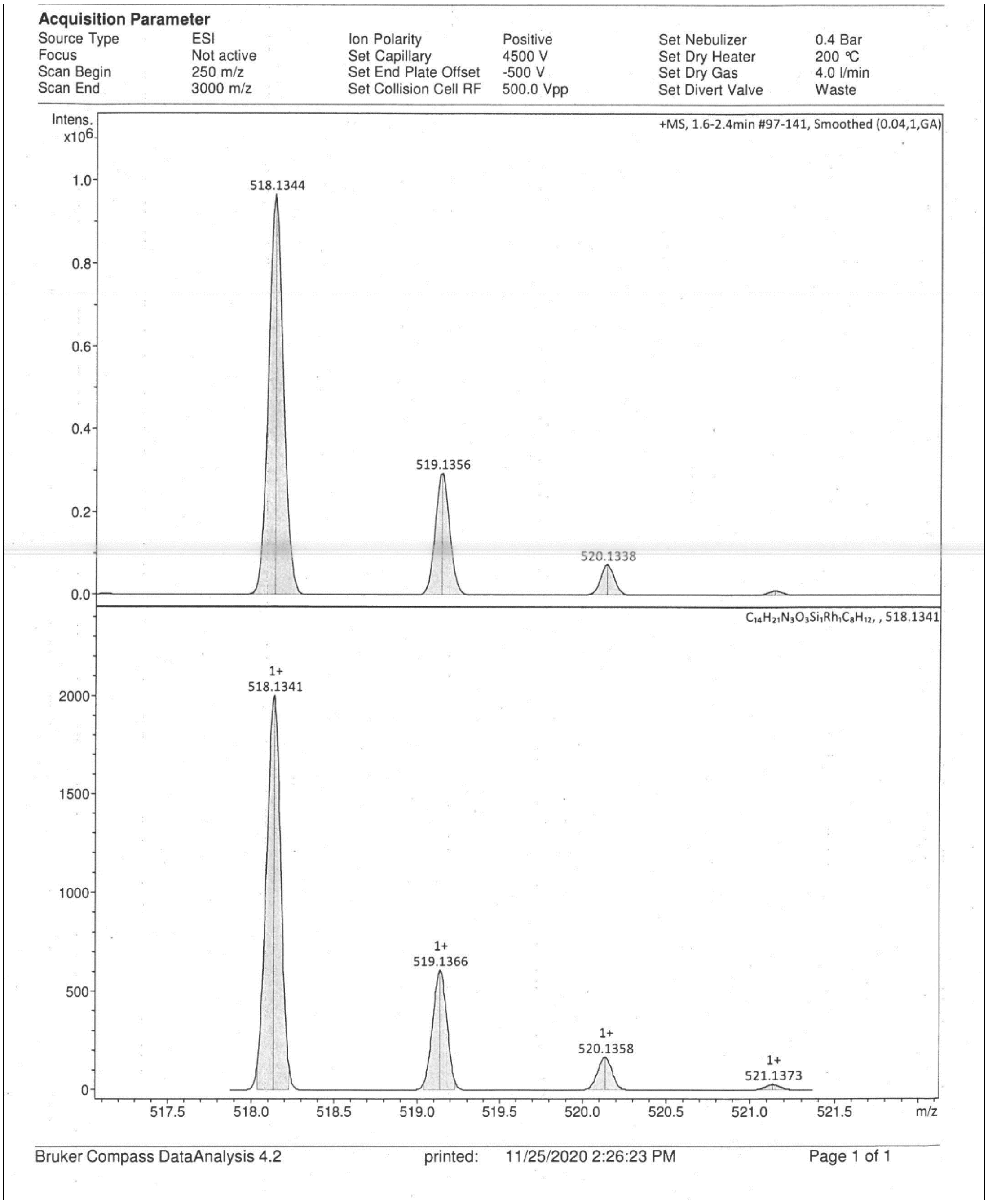

Figure S60. High-resolution mass spectrum of **Rh6**. Found (top) and calculated (bottom) isotope distribution of the molecular ion peak.

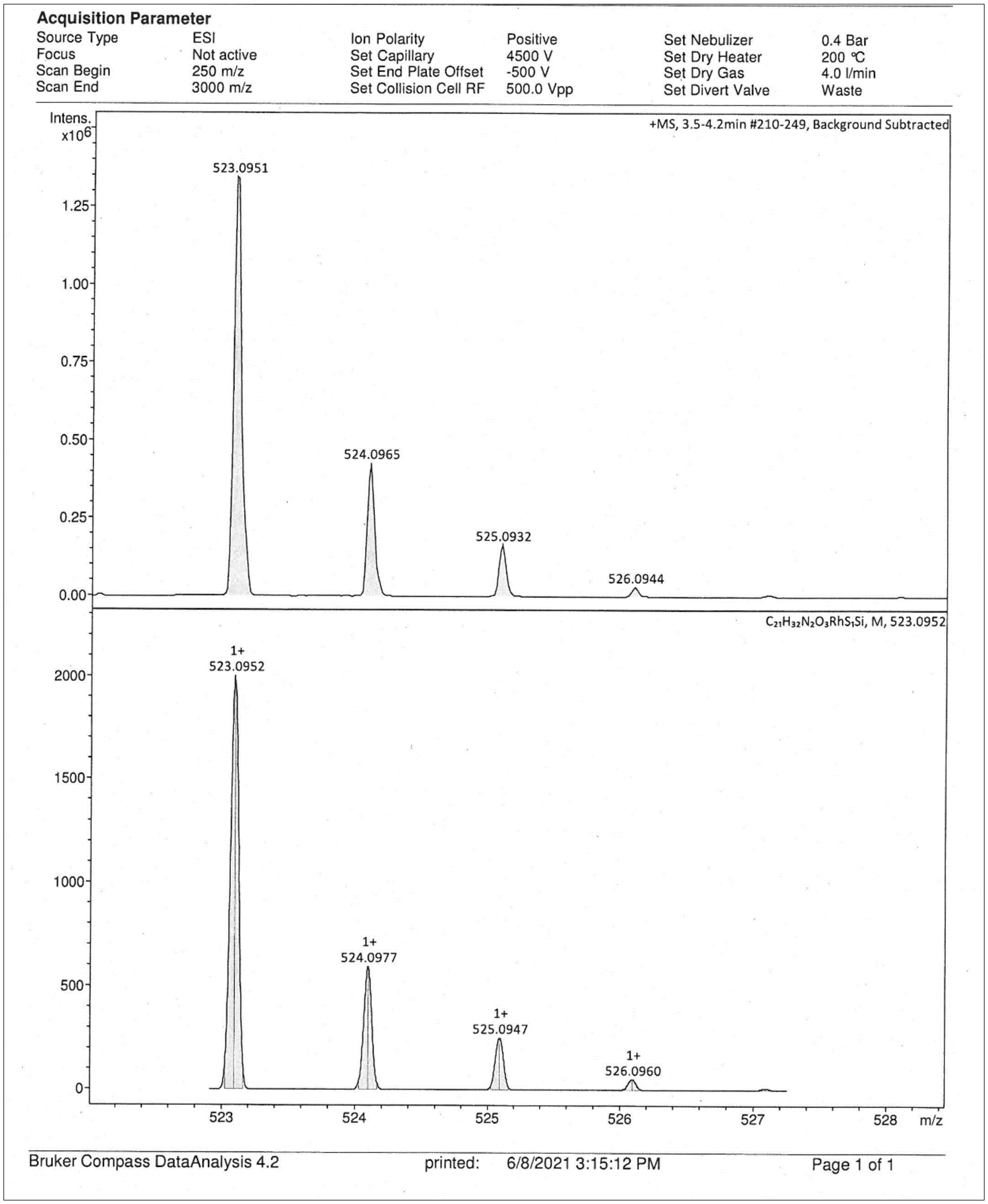

Figure S61. High-resolution mass spectrum of **Rh7**. Found (top) and calculated (bottom) isotope distribution of the molecular ion peak.

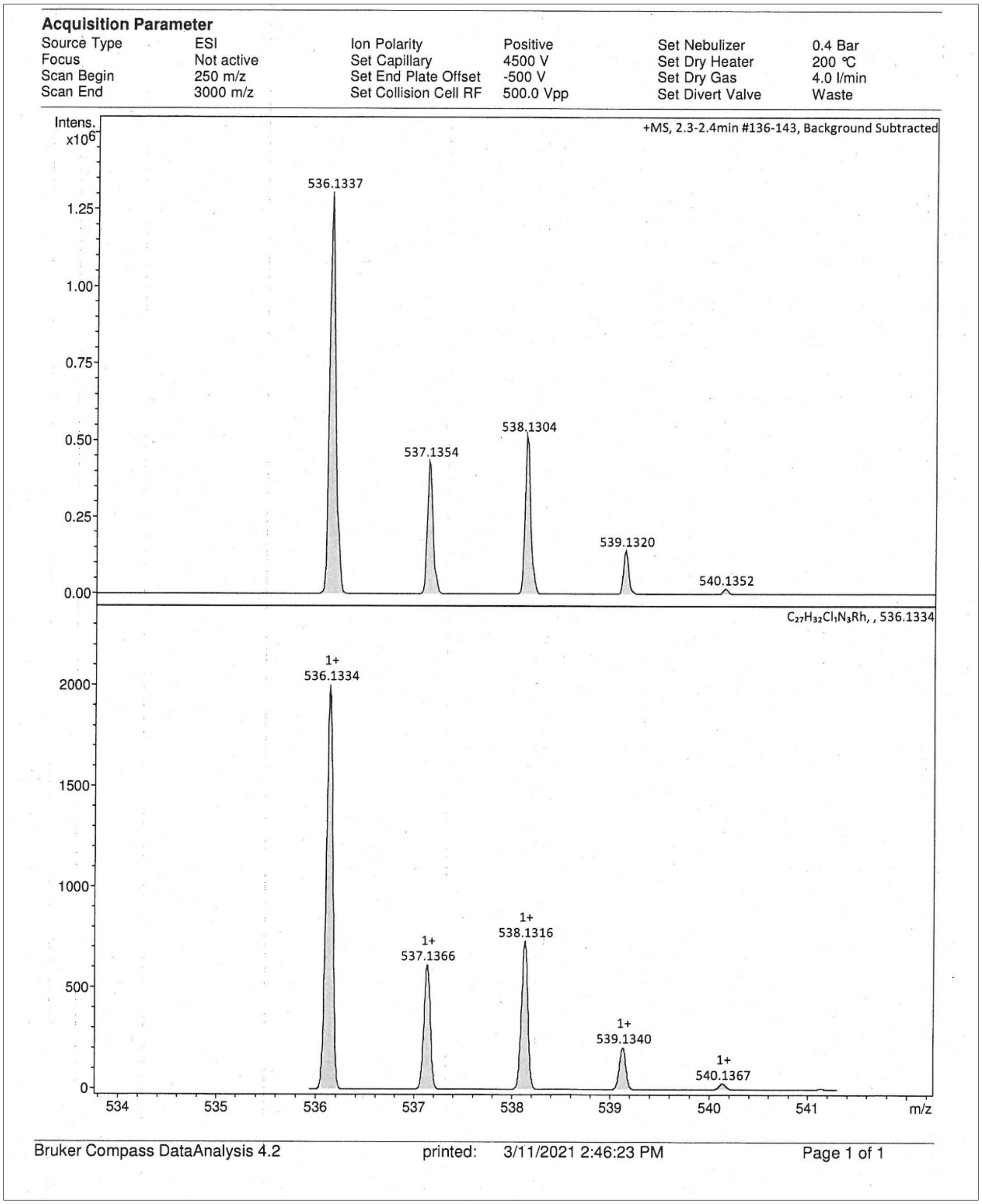

Figure S62. High-resolution mass spectrum of **Rh9**. Found (top) and calculated (bottom) isotope distribution of the molecular ion peak.

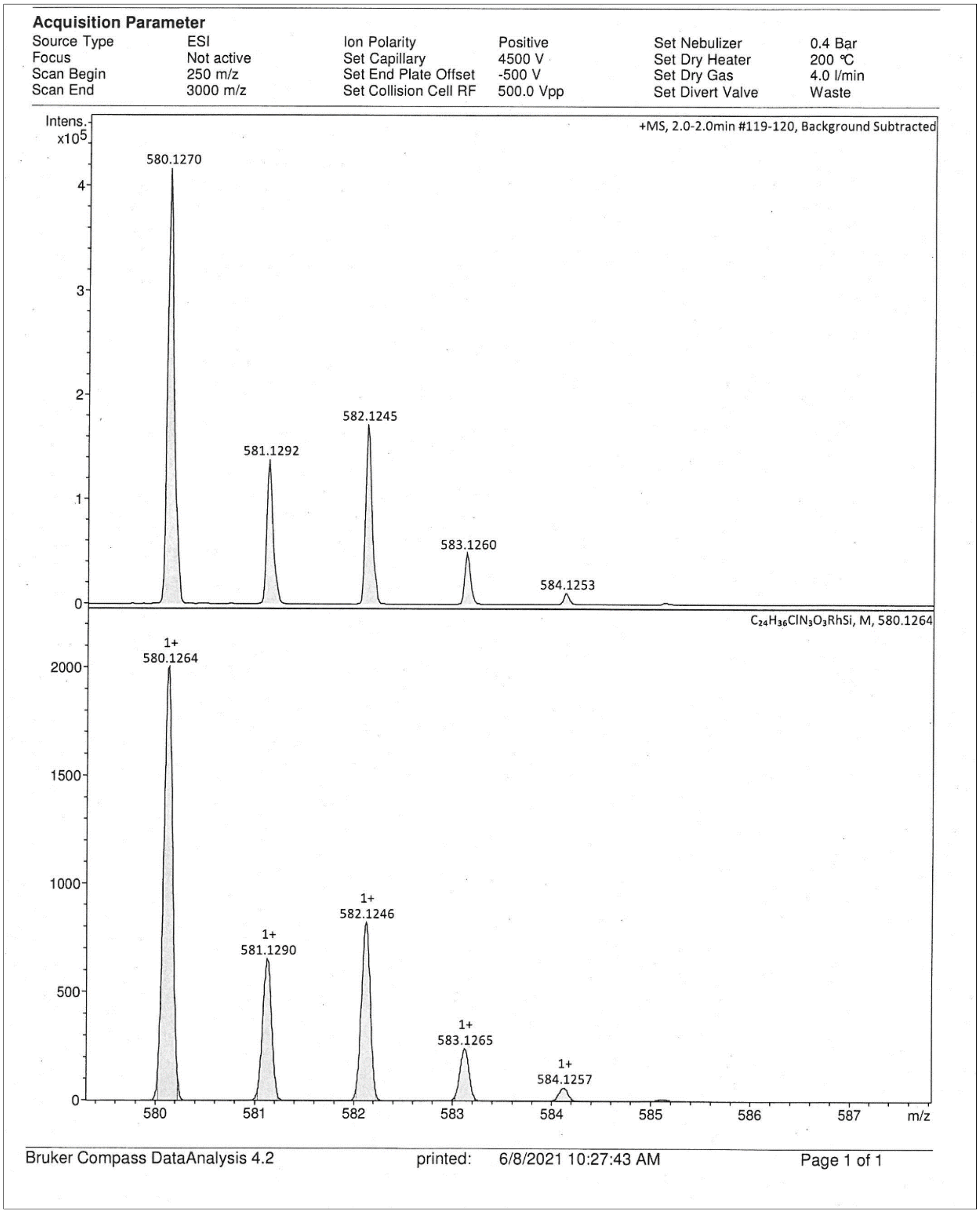

Figure S63. High-resolution mass spectrum of **Rh10**. Found (top) and calculated (bottom) isotope distribution of the molecular ion peak.

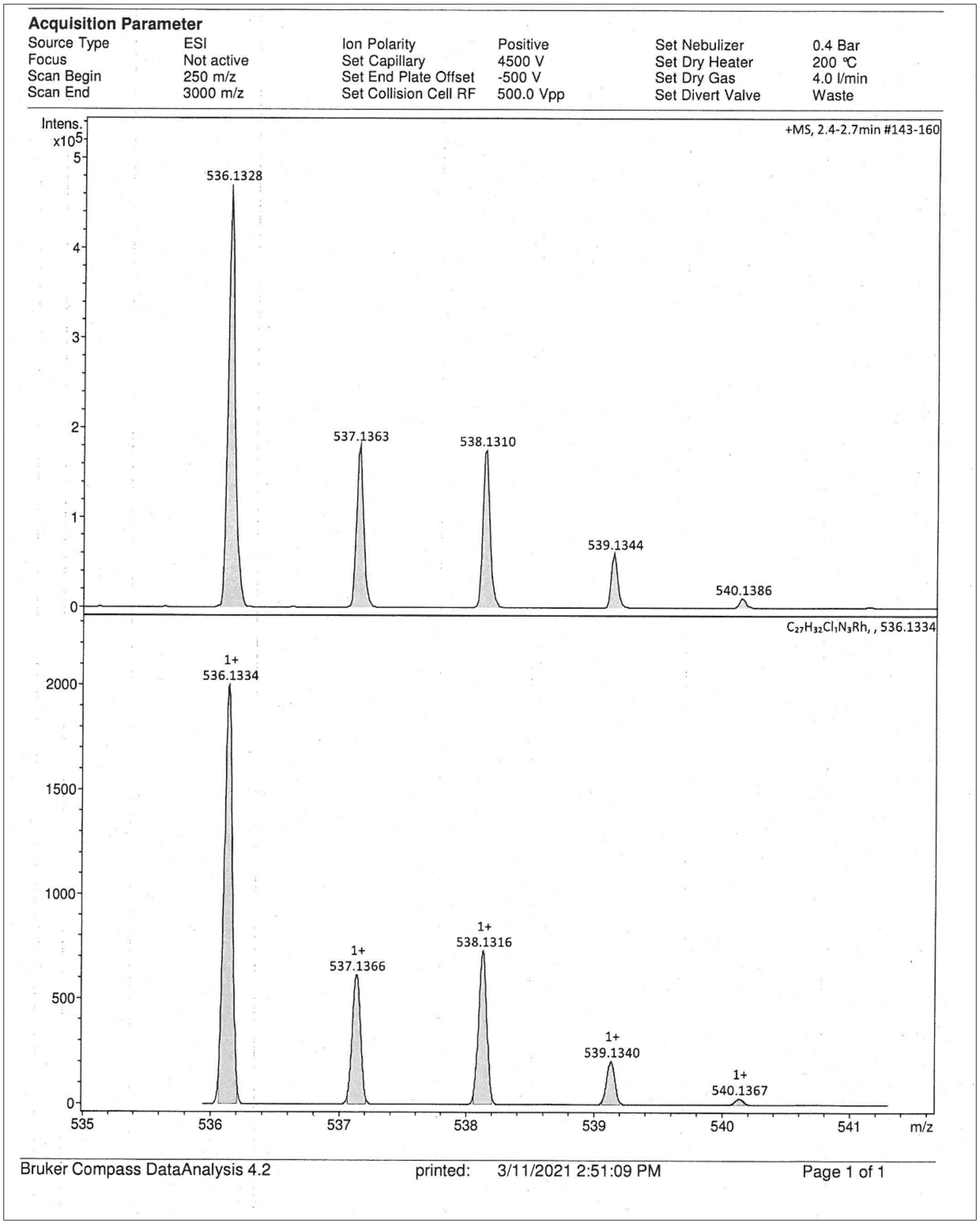

Figure S64. High-resolution mass spectrum of **Rh11**. Found (top) and calculated (bottom) isotope distribution of the molecular ion peak.

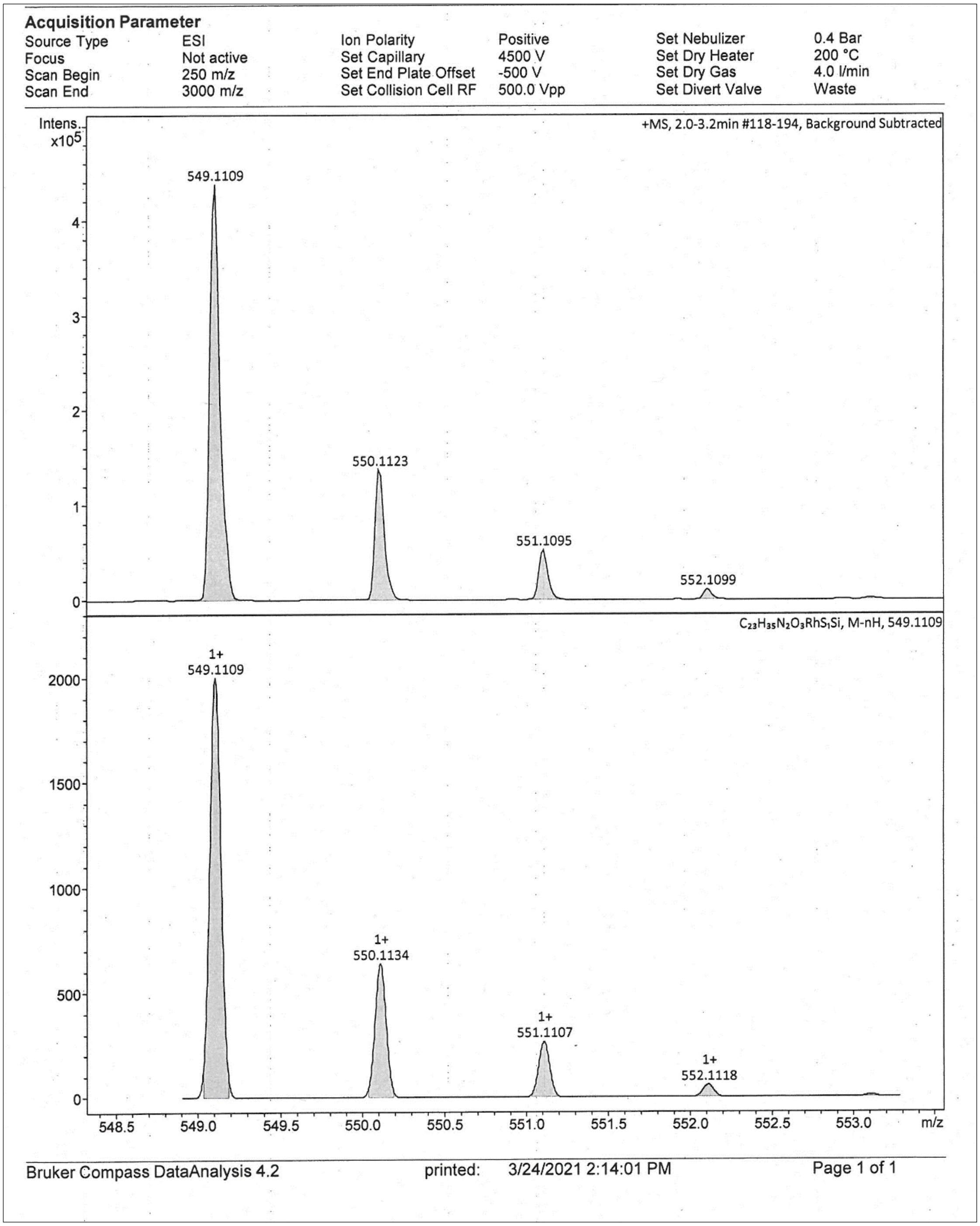

Figure S65. High-resolution mass spectrum of **Rh12**. Found (top) and calculated (bottom) isotope distribution of the molecular ion peak.

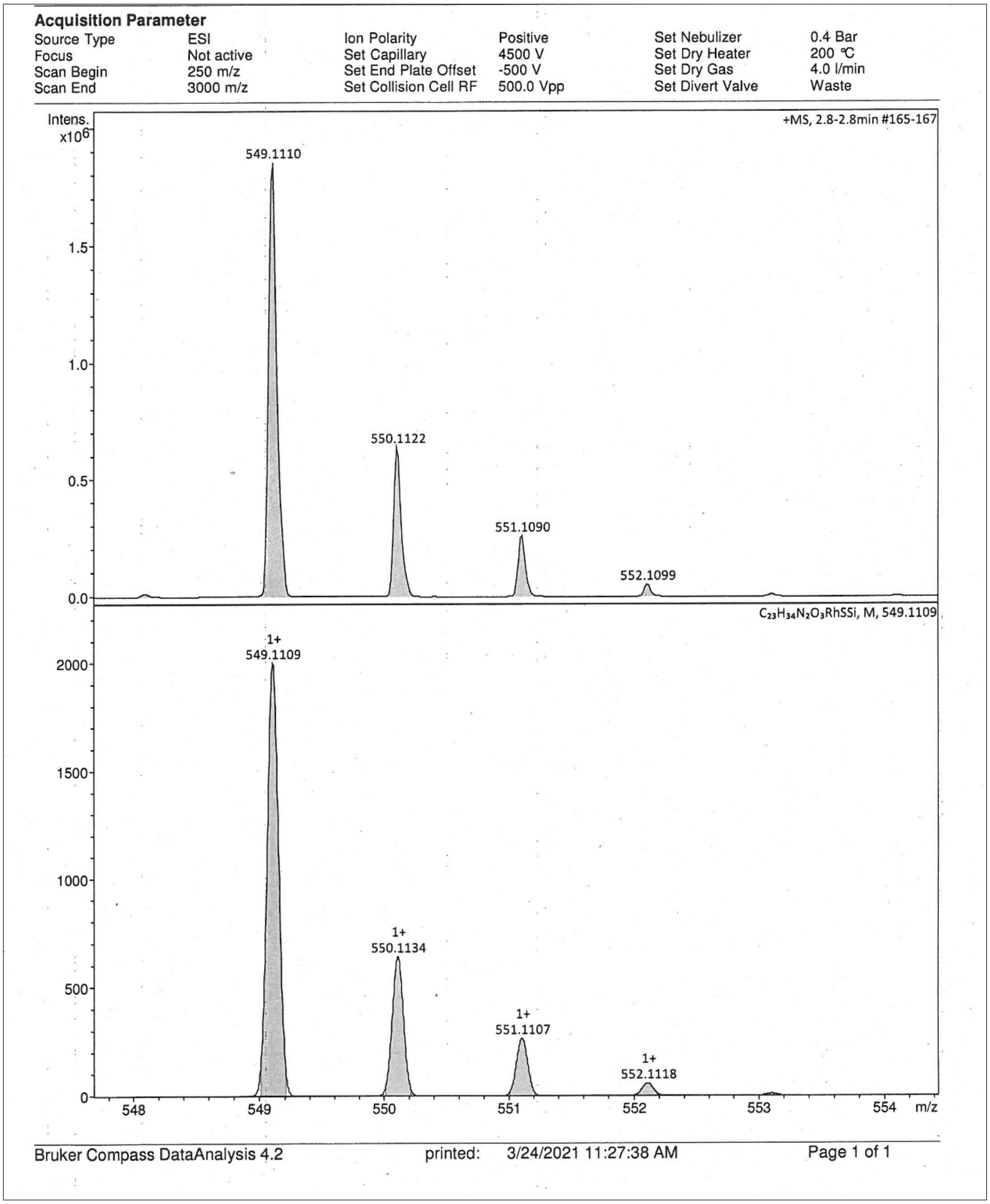

Figure S66. High-resolution mass spectrum of **Rh13**. Found (top) and calculated (bottom) isotope distribution of the molecular ion peak.

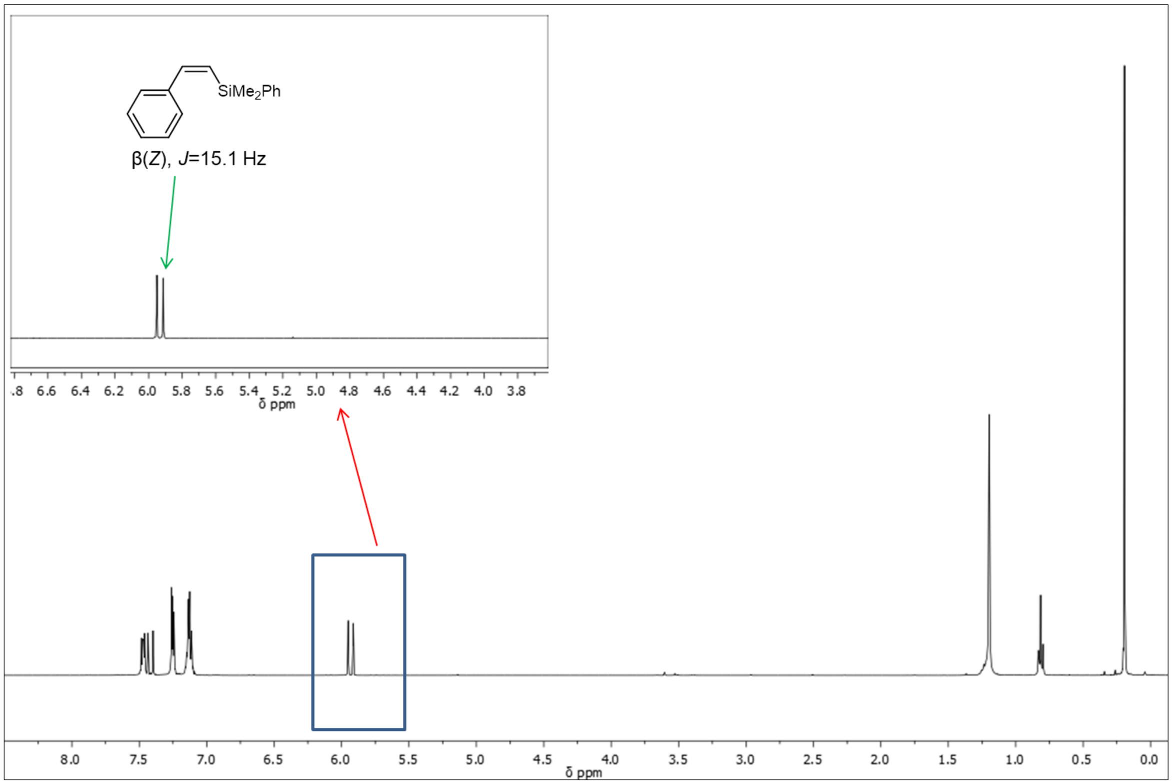

Figure S67. ^{1}H NMR of the hydrosilylation of phenylacetylene catalyzed by **Rh12**.

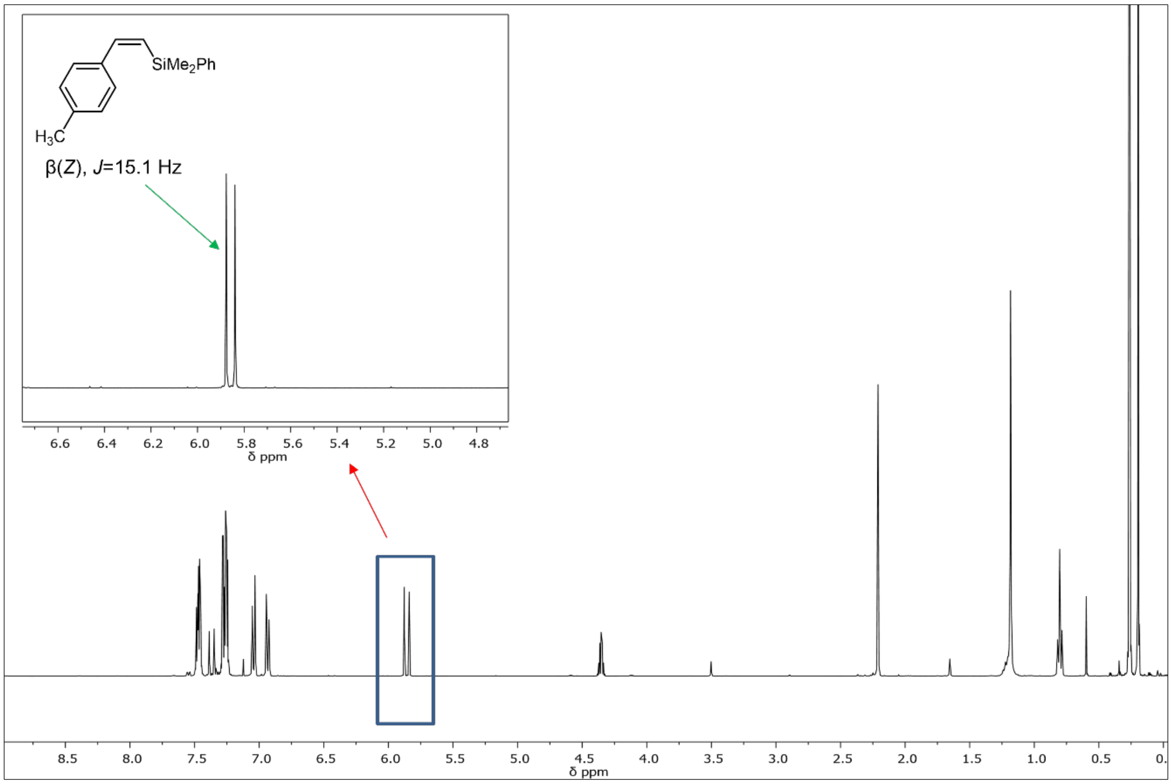

Figure S68. ^{1}H NMR of the hydrosilylation of 4-ethynyltoluene catalyzed by **Rh12**.

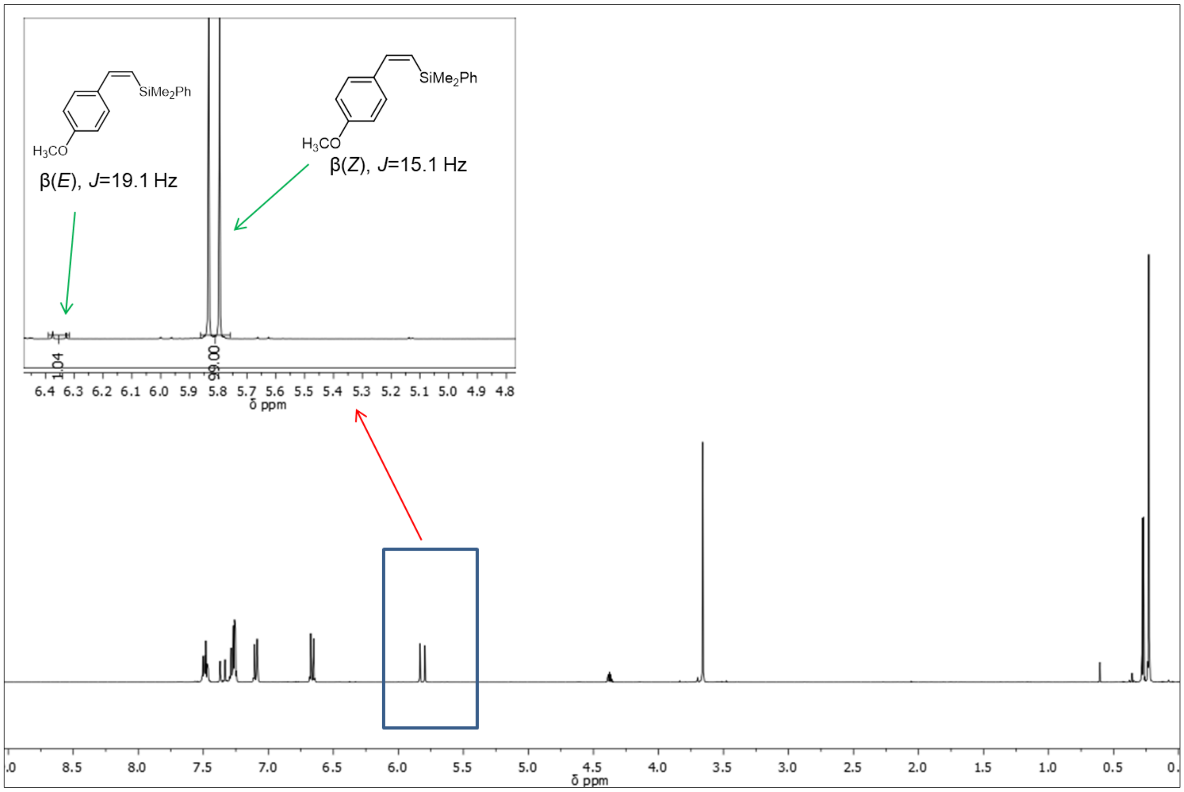

Figure S69. ^{1}H NMR of the hydrosilylation of 4-ethynylanisole catalyzed by **Rh12**.

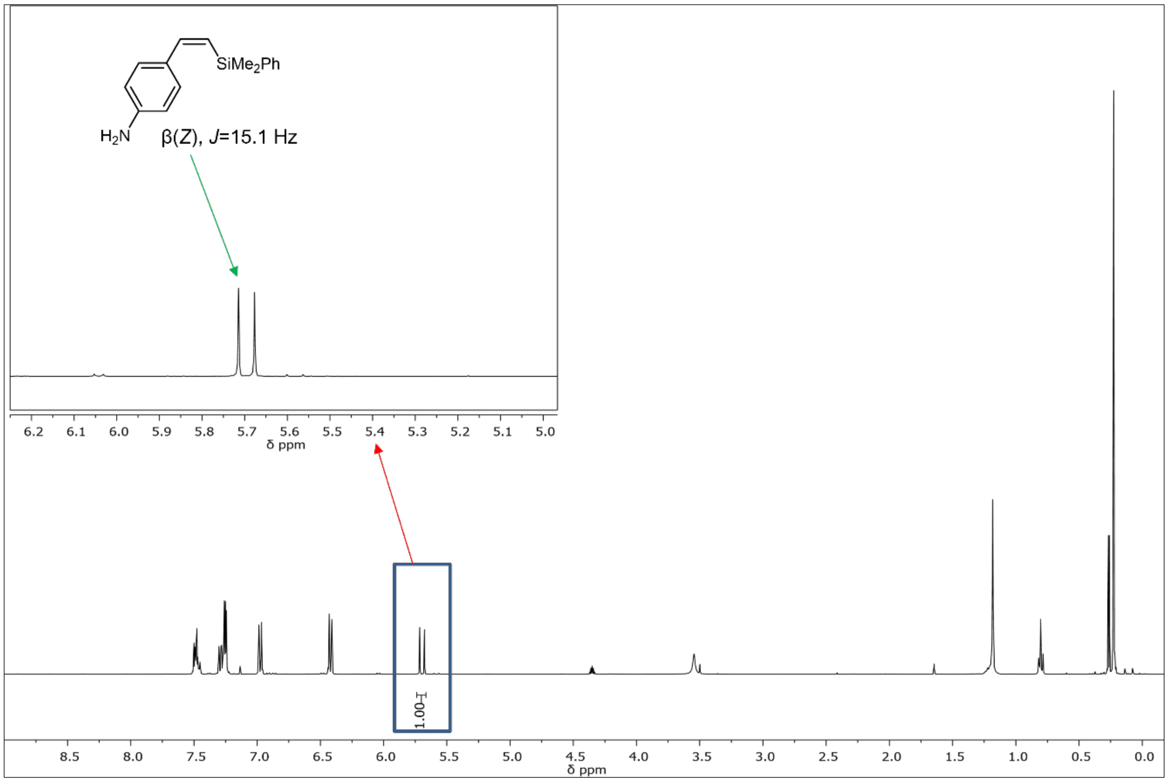

Figure S70. ^{1}H NMR of the hydrosilylation of 4-ethynylaniline catalyzed by **Rh12@SBA-15**.

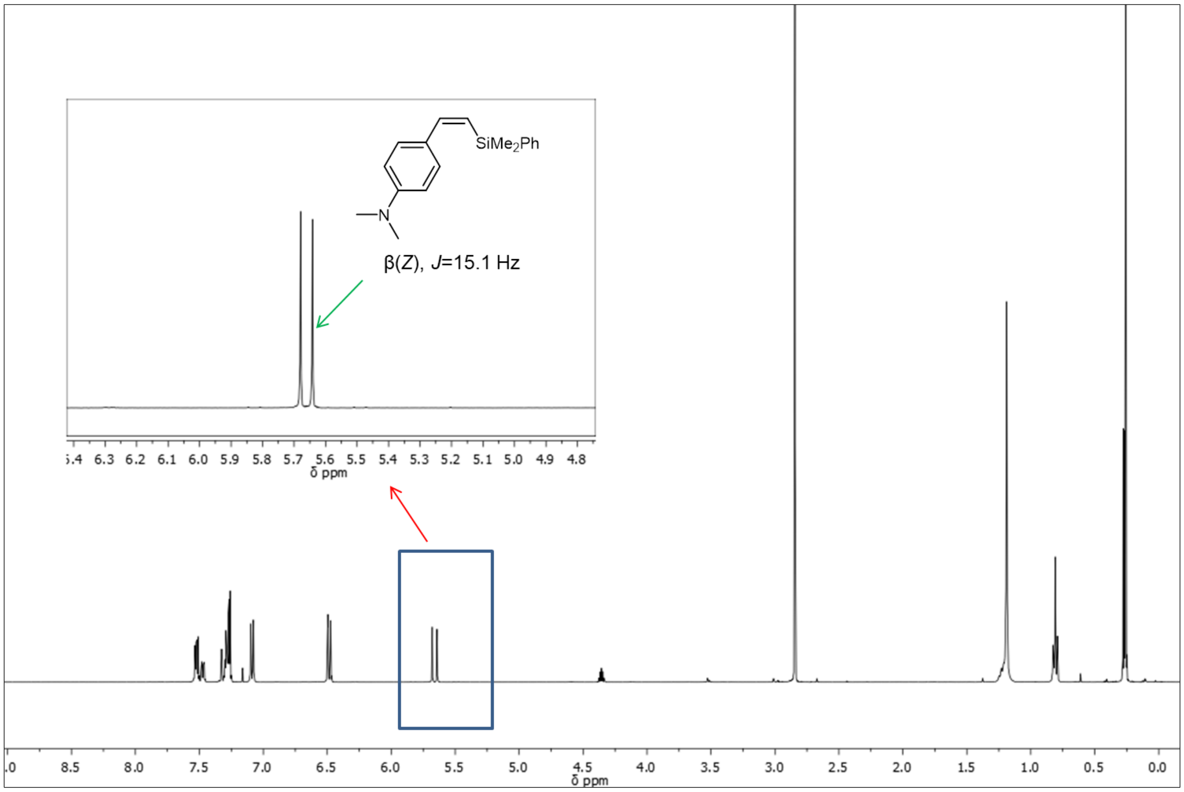

Figure S71. ^{1}H NMR of the hydrosilylation of 4-ethynyl-*N*,*N*-dimethylaniline catalyzed by **Rh12**.

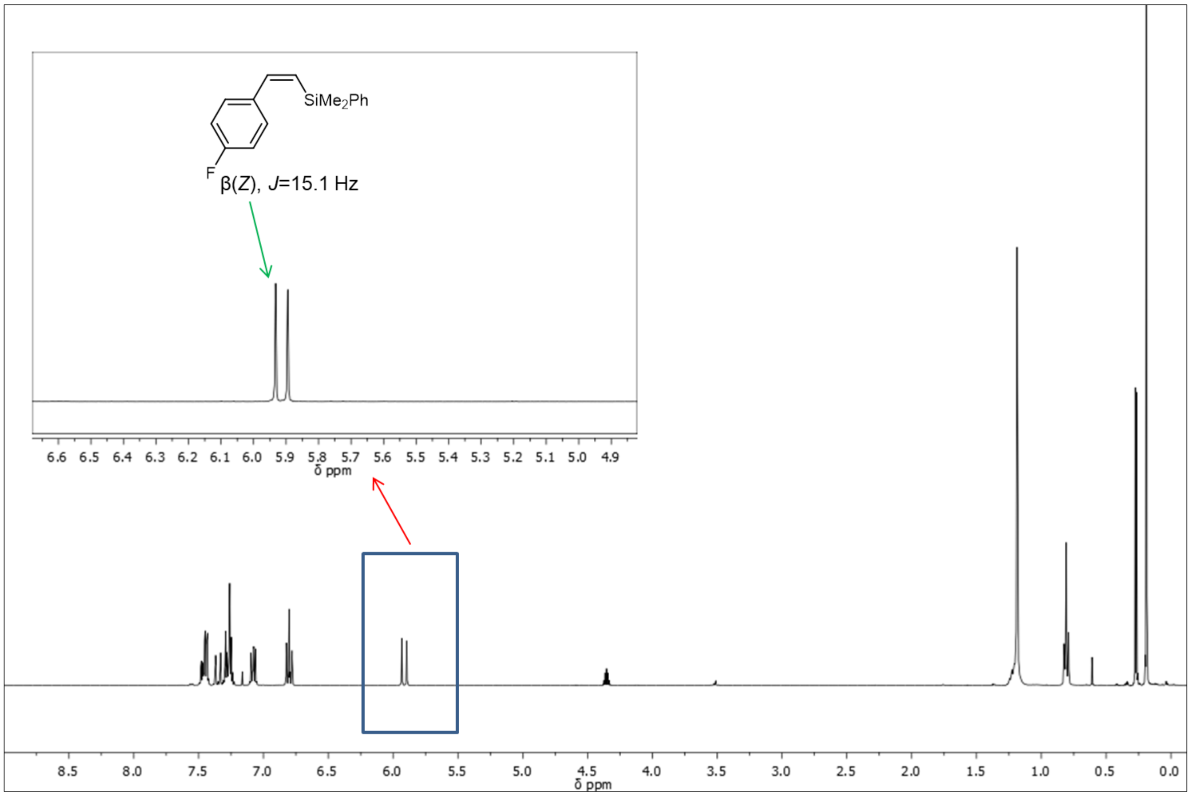

Figure S72. ^{1}H NMR of the hydrosilylation of 1-ethynyl-4-fluorobenzene catalyzed by **Rh12**.

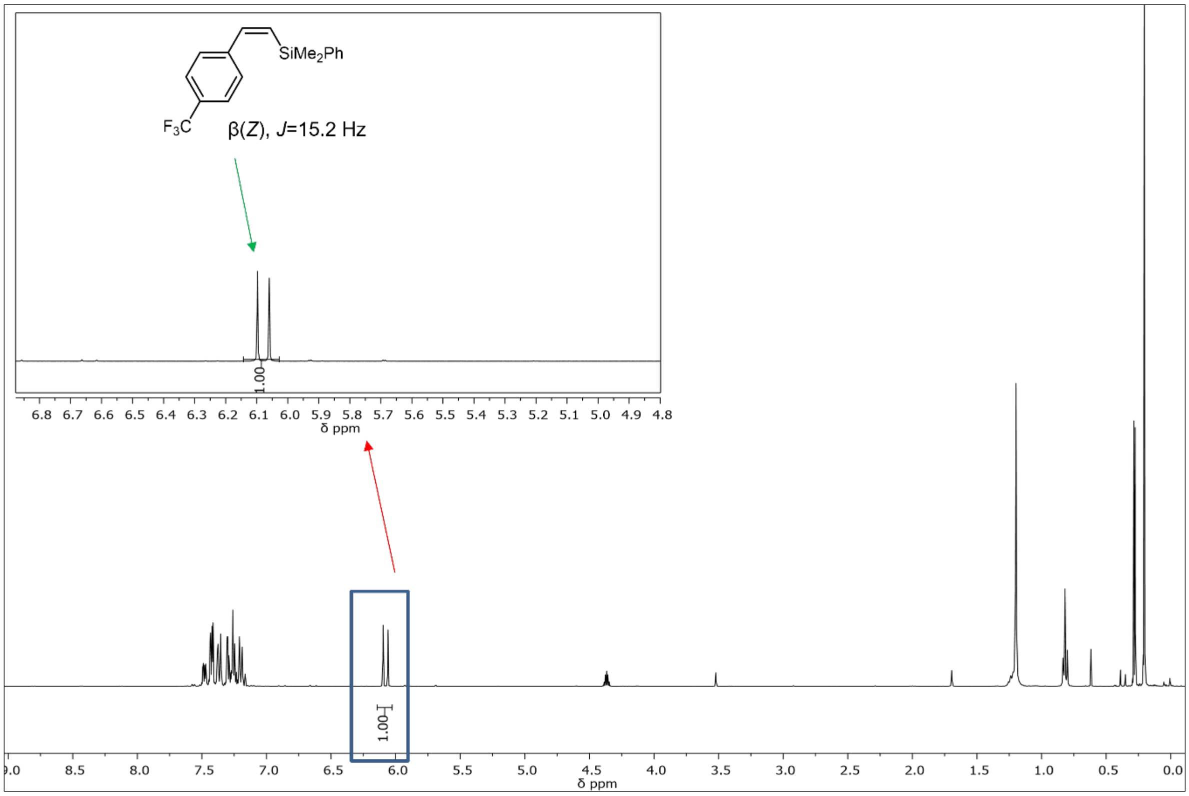

Figure S73. ^{1}H NMR of the hydrosilylation of 1-ethynyl-4-(trifluoromethyl)benzene catalyzed by **Rh12**.

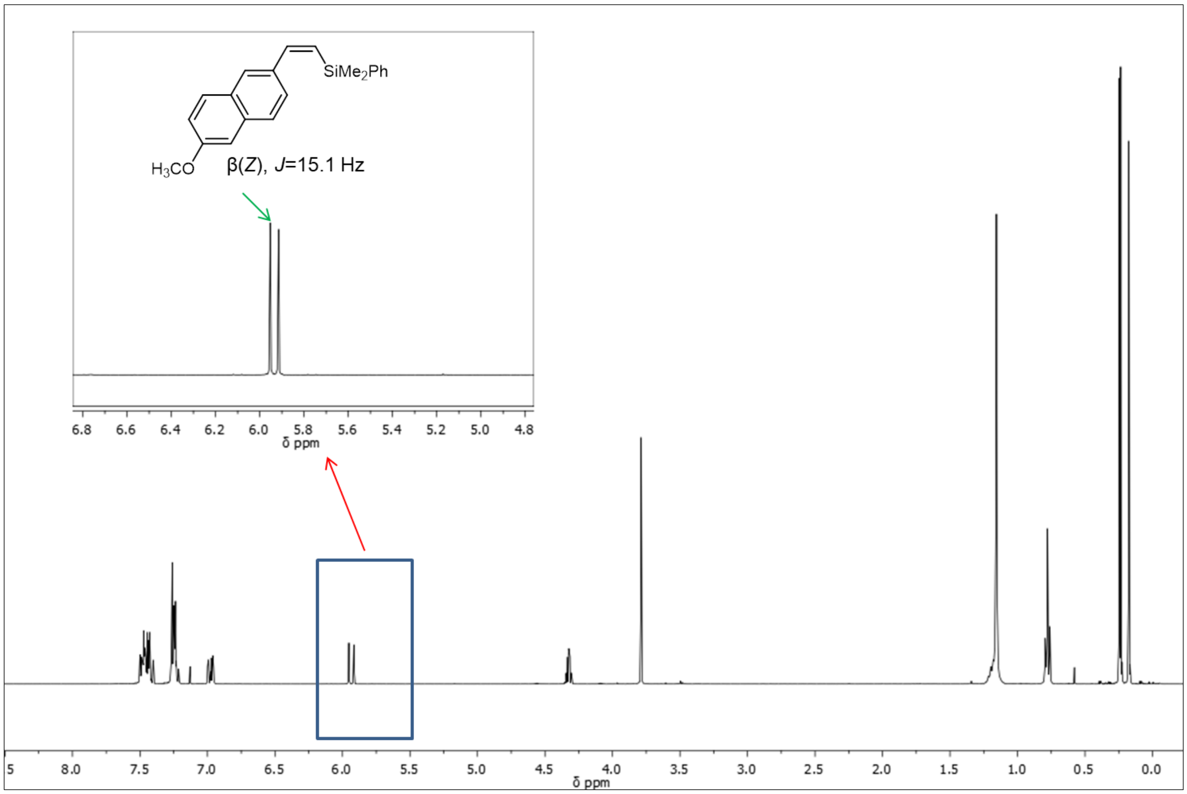

Figure S74. ^{1}H NMR of the hydrosilylation of 2-ethynyl-6-methoxynaphthalene catalyzed by **Rh12**.

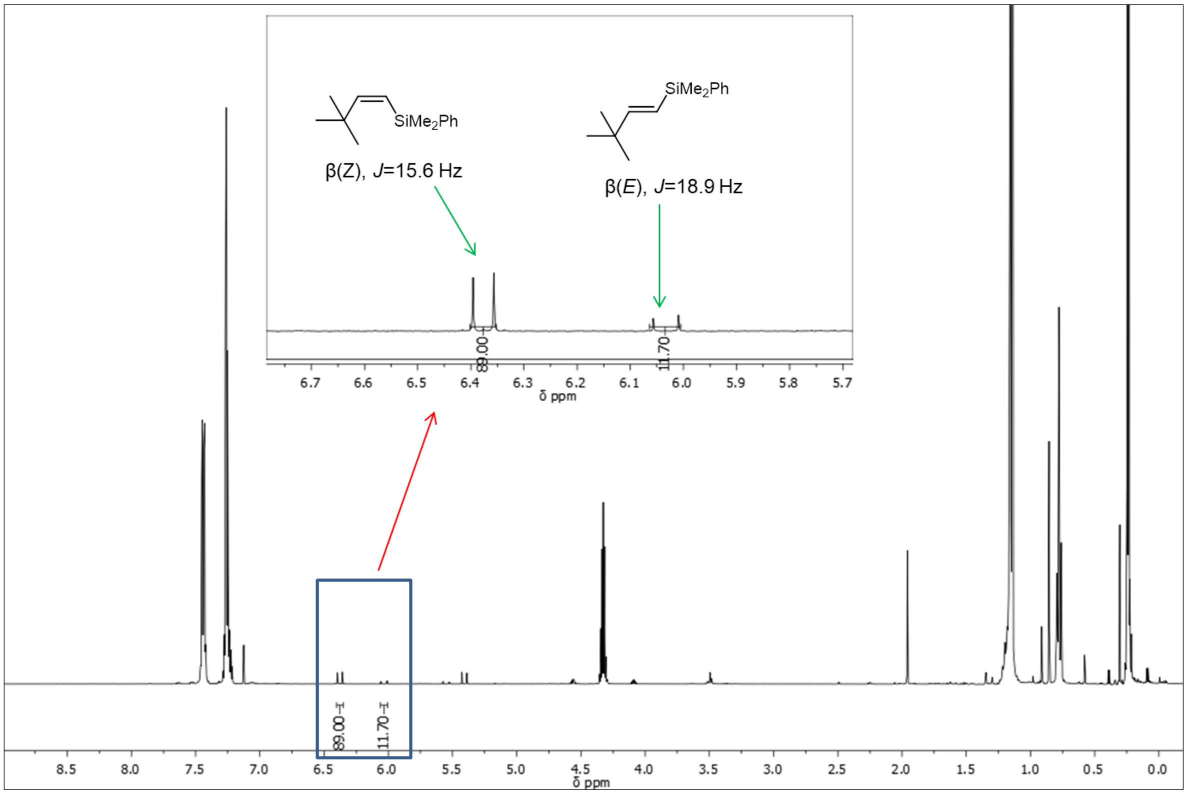

Figure S75. ^{1}H NMR of the hydrosilylation of 3,3-dimethyl-1-butyne catalyzed by **Rh12**.

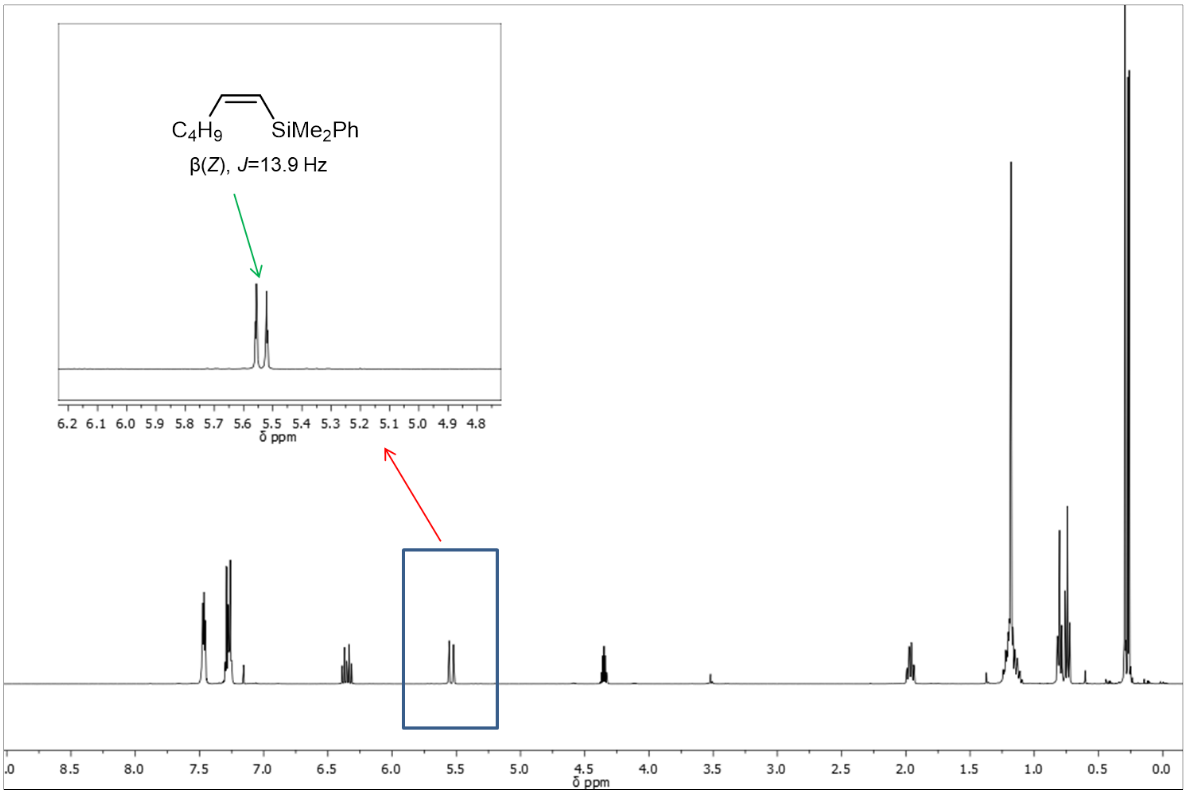

Figure S76. ^{1}H NMR of the hydrosilylation of 1-hexyne catalyzed by **Rh12**.

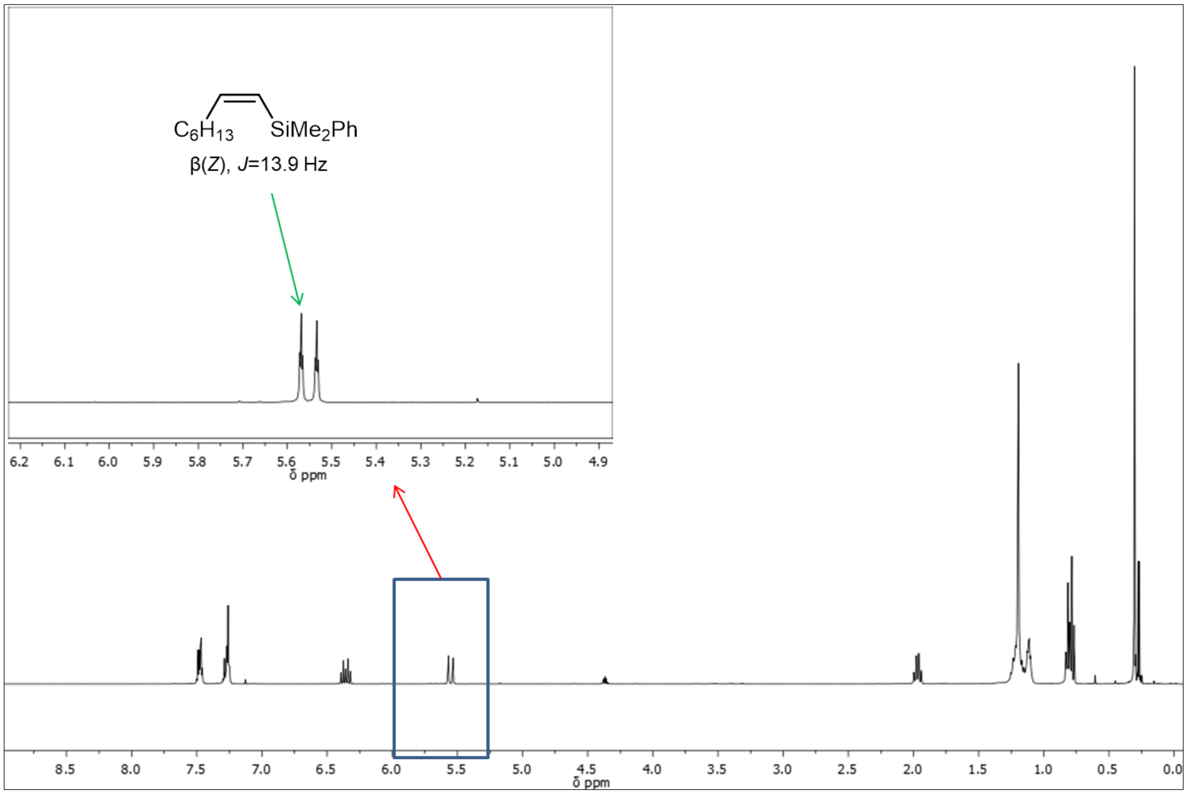

Figure S77. ^{1}H NMR of the hydrosilylation of 1-octyne catalyzed by **Rh12**.

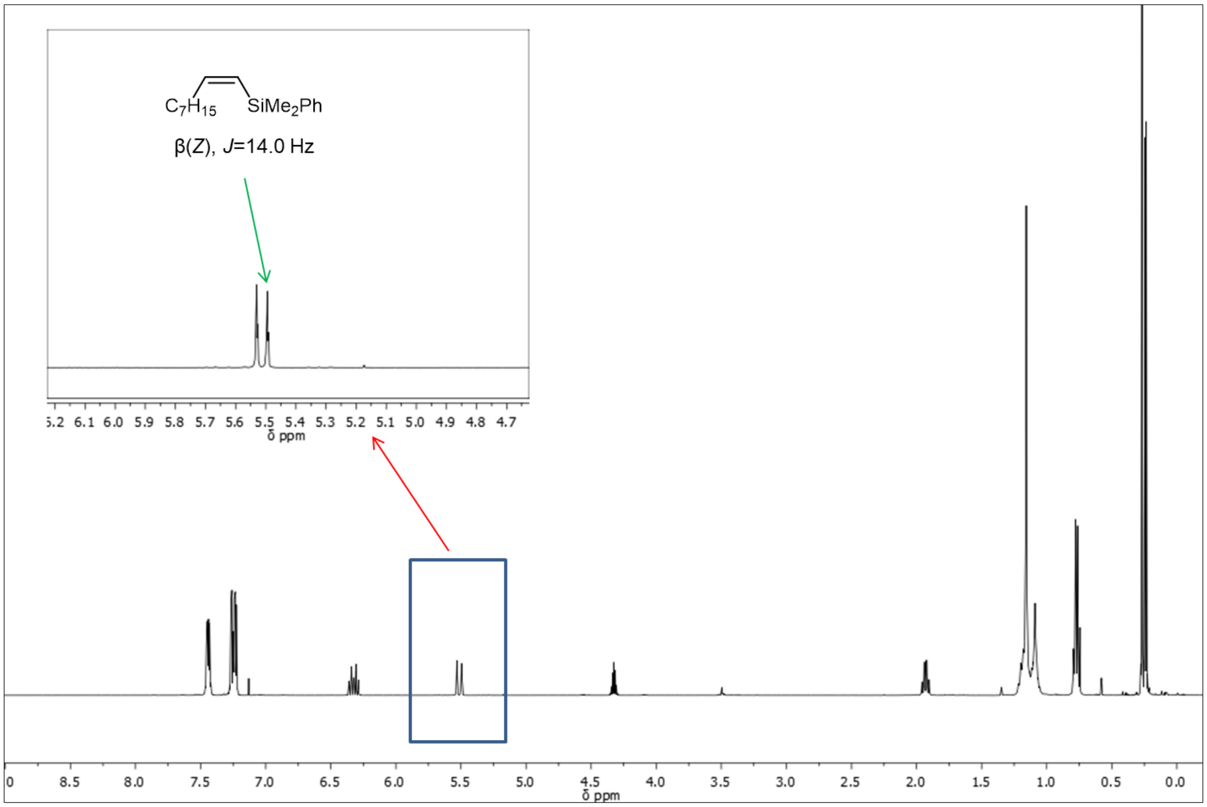

Figure S78. ^{1}H NMR of the hydrosilylation of 1-nonyne catalyzed by **Rh12**.

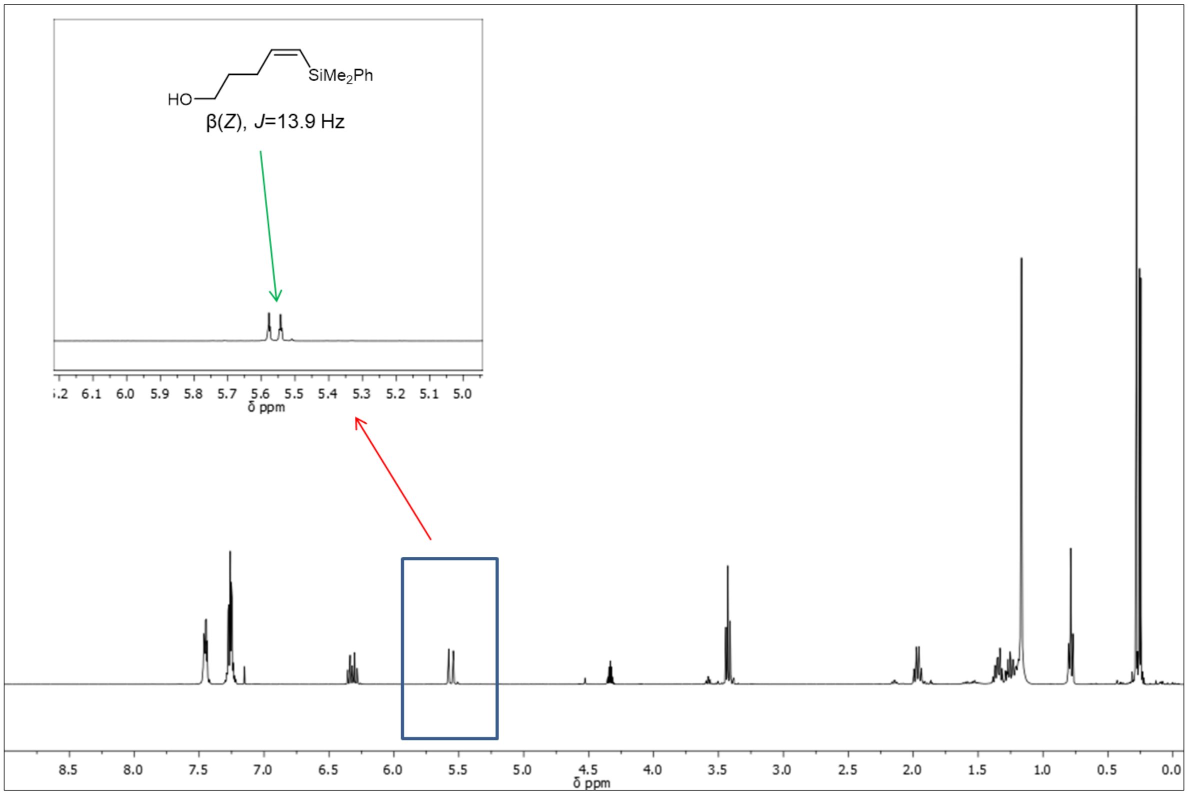

Figure S79. ^{1}H NMR of the hydrosilylation of 4-pentyn-1-ol catalyzed by **Rh12**.

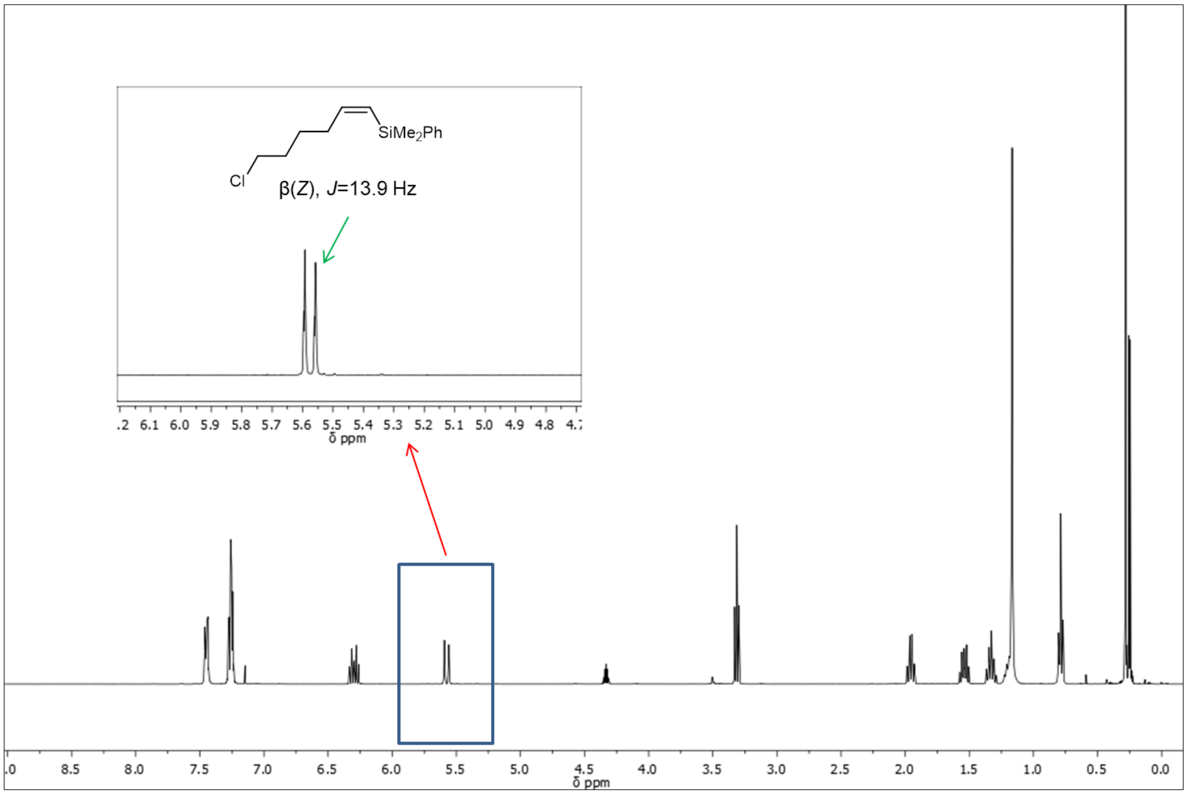

Figure S80. ^{1}H NMR of the hydrosilylation of 6-chloro-1-hexyne catalyzed by **Rh12**.

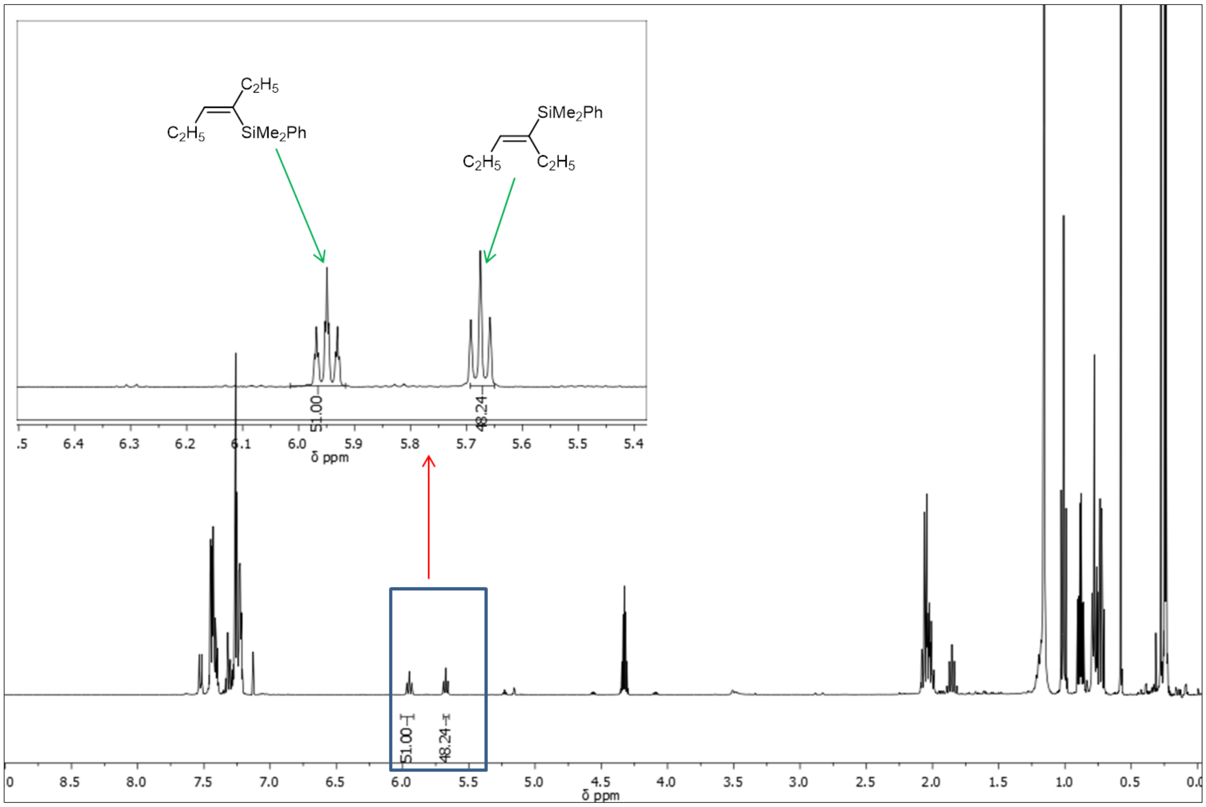

Figure S81. ^{1}H NMR of the hydrosilylation of 3-hexyne catalyzed by **Rh12**.

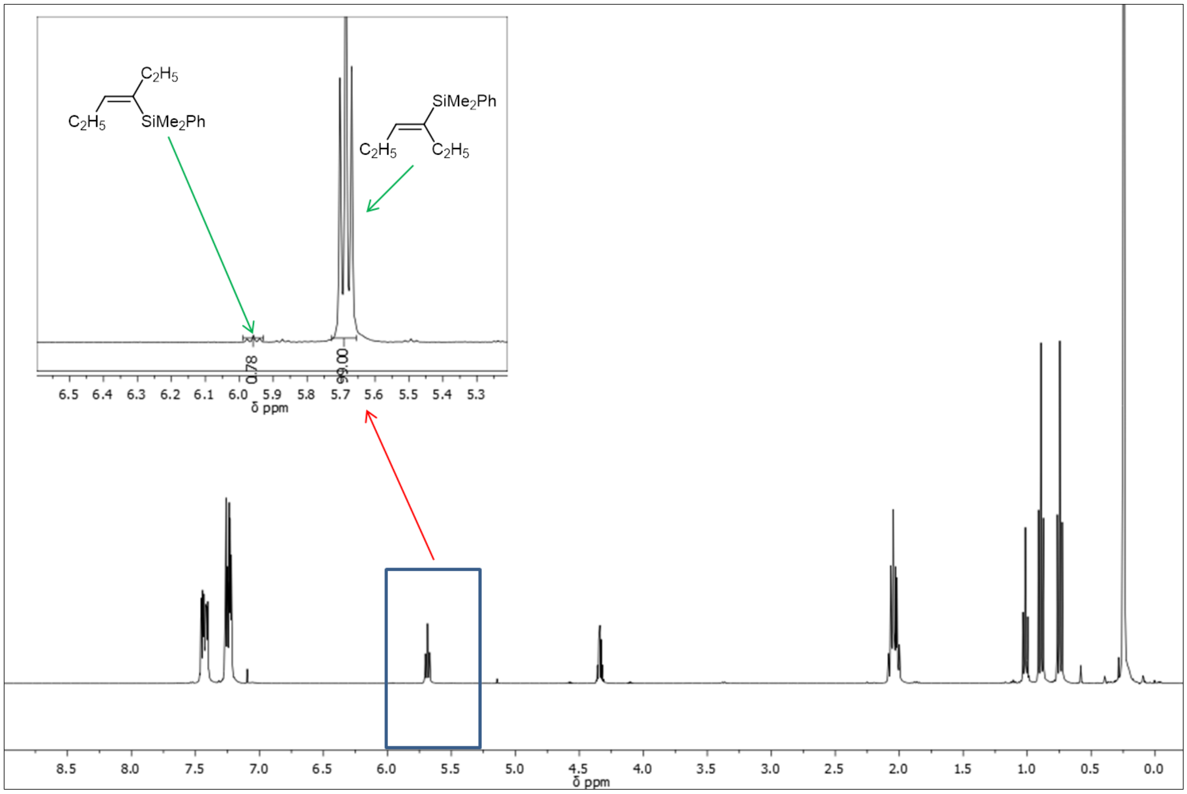

Figure S82. ^{1}H NMR of the hydrosilylation of 3-hexyne catalyzed by **Rh12@SBA-15**.

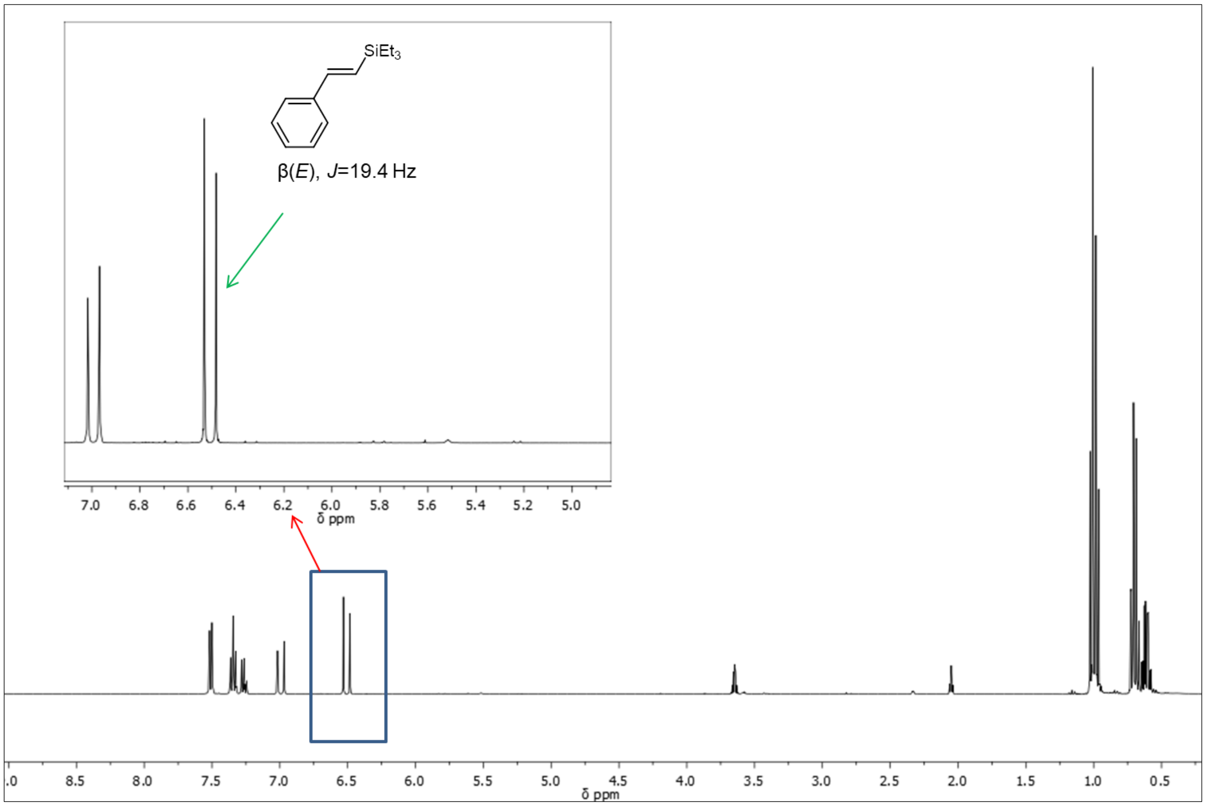

Figure S83. ^{1}H NMR of the hydrosilylation of phenylacetylene catalyzed by **Rh1**.

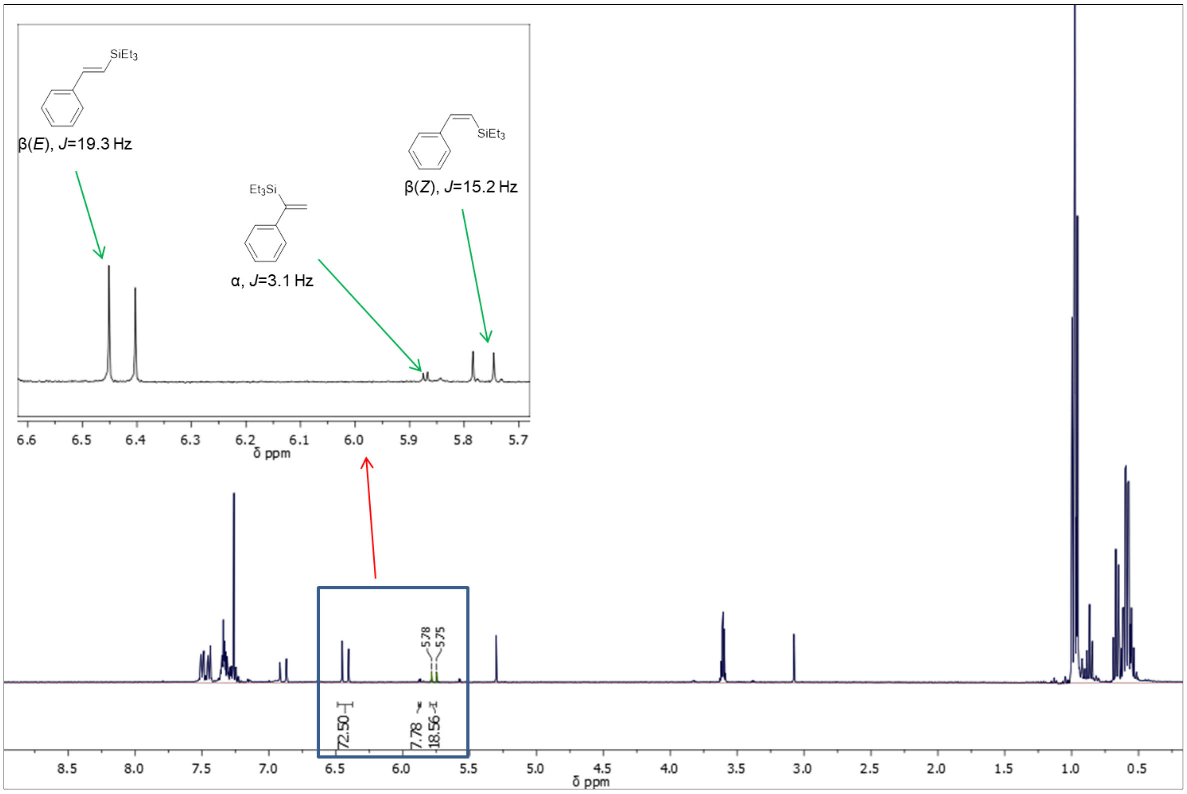

Figure S84. ^{1}H NMR of the hydrosilylation of phenylacetylene catalyzed by **Rh1@SBA-15**.

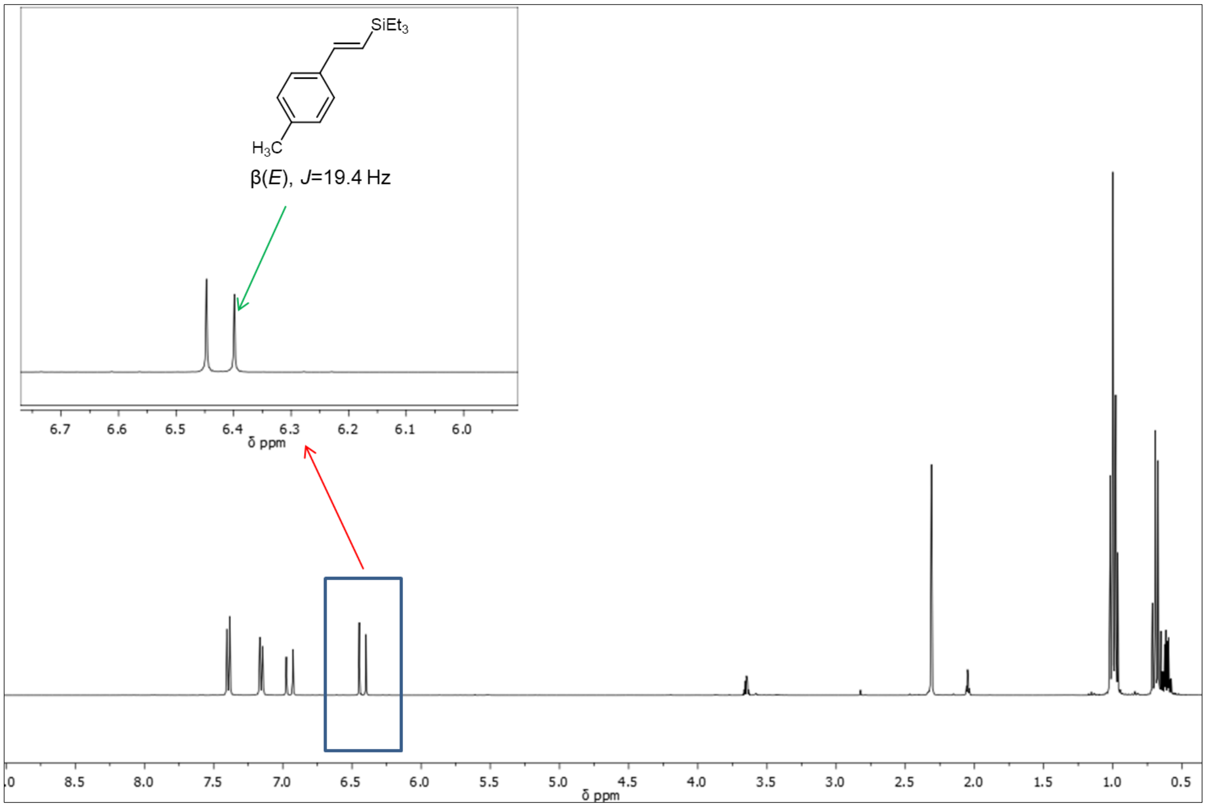

Figure S85. ^{1}H NMR of the hydrosilylation of 4-ethynyltoluene catalyzed by **Rh1**.

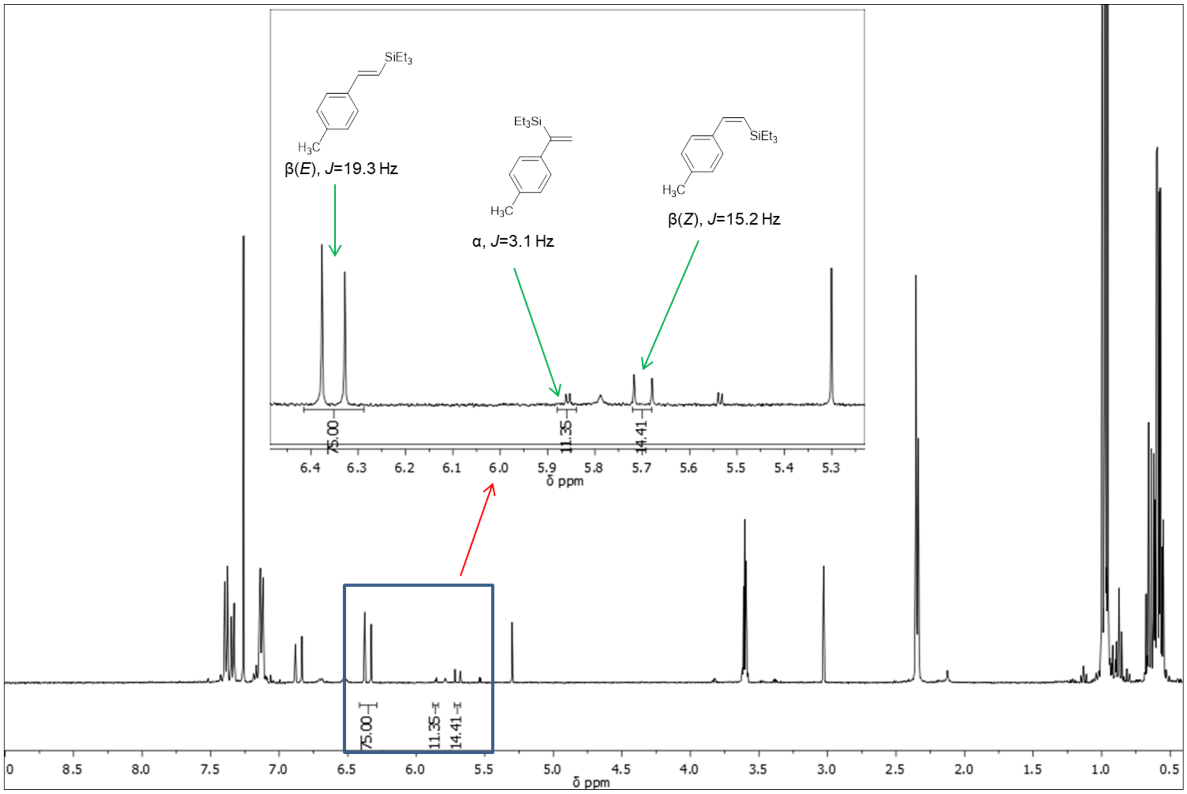

Figure S86. ^{1}H NMR of the hydrosilylation of 4-ethynyltoluene catalyzed by **Rh1@SBA-15**.

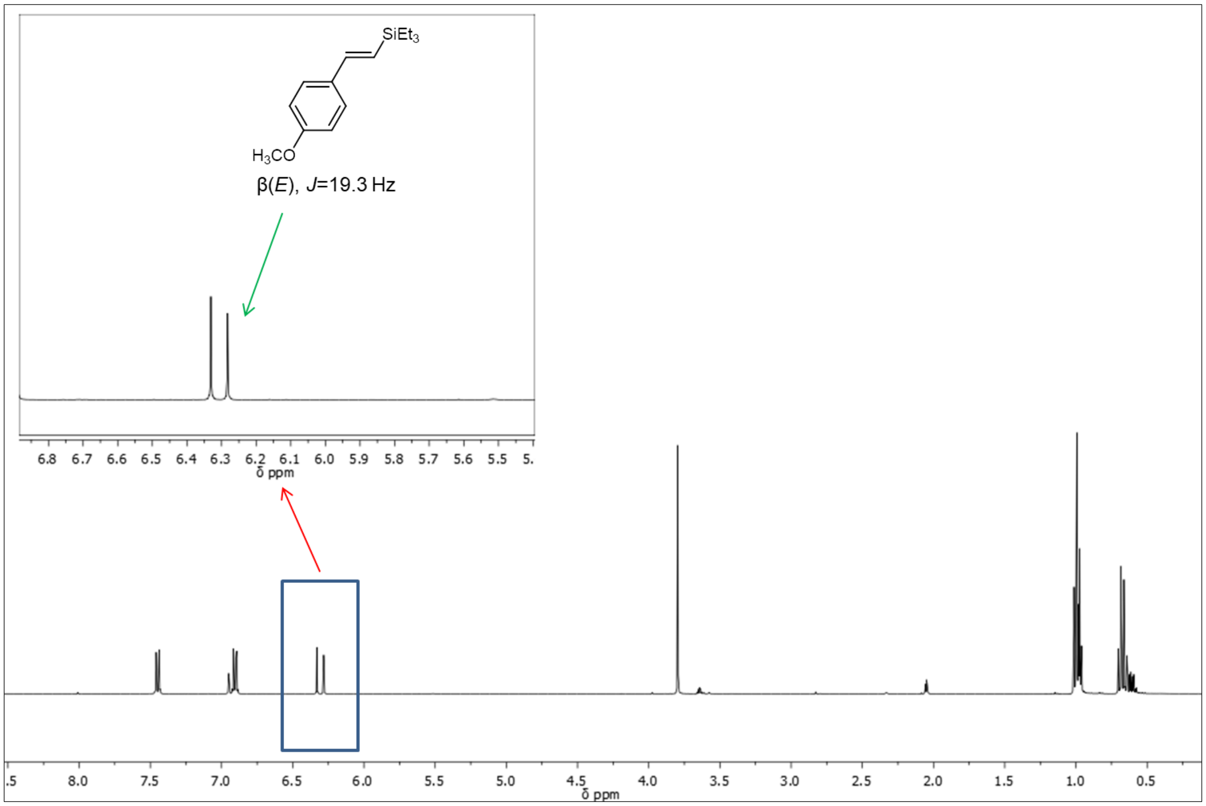

Figure S87. ^{1}H NMR of the hydrosilylation of 4-ethynylanisole catalyzed by **Rh1**.

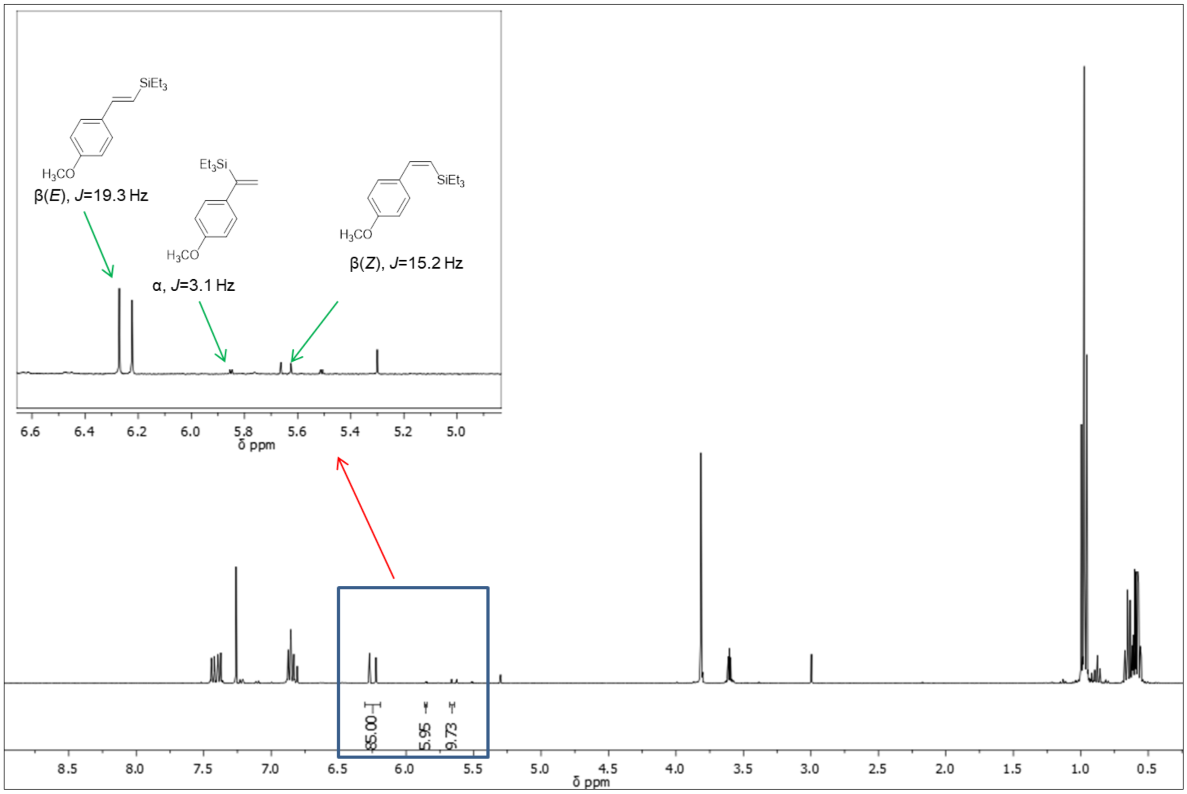

Figure S88. ^{1}H NMR of the hydrosilylation of 4-ethynylanisole catalyzed by **Rh1@SBA-15**.

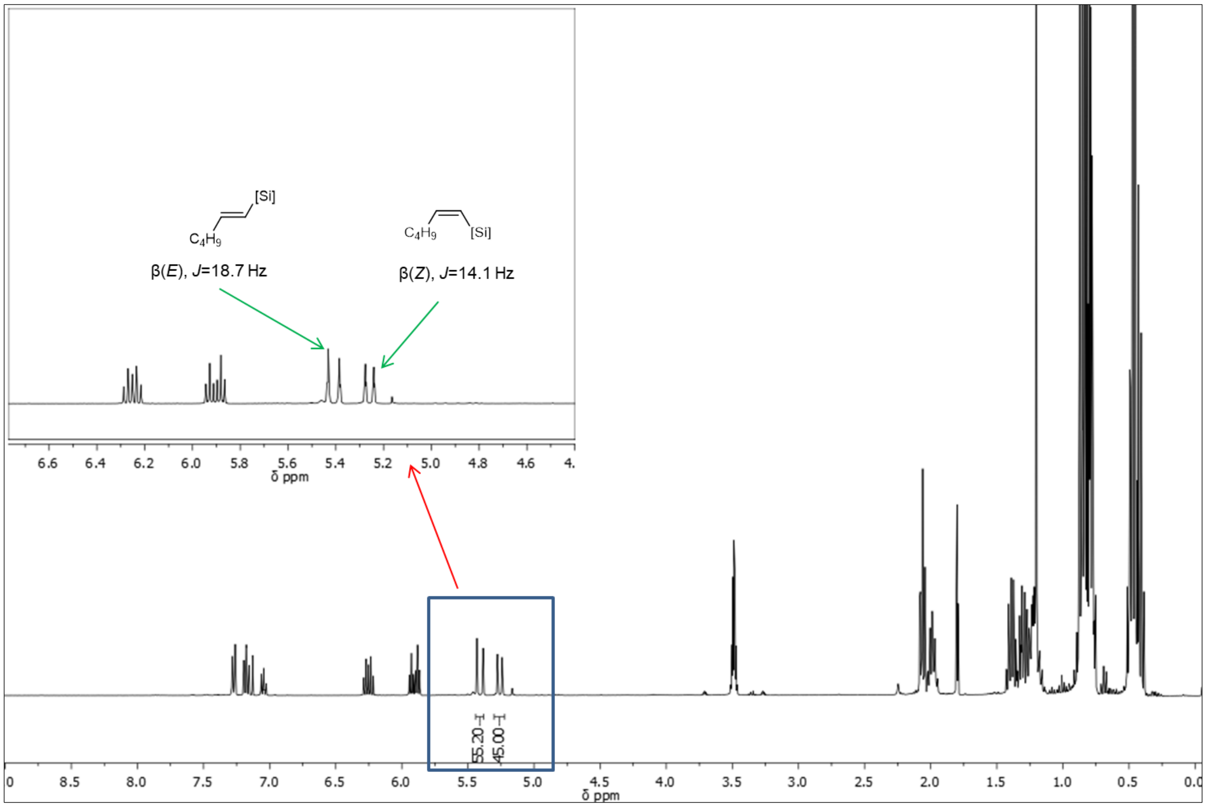

Figure S89. ^{1}H NMR of the hydrosilylation of 1-hexyne catalyzed by **Rh1**.

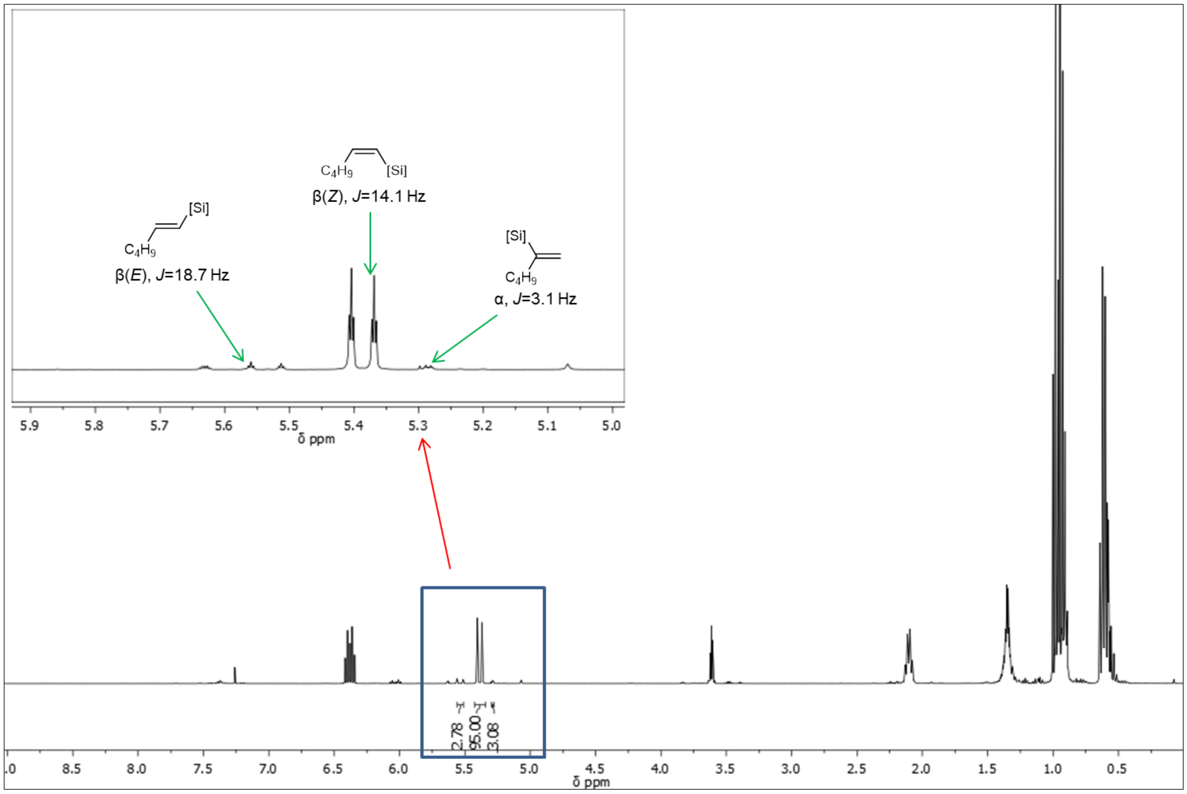

Figure S90. ^{1}H NMR of the hydrosilylation of 1-hexyne catalyzed by **Rh1@SBA-15**.

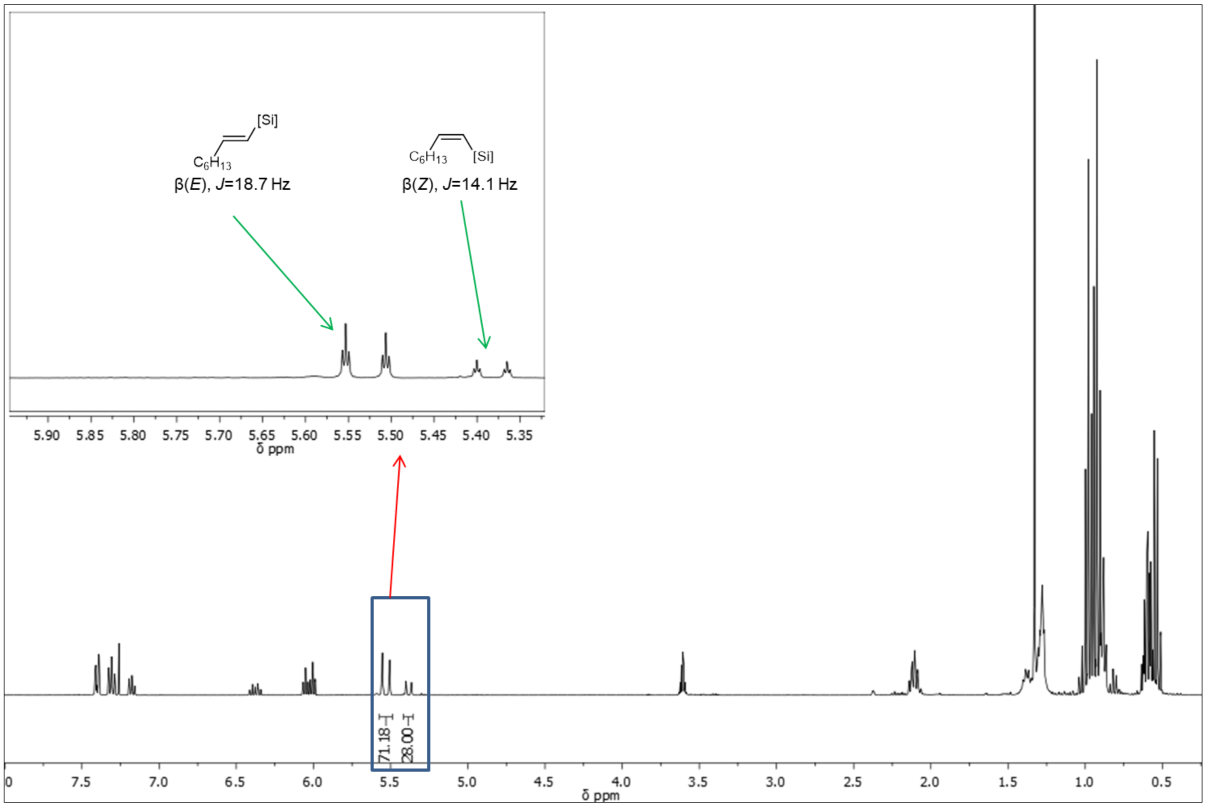

Figure S91. ^{1}H NMR of the hydrosilylation of 1-octyne catalyzed by **Rh1**.

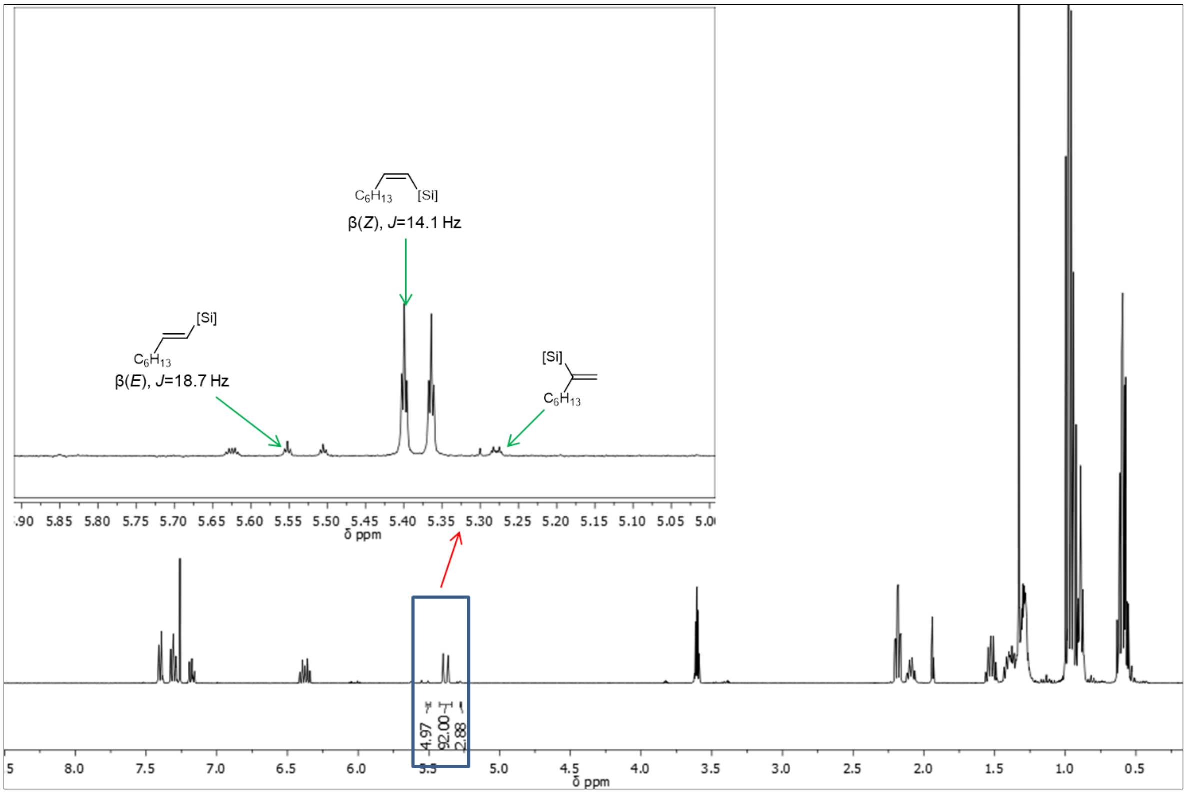

Figure S92. ^{1}H NMR of the hydrosilylation of 1-octyne catalyzed by **Rh1@SBA-15**.

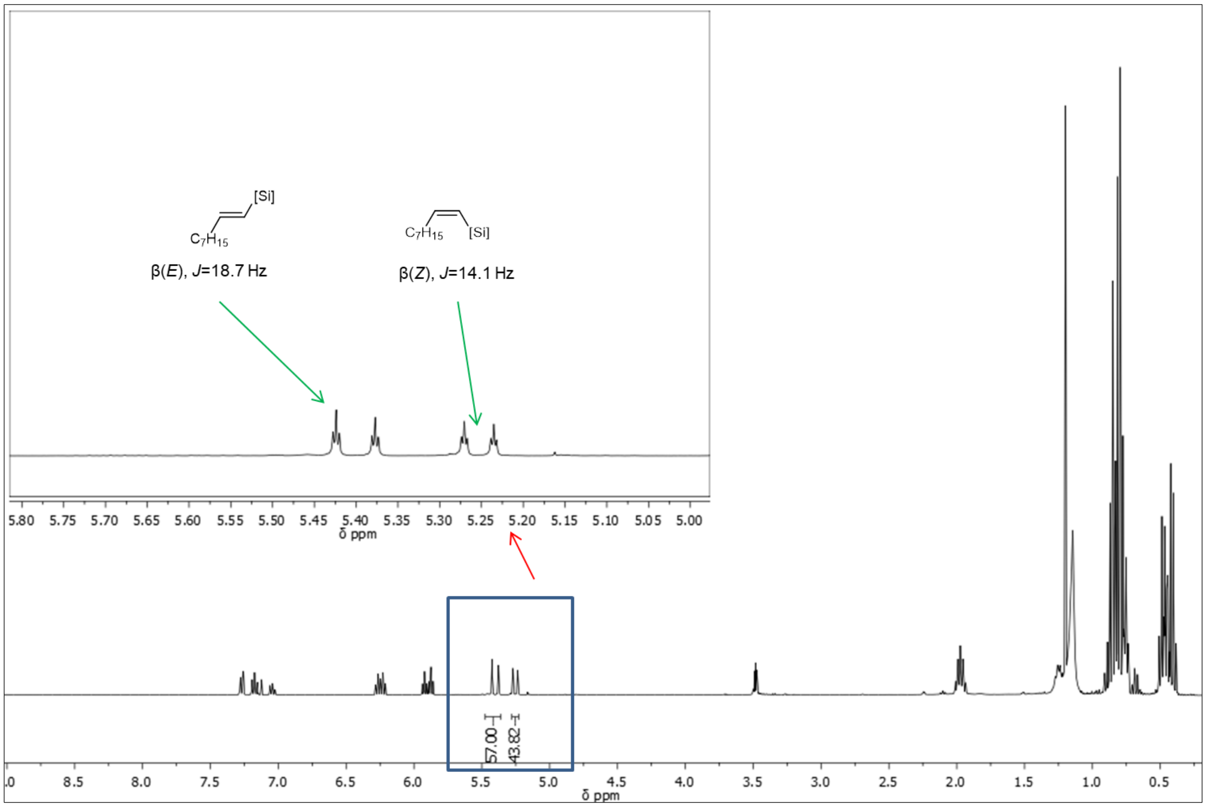

Figure S93. ^{1}H NMR of the hydrosilylation of 1-nonyne catalyzed by **Rh1**.

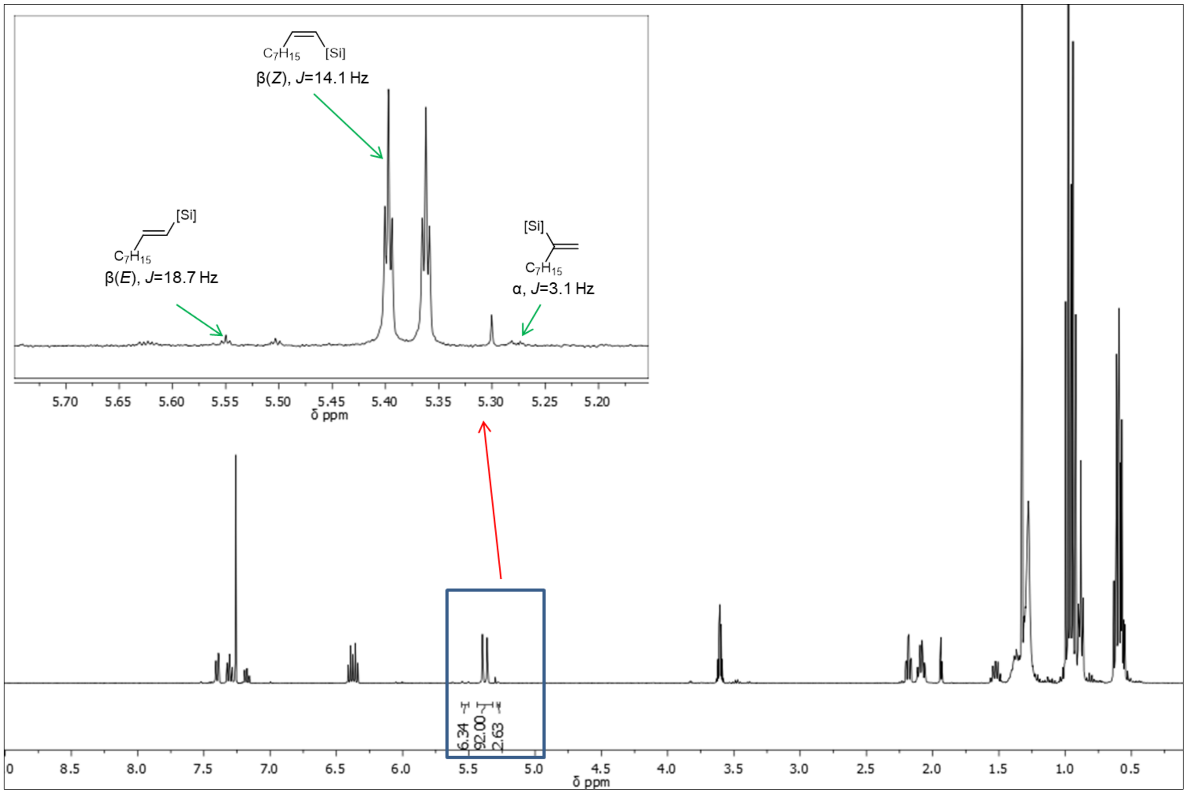

Figure S94. ^{1}H NMR of the hydrosilylation of 1-nonyne catalyzed by **Rh3@SBA-15**.

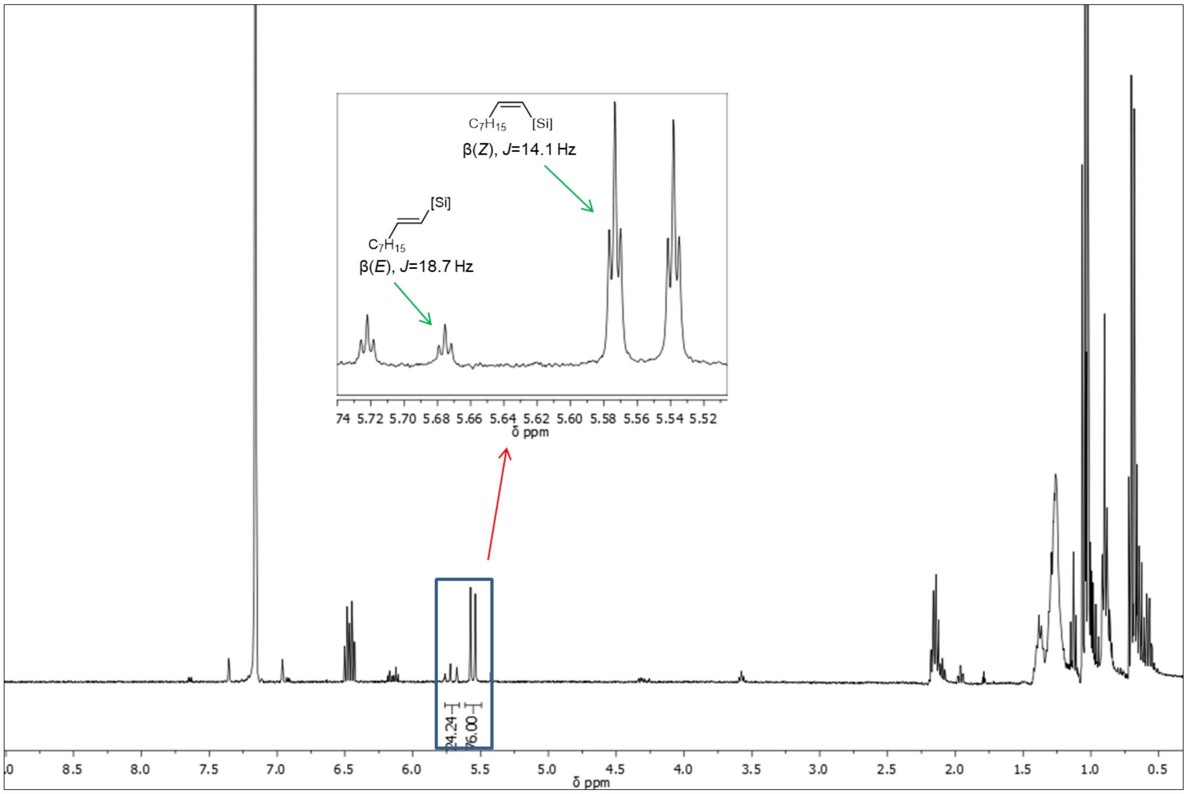

Figure S95. ^{1}H NMR of the hydrosilylation of 1-nonyne catalyzed by **Rh3@SBA-15***.

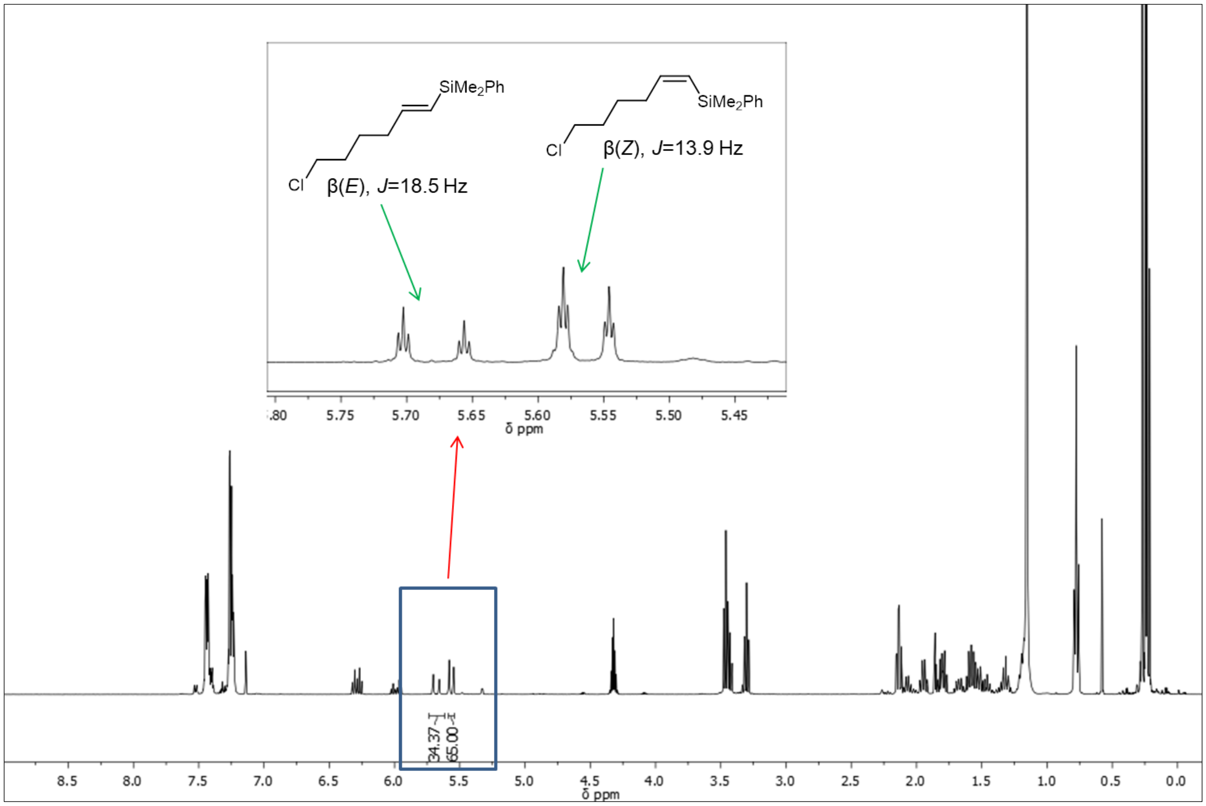

Figure S96. ^{1}H NMR of the hydrosilylation of 6-chloro-1-hexyne catalyzed by **Rh1**.

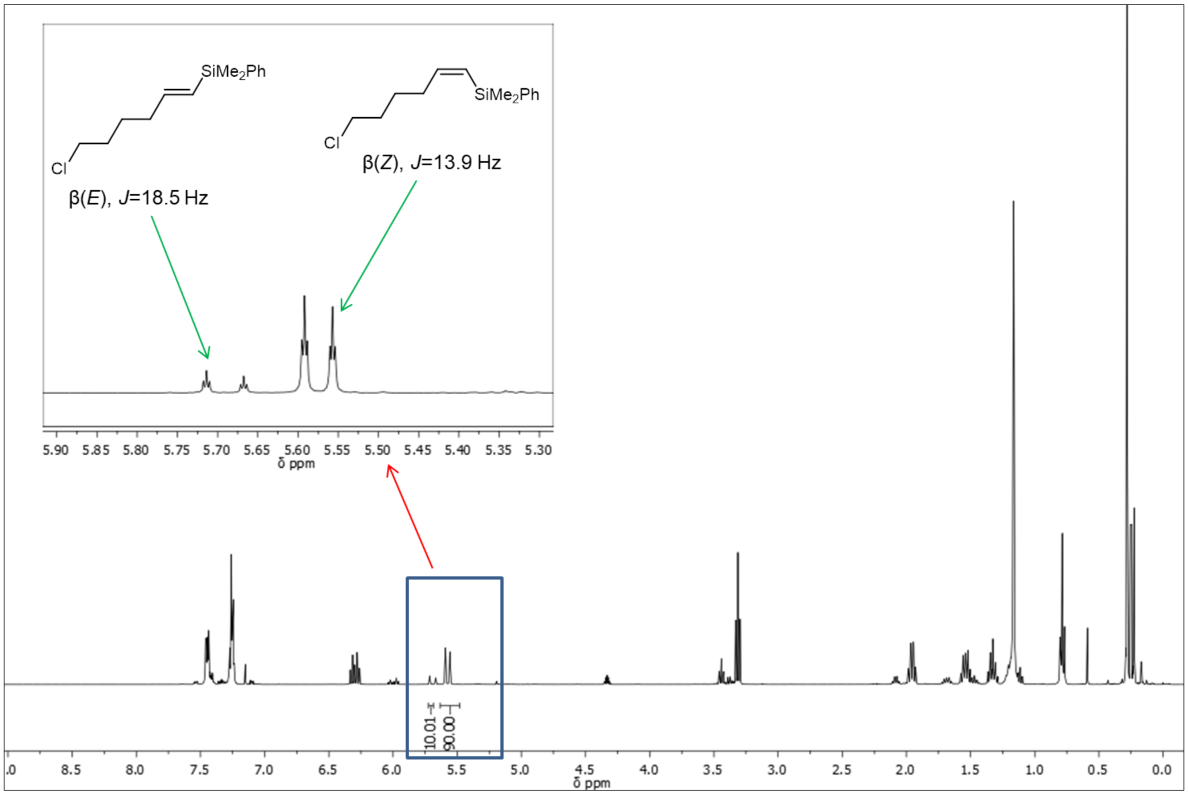

Figure S97. ^{1}H NMR of the hydrosilylation of 6-chloro-1-hexyne catalyzed by **Rh1@SBA-15**.

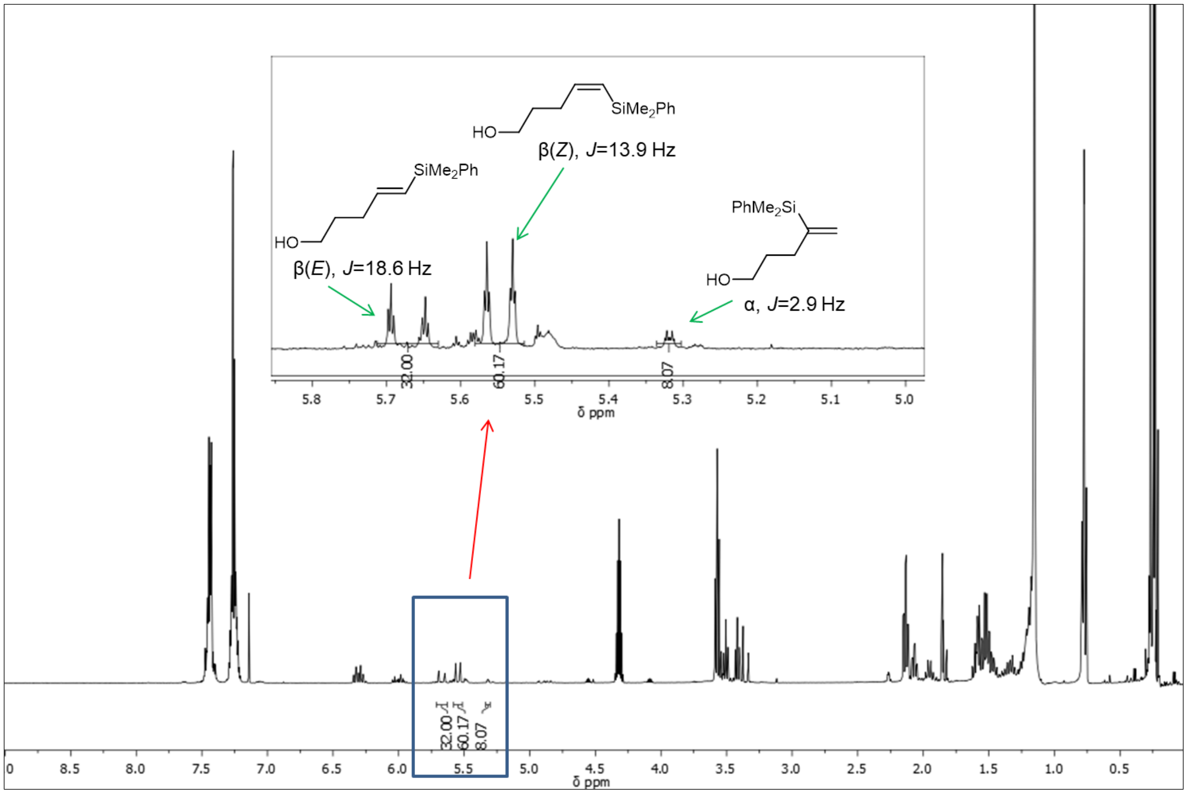

Figure S98. ^{1}H NMR of the hydrosilylation of 4-pentyn-1-ol catalyzed by **Rh1**.

Single-Crystal X-Ray Data

Crystal data of **Rh3:**

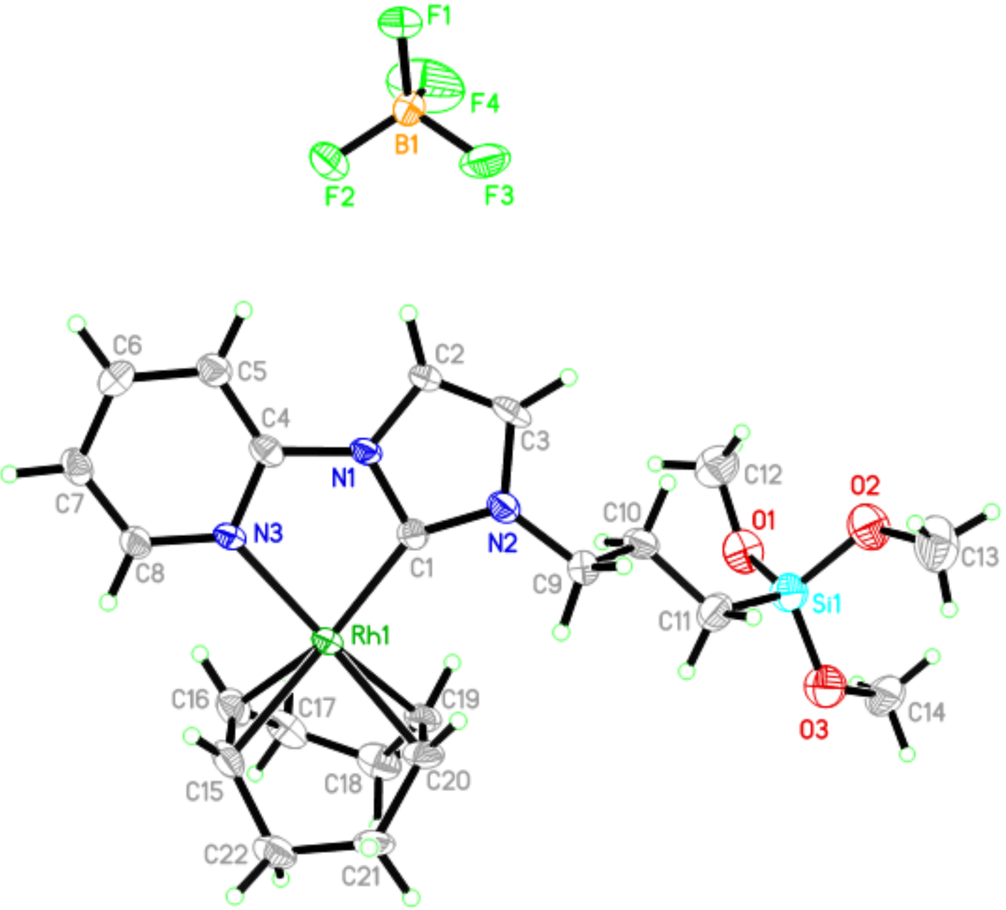

Figure S99. Single crystal X-ray structure of **Rh3**. Co-crystallized solvent molecules were omitted for clarity.

Table S3. Crystal data and structure refinement for **Rh3**.

Empirical formula	$C_{23}H_{35}BCl_2F_4N_3O_3RhSi$
Formula weight	690.25
Temperature	140(2) K
Wavelength	0.71073 A
Crystal system, space group	Triclinic, P-1
Unit cell dimensions	a = 10.4252(9) Å, α = 93.374(5)°
	b = 10.8008(9) Å, β = 95.300(5)°
	c = 13.8830(12) Å, γ = 115.441(4)°
Volume	1397.1(2) Å^3
Z, Calculated density	2, 1.641 Mg/m^3

Absorption coefficient	0.903 mm^{-1}
F (000)	704
Crystal size	0.111 x 0.101 x 0.060 mm
Θ range for data collection	1.482 to 25.427 °.
Limiting indices	-12<=h<=12 -13<=k<=13 -16<=l<=16
Reflections collected / unique	22306 / 5119 [R(int) = 0.0990]
Completeness to Θ = 25.242	99.7 %
Absorption correction	Numerical
Max. and min. transmission	0.9632 and 0.8919
Refinement method	Full-matrix least-squares on F^2
Data / restraints / parameters	5119 / 0 / 346
Goodness-of-fit on F^2	1.012
Final R indices [I>2sigma(I)]	R1 = 0.0613, wR2 = 0.1232
R indices (all data)	R1 = 0.1163, wR2 = 0.1372
Extinction coefficient	n/a
Largest diff. peak and hole	0.927 and -0.695 e.$Å^{-3}$

Table S4. Atomic coordinates ($x10^4$) and equivalent isotropic displacement parameters ($Å^2x10^3$) for **Rh3**. U(eq) is defined as one third of the trace of the orthogonalized U_{ij} tensor.

	x	y	z	U(eq)
Rh(1)	3393(1)	10064(1)	3128(1)	22(1)
Si(1)	-1448(2)	3068(2)	773(1)	34(1)
O(1)	-446(5)	3350(5)	-79(3)	41(1)
N(1)	4870(5)	8499(5)	3771(3)	21(1)
C(1)	3507(6)	8293(6)	3410(4)	21(1)
N(2)	2758(5)	6894(5)	3346(4)	24(1)
O(2)	-1668(5)	1576(5)	1083(3)	45(1)
C(2)	4937(7)	7289(6)	3927(5)	26(2)
N(3)	5528(5)	10824(5)	3798(4)	22(1)
C(3)	3634(7)	6289(7)	3669(4)	29(2)
O(3)	-2959(5)	3024(5)	314(3)	44(1)
C(4)	5969(6)	9850(6)	3991(4)	21(1)
C(5)	7349(6)	10134(6)	4356(4)	26(2)
C(6)	8322(7)	11497(7)	4559(4)	28(2)
C(7)	7893(7)	12513(6)	4390(4)	25(2)
C(8)	6500(6)	12131(6)	4019(4)	24(1)
C(9)	1231(6)	6109(6)	3036(5)	28(2)
C(10)	862(7)	5094(6)	2144(4)	28(2)
C(11)	-750(7)	4398(7)	1826(5)	35(2)

C(12)	867(8)	3239(8)	-2(6)	49(2)
C(13)	-2597(9)	888(9)	1766(6)	58(2)
C(14)	-3876(7)	2056(8)	-465(5)	44(2)
C(15)	3288(7)	12083(6)	3104(5)	29(2)
C(16)	3883(7)	11838(6)	2314(5)	33(2)
C(17)	3127(8)	11295(7)	1288(5)	38(2)
C(18)	1636(8)	10104(7)	1237(5)	36(2)
C(19)	1546(6)	9179(6)	2039(4)	28(2)
C(20)	1108(7)	9328(7)	2936(5)	28(2)
C(21)	728(7)	10463(7)	3281(5)	32(2)
C(22)	1771(7)	11909(7)	3048(5)	34(2)
B(1)	7882(8)	6090(8)	4408(6)	28(2)
F(1)	8655(4)	5812(4)	5155(3)	44(1)
F(2)	8231(4)	7463(4)	4456(3)	49(1)
F(3)	6443(4)	5351(5)	4425(4)	69(2)
F(4)	8200(6)	5666(6)	3551(3)	82(2)
C(1X)	5997(8)	5518(9)	1796(7)	65(3)
Cl(1)	4275(2)	5056(2)	1289(2)	74(1)
Cl(2)	7062(3)	7315(3)	1879(2)	78(1)

Table S5. Bond lengths [Å] and angles [°] for **Rh3**.

Rh(1)-C(1)	2.025(6)
Rh(1)-N(3)	2.110(5)
Rh(1)-C(20)	2.146(6)
Rh(1)-C(19)	2.149(6)
Rh(1)-C(16)	2.175(6)
Rh(1)-C(15)	2.232(6)
Si(1)-O(1)	1.607(5)
Si(1)-O(2)	1.617(5)
Si(1)-O(3)	1.624(5)
Si(1)-C(11)	1.840(7)
O(1)-C(12)	1.420(8)
N(1)-C(2)	1.368(7)
N(1)-C(1)	1.380(7)
N(1)-C(4)	1.408(7)
C(1)-N(2)	1.363(7)
N(2)-C(3)	1.390(7)
N(2)-C(9)	1.453(7)
O(2)-C(13)	1.423(8)
C(2)-C(3)	1.321(9)

C(2)-H(2)	0.9500
N(3)-C(8)	1.333(7)
N(3)-C(4)	1.347(7)
C(3)-H(3)	0.9500
O(3)-C(14)	1.412(8)
C(4)-C(5)	1.374(8)
C(5)-C(6)	1.375(9)
C(5)-H(5)	0.9500
C(6)-C(7)	1.376(9)
C(6)-H(6)	0.9500
C(7)-C(8)	1.366(8)
C(7)-H(7)	0.9500
C(8)-H(8)	0.9500
C(9)-C(10)	1.508(9)
C(9)-H(9A)	0.9900
C(9)-H(9B)	0.9900
C(10)-C(11)	1.524(8)
C(10)-H(10A)	0.9900
C(10)-H(10B)	0.9900
C(11)-H(11A)	0.9900
C(11)-H(11B)	0.9900
C(12)-H(12A)	0.9800
C(12)-H(12B)	0.9800
C(12)-H(12C)	0.9800
C(13)-H(13A)	0.9800
C(13)-H(13B)	0.9800
C(13)-H(13C)	0.9800
C(14)-H(14A)	0.9800
C(14)-H(14B)	0.9800
C(14)-H(14C)	0.9800
C(15)-C(16)	1.375(9)
C(15)-C(22)	1.504(9)
C(15)-H(15)	0.9500
C(16)-C(17)	1.514(9)
C(16)-H(16)	0.9500
C(17)-C(18)	1.525(9)
C(17)-H(17A)	0.9900
C(17)-H(17B)	0.9900
C(18)-C(19)	1.521(9)
C(18)-H(18A)	0.9900
C(18)-H(18B)	0.9900

C(19)-C(20)	1.389(9)
C(19)-H(19)	0.9500
C(20)-C(21)	1.508(8)
C(20)-H(20)	0.9500
C(21)-C(22)	1.546(9)
C(21)-H(21A)	0.9900
C(21)-H(21B)	0.9900
C(22)-H(22A)	0.9900
C(22)-H(22B)	0.9900
B(1)-F(2)	1.362(8)
B(1)-F(3)	1.367(8)
B(1)-F(4)	1.368(9)
B(1)-F(1)	1.381(8)
C(1X)-Cl(1)	1.710(8)
C(1X)-Cl(2)	1.764(8)
C(1X)-H(1X1)	0.9900
C(1X)-H(1X2)	0.9900

C(1)-Rh(1)-N(3)	78.7(2)
C(1)-Rh(1)-C(20)	99.4(2)
N(3)-Rh(1)-C(20)	161.2(2)
C(1)-Rh(1)-C(19)	97.8(2)
N(3)-Rh(1)-C(19)	160.8(2)
C(20)-Rh(1)-C(19)	37.7(2)
C(1)-Rh(1)-C(16)	152.4(3)
N(3)-Rh(1)-C(16)	92.7(2)
C(20)-Rh(1)-C(16)	96.9(3)
C(19)-Rh(1)-C(16)	81.8(2)
C(1)-Rh(1)-C(15)	169.8(2)
N(3)-Rh(1)-C(15)	97.7(2)
C(20)-Rh(1)-C(15)	80.9(2)
C(19)-Rh(1)-C(15)	88.7(2)
C(16)-Rh(1)-C(15)	36.3(2)
O(1)-Si(1)-O(2)	105.0(3)
O(1)-Si(1)-O(3)	107.4(3)
O(2)-Si(1)-O(3)	111.0(3)
O(1)-Si(1)-C(11)	115.2(3)
O(2)-Si(1)-C(11)	111.7(3)
O(3)-Si(1)-C(11)	106.5(3)
C(12)-O(1)-Si(1)	123.8(4)

C(2)-N(1)-C(1)	112.2(5)
C(2)-N(1)-C(4)	127.8(5)
C(1)-N(1)-C(4)	119.8(5)
N(2)-C(1)-N(1)	102.3(5)
N(2)-C(1)-Rh(1)	144.7(4)
N(1)-C(1)-Rh(1)	113.0(4)
C(1)-N(2)-C(3)	111.2(5)
C(1)-N(2)-C(9)	125.8(5)
C(3)-N(2)-C(9)	122.9(5)
C(13)-O(2)-Si(1)	122.9(5)
C(3)-C(2)-N(1)	106.9(5)
C(3)-C(2)-H(2)	126.6
N(1)-C(2)-H(2)	126.6
C(8)-N(3)-C(4)	116.4(5)
C(8)-N(3)-Rh(1)	128.5(4)
C(4)-N(3)-Rh(1)	115.0(4)
C(2)-C(3)-N(2)	107.5(5)
C(2)-C(3)-H(3)	126.3
N(2)-C(3)-H(3)	126.3
C(14)-O(3)-Si(1)	123.4(5)
N(3)-C(4)-C(5)	124.0(6)
N(3)-C(4)-N(1)	113.0(5)
C(5)-C(4)-N(1)	123.0(5)
C(4)-C(5)-C(6)	117.6(6)
C(4)-C(5)-H(5)	121.2
C(6)-C(5)-H(5)	121.2
C(5)-C(6)-C(7)	119.6(6)
C(5)-C(6)-H(6)	120.2
C(7)-C(6)-H(6)	120.2
C(8)-C(7)-C(6)	118.6(6)
C(8)-C(7)-H(7)	120.7
C(6)-C(7)-H(7)	120.7
N(3)-C(8)-C(7)	123.7(6)
N(3)-C(8)-H(8)	118.1
C(7)-C(8)-H(8)	118.1
N(2)-C(9)-C(10)	114.2(5)
N(2)-C(9)-H(9A)	108.7
C(10)-C(9)-H(9A)	108.7
N(2)-C(9)-H(9B)	108.7
C(10)-C(9)-H(9B)	108.7
H(9A)-C(9)-H(9B)	107.6

C(9)-C(10)-C(11)	110.7(5)
C(9)-C(10)-H(10A)	109.5
C(11)-C(10)-H(10A)	109.5
C(9)-C(10)-H(10B)	109.5
C(11)-C(10)-H(10B)	109.5
H(10A)-C(10)-H(10B)	108.1
C(10)-C(11)-Si(1)	118.0(5)
C(10)-C(11)-H(11A)	107.8
Si(1)-C(11)-H(11A)	107.8
C(10)-C(11)-H(11B)	107.8
Si(1)-C(11)-H(11B)	107.8
H(11A)-C(11)-H(11B)	107.1
O(1)-C(12)-H(12A)	109.5
O(1)-C(12)-H(12B)	109.5
H(12A)-C(12)-H(12B)	109.5
O(1)-C(12)-H(12C)	109.5
H(12A)-C(12)-H(12C)	109.5
H(12B)-C(12)-H(12C)	109.5
O(2)-C(13)-H(13A)	109.5
O(2)-C(13)-H(13B)	109.5
H(13A)-C(13)-H(13B)	109.5
O(2)-C(13)-H(13C)	109.5
H(13A)-C(13)-H(13C)	109.5
H(13B)-C(13)-H(13C)	109.5
O(3)-C(14)-H(14A)	109.5
O(3)-C(14)-H(14B)	109.5
H(14A)-C(14)-H(14B)	109.5
O(3)-C(14)-H(14C)	109.5
H(14A)-C(14)-H(14C)	109.5
H(14B)-C(14)-H(14C)	109.5
C(16)-C(15)-C(22)	123.7(6)
C(16)-C(15)-Rh(1)	69.6(4)
C(22)-C(15)-Rh(1)	111.7(4)
C(16)-C(15)-H(15)	118.2
C(22)-C(15)-H(15)	118.2
Rh(1)-C(15)-H(15)	88.7
C(15)-C(16)-C(17)	126.4(6)
C(15)-C(16)-Rh(1)	74.1(4)
C(17)-C(16)-Rh(1)	107.1(4)
C(15)-C(16)-H(16)	116.8
C(17)-C(16)-H(16)	116.8

Rh(1)-C(16)-H(16)	88.8
C(16)-C(17)-C(18)	113.8(6)
C(16)-C(17)-H(17A)	108.8
C(18)-C(17)-H(17A)	108.8
C(16)-C(17)-H(17B)	108.8
C(18)-C(17)-H(17B)	108.8
H(17A)-C(17)-H(17B)	107.7
C(19)-C(18)-C(17)	112.1(5)
C(19)-C(18)-H(18A)	109.2
C(17)-C(18)-H(18A)	109.2
C(19)-C(18)-H(18B)	109.2
C(17)-C(18)-H(18B)	109.2
H(18A)-C(18)-H(18B)	107.9
C(20)-C(19)-C(18)	124.5(6)
C(20)-C(19)-Rh(1)	71.0(3)
C(18)-C(19)-Rh(1)	112.5(4)
C(20)-C(19)-H(19)	117.7
C(18)-C(19)-H(19)	117.7
Rh(1)-C(19)-H(19)	86.4
C(19)-C(20)-C(21)	125.5(6)
C(19)-C(20)-Rh(1)	71.2(4)
C(21)-C(20)-Rh(1)	109.8(4)
C(19)-C(20)-H(20)	117.3
C(21)-C(20)-H(20)	117.3
Rh(1)-C(20)-H(20)	88.9
C(20)-C(21)-C(22)	113.8(6)
C(20)-C(21)-H(21A)	108.8
C(22)-C(21)-H(21A)	108.8
C(20)-C(21)-H(21B)	108.8
C(22)-C(21)-H(21B)	108.8
H(21A)-C(21)-H(21B)	107.7
C(15)-C(22)-C(21)	112.1(5)
C(15)-C(22)-H(22A)	109.2
C(21)-C(22)-H(22A)	109.2
C(15)-C(22)-H(22B)	109.2
C(21)-C(22)-H(22B)	109.2
H(22A)-C(22)-H(22B)	107.9
F(2)-B(1)-F(3)	110.1(6)
F(2)-B(1)-F(4)	109.9(6)
F(3)-B(1)-F(4)	107.5(6)
F(2)-B(1)-F(1)	111.1(6)

F(3)-B(1)-F(1)	110.8(6)
F(4)-B(1)-F(1)	107.4(6)
Cl(1)-C(1X)-Cl(2)	112.1(5)
Cl(1)-C(1X)-H(1X1)	109.2
Cl(2)-C(1X)-H(1X1)	109.2
Cl(1)-C(1X)-H(1X2)	109.2
Cl(2)-C(1X)-H(1X2)	109.2
H(1X1)-C(1X)-H(1X2)	107.9

Table S6. Anisotropic displacement parameters ($Å^2x10^3$) for **Rh3**. The anisotropic displacement factor exponent takes the form: $-2 \Pi^2[h^2a^{*2}U_{11} + ... +2hka^*b^*U_{12}]$.

	U11	U22	U33	U23	U13	U12
Rh(1)	22(1)	18(1)	29(1)	0(1)	1(1)	12(1)
Si(1)	34(1)	32(1)	34(1)	-4(1)	-1(1)	14(1)
O(1)	49(3)	41(3)	31(3)	5(2)	6(2)	18(3)
N(1)	22(3)	18(3)	24(3)	-2(2)	0(2)	13(2)
C(1)	23(3)	20(4)	21(3)	-6(3)	-2(3)	11(3)
N(2)	26(3)	20(3)	27(3)	0(2)	1(2)	12(3)
O(2)	52(3)	42(3)	40(3)	9(2)	7(2)	19(3)
C(2)	24(4)	19(4)	39(4)	3(3)	-2(3)	13(3)
N(3)	23(3)	19(3)	30(3)	-1(2)	1(2)	14(3)
C(3)	40(4)	22(4)	35(4)	9(3)	8(3)	22(4)
O(3)	43(3)	40(3)	44(3)	-10(2)	-7(2)	17(3)
C(4)	28(4)	21(4)	20(3)	2(3)	9(3)	14(3)
C(5)	28(4)	24(4)	27(4)	0(3)	2(3)	14(3)
C(6)	20(3)	33(4)	28(4)	2(3)	0(3)	10(3)
C(7)	29(4)	20(4)	27(3)	-3(3)	9(3)	10(3)
C(8)	23(4)	20(4)	30(4)	0(3)	7(3)	10(3)
C(9)	24(4)	22(4)	36(4)	4(3)	4(3)	8(3)
C(10)	31(4)	26(4)	30(4)	-1(3)	-1(3)	17(3)
C(11)	26(4)	34(4)	42(4)	-3(3)	5(3)	11(3)
C(12)	43(5)	55(5)	49(5)	1(4)	6(4)	22(4)
C(13)	62(6)	55(6)	52(5)	25(4)	19(4)	17(5)
C(14)	31(4)	46(5)	49(5)	-4(4)	-9(4)	13(4)
C(15)	36(4)	17(4)	34(4)	3(3)	2(3)	12(3)
C(16)	39(4)	23(4)	37(4)	8(3)	0(3)	14(3)
C(17)	57(5)	32(4)	35(4)	5(3)	11(4)	27(4)
C(18)	51(5)	41(5)	23(4)	-2(3)	2(3)	29(4)
C(19)	30(4)	28(4)	31(4)	1(3)	-4(3)	19(3)
C(20)	30(4)	34(4)	26(4)	-3(3)	-6(3)	22(3)
C(21)	28(4)	36(4)	38(4)	-9(3)	1(3)	23(3)
C(22)	43(4)	34(4)	31(4)	-6(3)	-3(3)	26(4)
B(1)	20(4)	23(4)	38(5)	1(4)	1(3)	8(4)
F(1)	35(2)	35(2)	63(3)	7(2)	-11(2)	21(2)
F(2)	47(3)	25(2)	72(3)	3(2)	-16(2)	17(2)
F(3)	26(2)	60(3)	131(4)	50(3)	13(3)	22(2)
F(4)	122(5)	111(4)	52(3)	-7(3)	15(3)	87(4)
C(1X)	43(5)	52(6)	98(7)	15(5)	1(5)	19(5)
Cl(1)	59(2)	57(2)	99(2)	5(1)	-14(1)	25(1)

Cl(2)	59(2)	60(2)	103(2)	14(1)	12(1)	14(1)

Table S7. Hydrogen coordinates (x10^4) and isotropic displacement parameters (Å^{2}x10^3) for **Rh3**.

	x	y	z	U(eq)
H(2)	5763	7187	4174	32
H(3)	3347	5331	3699	35
H(5)	7621	9414	4464	31
H(6)	9286	11735	4814	33
H(7)	8551	13459	4528	30
H(8)	6207	12838	3913	29
H(9A)	815	6760	2902	33
H(9B)	779	5600	3577	33
H(10A)	1196	4386	2288	34
H(10B)	1359	5581	1609	34
H(11A)	-1052	5126	1679	42
H(11B)	-1222	3973	2388	42
H(12A)	1669	4163	87	73
H(12B)	909	2731	-598	73
H(12C)	930	2745	557	73
H(13A)	-2088	1219	2428	87
H(13B)	-2906	-108	1644	87
H(13C)	-3437	1078	1698	87
H(14A)	-3456	2289	-1071	67
H(14B)	-4811	2077	-525	67
H(14C)	-3998	1131	-341	67
H(15)	3866	12379	3719	35
H(16)	4867	12029	2423	40
H(17A)	3722	10983	917	46
H(17B)	3045	12058	969	46
H(18A)	929	10480	1294	43
H(18B)	1386	9548	597	43
H(19)	1808	8449	1917	34
H(20)	1042	8659	3367	34
H(21A)	716	10483	3994	38
H(21B)	-250	10249	2975	38
H(22A)	1459	12053	2386	40
H(22B)	1734	12619	3514	40
H(1X1)	6426	5042	1397	78
H(1X2)	5989	5214	2455	78

Table S8. Torsion angles [°] for **Rh3**.

O(2)-Si(1)-O(1)-C(12)	-51.2(6)
O(3)-Si(1)-O(1)-C(12)	-169.4(5)
C(11)-Si(1)-O(1)-C(12)	72.2(6)
C(2)-N(1)-C(1)-N(2)	-0.7(6)
C(4)-N(1)-C(1)-N(2)	-176.7(5)
C(2)-N(1)-C(1)-Rh(1)	-178.0(4)
C(4)-N(1)-C(1)-Rh(1)	6.0(7)
N(1)-C(1)-N(2)-C(3)	0.9(6)
Rh(1)-C(1)-N(2)-C(3)	176.6(6)
N(1)-C(1)-N(2)-C(9)	177.6(5)
Rh(1)-C(1)-N(2)-C(9)	-6.7(11)
O(1)-Si(1)-O(2)-C(13)	-174.5(5)
O(3)-Si(1)-O(2)-C(13)	-58.7(6)
C(11)-Si(1)-O(2)-C(13)	60.0(6)
C(1)-N(1)-C(2)-C(3)	0.2(7)
C(4)-N(1)-C(2)-C(3)	175.9(6)
N(1)-C(2)-C(3)-N(2)	0.3(7)
C(1)-N(2)-C(3)-C(2)	-0.8(7)
C(9)-N(2)-C(3)-C(2)	-177.6(5)
O(1)-Si(1)-O(3)-C(14)	61.0(6)
O(2)-Si(1)-O(3)-C(14)	-53.3(6)
C(11)-Si(1)-O(3)-C(14)	-175.1(5)
C(8)-N(3)-C(4)-C(5)	-2.8(9)
Rh(1)-N(3)-C(4)-C(5)	174.9(5)
C(8)-N(3)-C(4)-N(1)	177.8(5)
Rh(1)-N(3)-C(4)-N(1)	-4.5(6)
C(2)-N(1)-C(4)-N(3)	-176.2(6)
C(1)-N(1)-C(4)-N(3)	-0.9(8)
C(2)-N(1)-C(4)-C(5)	4.4(9)
C(1)-N(1)-C(4)-C(5)	179.7(6)
N(3)-C(4)-C(5)-C(6)	1.8(9)
N(1)-C(4)-C(5)-C(6)	-178.9(5)
C(4)-C(5)-C(6)-C(7)	-0.2(9)
C(5)-C(6)-C(7)-C(8)	-0.3(9)
C(4)-N(3)-C(8)-C(7)	2.3(9)
Rh(1)-N(3)-C(8)-C(7)	-175.0(4)
C(6)-C(7)-C(8)-N(3)	-0.8(9)
C(1)-N(2)-C(9)-C(10)	119.5(6)

C(3)-N(2)-C(9)-C(10)	-64.1(8)
N(2)-C(9)-C(10)-C(11)	-175.7(5)
C(9)-C(10)-C(11)-Si(1)	-177.8(5)
O(1)-Si(1)-C(11)-C(10)	-36.1(6)
O(2)-Si(1)-C(11)-C(10)	83.6(6)
O(3)-Si(1)-C(11)-C(10)	-155.0(5)
C(22)-C(15)-C(16)-C(17)	-3.4(10)
Rh(1)-C(15)-C(16)-C(17)	99.7(6)
C(22)-C(15)-C(16)-Rh(1)	-103.1(6)
C(15)-C(16)-C(17)-C(18)	-43.3(9)
Rh(1)-C(16)-C(17)-C(18)	39.2(7)
C(16)-C(17)-C(18)-C(19)	-34.3(8)
C(17)-C(18)-C(19)-C(20)	93.2(8)
C(17)-C(18)-C(19)-Rh(1)	11.3(7)
C(18)-C(19)-C(20)-C(21)	-3.4(10)
Rh(1)-C(19)-C(20)-C(21)	101.4(6)
C(18)-C(19)-C(20)-Rh(1)	-104.8(6)
C(19)-C(20)-C(21)-C(22)	-43.0(8)
Rh(1)-C(20)-C(21)-C(22)	37.6(6)
C(16)-C(15)-C(22)-C(21)	93.7(7)
Rh(1)-C(15)-C(22)-C(21)	14.4(7)
C(20)-C(21)-C(22)-C(15)	-34.6(8)

Crystal data of **Rh7:**

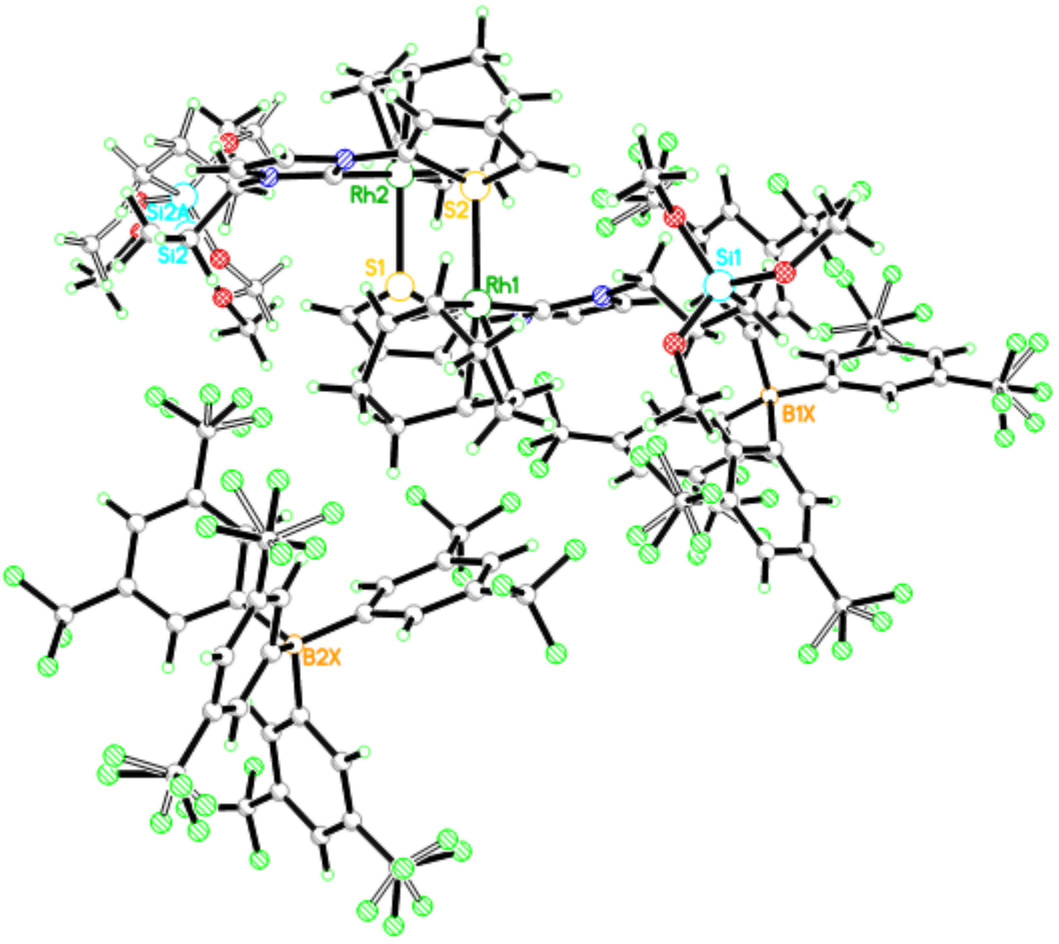

Figure S100. Single crystal X-ray structure of **Rh7**.

Table S9. Crystal data and structure refinement for **Rh7**

Identification code	**Rh7**
Empirical formula	$C_{106}H_{88}B_2F_{48}N_4O_6Rh_2S_2Si_2$
Formula weight	2773.54
Temperature	140(2) K
Wavelength	1.54178 Å
Crystal system, space group	Triclinic, P_{-1}
Unit cell dimensions	a = 12.9182(4) Å, α = 82.192(3)°
	b = 14.1003(5) Å, β = 86.437(3)°
	c = 32.0090(12) Å, γ = 86.386(3)°
Volume	5756.4(3) Å^3
Z, Calculated density	2, 1.600 Mg/m^3
Absorption coefficient	4.050 mm^{-1}
F(000)	2784

Crystal size	0.202 x 0.195 x 0.050 mm
Theta range for data collection	2.791 to 63.599°
Limiting indices	-14<=h<=15 -16<=k<=15 -36<=l<=36
Reflections collected / unique	58345 / 18216 [R(int) = 0.0824]
Completeness to theta = 63.599	96.4 %
Absorption correction	Numerical
Max. and min. transmission	0.8679 and 0.5466
Refinement method	Full-matrix least-squares on F^2
Data / restraints / parameters	18216 / 999 / 1899
Goodness-of-fit on F^2	1.036
Final R indices [I>2σ (I)]	R1 = 0.1011 wR2 = 0.2257
R indices (all data)	R1 = 0.1344 wR2 = 0.2369
Extinction coefficient	n/a
Largest diff. peak and hole	1.484 and -2.081 e.Å^{-3}

Table S10. Atomic coordinates (x10^4) and equivalent isotropic displacement parameters (Å^{2}x10^3) for **Rh7**. U(eq) is defined as one third of the trace of the orthogonalized U_{ij} tensor.

	**x**	**y**	**z**	**U(eq)**
Rh(1)	6780(1)	3722(1)	2292(1)	31(1)
Rh(2)	8414(1)	5590(1)	2859(1)	31(1)
S(1)	6768(2)	4891(2)	3011(1)	33(1)
S(2)	8457(2)	4397(2)	2146(1)	33(1)
Si(1)	6670(4)	3492(4)	602(1)	78(1)
C(1)	5993(7)	5027(7)	2079(3)	30(2)
N(1)	5671(6)	5655(6)	2339(3)	35(2)
O(1)	6863(9)	3214(9)	129(3)	90(4)
N(2)	5930(6)	5442(6)	1690(3)	35(2)
C(2)	5225(8)	6483(7)	2097(4)	40(3)
O(2)	7764(10)	3725(9)	783(4)	101(4)
N(3)	9488(6)	3619(5)	2842(3)	30(2)
C(3)	5389(8)	6317(9)	1696(4)	43(3)
O(3)	6206(13)	2626(9)	891(4)	114(5)
N(4)	9293(7)	3924(7)	3484(3)	41(2)
C(4)	5714(7)	5553(7)	2773(3)	31(2)
C(5)	6290(8)	5132(7)	3502(3)	36(2)
C(6)	5369(8)	5622(8)	3484(4)	41(3)
C(7)	5041(8)	5882(7)	3066(4)	38(3)
C(8)	6266(9)	5035(9)	1300(4)	50(3)
C(9)	5413(10)	4877(11)	1034(4)	63(4)
C(10)	5825(13)	4589(12)	607(4)	75(4)
C(11)	5520(20)	1960(20)	803(8)	169(12)

C(12)	8690(20)	3040(20)	748(8)	164(11)
C(13)	7235(18)	3911(18)	-218(6)	137(10)
C(14)	5440(8)	3031(9)	2132(4)	43(3)
C(15)	5267(7)	3301(8)	2539(4)	40(3)
C(16)	5381(9)	2664(9)	2958(4)	53(3)
C(17)	6334(9)	1988(9)	2978(5)	62(4)
C(18)	7229(7)	2357(6)	2705(3)	27(2)
C(19)	7459(9)	2256(8)	2313(4)	42(3)
C(20)	6856(10)	1725(10)	2038(5)	70(4)
C(21)	5726(10)	2004(10)	2048(5)	70(4)
C(22)	9031(7)	4296(6)	3089(3)	26(2)
C(23)	9926(8)	2838(7)	3098(3)	34(2)
C(24)	9811(9)	3024(7)	3492(4)	42(3)
C(25)	9436(7)	3667(6)	2406(3)	28(2)
C(26)	8880(8)	3994(8)	1671(4)	39(3)
C(27)	9733(8)	3387(7)	1718(4)	38(3)
C(28)	10051(8)	3215(7)	2139(3)	35(2)
C(29)	9055(11)	4414(10)	3845(4)	68(4)
C(30)	8476(19)	3872(15)	4218(5)	59(3)
C(31)	8500(20)	4376(11)	4606(7)	61(2)
Si(2)	7916(7)	5608(5)	4608(2)	62(2)
O(4)	7950(13)	5975(12)	5058(3)	65(2)
O(5)	8419(13)	6396(10)	4257(4)	67(2)
O(6)	6737(9)	5517(11)	4499(5)	68(2)
C(32)	7350(20)	5650(20)	5447(5)	73(4)
C(33)	9150(20)	7110(18)	4327(8)	76(4)
C(34)	5859(15)	6217(19)	4545(9)	80(4)
C(30A)	9856(16)	4460(20)	4168(6)	63(4)
C(31A)	9380(20)	4737(15)	4581(7)	63(2)
Si(2A)	8514(8)	5825(6)	4582(3)	64(2)
O(4A)	8410(15)	6218(13)	5033(4)	65(2)
O(5A)	9070(14)	6605(11)	4249(4)	68(2)
O(6A)	7394(11)	5632(13)	4437(5)	66(2)
C(32A)	7960(30)	5710(20)	5427(6)	71(4)
C(33A)	8840(30)	7653(11)	4247(9)	71(4)
C(34A)	6501(16)	6350(20)	4408(10)	75(4)
C(35)	9924(8)	6017(8)	2596(4)	42(3)
C(36)	9749(7)	6324(8)	3001(4)	38(3)
C(37)	9466(8)	7334(7)	3081(4)	43(3)
C(38)	8319(10)	7565(10)	3107(5)	65(4)
C(39)	7634(8)	7093(8)	2830(3)	39(3)
C(40)	7796(11)	6842(10)	2415(4)	62(4)
C(41)	8824(10)	7300(9)	2170(5)	59(3)
C(42)	9810(9)	6662(9)	2182(4)	53(3)
B(1X)	2397(9)	8580(8)	1169(4)	33(3)
C(1X)	2103(8)	9437(8)	789(3)	38(3)
C(2X)	1917(9)	9280(9)	378(4)	47(3)

C(3X)	1706(10)	10053(9)	60(4)	54(3)
C(4X)	1682(9)	10980(9)	141(4)	54(3)
C(5X)	1899(9)	11169(8)	540(4)	49(3)
C(6X)	2110(8)	10389(8)	855(4)	43(3)
C(7X)	1499(8)	9851(7)	-364(4)	70(4)
F(7A)	907(11)	9112(9)	-365(4)	78(4)
F(7B)	2368(10)	9644(16)	-588(4)	113(6)
F(7C)	979(19)	10560(12)	-593(6)	132(7)
F(7D)	2000(40)	10380(30)	-680(9)	79(12)
F(7E)	1650(50)	8942(13)	-430(14)	100(15)
F(7F)	590(20)	10210(40)	-507(18)	100(18)
C(8X)	1886(8)	12132(8)	654(3)	60(4)
F(8A)	1146(13)	12303(9)	946(5)	93(5)
F(8B)	2778(10)	12347(9)	798(6)	106(5)
F(8C)	1725(18)	12802(8)	327(4)	103(5)
F(8D)	2580(30)	12730(30)	468(15)	81(13)
F(8E)	1830(40)	12230(30)	1062(5)	84(13)
F(8F)	1060(30)	12660(30)	517(19)	111(17)
C(9X)	1890(9)	8833(7)	1624(3)	39(3)
C(10X)	969(8)	9398(8)	1660(4)	40(3)
C(11X)	540(9)	9595(8)	2045(4)	44(3)
C(12X)	971(8)	9197(7)	2419(4)	39(3)
C(13X)	1839(8)	8589(8)	2397(3)	39(3)
C(14X)	2301(8)	8408(7)	2004(3)	36(2)
C(15X)	-427(9)	10233(7)	2068(3)	62(4)
F(15B)	-1212(6)	9856(6)	2300(4)	98(3)
F(15A)	-314(11)	10897(10)	2321(5)	78(4)
F(15C)	-660(20)	10764(17)	1710(5)	124(8)
F(15D)	-333(17)	11147(9)	1938(12)	79(8)
F(15F)	-1052(16)	10060(20)	1771(8)	79(8)
C(16X)	2320(10)	8135(10)	2770(4)	53(3)
F(16A)	3352(7)	8135(9)	2761(3)	104(3)
F(16B)	2158(9)	7203(7)	2864(3)	106(3)
F(16C)	1978(8)	8475(8)	3126(3)	104(3)
C(17X)	3666(8)	8489(8)	1140(3)	38(3)
C(18X)	4298(8)	8882(7)	1403(4)	39(3)
C(19X)	5364(9)	8865(8)	1344(4)	46(3)
C(20X)	5885(9)	8451(8)	1019(4)	47(3)
C(21X)	5279(9)	8050(9)	747(4)	47(3)
C(22X)	4201(9)	8070(8)	808(3)	41(3)
C(23X)	5989(7)	9255(6)	1651(3)	56(3)
F(23A)	5461(9)	9860(11)	1881(5)	74(4)
F(23B)	6796(12)	9732(14)	1484(4)	94(6)
F(23C)	6377(16)	8601(8)	1946(5)	93(6)
F(23D)	6973(11)	8930(20)	1615(9)	84(8)
F(23E)	6000(30)	10198(8)	1563(11)	112(10)
F(23F)	5680(20)	9040(30)	2050(4)	106(10)

C(24X)	5822(11)	7608(12)	389(4)	63(4)
F(24A)	5265(6)	6987(7)	243(3)	79(2)
F(24B)	6727(7)	7204(8)	476(3)	97(3)
F(24C)	5980(8)	8296(8)	48(3)	97(3)
C(25X)	1897(8)	7561(7)	1128(3)	35(2)
C(26X)	918(8)	7498(8)	991(4)	43(3)
C(27X)	434(9)	6645(9)	1008(4)	49(3)
C(28X)	935(9)	5785(9)	1174(4)	48(3)
C(29X)	1918(9)	5814(7)	1324(4)	43(3)
C(30X)	2374(8)	6697(8)	1296(3)	40(3)
C(31X)	-645(11)	6628(8)	860(4)	80(5)
F(31A)	-968(15)	5759(9)	855(8)	122(8)
F(31B)	-680(13)	6955(16)	452(4)	105(6)
F(31C)	-1299(19)	7260(20)	1023(9)	164(12)
F(31D)	-1288(12)	6250(15)	1173(5)	49(5)
F(31E)	-1173(18)	7460(11)	783(11)	80(9)
F(31F)	-680(30)	6090(30)	554(12)	131(13)
C(32X)	2443(7)	4904(9)	1510(3)	64(4)
F(32A)	3260(9)	5020(14)	1727(5)	54(4)
F(32B)	2816(13)	4357(9)	1220(4)	103(5)
F(32C)	1840(11)	4390(13)	1794(6)	107(7)
F(32D)	1990(50)	4750(50)	1896(10)	110(30)
F(32E)	2080(30)	4149(18)	1372(13)	67(11)
F(32F)	3433(17)	4980(60)	1583(17)	70(20)
B(2X)	2622(9)	968(8)	3893(4)	32(3)
C(33X)	2675(7)	1684(7)	3455(3)	32(2)
C(34X)	2575(7)	2686(7)	3455(3)	32(2)
C(35X)	2594(8)	3337(7)	3083(3)	35(2)
C(36X)	2672(8)	3001(8)	2693(3)	38(3)
C(37X)	2738(7)	2013(7)	2683(3)	33(2)
C(38X)	2737(7)	1397(7)	3053(3)	32(2)
C(39X)	2505(8)	4384(8)	3115(4)	44(3)
F(39X)	3166(6)	4616(5)	3386(3)	72(2)
F(39B)	2715(8)	4929(5)	2760(3)	85(3)
F(39C)	1575(6)	4667(5)	3266(3)	86(3)
C(40X)	2800(8)	1634(8)	2268(4)	39(3)
F(40A)	3411(5)	860(5)	2260(2)	58(2)
F(40B)	3141(7)	2287(5)	1946(2)	68(2)
F(40C)	1868(5)	1414(5)	2162(2)	60(2)
C(41X)	3128(7)	-100(7)	3824(3)	31(2)
C(42X)	4089(8)	-190(8)	3595(3)	37(2)
C(43X)	4573(8)	-1075(8)	3544(4)	42(3)
C(44X)	4146(9)	-1909(8)	3723(4)	43(3)
C(45X)	3212(9)	-1851(8)	3957(4)	42(3)
C(46X)	2717(8)	-964(7)	4001(3)	35(2)
C(47X)	5555(9)	-1155(6)	3304(3)	73(4)
F(47A)	5536(12)	-1486(11)	2935(3)	70(5)

F(47B)	6131(9)	-393(7)	3258(5)	78(5)
F(47C)	6216(9)	-1834(9)	3517(4)	76(4)
F(47D)	5710(20)	-1900(11)	3079(6)	69(8)
F(47E)	5540(20)	-442(12)	2963(6)	83(7)
F(47F)	6513(14)	-1040(20)	3421(10)	137(13)
C(48X)	2748(8)	-2755(7)	4151(3)	57(3)
F(48A)	3015(16)	-3494(11)	3944(7)	74(6)
F(48B)	1718(8)	-2672(14)	4170(7)	82(6)
F(48C)	2983(19)	-3022(13)	4547(4)	95(6)
F(48D)	1900(20)	-2700(20)	4403(10)	86(10)
F(48E)	3450(20)	-3300(20)	4375(11)	109(12)
F(48F)	2570(30)	-3330(20)	3874(10)	79(11)
C(49X)	1377(7)	909(7)	4033(3)	29(2)
C(50X)	733(8)	538(7)	3768(3)	33(2)
C(51X)	-321(7)	505(7)	3847(3)	33(2)
C(52X)	-803(8)	850(7)	4206(3)	36(2)
C(53X)	-169(8)	1208(7)	4477(3)	33(2)
C(54X)	885(7)	1277(7)	4385(3)	30(2)
C(55X)	-964(8)	124(10)	3545(4)	48(3)
F(55A)	-506(10)	-578(10)	3351(5)	55(5)
F(55B)	-1873(15)	-110(20)	3685(7)	93(8)
F(55C)	-1180(20)	815(11)	3203(7)	96(8)
F(55D)	-1942(18)	479(19)	3558(8)	65(7)
F(55E)	-631(18)	330(30)	3165(8)	85(9)
F(55F)	-1090(30)	-805(16)	3669(10)	96(11)
C(56X)	-655(8)	1560(8)	4864(4)	41(3)
F(56A)	-441(8)	2426(6)	4909(3)	95(3)
F(56B)	-1645(7)	1516(10)	4913(3)	122(4)
F(56C)	-299(9)	1070(7)	5214(2)	100(3)
C(57X)	3330(7)	1282(7)	4257(3)	32(2)
C(58X)	4124(8)	1916(8)	4169(4)	40(3)
C(59X)	4771(8)	2093(8)	4477(4)	43(3)
C(60X)	4638(9)	1663(9)	4889(4)	47(3)
C(61X)	3865(8)	1024(8)	4983(3)	38(3)
C(62X)	3238(8)	851(8)	4673(3)	37(2)
C(63X)	5623(12)	2772(12)	4364(5)	69(4)
F(63A)	6319(18)	2720(20)	4644(7)	103(8)
F(63B)	6119(17)	2720(20)	4003(6)	96(7)
F(63C)	5180(15)	3702(12)	4318(10)	109(7)
F(63D)	6430(40)	2290(40)	4130(20)	96(19)
F(63E)	5900(50)	3170(40)	4677(18)	85(17)
F(63F)	5400(40)	3470(40)	4081(19)	69(16)
C(64X)	3702(9)	578(10)	5421(4)	51(3)
F(64A)	4569(6)	387(7)	5632(2)	84(3)
F(64B)	3298(6)	-301(6)	5454(2)	76(2)
F(64C)	3050(10)	1049(8)	5643(3)	133(5)

Table S11. Bond lengths [Å] and angles [°] for **Rh7**.

Rh(1)-C(1)	2.096(10)
Rh(1)-C(15)	2.150(10)
Rh(1)-C(14)	2.161(10)
Rh(1)-C(19)	2.189(11)
Rh(1)-C(18)	2.247(9)
Rh(1)-S(2)	2.415(3)
Rh(2)-C(22)	2.007(9)
Rh(2)-C(35)	2.163(10)
Rh(2)-C(36)	2.169(10)
Rh(2)-C(40)	2.248(14)
Rh(2)-C(39)	2.282(10)
Rh(2)-S(1)	2.395(3)
S(1)-C(5)	1.723(11)
S(1)-C(4)	1.754(10)
S(2)-C(26)	1.740(11)
S(2)-C(25)	1.757(10)
Si(1)-O(3)	1.556(14)
Si(1)-O(1)	1.616(11)
Si(1)-O(2)	1.628(14)
Si(1)-C(10)	1.837(15)
C(1)-N(2)	1.308(12)
C(1)-N(1)	1.326(13)
N(1)-C(4)	1.384(13)
N(1)-C(2)	1.421(12)
O(1)-C(13)	1.46(2)
N(2)-C(3)	1.381(14)
N(2)-C(8)	1.474(13)
C(2)-C(3)	1.338(15)
C(2)-H(2)	0.9500
O(2)-C(12)	1.49(3)
N(3)-C(25)	1.392(13)
N(3)-C(23)	1.393(12)
N(3)-C(22)	1.401(12)
C(3)-H(3)	0.9500
O(3)-C(11)	1.40(3)
N(4)-C(22)	1.358(12)
N(4)-C(24)	1.394(13)
N(4)-C(29)	1.433(14)
C(4)-C(7)	1.346(14)
C(5)-C(6)	1.337(15)
C(5)-H(5)	0.9500
C(6)-C(7)	1.423(15)
C(6)-H(6)	0.9500
C(7)-H(7)	0.9500

C(8)-C(9)	1.480(17)
C(8)-H(8A)	0.9900
C(8)-H(8B)	0.9900
C(9)-C(10)	1.532(17)
C(9)-H(9A)	0.9900
C(9)-H(9B)	0.9900
C(10)-H(10A)	0.9900
C(10)-H(10B)	0.9900
C(11)-H(11A)	0.9800
C(11)-H(11B)	0.9800
C(11)-H(11C)	0.9800
C(12)-H(12A)	0.9800
C(12)-H(12B)	0.9800
C(12)-H(12C)	0.9800
C(13)-H(13A)	0.9800
C(13)-H(13B)	0.9800
C(13)-H(13C)	0.9800
C(14)-C(15)	1.409(16)
C(14)-C(21)	1.526(17)
C(14)-H(14)	0.9500
C(15)-C(16)	1.517(16)
C(15)-H(15)	0.9500
C(16)-C(17)	1.508(17)
C(16)-H(16A)	0.9900
C(16)-H(16B)	0.9900
C(17)-C(18)	1.483(14)
C(17)-H(17A)	0.9900
C(17)-H(17B)	0.9900
C(18)-C(19)	1.298(14)
C(18)-H(18)	0.9500
C(19)-C(20)	1.509(16)
C(19)-H(19)	0.9500
C(20)-C(21)	1.487(17)
C(20)-H(20A)	0.9900
C(20)-H(20B)	0.9900
C(21)-H(21A)	0.9900
C(21)-H(21B)	0.9900
C(23)-C(24)	1.319(15)
C(23)-H(23)	0.9500
C(24)-H(24)	0.9500
C(25)-C(28)	1.331(14)
C(26)-C(27)	1.355(15)
C(26)-H(26)	0.9500
C(27)-C(28)	1.416(15)
C(27)-H(27)	0.9500
C(28)-H(28)	0.9500
C(29)-C(30)	1.508(10)

C(29)-C(30A)	1.516(10)
C(29)-H(29A)	0.9889
C(29)-H(29B)	0.9637
C(29)-H(29C)	0.9554
C(29)-H(29D)	0.9897
C(30)-C(31)	1.518(10)
C(30)-H(30A)	0.9900
C(30)-H(30B)	0.9900
C(31)-Si(2)	1.849(10)
C(31)-H(31A)	0.9900
C(31)-H(31B)	0.9900
Si(2)-O(4)	1.599(9)
Si(2)-O(6)	1.599(9)
Si(2)-O(5)	1.607(9)
O(4)-C(32)	1.465(10)
O(5)-C(33)	1.472(10)
O(6)-C(34)	1.470(10)
C(32)-H(32A)	0.9800
C(32)-H(32B)	0.9800
C(32)-H(32C)	0.9800
C(33)-H(33A)	0.9800
C(33)-H(33B)	0.9800
C(33)-H(33C)	0.9800
C(34)-H(34A)	0.9800
C(34)-H(34B)	0.9800
C(34)-H(34C)	0.9800
C(30A)-C(31A)	1.515(10)
C(30A)-H(30C)	0.9900
C(30A)-H(30D)	0.9900
C(31A)-Si(2A)	1.844(10)
C(31A)-H(31C)	0.9900
C(31A)-H(31D)	0.9900
Si(2A)-O(5A)	1.596(9)
Si(2A)-O(6A)	1.597(9)
Si(2A)-O(4A)	1.610(9)
O(4A)-C(32A)	1.470(10)
O(5A)-C(33A)	1.489(10)
O(6A)-C(34A)	1.483(10)
C(32A)-H(32D)	0.9800
C(32A)-H(32E)	0.9800
C(32A)-H(32F)	0.9800
C(33A)-H(33D)	0.9800
C(33A)-H(33E)	0.9800
C(33A)-H(33F)	0.9800
C(34A)-H(34D)	0.9800
C(34A)-H(34E)	0.9800
C(34A)-H(34F)	0.9800

C(35)-C(36)	1.424(15)
C(35)-C(42)	1.512(16)
C(35)-H(35)	0.9500
C(36)-C(37)	1.499(14)
C(36)-H(36)	0.9500
C(37)-C(38)	1.496(16)
C(37)-H(37A)	0.9900
C(37)-H(37B)	0.9900
C(38)-C(39)	1.530(16)
C(38)-H(38A)	0.9900
C(38)-H(38B)	0.9900
C(39)-C(40)	1.420(17)
C(39)-H(39)	0.9500
C(40)-C(41)	1.625(18)
C(40)-H(40)	0.9500
C(41)-C(42)	1.511(17)
C(41)-H(41A)	0.9900
C(41)-H(41B)	0.9900
C(42)-H(42A)	0.9900
C(42)-H(42B)	0.9900
B(1X)-C(9X)	1.634(16)
B(1X)-C(17X)	1.634(15)
B(1X)-C(25X)	1.636(16)
B(1X)-C(1X)	1.640(15)
C(1X)-C(6X)	1.388(15)
C(1X)-C(2X)	1.401(16)
C(2X)-C(3X)	1.411(16)
C(2X)-H(2X)	0.9500
C(3X)-C(4X)	1.365(18)
C(3X)-C(7X)	1.469(18)
C(4X)-C(5X)	1.388(18)
C(4X)-H(4X)	0.9500
C(5X)-C(6X)	1.412(16)
C(5X)-C(8X)	1.452(17)
C(6X)-H(6X)	0.9500
C(7X)-F(7D)	1.327(10)
C(7X)-F(7E)	1.328(10)
C(7X)-F(7C)	1.328(9)
C(7X)-F(7A)	1.330(7)
C(7X)-F(7F)	1.331(10)
C(7X)-F(7B)	1.332(9)
C(8X)-F(8D)	1.329(10)
C(8X)-F(8C)	1.329(9)
C(8X)-F(8E)	1.330(10)
C(8X)-F(8A)	1.330(6)
C(8X)-F(8F)	1.332(10)
C(8X)-F(8B)	1.333(9)

C(9X)-C(10X)	1.396(15)
C(9X)-C(14X)	1.400(15)
C(10X)-C(11X)	1.376(15)
C(10X)-H(10X)	0.9500
C(11X)-C(12X)	1.385(16)
C(11X)-C(15X)	1.496(15)
C(12X)-C(13X)	1.372(15)
C(12X)-H(12X)	0.9500
C(13X)-C(14X)	1.408(15)
C(13X)-C(16X)	1.432(17)
C(14X)-H(14X)	0.9500
C(15X)-F(15D)	1.310(9)
C(15X)-F(15B)	1.315(8)
C(15X)-F(15C)	1.320(9)
C(15X)-F(15A)	1.339(6)
C(15X)-F(15F)	1.341(10)
C(16X)-F(16B)	1.336(16)
C(16X)-F(16A)	1.332(15)
C(16X)-F(16C)	1.336(14)
C(17X)-C(18X)	1.395(15)
C(17X)-C(22X)	1.410(15)
C(18X)-C(19X)	1.378(16)
C(18X)-H(18X)	0.9500
C(19X)-C(20X)	1.379(16)
C(19X)-C(23X)	1.490(16)
C(20X)-C(21X)	1.401(17)
C(20X)-H(20X)	0.9500
C(21X)-C(22X)	1.393(16)
C(21X)-C(24X)	1.496(18)
C(22X)-H(22X)	0.9500
C(23X)-F(23F)	1.316(9)
C(23X)-F(23B)	1.321(9)
C(23X)-F(23E)	1.321(9)
C(23X)-F(23C)	1.325(9)
C(23X)-F(23D)	1.328(9)
C(23X)-F(23A)	1.331(6)
C(24X)-F(24B)	1.297(16)
C(24X)-F(24A)	1.317(16)
C(24X)-F(24C)	1.371(16)
C(25X)-C(26X)	1.376(15)
C(25X)-C(30X)	1.387(14)
C(26X)-C(27X)	1.383(16)
C(26X)-H(26X)	0.9500
C(27X)-C(28X)	1.393(16)
C(27X)-C(31X)	1.502(18)
C(28X)-C(29X)	1.391(16)
C(28X)-H(28X)	0.9500

C(29X)-C(30X)	1.400(15)
C(29X)-C(32X)	1.480(16)
C(30X)-H(30X)	0.9500
C(31X)-F(31E)	1.321(10)
C(31X)-F(31A)	1.322(7)
C(31X)-F(31C)	1.323(10)
C(31X)-F(31F)	1.325(10)
C(31X)-F(31B)	1.327(9)
C(31X)-F(31D)	1.336(10)
C(32X)-F(32F)	1.328(10)
C(32X)-F(32C)	1.328(9)
C(32X)-F(32E)	1.329(10)
C(32X)-F(32A)	1.330(6)
C(32X)-F(32D)	1.332(10)
C(32X)-F(32B)	1.333(9)
B(2X)-C(33X)	1.613(16)
B(2X)-C(41X)	1.640(14)
B(2X)-C(49X)	1.647(14)
B(2X)-C(57X)	1.648(16)
C(33X)-C(38X)	1.394(14)
C(33X)-C(34X)	1.411(14)
C(34X)-C(35X)	1.402(14)
C(34X)-H(34X)	0.9500
C(35X)-C(36X)	1.387(15)
C(35X)-C(39X)	1.491(15)
C(36X)-C(37X)	1.395(15)
C(36X)-H(36X)	0.9500
C(37X)-C(38X)	1.370(14)
C(37X)-C(40X)	1.493(15)
C(38X)-H(38X)	0.9500
C(39X)-F(39B)	1.306(13)
C(39X)-F(39C)	1.325(13)
C(39X)-F(39X)	1.339(14)
C(40X)-F(40A)	1.308(12)
C(40X)-F(40C)	1.337(12)
C(40X)-F(40B)	1.356(12)
C(41X)-C(46X)	1.395(14)
C(41X)-C(42X)	1.409(14)
C(42X)-C(43X)	1.385(15)
C(42X)-H(42X)	0.9500
C(43X)-C(44X)	1.369(16)
C(43X)-C(47X)	1.448(16)
C(44X)-C(45X)	1.384(15)
C(44X)-H(44X)	0.9500
C(45X)-C(46X)	1.388(14)
C(45X)-C(48X)	1.485(16)
C(46X)-H(46X)	0.9500

C(47X)-F(47A)	1.330(6)
C(47X)-F(47B)	1.332(9)
C(47X)-F(47F)	1.339(10)
C(47X)-F(47D)	1.350(9)
C(47X)-F(47C)	1.379(9)
C(47X)-F(47E)	1.380(9)
C(48X)-F(48D)	1.319(10)
C(48X)-F(48C)	1.322(9)
C(48X)-F(48F)	1.324(10)
C(48X)-F(48A)	1.326(6)
C(48X)-F(48B)	1.327(9)
C(48X)-F(48E)	1.327(10)
C(49X)-C(50X)	1.397(14)
C(49X)-C(54X)	1.405(14)
C(50X)-C(51X)	1.371(14)
C(50X)-H(50X)	0.9500
C(51X)-C(52X)	1.404(14)
C(51X)-C(55X)	1.487(15)
C(52X)-C(53X)	1.393(14)
C(52X)-H(52X)	0.9500
C(53X)-C(54X)	1.382(14)
C(53X)-C(56X)	1.483(15)
C(54X)-H(54X)	0.9500
C(55X)-F(55E)	1.26(3)
C(55X)-F(55B)	1.28(2)
C(55X)-F(55A)	1.326(18)
C(55X)-F(55D)	1.33(3)
C(55X)-F(55F)	1.33(3)
C(55X)-F(55C)	1.39(2)
C(56X)-F(56B)	1.284(13)
C(56X)-F(56A)	1.297(13)
C(56X)-F(56C)	1.327(13)
C(57X)-C(62X)	1.388(14)
C(57X)-C(58X)	1.393(15)
C(58X)-C(59X)	1.389(15)
C(58X)-H(58X)	0.9500
C(59X)-C(60X)	1.379(16)
C(59X)-C(63X)	1.499(18)
C(60X)-C(61X)	1.380(16)
C(60X)-H(60X)	0.9500
C(61X)-C(62X)	1.375(14)
C(61X)-C(64X)	1.465(16)
C(62X)-H(62X)	0.9500
C(63X)-F(63F)	1.28(4)
C(63X)-F(63B)	1.29(2)
C(63X)-F(63E)	1.29(5)
C(63X)-F(63A)	1.30(2)

C(63X)-F(63C)	1.39(2)
C(63X)-F(63D)	1.44(6)
C(64X)-F(64C)	1.279(15)
C(64X)-F(64A)	1.337(14)
C(64X)-F(64B)	1.363(15)

C(1)-Rh(1)-C(15)	84.3(4)
C(1)-Rh(1)-C(14)	86.9(4)
C(15)-Rh(1)-C(14)	38.1(4)
C(1)-Rh(1)-C(19)	161.1(4)
C(15)-Rh(1)-C(19)	94.9(4)
C(14)-Rh(1)-C(19)	81.1(4)
C(1)-Rh(1)-C(18)	160.5(3)
C(15)-Rh(1)-C(18)	80.2(4)
C(14)-Rh(1)-C(18)	88.1(4)
C(19)-Rh(1)-C(18)	34.0(4)
C(1)-Rh(1)-S(2)	92.4(3)
C(15)-Rh(1)-S(2)	167.2(3)
C(14)-Rh(1)-S(2)	154.2(3)
C(19)-Rh(1)-S(2)	92.2(3)
C(18)-Rh(1)-S(2)	100.3(2)
C(22)-Rh(2)-C(35)	90.6(4)
C(22)-Rh(2)-C(36)	93.1(4)
C(35)-Rh(2)-C(36)	38.4(4)
C(22)-Rh(2)-C(40)	162.4(4)
C(35)-Rh(2)-C(40)	84.8(5)
C(36)-Rh(2)-C(40)	93.8(5)
C(22)-Rh(2)-C(39)	161.0(4)
C(35)-Rh(2)-C(39)	97.0(4)
C(36)-Rh(2)-C(39)	82.3(4)
C(40)-Rh(2)-C(39)	36.5(4)
C(22)-Rh(2)-S(1)	85.6(3)
C(35)-Rh(2)-S(1)	166.1(3)
C(36)-Rh(2)-S(1)	155.1(3)
C(40)-Rh(2)-S(1)	94.8(4)
C(39)-Rh(2)-S(1)	90.8(3)
C(5)-S(1)-C(4)	90.8(5)
C(5)-S(1)-Rh(2)	108.7(3)
C(4)-S(1)-Rh(2)	115.2(3)
C(26)-S(2)-C(25)	90.2(5)
C(26)-S(2)-Rh(1)	102.8(4)
C(25)-S(2)-Rh(1)	111.5(3)
O(3)-Si(1)-O(1)	108.5(7)
O(3)-Si(1)-O(2)	109.1(8)
O(1)-Si(1)-O(2)	110.1(7)
O(3)-Si(1)-C(10)	111.9(8)

O(1)-Si(1)-C(10)	111.1(7)
O(2)-Si(1)-C(10)	106.1(7)
N(2)-C(1)-N(1)	108.8(9)
N(2)-C(1)-Rh(1)	127.8(7)
N(1)-C(1)-Rh(1)	122.0(7)
C(1)-N(1)-C(4)	128.0(9)
C(1)-N(1)-C(2)	108.5(9)
C(4)-N(1)-C(2)	123.5(9)
C(13)-O(1)-Si(1)	121.0(11)
C(1)-N(2)-C(3)	108.4(9)
C(1)-N(2)-C(8)	127.5(9)
C(3)-N(2)-C(8)	123.8(9)
C(3)-C(2)-N(1)	104.6(9)
C(3)-C(2)-H(2)	127.7
N(1)-C(2)-H(2)	127.7
C(12)-O(2)-Si(1)	119.4(14)
C(25)-N(3)-C(23)	124.1(8)
C(25)-N(3)-C(22)	125.4(8)
C(23)-N(3)-C(22)	110.2(8)
C(2)-C(3)-N(2)	108.7(10)
C(2)-C(3)-H(3)	125.6
N(2)-C(3)-H(3)	125.6
C(11)-O(3)-Si(1)	130.4(14)
C(22)-N(4)-C(24)	111.9(9)
C(22)-N(4)-C(29)	122.9(9)
C(24)-N(4)-C(29)	125.2(9)
C(7)-C(4)-N(1)	129.8(9)
C(7)-C(4)-S(1)	111.1(8)
N(1)-C(4)-S(1)	119.1(7)
C(6)-C(5)-S(1)	111.8(9)
C(6)-C(5)-H(5)	124.1
S(1)-C(5)-H(5)	124.1
C(5)-C(6)-C(7)	113.6(10)
C(5)-C(6)-H(6)	123.2
C(7)-C(6)-H(6)	123.2
C(4)-C(7)-C(6)	112.6(10)
C(4)-C(7)-H(7)	123.7
C(6)-C(7)-H(7)	123.7
N(2)-C(8)-C(9)	114.7(10)
N(2)-C(8)-H(8A)	108.6
C(9)-C(8)-H(8A)	108.6
N(2)-C(8)-H(8B)	108.6
C(9)-C(8)-H(8B)	108.6
H(8A)-C(8)-H(8B)	107.6
C(8)-C(9)-C(10)	111.7(11)
C(8)-C(9)-H(9A)	109.3
C(10)-C(9)-H(9A)	109.3

C(8)-C(9)-H(9B)	109.3
C(10)-C(9)-H(9B)	109.3
H(9A)-C(9)-H(9B)	107.9
C(9)-C(10)-Si(1)	118.5(10)
C(9)-C(10)-H(10A)	107.7
Si(1)-C(10)-H(10A)	107.7
C(9)-C(10)-H(10B)	107.7
Si(1)-C(10)-H(10B)	107.7
H(10A)-C(10)-H(10B)	107.1
O(3)-C(11)-H(11A)	109.5
O(3)-C(11)-H(11B)	109.5
H(11A)-C(11)-H(11B)	109.5
O(3)-C(11)-H(11C)	109.5
H(11A)-C(11)-H(11C)	109.5
H(11B)-C(11)-H(11C)	109.5
O(2)-C(12)-H(12A)	109.5
O(2)-C(12)-H(12B)	109.5
H(12A)-C(12)-H(12B)	109.5
O(2)-C(12)-H(12C)	109.5
H(12A)-C(12)-H(12C)	109.5
H(12B)-C(12)-H(12C)	109.5
O(1)-C(13)-H(13A)	109.5
O(1)-C(13)-H(13B)	109.5
H(13A)-C(13)-H(13B)	109.5
O(1)-C(13)-H(13C)	109.5
H(13A)-C(13)-H(13C)	109.5
H(13B)-C(13)-H(13C)	109.5
C(15)-C(14)-C(21)	123.6(12)
C(15)-C(14)-Rh(1)	70.5(6)
C(21)-C(14)-Rh(1)	111.3(7)
C(15)-C(14)-H(14)	118.2
C(21)-C(14)-H(14)	118.2
Rh(1)-C(14)-H(14)	88.2
C(14)-C(15)-C(16)	127.2(11)
C(14)-C(15)-Rh(1)	71.3(6)
C(16)-C(15)-Rh(1)	108.8(7)
C(14)-C(15)-H(15)	116.4
C(16)-C(15)-H(15)	116.4
Rh(1)-C(15)-H(15)	89.9
C(17)-C(16)-C(15)	115.0(10)
C(17)-C(16)-H(16A)	108.5
C(15)-C(16)-H(16A)	108.5
C(17)-C(16)-H(16B)	108.5
C(15)-C(16)-H(16B)	108.5
H(16A)-C(16)-H(16B)	107.5
C(18)-C(17)-C(16)	113.6(9)
C(18)-C(17)-H(17A)	108.8

C(16)-C(17)-H(17A)	108.8
C(18)-C(17)-H(17B)	108.8
C(16)-C(17)-H(17B)	108.8
H(17A)-C(17)-H(17B)	107.7
C(19)-C(18)-C(17)	128.1(11)
C(19)-C(18)-Rh(1)	70.5(6)
C(17)-C(18)-Rh(1)	111.5(7)
C(19)-C(18)-H(18)	115.9
C(17)-C(18)-H(18)	115.9
Rh(1)-C(18)-H(18)	87.8
C(18)-C(19)-C(20)	126.4(11)
C(18)-C(19)-Rh(1)	75.5(6)
C(20)-C(19)-Rh(1)	108.7(8)
C(18)-C(19)-H(19)	116.8
C(20)-C(19)-H(19)	116.8
Rh(1)-C(19)-H(19)	85.6
C(21)-C(20)-C(19)	113.4(11)
C(21)-C(20)-H(20A)	108.9
C(19)-C(20)-H(20A)	108.9
C(21)-C(20)-H(20B)	108.9
C(19)-C(20)-H(20B)	108.9
H(20A)-C(20)-H(20B)	107.7
C(20)-C(21)-C(14)	115.4(11)
C(20)-C(21)-H(21A)	108.4
C(14)-C(21)-H(21A)	108.4
C(20)-C(21)-H(21B)	108.4
C(14)-C(21)-H(21B)	108.4
H(21A)-C(21)-H(21B)	107.5
N(4)-C(22)-N(3)	102.7(8)
N(4)-C(22)-Rh(2)	131.5(7)
N(3)-C(22)-Rh(2)	124.7(7)
C(24)-C(23)-N(3)	107.7(9)
C(24)-C(23)-H(23)	126.1
N(3)-C(23)-H(23)	126.1
C(23)-C(24)-N(4)	107.2(9)
C(23)-C(24)-H(24)	126.4
N(4)-C(24)-H(24)	126.4
C(28)-C(25)-N(3)	128.4(9)
C(28)-C(25)-S(2)	111.8(8)
N(3)-C(25)-S(2)	119.8(7)
C(27)-C(26)-S(2)	111.3(9)
C(27)-C(26)-H(26)	124.4
S(2)-C(26)-H(26)	124.4
C(26)-C(27)-C(28)	113.3(10)
C(26)-C(27)-H(27)	123.3
C(28)-C(27)-H(27)	123.3
C(25)-C(28)-C(27)	113.2(9)

C(25)-C(28)-H(28)	123.4
C(27)-C(28)-H(28)	123.4
N(4)-C(29)-C(30)	116.9(12)
N(4)-C(29)-C(30A)	121.0(12)
N(4)-C(29)-H(29A)	107.9
C(30)-C(29)-H(29A)	108.0
N(4)-C(29)-H(29B)	109.8
C(30)-C(29)-H(29B)	108.8
H(29A)-C(29)-H(29B)	105.0
N(4)-C(29)-H(29C)	108.9
C(30A)-C(29)-H(29C)	107.5
N(4)-C(29)-H(29D)	106.8
C(30A)-C(29)-H(29D)	106.6
H(29C)-C(29)-H(29D)	105.0
C(29)-C(30)-C(31)	110.8(17)
C(29)-C(30)-H(30A)	109.5
C(31)-C(30)-H(30A)	109.5
C(29)-C(30)-H(30B)	109.5
C(31)-C(30)-H(30B)	109.5
H(30A)-C(30)-H(30B)	108.1
C(30)-C(31)-Si(2)	120.5(15)
C(30)-C(31)-H(31A)	107.2
Si(2)-C(31)-H(31A)	107.2
C(30)-C(31)-H(31B)	107.2
Si(2)-C(31)-H(31B)	107.2
H(31A)-C(31)-H(31B)	106.8
O(4)-Si(2)-O(6)	109.9(7)
O(4)-Si(2)-O(5)	108.0(7)
O(6)-Si(2)-O(5)	108.0(8)
O(4)-Si(2)-C(31)	112.4(10)
O(6)-Si(2)-C(31)	103.6(11)
O(5)-Si(2)-C(31)	114.8(11)
C(32)-O(4)-Si(2)	127.6(13)
C(33)-O(5)-Si(2)	126.7(12)
C(34)-O(6)-Si(2)	127.4(13)
O(4)-C(32)-H(32A)	109.5
O(4)-C(32)-H(32B)	109.5
H(32A)-C(32)-H(32B)	109.5
O(4)-C(32)-H(32C)	109.5
H(32A)-C(32)-H(32C)	109.5
H(32B)-C(32)-H(32C)	109.5
O(5)-C(33)-H(33A)	109.5
O(5)-C(33)-H(33B)	109.5
H(33A)-C(33)-H(33B)	109.5
O(5)-C(33)-H(33C)	109.5
H(33A)-C(33)-H(33C)	109.5
H(33B)-C(33)-H(33C)	109.5

O(6)-C(34)-H(34A)	109.5
O(6)-C(34)-H(34B)	109.5
H(34A)-C(34)-H(34B)	109.5
O(6)-C(34)-H(34C)	109.5
H(34A)-C(34)-H(34C)	109.5
H(34B)-C(34)-H(34C)	109.5
C(31A)-C(30A)-C(29)	112.9(18)
C(31A)-C(30A)-H(30C)	109.0
C(29)-C(30A)-H(30C)	109.0
C(31A)-C(30A)-H(30D)	109.0
C(29)-C(30A)-H(30D)	109.0
H(30C)-C(30A)-H(30D)	107.8
C(30A)-C(31A)-Si(2A)	119.9(17)
C(30A)-C(31A)-H(31C)	107.3
Si(2A)-C(31A)-H(31C)	107.3
C(30A)-C(31A)-H(31D)	107.3
Si(2A)-C(31A)-H(31D)	107.3
H(31C)-C(31A)-H(31D)	106.9
O(5A)-Si(2A)-O(6A)	110.9(8)
O(5A)-Si(2A)-O(4A)	108.2(7)
O(6A)-Si(2A)-O(4A)	110.2(8)
O(5A)-Si(2A)-C(31A)	104.2(12)
O(6A)-Si(2A)-C(31A)	110.4(13)
O(4A)-Si(2A)-C(31A)	112.8(11)
C(32A)-O(4A)-Si(2A)	125.7(13)
C(33A)-O(5A)-Si(2A)	122.1(13)
C(34A)-O(6A)-Si(2A)	124.6(14)
O(4A)-C(32A)-H(32D)	109.5
O(4A)-C(32A)-H(32E)	109.5
H(32D)-C(32A)-H(32E)	109.5
O(4A)-C(32A)-H(32F)	109.5
H(32D)-C(32A)-H(32F)	109.5
H(32E)-C(32A)-H(32F)	109.5
O(5A)-C(33A)-H(33D)	109.5
O(5A)-C(33A)-H(33E)	109.5
H(33D)-C(33A)-H(33E)	109.5
O(5A)-C(33A)-H(33F)	109.5
H(33D)-C(33A)-H(33F)	109.5
H(33E)-C(33A)-H(33F)	109.5
O(6A)-C(34A)-H(34D)	109.5
O(6A)-C(34A)-H(34E)	109.5
H(34D)-C(34A)-H(34E)	109.5
O(6A)-C(34A)-H(34F)	109.5
H(34D)-C(34A)-H(34F)	109.5
H(34E)-C(34A)-H(34F)	109.5
C(36)-C(35)-C(42)	124.5(10)
C(36)-C(35)-Rh(2)	71.0(6)

C(42)-C(35)-Rh(2)	110.0(7)
C(36)-C(35)-H(35)	117.7
C(42)-C(35)-H(35)	117.7
Rh(2)-C(35)-H(35)	89.0
C(35)-C(36)-C(37)	125.3(11)
C(35)-C(36)-Rh(2)	70.6(6)
C(37)-C(36)-Rh(2)	112.3(7)
C(35)-C(36)-H(36)	117.3
C(37)-C(36)-H(36)	117.3
Rh(2)-C(36)-H(36)	87.1
C(38)-C(37)-C(36)	113.3(9)
C(38)-C(37)-H(37A)	108.9
C(36)-C(37)-H(37A)	108.9
C(38)-C(37)-H(37B)	108.9
C(36)-C(37)-H(37B)	108.9
H(37A)-C(37)-H(37B)	107.7
C(37)-C(38)-C(39)	118.6(10)
C(37)-C(38)-H(38A)	107.7
C(39)-C(38)-H(38A)	107.7
C(37)-C(38)-H(38B)	107.7
C(39)-C(38)-H(38B)	107.7
H(38A)-C(38)-H(38B)	107.1
C(40)-C(39)-C(38)	132.3(11)
C(40)-C(39)-Rh(2)	70.4(7)
C(38)-C(39)-Rh(2)	101.6(7)
C(40)-C(39)-H(39)	113.9
C(38)-C(39)-H(39)	113.9
Rh(2)-C(39)-H(39)	99.6
C(39)-C(40)-C(41)	112.3(11)
C(39)-C(40)-Rh(2)	73.1(7)
C(41)-C(40)-Rh(2)	104.6(8)
C(39)-C(40)-H(40)	123.9
C(41)-C(40)-H(40)	123.9
Rh(2)-C(40)-H(40)	92.0
C(42)-C(41)-C(40)	116.5(11)
C(42)-C(41)-H(41A)	108.2
C(40)-C(41)-H(41A)	108.2
C(42)-C(41)-H(41B)	108.2
C(40)-C(41)-H(41B)	108.2
H(41A)-C(41)-H(41B)	107.3
C(35)-C(42)-C(41)	113.6(10)
C(35)-C(42)-H(42A)	108.8
C(41)-C(42)-H(42A)	108.8
C(35)-C(42)-H(42B)	108.8
C(41)-C(42)-H(42B)	108.8
H(42A)-C(42)-H(42B)	107.7
C(9X)-B(1X)-C(17X)	114.1(9)

C(9X)-B(1X)-C(25X)	102.6(8)
C(17X)-B(1X)-C(25X)	111.9(8)
C(9X)-B(1X)-C(1X)	110.9(8)
C(17X)-B(1X)-C(1X)	104.1(9)
C(25X)-B(1X)-C(1X)	113.7(9)
C(6X)-C(1X)-C(2X)	115.9(10)
C(6X)-C(1X)-B(1X)	120.0(10)
C(2X)-C(1X)-B(1X)	123.9(10)
C(1X)-C(2X)-C(3X)	121.2(12)
C(1X)-C(2X)-H(2X)	119.4
C(3X)-C(2X)-H(2X)	119.4
C(4X)-C(3X)-C(2X)	121.3(12)
C(4X)-C(3X)-C(7X)	119.6(11)
C(2X)-C(3X)-C(7X)	119.2(12)
C(3X)-C(4X)-C(5X)	119.4(11)
C(3X)-C(4X)-H(4X)	120.3
C(5X)-C(4X)-H(4X)	120.3
C(4X)-C(5X)-C(6X)	118.7(12)
C(4X)-C(5X)-C(8X)	123.0(10)
C(6X)-C(5X)-C(8X)	118.2(11)
C(1X)-C(6X)-C(5X)	123.5(12)
C(1X)-C(6X)-H(6X)	118.3
C(5X)-C(6X)-H(6X)	118.3
F(7D)-C(7X)-F(7E)	107(3)
F(7C)-C(7X)-F(7A)	102.9(14)
F(7D)-C(7X)-F(7F)	91(3)
F(7E)-C(7X)-F(7F)	110(4)
F(7C)-C(7X)-F(7B)	107.3(16)
F(7A)-C(7X)-F(7B)	105.6(13)
F(7D)-C(7X)-C(3X)	115(2)
F(7E)-C(7X)-C(3X)	116(2)
F(7C)-C(7X)-C(3X)	114.2(13)
F(7A)-C(7X)-C(3X)	113.9(11)
F(7F)-C(7X)-C(3X)	115(3)
F(7B)-C(7X)-C(3X)	112.2(11)
F(8D)-C(8X)-F(8E)	107(3)
F(8C)-C(8X)-F(8A)	105.6(12)
F(8D)-C(8X)-F(8F)	96(3)
F(8E)-C(8X)-F(8F)	102(3)
F(8C)-C(8X)-F(8B)	104.2(13)
F(8A)-C(8X)-F(8B)	106.6(13)
F(8D)-C(8X)-C(5X)	119.2(18)
F(8C)-C(8X)-C(5X)	112.4(11)
F(8E)-C(8X)-C(5X)	118(2)
F(8A)-C(8X)-C(5X)	113.3(11)
F(8F)-C(8X)-C(5X)	112(3)
F(8B)-C(8X)-C(5X)	113.9(10)

C(10X)-C(9X)-C(14X)	116.1(10)
C(10X)-C(9X)-B(1X)	122.4(10)
C(14X)-C(9X)-B(1X)	121.1(9)
C(11X)-C(10X)-C(9X)	122.0(11)
C(11X)-C(10X)-H(10X)	119.0
C(9X)-C(10X)-H(10X)	119.0
C(10X)-C(11X)-C(12X)	121.4(10)
C(10X)-C(11X)-C(15X)	120.1(10)
C(12X)-C(11X)-C(15X)	118.5(10)
C(13X)-C(12X)-C(11X)	118.2(10)
C(13X)-C(12X)-H(12X)	120.9
C(11X)-C(12X)-H(12X)	120.9
C(12X)-C(13X)-C(14X)	120.7(10)
C(12X)-C(13X)-C(16X)	121.6(10)
C(14X)-C(13X)-C(16X)	117.7(10)
C(9X)-C(14X)-C(13X)	121.4(10)
C(9X)-C(14X)-H(14X)	119.3
C(13X)-C(14X)-H(14X)	119.3
F(15B)-C(15X)-F(15C)	116.3(16)
F(15B)-C(15X)-F(15A)	93.8(10)
F(15C)-C(15X)-F(15A)	102.0(15)
F(15D)-C(15X)-F(15F)	97.3(18)
F(15D)-C(15X)-C(11X)	116.6(13)
F(15B)-C(15X)-C(11X)	115.6(8)
F(15C)-C(15X)-C(11X)	115.2(11)
F(15A)-C(15X)-C(11X)	110.4(11)
F(15F)-C(15X)-C(11X)	108.7(12)
F(16B)-C(16X)-F(16A)	101.9(11)
F(16B)-C(16X)-F(16C)	101.9(11)
F(16A)-C(16X)-F(16C)	105.9(12)
F(16B)-C(16X)-C(13X)	114.3(11)
F(16A)-C(16X)-C(13X)	116.3(11)
F(16C)-C(16X)-C(13X)	114.9(11)
C(18X)-C(17X)-C(22X)	115.0(10)
C(18X)-C(17X)-B(1X)	124.7(10)
C(22X)-C(17X)-B(1X)	120.0(10)
C(19X)-C(18X)-C(17X)	122.9(11)
C(19X)-C(18X)-H(18X)	118.5
C(17X)-C(18X)-H(18X)	118.5
C(18X)-C(19X)-C(20X)	122.0(11)
C(18X)-C(19X)-C(23X)	119.7(10)
C(20X)-C(19X)-C(23X)	118.3(10)
C(19X)-C(20X)-C(21X)	116.9(11)
C(19X)-C(20X)-H(20X)	121.5
C(21X)-C(20X)-H(20X)	121.5
C(22X)-C(21X)-C(20X)	120.9(11)
C(22X)-C(21X)-C(24X)	121.1(11)

C(20X)-C(21X)-C(24X)	118.0(11)
C(21X)-C(22X)-C(17X)	122.3(11)
C(21X)-C(22X)-H(22X)	118.8
C(17X)-C(22X)-H(22X)	118.8
F(23F)-C(23X)-F(23E)	108.3(13)
F(23B)-C(23X)-F(23C)	104.6(11)
F(23F)-C(23X)-F(23D)	106.8(12)
F(23E)-C(23X)-F(23D)	106.0(11)
F(23B)-C(23X)-F(23A)	104.0(9)
F(23C)-C(23X)-F(23A)	102.0(10)
F(23F)-C(23X)-C(19X)	115.0(14)
F(23B)-C(23X)-C(19X)	115.4(10)
F(23E)-C(23X)-C(19X)	110.0(15)
F(23C)-C(23X)-C(19X)	114.6(10)
F(23D)-C(23X)-C(19X)	110.2(13)
F(23A)-C(23X)-C(19X)	114.7(10)
F(24B)-C(24X)-F(24A)	109.0(13)
F(24B)-C(24X)-F(24C)	106.7(12)
F(24A)-C(24X)-F(24C)	103.9(11)
F(24B)-C(24X)-C(21X)	113.7(11)
F(24A)-C(24X)-C(21X)	113.0(11)
F(24C)-C(24X)-C(21X)	110.0(12)
C(26X)-C(25X)-C(30X)	115.0(10)
C(26X)-C(25X)-B(1X)	123.4(9)
C(30X)-C(25X)-B(1X)	120.9(9)
C(25X)-C(26X)-C(27X)	123.8(11)
C(25X)-C(26X)-H(26X)	118.1
C(27X)-C(26X)-H(26X)	118.1
C(26X)-C(27X)-C(28X)	120.2(11)
C(26X)-C(27X)-C(31X)	121.0(11)
C(28X)-C(27X)-C(31X)	118.8(10)
C(29X)-C(28X)-C(27X)	118.3(11)
C(29X)-C(28X)-H(28X)	120.9
C(27X)-C(28X)-H(28X)	120.9
C(28X)-C(29X)-C(30X)	119.1(10)
C(28X)-C(29X)-C(32X)	118.5(10)
C(30X)-C(29X)-C(32X)	122.4(10)
C(25X)-C(30X)-C(29X)	123.7(11)
C(25X)-C(30X)-H(30X)	118.2
C(29X)-C(30X)-H(30X)	118.2
F(31A)-C(31X)-F(31C)	117(2)
F(31E)-C(31X)-F(31F)	113(2)
F(31A)-C(31X)-F(31B)	99.3(14)
F(31C)-C(31X)-F(31B)	100.4(18)
F(31E)-C(31X)-F(31D)	96.4(17)
F(31F)-C(31X)-F(31D)	106(2)
F(31E)-C(31X)-C(27X)	117.2(14)

F(31A)-C(31X)-C(27X)	114.4(12)
F(31C)-C(31X)-C(27X)	112.3(16)
F(31F)-C(31X)-C(27X)	112(2)
F(31B)-C(31X)-C(27X)	111.1(11)
F(31D)-C(31X)-C(27X)	110.8(12)
F(32F)-C(32X)-F(32E)	124(4)
F(32C)-C(32X)-F(32A)	102.6(13)
F(32F)-C(32X)-F(32D)	103(4)
F(32E)-C(32X)-F(32D)	96(3)
F(32C)-C(32X)-F(32B)	108.9(15)
F(32A)-C(32X)-F(32B)	104.5(13)
F(32F)-C(32X)-C(29X)	114(4)
F(32C)-C(32X)-C(29X)	113.0(11)
F(32E)-C(32X)-C(29X)	111.8(18)
F(32A)-C(32X)-C(29X)	114.1(12)
F(32D)-C(32X)-C(29X)	103(3)
F(32B)-C(32X)-C(29X)	113.1(10)
C(33X)-B(2X)-C(41X)	110.6(8)
C(33X)-B(2X)-C(49X)	105.5(8)
C(41X)-B(2X)-C(49X)	110.0(8)
C(33X)-B(2X)-C(57X)	113.6(9)
C(41X)-B(2X)-C(57X)	103.1(8)
C(49X)-B(2X)-C(57X)	114.1(8)
C(38X)-C(33X)-C(34X)	114.3(9)
C(38X)-C(33X)-B(2X)	125.0(9)
C(34X)-C(33X)-B(2X)	120.5(9)
C(35X)-C(34X)-C(33X)	122.7(10)
C(35X)-C(34X)-H(34X)	118.6
C(33X)-C(34X)-H(34X)	118.6
C(36X)-C(35X)-C(34X)	119.9(9)
C(36X)-C(35X)-C(39X)	121.4(10)
C(34X)-C(35X)-C(39X)	118.8(10)
C(35X)-C(36X)-C(37X)	118.7(10)
C(35X)-C(36X)-H(36X)	120.7
C(37X)-C(36X)-H(36X)	120.7
C(38X)-C(37X)-C(36X)	119.9(10)
C(38X)-C(37X)-C(40X)	120.5(10)
C(36X)-C(37X)-C(40X)	119.6(9)
C(37X)-C(38X)-C(33X)	124.5(10)
C(37X)-C(38X)-H(38X)	117.7
C(33X)-C(38X)-H(38X)	117.7
F(39B)-C(39X)-F(39C)	108.1(10)
F(39B)-C(39X)-F(39X)	105.3(10)
F(39C)-C(39X)-F(39X)	104.7(10)
F(39B)-C(39X)-C(35X)	114.1(10)
F(39C)-C(39X)-C(35X)	112.5(10)
F(39X)-C(39X)-C(35X)	111.5(9)

F(40A)-C(40X)-F(40C)	106.5(9)
F(40A)-C(40X)-F(40B)	106.9(9)
F(40C)-C(40X)-F(40B)	105.2(9)
F(40A)-C(40X)-C(37X)	113.6(9)
F(40C)-C(40X)-C(37X)	111.7(9)
F(40B)-C(40X)-C(37X)	112.4(9)
C(46X)-C(41X)-C(42X)	115.0(9)
C(46X)-C(41X)-B(2X)	125.1(9)
C(42X)-C(41X)-B(2X)	119.8(9)
C(43X)-C(42X)-C(41X)	122.2(10)
C(43X)-C(42X)-H(42X)	118.9
C(41X)-C(42X)-H(42X)	118.9
C(44X)-C(43X)-C(42X)	121.1(10)
C(44X)-C(43X)-C(47X)	117.4(9)
C(42X)-C(43X)-C(47X)	121.5(10)
C(43X)-C(44X)-C(45X)	118.5(10)
C(43X)-C(44X)-H(44X)	120.8
C(45X)-C(44X)-H(44X)	120.8
C(44X)-C(45X)-C(46X)	120.3(10)
C(44X)-C(45X)-C(48X)	118.5(9)
C(46X)-C(45X)-C(48X)	121.2(10)
C(45X)-C(46X)-C(41X)	122.9(10)
C(45X)-C(46X)-H(46X)	118.6
C(41X)-C(46X)-H(46X)	118.6
F(47A)-C(47X)-F(47B)	109.4(10)
F(47F)-C(47X)-F(47D)	102.5(14)
F(47A)-C(47X)-F(47C)	101.2(9)
F(47B)-C(47X)-F(47C)	100.6(9)
F(47F)-C(47X)-F(47E)	98.1(13)
F(47D)-C(47X)-F(47E)	96.7(10)
F(47A)-C(47X)-C(43X)	116.8(11)
F(47B)-C(47X)-C(43X)	116.4(9)
F(47F)-C(47X)-C(43X)	128.8(17)
F(47D)-C(47X)-C(43X)	117.1(14)
F(47C)-C(47X)-C(43X)	110.2(9)
F(47E)-C(47X)-C(43X)	107.5(13)
F(48D)-C(48X)-F(48F)	107(2)
F(48C)-C(48X)-F(48A)	106.6(15)
F(48C)-C(48X)-F(48B)	103.9(15)
F(48A)-C(48X)-F(48B)	106.4(15)
F(48D)-C(48X)-F(48E)	106(2)
F(48F)-C(48X)-F(48E)	100(2)
F(48D)-C(48X)-C(45X)	118.6(16)
F(48C)-C(48X)-C(45X)	113.6(11)
F(48F)-C(48X)-C(45X)	114(2)
F(48A)-C(48X)-C(45X)	113.6(12)
F(48B)-C(48X)-C(45X)	111.9(11)

F(48E)-C(48X)-C(45X)	109.2(17)
C(50X)-C(49X)-C(54X)	116.3(9)
C(50X)-C(49X)-B(2X)	118.4(9)
C(54X)-C(49X)-B(2X)	125.0(9)
C(51X)-C(50X)-C(49X)	122.8(10)
C(51X)-C(50X)-H(50X)	118.6
C(49X)-C(50X)-H(50X)	118.6
C(50X)-C(51X)-C(52X)	120.5(9)
C(50X)-C(51X)-C(55X)	119.9(10)
C(52X)-C(51X)-C(55X)	119.5(9)
C(53X)-C(52X)-C(51X)	117.4(9)
C(53X)-C(52X)-H(52X)	121.3
C(51X)-C(52X)-H(52X)	121.3
C(54X)-C(53X)-C(52X)	121.5(10)
C(54X)-C(53X)-C(56X)	119.8(9)
C(52X)-C(53X)-C(56X)	118.6(9)
C(53X)-C(54X)-C(49X)	121.2(9)
C(53X)-C(54X)-H(54X)	119.4
C(49X)-C(54X)-H(54X)	119.4
F(55B)-C(55X)-F(55A)	109.3(15)
F(55E)-C(55X)-F(55D)	105.3(18)
F(55E)-C(55X)-F(55F)	115(2)
F(55D)-C(55X)-F(55F)	101.2(18)
F(55B)-C(55X)-F(55C)	101.8(17)
F(55A)-C(55X)-F(55C)	101.4(15)
F(55E)-C(55X)-C(51X)	112.5(14)
F(55B)-C(55X)-C(51X)	115.9(13)
F(55A)-C(55X)-C(51X)	115.3(10)
F(55D)-C(55X)-C(51X)	112.6(15)
F(55F)-C(55X)-C(51X)	109.5(13)
F(55C)-C(55X)-C(51X)	111.3(11)
F(56B)-C(56X)-F(56A)	107.1(10)
F(56B)-C(56X)-F(56C)	104.3(11)
F(56A)-C(56X)-F(56C)	101.3(11)
F(56B)-C(56X)-C(53X)	115.7(10)
F(56A)-C(56X)-C(53X)	114.6(9)
F(56C)-C(56X)-C(53X)	112.4(9)
C(62X)-C(57X)-C(58X)	114.8(10)
C(62X)-C(57X)-B(2X)	121.3(9)
C(58X)-C(57X)-B(2X)	123.6(9)
C(59X)-C(58X)-C(57X)	122.1(10)
C(59X)-C(58X)-H(58X)	119.0
C(57X)-C(58X)-H(58X)	119.0
C(60X)-C(59X)-C(58X)	121.1(10)
C(60X)-C(59X)-C(63X)	119.1(11)
C(58X)-C(59X)-C(63X)	119.8(11)
C(61X)-C(60X)-C(59X)	118.0(10)

C(61X)-C(60X)-H(60X)	121.0
C(59X)-C(60X)-H(60X)	121.0
C(62X)-C(61X)-C(60X)	120.0(10)
C(62X)-C(61X)-C(64X)	121.5(10)
C(60X)-C(61X)-C(64X)	118.5(10)
C(61X)-C(62X)-C(57X)	124.0(10)
C(61X)-C(62X)-H(62X)	118.0
C(57X)-C(62X)-H(62X)	118.0
F(63F)-C(63X)-F(63E)	105(3)
F(63B)-C(63X)-F(63A)	106.9(18)
F(63B)-C(63X)-F(63C)	103.2(17)
F(63A)-C(63X)-F(63C)	107.2(17)
F(63F)-C(63X)-F(63D)	99(3)
F(63E)-C(63X)-F(63D)	117(4)
F(63F)-C(63X)-C(59X)	114(2)
F(63B)-C(63X)-C(59X)	115.9(14)
F(63E)-C(63X)-C(59X)	114(3)
F(63A)-C(63X)-C(59X)	114.5(14)
F(63C)-C(63X)-C(59X)	108.3(14)
F(63D)-C(63X)-C(59X)	108(3)
F(64C)-C(64X)-F(64A)	108.2(11)
F(64C)-C(64X)-F(64B)	103.4(12)
F(64A)-C(64X)-F(64B)	102.4(10)
F(64C)-C(64X)-C(61X)	113.9(11)
F(64A)-C(64X)-C(61X)	114.7(10)
F(64B)-C(64X)-C(61X)	113.0(10)

Table S12. Anisotropic displacement parameters ($Å^2 \times 10^3$) for **Rh7**. The anisotropic displacement factor exponent takes the form: $-2\Pi^2[h^2a^{*2}U_{11} + ... + 2hka^*b^*U_{12}]$

	U_{11}	U_{22}	U_{33}	U_{23}	U_{13}	U_{12}
Rh(1)	19(1)	25(1)	50(1)	-7(1)	-4(1)	0(1)
Rh(2)	20(1)	22(1)	51(1)	-7(1)	-5(1)	1(1)
S(1)	24(1)	30(1)	46(2)	-6(1)	-5(1)	-1(1)
S(2)	22(1)	27(1)	49(2)	-7(1)	-1(1)	-2(1)
Si(1)	76(3)	98(3)	65(3)	-34(2)	-20(2)	17(3)
C(1)	12(3)	42(3)	37(3)	-3(3)	-1(2)	-8(3)
N(1)	31(5)	36(5)	37(5)	-7(4)	-2(4)	-1(4)
O(1)	81(8)	125(10)	75(7)	-51(7)	-12(6)	3(7)
N(2)	28(3)	36(3)	39(3)	-4(2)	0(2)	8(2)
C(2)	25(5)	24(5)	66(8)	1(5)	-12(5)	16(4)
O(2)	100(5)	104(4)	101(4)	-23(3)	-11(3)	0(3)
N(3)	25(4)	18(4)	48(5)	-1(4)	-3(4)	2(3)
C(3)	20(5)	57(7)	51(7)	-2(6)	-9(5)	-6(5)
O(3)	175(15)	88(9)	85(9)	-23(7)	-4(9)	-26(9)

N(4)	44(5)	47(6)	35(5)	-11(4)	-8(4)	3(4)
C(4)	26(5)	24(5)	43(6)	-3(4)	-1(4)	6(4)
C(5)	32(6)	37(6)	43(6)	-13(5)	-2(5)	-14(5)
C(6)	36(6)	41(6)	48(7)	-13(5)	8(5)	-4(5)
C(7)	26(5)	31(6)	58(7)	-7(5)	0(5)	-1(4)
C(8)	47(7)	63(8)	39(7)	-10(6)	-2(5)	6(6)
C(9)	44(7)	93(11)	52(8)	-20(7)	-14(6)	28(7)
C(10)	80(11)	90(11)	56(9)	-25(8)	-13(8)	21(9)
C(11)	230(40)	150(30)	120(20)	12(18)	-50(20)	-40(20)
C(12)	163(12)	167(12)	165(12)	-34(6)	-13(6)	2(6)
C(13)	140(20)	210(30)	70(12)	-57(15)	52(13)	-83(18)
C(14)	19(5)	55(7)	57(7)	-11(6)	-1(5)	-12(5)
C(15)	15(5)	32(6)	70(8)	-3(5)	-4(5)	1(4)
C(16)	37(7)	54(8)	67(8)	4(6)	2(6)	-23(6)
C(17)	32(7)	54(8)	90(10)	24(7)	2(6)	-6(6)
C(18)	22(3)	20(3)	37(3)	0(2)	-3(2)	-1(2)
C(19)	40(4)	38(4)	49(4)	-4(3)	-5(3)	-6(3)
C(20)	44(8)	51(8)	127(13)	-49(9)	-15(8)	1(6)
C(21)	43(8)	69(9)	107(12)	-46(9)	-17(8)	5(7)
C(22)	24(3)	22(3)	32(3)	-8(2)	-5(2)	2(2)
C(23)	31(6)	22(5)	50(7)	-4(5)	-6(5)	0(4)
C(24)	44(7)	24(6)	55(7)	1(5)	-5(5)	16(5)
C(25)	21(5)	20(5)	45(6)	-7(4)	-1(4)	-6(4)
C(26)	30(6)	45(6)	44(6)	-12(5)	7(5)	-4(5)
C(27)	35(6)	29(6)	51(7)	-11(5)	8(5)	-7(5)
C(28)	23(5)	29(6)	53(7)	-9(5)	-1(5)	5(4)
C(29)	84(10)	71(9)	48(8)	-20(7)	-24(7)	38(8)
C(30)	66(6)	58(6)	55(6)	-14(5)	-9(5)	0(5)
C(31)	68(4)	59(4)	57(4)	-17(4)	-10(4)	1(4)
Si(2)	71(3)	60(3)	58(2)	-19(2)	-11(3)	3(3)
O(4)	72(4)	65(4)	61(3)	-19(3)	-9(4)	3(3)
O(5)	76(4)	64(3)	63(3)	-19(3)	-8(3)	1(3)
O(6)	76(4)	67(4)	65(4)	-19(4)	-11(4)	0(4)
C(32)	75(7)	75(6)	69(6)	-15(6)	-9(6)	1(7)
C(33)	86(7)	73(7)	72(6)	-18(6)	-11(6)	-2(7)
C(34)	83(8)	87(8)	74(8)	-20(7)	-10(7)	0(8)
C(30A)	69(6)	62(6)	60(6)	-17(5)	-10(6)	-1(6)
C(31A)	69(4)	62(4)	59(4)	-17(4)	-10(4)	1(4)
Si(2A)	74(3)	63(3)	59(3)	-20(2)	-10(3)	4(3)
O(4A)	72(4)	65(4)	61(3)	-20(3)	-9(4)	2(4)
O(5A)	77(4)	65(4)	65(4)	-21(4)	-9(4)	3(4)
O(6A)	74(4)	64(3)	63(3)	-20(3)	-9(3)	2(3)
C(32A)	74(7)	73(6)	69(6)	-16(6)	-6(7)	2(7)
C(33A)	80(7)	67(7)	69(6)	-16(6)	-13(6)	4(6)
C(34A)	78(7)	78(7)	71(7)	-20(6)	-9(7)	-1(7)
C(35)	31(6)	33(6)	62(8)	-9(5)	7(5)	-7(5)
C(36)	18(5)	39(6)	57(7)	-12(5)	-2(5)	0(4)

C(37)	37(6)	23(5)	73(8)	-16(5)	-11(6)	-4(5)
C(38)	42(7)	51(8)	111(12)	-35(8)	-9(7)	-6(6)
C(39)	32(3)	37(3)	47(4)	-10(3)	2(3)	12(3)
C(40)	60(4)	64(4)	65(4)	-8(3)	-9(3)	-15(3)
C(41)	55(8)	44(7)	78(10)	1(7)	-10(7)	-5(6)
C(42)	36(7)	66(9)	56(8)	-8(6)	6(6)	0(6)
B(1X)	27(6)	24(6)	47(7)	-5(5)	-5(5)	9(5)
C(1X)	29(6)	41(6)	42(6)	2(5)	1(5)	2(5)
C(2X)	38(6)	50(7)	49(7)	2(6)	-10(5)	9(5)
C(3X)	54(8)	56(8)	49(8)	10(6)	-4(6)	-5(6)
C(4X)	45(7)	50(8)	61(8)	12(6)	1(6)	10(6)
C(5X)	46(7)	39(7)	56(8)	11(6)	14(6)	4(5)
C(6X)	31(6)	42(7)	55(7)	-6(5)	5(5)	4(5)
C(7X)	65(10)	70(10)	69(10)	15(8)	-19(8)	11(8)
F(7A)	76(8)	99(9)	64(7)	-7(6)	-18(6)	-37(7)
F(7B)	86(9)	203(17)	58(8)	-44(9)	13(7)	-27(10)
F(7C)	202(18)	96(11)	94(11)	19(9)	-69(13)	11(12)
F(7D)	80(20)	80(20)	68(19)	-1(16)	4(16)	-20(17)
F(7E)	130(30)	80(20)	80(20)	2(17)	-10(20)	-10(20)
F(7F)	60(20)	130(30)	110(30)	0(20)	-15(17)	-41(19)
C(8X)	57(9)	37(7)	76(10)	10(7)	18(8)	15(6)
F(8A)	95(10)	65(8)	112(11)	-20(7)	50(9)	1(7)
F(8B)	103(11)	51(7)	173(15)	-45(9)	-14(10)	-2(7)
F(8C)	182(15)	41(6)	77(8)	12(6)	16(10)	10(8)
F(8D)	90(20)	55(19)	100(20)	-26(17)	14(18)	-14(17)
F(8E)	100(20)	60(18)	90(20)	-11(16)	8(18)	20(18)
F(8F)	110(20)	100(20)	130(30)	-10(20)	0(20)	20(19)
C(9X)	44(7)	31(6)	41(6)	-2(5)	-2(5)	-8(5)
C(10X)	28(6)	37(6)	52(7)	7(5)	-2(5)	2(5)
C(11X)	43(7)	30(6)	53(7)	3(5)	11(6)	0(5)
C(12X)	40(6)	30(6)	47(7)	-8(5)	8(5)	-5(5)
C(13X)	39(6)	37(6)	45(7)	-11(5)	0(5)	-10(5)
C(14X)	26(5)	30(6)	52(7)	-6(5)	-1(5)	6(4)
C(15X)	60(9)	37(7)	82(10)	-2(7)	13(8)	23(6)
F(15B)	52(5)	74(6)	156(9)	6(6)	24(5)	14(4)
F(15A)	73(6)	63(6)	98(6)	-20(5)	10(5)	9(5)
F(15C)	144(16)	102(13)	97(11)	31(10)	26(11)	95(12)
F(15D)	41(11)	29(10)	160(20)	-6(13)	-9(13)	2(8)
F(15F)	46(11)	75(14)	118(17)	-28(13)	-26(12)	44(11)
C(16X)	47(8)	63(9)	45(7)	-7(6)	11(6)	2(6)
F(16A)	50(5)	183(10)	73(6)	9(6)	-19(4)	-10(6)
F(16B)	129(8)	95(7)	86(6)	32(5)	-44(6)	-4(6)
F(16C)	115(8)	147(9)	53(5)	-35(5)	-22(5)	36(7)
C(17X)	32(6)	42(6)	38(6)	1(5)	-3(5)	-3(5)
C(18X)	37(6)	28(6)	52(7)	-8(5)	-5(5)	-4(5)
C(19X)	44(7)	41(7)	52(7)	-2(6)	-6(6)	-5(5)
C(20X)	38(6)	48(7)	53(7)	-3(6)	-3(6)	-1(5)

C(21X)	42(7)	53(7)	43(7)	-1(6)	-1(5)	4(6)
C(22X)	39(6)	46(7)	34(6)	2(5)	-2(5)	0(5)
C(23X)	46(8)	49(8)	77(10)	-20(7)	-9(7)	-15(6)
F(23A)	55(8)	80(10)	102(11)	-54(8)	-12(7)	-8(7)
F(23B)	86(11)	123(13)	85(10)	-31(10)	10(8)	-82(10)
F(23C)	144(14)	51(8)	93(11)	-18(7)	-79(10)	10(9)
F(23D)	62(13)	105(17)	93(16)	-38(14)	-20(12)	-8(13)
F(23E)	130(20)	68(15)	140(20)	-26(15)	-59(17)	11(15)
F(23F)	78(16)	170(20)	78(15)	-23(16)	-15(13)	-51(16)
C(24X)	45(8)	90(11)	57(9)	-15(8)	-3(6)	-9(8)
F(24A)	55(5)	103(6)	88(6)	-50(5)	9(4)	-4(4)
F(24B)	53(5)	152(9)	92(6)	-54(6)	-12(4)	36(5)
F(24C)	102(7)	126(8)	61(5)	-5(5)	23(5)	-16(6)
C(25X)	34(6)	31(6)	36(6)	-2(5)	-1(5)	8(4)
C(26X)	33(6)	44(7)	51(7)	7(5)	-5(5)	-8(5)
C(27X)	47(7)	56(8)	43(7)	6(6)	-17(5)	-13(6)
C(28X)	47(7)	51(7)	48(7)	-6(6)	-8(6)	-12(6)
C(29X)	53(7)	26(6)	46(7)	-1(5)	1(5)	7(5)
C(30X)	35(6)	46(7)	38(6)	-5(5)	-1(5)	-3(5)
C(31X)	72(11)	72(11)	96(13)	11(10)	-32(10)	-25(9)
F(31A)	108(12)	79(10)	177(16)	53(11)	-87(12)	-62(9)
F(31B)	95(11)	119(13)	99(11)	30(10)	-70(9)	-31(10)
F(31C)	108(16)	220(20)	170(20)	-47(17)	-42(15)	3(16)
F(31D)	39(7)	50(7)	57(7)	5(5)	-9(5)	-12(5)
F(31E)	61(14)	54(12)	120(19)	37(13)	-56(13)	-28(10)
F(31F)	120(20)	160(20)	120(20)	-49(18)	-37(17)	-26(19)
C(32X)	53(9)	52(8)	85(11)	2(8)	-8(8)	-6(7)
F(32A)	38(6)	42(6)	77(10)	7(7)	2(6)	4(5)
F(32B)	116(12)	56(7)	145(12)	-42(8)	-47(9)	34(8)
F(32C)	50(7)	86(11)	164(14)	80(10)	-24(9)	-28(7)
F(32D)	110(30)	110(30)	120(30)	10(20)	-30(20)	0(20)
F(32E)	80(20)	39(15)	90(20)	-12(14)	-4(17)	-5(14)
F(32F)	50(20)	70(20)	90(30)	10(20)	24(17)	11(16)
B(2X)	26(6)	25(6)	46(7)	-8(5)	2(5)	8(5)
C(33X)	12(4)	40(6)	44(6)	-2(5)	0(4)	3(4)
C(34X)	29(5)	21(5)	45(6)	-7(4)	-6(4)	10(4)
C(35X)	30(6)	28(5)	47(7)	-1(5)	-6(5)	3(4)
C(36X)	28(6)	39(6)	44(6)	5(5)	-6(5)	0(5)
C(37X)	15(5)	42(6)	43(6)	-4(5)	-4(4)	1(4)
C(38X)	27(5)	25(5)	46(6)	-2(5)	-8(4)	-5(4)
C(39X)	25(6)	39(6)	66(8)	-1(6)	-3(5)	8(5)
F(39X)	74(5)	36(4)	113(6)	-12(4)	-42(5)	-8(4)
F(39B)	147(8)	31(4)	72(5)	8(4)	5(5)	-12(4)
F(39C)	58(5)	46(4)	153(8)	-25(5)	13(5)	6(4)
C(40X)	32(6)	38(6)	48(7)	0(5)	0(5)	-13(5)
F(40A)	52(4)	68(5)	53(4)	-16(3)	-2(3)	23(4)
F(40B)	96(6)	69(5)	39(4)	1(3)	-4(4)	-26(4)

F(40C)	38(4)	68(5)	83(5)	-34(4)	-14(3)	-7(3)
C(41X)	26(5)	32(6)	37(6)	-9(4)	-5(4)	3(4)
C(42X)	31(6)	41(6)	42(6)	-8(5)	-7(5)	-3(5)
C(43X)	28(6)	41(7)	59(7)	-17(6)	-6(5)	3(5)
C(44X)	40(7)	34(6)	54(7)	-10(5)	-5(5)	23(5)
C(45X)	44(7)	33(6)	47(7)	-6(5)	-5(5)	7(5)
C(46X)	34(6)	33(6)	35(6)	-3(5)	-3(5)	13(5)
C(47X)	29(7)	88(12)	100(13)	-20(9)	9(7)	9(7)
F(47A)	53(8)	108(12)	45(7)	-12(8)	4(6)	9(9)
F(47B)	40(7)	47(7)	145(13)	-23(8)	41(8)	-16(6)
F(47C)	43(7)	74(9)	102(10)	-2(7)	10(7)	31(6)
F(47D)	80(16)	51(12)	71(15)	-10(11)	40(13)	-8(11)
F(47E)	76(9)	83(9)	89(9)	-19(6)	9(6)	0(6)
F(47F)	110(20)	180(20)	130(20)	-33(19)	-9(16)	-41(19)
C(48X)	75(10)	29(6)	63(9)	-1(6)	4(7)	9(6)
F(48A)	91(13)	28(7)	103(13)	-25(8)	33(10)	-8(8)
F(48B)	72(10)	50(8)	117(14)	10(11)	31(9)	-16(7)
F(48C)	151(16)	71(10)	56(9)	17(8)	0(10)	-27(10)
F(48D)	120(20)	39(12)	93(18)	-14(15)	57(15)	-2(13)
F(48E)	108(19)	86(18)	120(20)	46(16)	-10(16)	11(14)
F(48F)	110(20)	58(17)	75(16)	-19(13)	-1(15)	-16(14)
C(49X)	21(5)	26(5)	38(6)	0(4)	-3(4)	2(4)
C(50X)	31(6)	29(5)	38(6)	-4(4)	-2(5)	2(4)
C(51X)	21(5)	34(6)	47(6)	-11(5)	-8(4)	2(4)
C(52X)	23(5)	40(6)	44(6)	-5(5)	0(5)	-2(4)
C(53X)	31(6)	23(5)	43(6)	-1(4)	1(5)	-3(4)
C(54X)	28(5)	21(5)	40(6)	-1(4)	-7(4)	-5(4)
C(55X)	23(6)	69(9)	54(8)	-5(7)	-9(5)	-9(6)
F(55A)	47(8)	56(9)	74(10)	-45(8)	-18(7)	-1(6)
F(55B)	50(10)	156(19)	89(13)	-56(13)	6(9)	-43(13)
F(55C)	143(17)	46(9)	105(13)	7(8)	-82(13)	-15(9)
F(55D)	43(11)	74(14)	82(15)	-9(12)	-32(10)	-6(11)
F(55E)	63(13)	140(20)	59(13)	-44(14)	-10(10)	-5(14)
F(55F)	120(20)	56(13)	120(20)	-38(12)	-58(16)	-3(12)
C(56X)	29(6)	41(7)	52(7)	-7(5)	2(5)	4(5)
F(56A)	133(8)	55(5)	95(6)	-29(5)	51(6)	-7(5)
F(56B)	46(5)	227(12)	115(7)	-106(8)	21(5)	-28(6)
F(56C)	138(8)	102(7)	49(5)	-9(5)	7(5)	55(6)
C(57X)	18(5)	26(5)	49(7)	-4(5)	-4(4)	9(4)
C(58X)	34(6)	44(7)	43(6)	-2(5)	-8(5)	-3(5)
C(59X)	29(6)	45(7)	56(7)	-1(6)	-9(5)	-11(5)
C(60X)	38(7)	58(8)	49(7)	-16(6)	-14(5)	-9(6)
C(61X)	29(6)	45(7)	41(6)	-6(5)	-8(5)	1(5)
C(62X)	25(5)	42(6)	44(6)	-8(5)	-3(5)	-4(5)
C(63X)	67(10)	81(12)	63(10)	-3(9)	-25(8)	-28(9)
F(63A)	85(12)	120(15)	107(11)	26(10)	-56(9)	-67(11)
F(63B)	74(11)	120(16)	99(12)	-18(10)	13(9)	-65(11)

F(63C)	111(12)	75(10)	141(16)	-17(10)	17(11)	-39(8)
F(63D)	70(20)	100(30)	110(30)	8(19)	22(18)	-9(18)
F(63E)	90(30)	80(20)	90(20)	-17(18)	-23(18)	-43(19)
F(63F)	70(20)	60(20)	80(20)	25(16)	-25(17)	-34(16)
C(64X)	38(7)	67(9)	51(8)	-11(6)	-10(6)	-3(6)
F(64A)	68(5)	118(7)	63(5)	16(5)	-34(4)	-23(5)
F(64B)	63(5)	94(6)	67(5)	19(4)	-10(4)	-22(5)
F(64C)	176(11)	136(9)	63(6)	9(6)	45(6)	79(8)

Table S13. Hydrogen coordinates (x10^4) and isotropic displacement parameters (Å^2 x10^3) for **Rh7**.

	x	y	z	U(eq)
H(2)	4886	7032	2198	48
H(3)	5170	6733	1455	51
H(5)	6638	4940	3755	43
H(6)	4972	5781	3727	49
H(7)	4416	6248	3000	46
H(8A)	6655	4414	1377	59
H(8B)	6751	5471	1130	59
H(9A)	4963	5472	986	76
H(9B)	4984	4366	1184	76
H(10A)	6211	5128	455	90
H(10B)	5219	4521	441	90
H(11A)	4818	2270	782	254
H(11B)	5511	1421	1030	254
H(11C)	5742	1729	535	254
H(12A)	8624	2494	970	246
H(12B)	9320	3367	780	246
H(12C)	8721	2817	471	246
H(13A)	6649	4188	-383	205
H(13B)	7743	3592	-401	205
H(13C)	7565	4421	-105	205
H(14)	5374	3514	1896	52
H(15)	5056	3954	2554	48
H(16A)	5395	3078	3183	64
H(16B)	4759	2281	3017	64
H(17A)	6542	1862	3274	74
H(17B)	6158	1371	2894	74
H(18)	7693	2713	2833	32
H(19)	8066	2544	2185	51
H(20A)	7144	1848	1743	84
H(20B)	6950	1028	2132	84
H(21A)	5370	1562	2270	84

H(21B)	5456	1915	1774	84
H(23)	10249	2273	3006	41
H(24)	10040	2621	3735	51
H(26)	8555	4176	1412	47
H(27)	10083	3104	1492	46
H(28)	10644	2817	2221	42
H(29A)	8635	5008	3753	81
H(29B)	9683	4629	3940	81
H(29C)	8446	4163	3994	81
H(29D)	8848	5086	3737	81
H(30A)	8797	3215	4276	71
H(30B)	7746	3822	4149	71
H(31A)	9235	4389	4673	73
H(31B)	8153	3967	4844	73
H(32A)	7529	6003	5673	109
H(32B)	6603	5766	5401	109
H(32C)	7500	4962	5529	109
H(33A)	9333	7500	4058	114
H(33B)	8828	7525	4526	114
H(33C)	9782	6781	4443	114
H(34A)	5229	5971	4454	120
H(34B)	5756	6324	4841	120
H(34C)	6006	6824	4369	120
H(30C)	10229	3820	4223	75
H(30D)	10371	4925	4049	75
H(31C)	9965	4808	4761	75
H(31D)	8993	4191	4723	75
H(32D)	7988	6105	5656	107
H(32E)	7232	5590	5390	107
H(32F)	8351	5099	5499	107
H(33D)	9255	8007	4019	107
H(33E)	8097	7804	4201	107
H(33F)	9000	7837	4519	107
H(34D)	5901	6063	4312	112
H(34E)	6326	6549	4687	112
H(34F)	6686	6908	4207	112
H(35)	10126	5361	2585	50
H(36)	9817	5850	3241	45
H(37A)	9756	7439	3349	52
H(37B)	9789	7780	2852	52
H(38A)	8204	8268	3038	78
H(38B)	8066	7394	3405	78
H(39)	6975	6939	2961	47
H(40)	7354	6451	2295	75
H(41A)	8670	7498	1870	71
H(41B)	8956	7887	2291	71
H(42A)	9822	6260	1951	64

H(42B)	10413	7070	2130	64
H(2X)	1933	8643	312	56
H(4X)	1518	11492	-73	65
H(6X)	2266	10524	1127	52
H(10X)	629	9654	1411	49
H(12X)	674	9342	2684	47
H(14X)	2905	7989	1996	43
H(18X)	3979	9175	1632	46
H(20X)	6621	8437	981	56
H(22X)	3812	7791	618	49
H(26X)	554	8072	878	52
H(28X)	614	5194	1183	58
H(30X)	3049	6704	1398	48
H(34X)	2492	2929	3718	38
H(36X)	2680	3435	2439	46
H(38X)	2782	729	3035	39
H(42X)	4416	374	3473	45
H(44X)	4483	-2513	3687	52
H(46X)	2069	-945	4158	42
H(50X)	1037	299	3523	40
H(52X)	-1534	841	4261	43
H(54X)	1284	1580	4564	36
H(58X)	4226	2237	3890	48
H(60X)	5066	1802	5101	56
H(62X)	2709	411	4748	44

Table S14. Torsion angles [°] for **Rh7**.

N(2)-C(1)-N(1)-C(4)	-172.3(9)
Rh(1)-C(1)-N(1)-C(4)	-5.0(13)
N(2)-C(1)-N(1)-C(2)	9.3(11)
Rh(1)-C(1)-N(1)-C(2)	176.6(7)
O(3)-Si(1)-O(1)-C(13)	176.1(15)
O(2)-Si(1)-O(1)-C(13)	-64.5(16)
C(10)-Si(1)-O(1)-C(13)	52.7(16)
N(1)-C(1)-N(2)-C(3)	-10.1(11)
Rh(1)-C(1)-N(2)-C(3)	-176.4(7)
N(1)-C(1)-N(2)-C(8)	175.9(10)
Rh(1)-C(1)-N(2)-C(8)	9.6(15)
C(1)-N(1)-C(2)-C(3)	-4.8(11)
C(4)-N(1)-C(2)-C(3)	176.8(9)
O(3)-Si(1)-O(2)-C(12)	70.2(17)
O(1)-Si(1)-O(2)-C(12)	-48.8(17)
C(10)-Si(1)-O(2)-C(12)	-169.1(15)
N(1)-C(2)-C(3)-N(2)	-1.3(11)

C(1)-N(2)-C(3)-C(2)	7.1(12)
C(8)-N(2)-C(3)-C(2)	-178.7(10)
O(1)-Si(1)-O(3)-C(11)	-35(3)
O(2)-Si(1)-O(3)-C(11)	-155(2)
C(10)-Si(1)-O(3)-C(11)	88(2)
C(1)-N(1)-C(4)-C(7)	-144.7(11)
C(2)-N(1)-C(4)-C(7)	33.5(16)
C(1)-N(1)-C(4)-S(1)	34.3(13)
C(2)-N(1)-C(4)-S(1)	-147.6(8)
C(5)-S(1)-C(4)-C(7)	-2.7(8)
Rh(2)-S(1)-C(4)-C(7)	-113.9(7)
C(5)-S(1)-C(4)-N(1)	178.1(8)
Rh(2)-S(1)-C(4)-N(1)	67.0(8)
C(4)-S(1)-C(5)-C(6)	3.9(9)
Rh(2)-S(1)-C(5)-C(6)	120.9(7)
S(1)-C(5)-C(6)-C(7)	-4.1(12)
N(1)-C(4)-C(7)-C(6)	-180.0(10)
S(1)-C(4)-C(7)-C(6)	1.0(12)
C(5)-C(6)-C(7)-C(4)	2.0(14)
C(1)-N(2)-C(8)-C(9)	113.1(13)
C(3)-N(2)-C(8)-C(9)	-60.0(15)
N(2)-C(8)-C(9)-C(10)	171.9(11)
C(8)-C(9)-C(10)-Si(1)	59.9(17)
O(3)-Si(1)-C(10)-C(9)	48.9(15)
O(1)-Si(1)-C(10)-C(9)	170.3(12)
O(2)-Si(1)-C(10)-C(9)	-70.0(14)
C(21)-C(14)-C(15)-C(16)	3.3(16)
Rh(1)-C(14)-C(15)-C(16)	-99.8(10)
C(21)-C(14)-C(15)-Rh(1)	103.1(10)
C(14)-C(15)-C(16)-C(17)	43.2(16)
Rh(1)-C(15)-C(16)-C(17)	-37.2(13)
C(15)-C(16)-C(17)-C(18)	29.7(17)
C(16)-C(17)-C(18)-C(19)	-88.8(16)
C(16)-C(17)-C(18)-Rh(1)	-7.2(15)
C(17)-C(18)-C(19)-C(20)	-0.2(19)
Rh(1)-C(18)-C(19)-C(20)	-102.7(12)
C(17)-C(18)-C(19)-Rh(1)	102.5(11)
C(18)-C(19)-C(20)-C(21)	49.9(19)
Rh(1)-C(19)-C(20)-C(21)	-35.5(16)
C(19)-C(20)-C(21)-C(14)	29(2)
C(15)-C(14)-C(21)-C(20)	-87.6(16)
Rh(1)-C(14)-C(21)-C(20)	-7.5(17)
C(24)-N(4)-C(22)-N(3)	-4.8(11)
C(29)-N(4)-C(22)-N(3)	175.3(10)
C(24)-N(4)-C(22)-Rh(2)	-173.1(8)
C(29)-N(4)-C(22)-Rh(2)	6.9(16)
C(25)-N(3)-C(22)-N(4)	179.5(8)

C(23)-N(3)-C(22)-N(4)	4.9(10)
C(25)-N(3)-C(22)-Rh(2)	-11.1(13)
C(23)-N(3)-C(22)-Rh(2)	174.3(7)
C(25)-N(3)-C(23)-C(24)	-178.0(9)
C(22)-N(3)-C(23)-C(24)	-3.4(11)
N(3)-C(23)-C(24)-N(4)	0.4(12)
C(22)-N(4)-C(24)-C(23)	2.9(13)
C(29)-N(4)-C(24)-C(23)	-177.1(11)
C(23)-N(3)-C(25)-C(28)	-25.8(15)
C(22)-N(3)-C(25)-C(28)	160.4(10)
C(23)-N(3)-C(25)-S(2)	154.0(8)
C(22)-N(3)-C(25)-S(2)	-19.8(12)
C(26)-S(2)-C(25)-C(28)	3.6(8)
Rh(1)-S(2)-C(25)-C(28)	107.4(7)
C(26)-S(2)-C(25)-N(3)	-176.3(8)
Rh(1)-S(2)-C(25)-N(3)	-72.4(7)
C(25)-S(2)-C(26)-C(27)	-2.8(8)
Rh(1)-S(2)-C(26)-C(27)	-114.9(8)
S(2)-C(26)-C(27)-C(28)	1.4(12)
N(3)-C(25)-C(28)-C(27)	176.4(9)
S(2)-C(25)-C(28)-C(27)	-3.5(11)
C(26)-C(27)-C(28)-C(25)	1.4(13)
C(22)-N(4)-C(29)-C(30)	126.4(15)
C(24)-N(4)-C(29)-C(30)	-53.5(19)
C(22)-N(4)-C(29)-C(30A)	-135.6(17)
C(24)-N(4)-C(29)-C(30A)	45(2)
N(4)-C(29)-C(30)-C(31)	166.8(15)
C(29)-C(30)-C(31)-Si(2)	61(3)
C(30)-C(31)-Si(2)-O(4)	175.9(18)
C(30)-C(31)-Si(2)-O(6)	57(2)
C(30)-C(31)-Si(2)-O(5)	-60(2)
O(6)-Si(2)-O(4)-C(32)	44(2)
O(5)-Si(2)-O(4)-C(32)	162(2)
C(31)-Si(2)-O(4)-C(32)	-71(2)
O(4)-Si(2)-O(5)-C(33)	23(2)
O(6)-Si(2)-O(5)-C(33)	142(2)
C(31)-Si(2)-O(5)-C(33)	-103(2)
O(4)-Si(2)-O(6)-C(34)	46(2)
O(5)-Si(2)-O(6)-C(34)	-72(2)
C(31)-Si(2)-O(6)-C(34)	166(2)
N(4)-C(29)-C(30A)-C(31A)	-163.1(18)
C(29)-C(30A)-C(31A)-Si(2A)	-52(3)
C(30A)-C(31A)-Si(2A)-O(5A)	-43(2)
C(30A)-C(31A)-Si(2A)-O(6A)	76(2)
C(30A)-C(31A)-Si(2A)-O(4A)	-160(2)
O(5A)-Si(2A)-O(4A)-C(32A)	-179(2)
O(6A)-Si(2A)-O(4A)-C(32A)	60(3)

C(31A)-Si(2A)-O(4A)-C(32A)	-64(3)
O(6A)-Si(2A)-O(5A)-C(33A)	82(2)
O(4A)-Si(2A)-O(5A)-C(33A)	-39(2)
C(31A)-Si(2A)-O(5A)-C(33A)	-160(2)
O(5A)-Si(2A)-O(6A)-C(34A)	-66(2)
O(4A)-Si(2A)-O(6A)-C(34A)	54(2)
C(31A)-Si(2A)-O(6A)-C(34A)	179(2)
C(42)-C(35)-C(36)-C(37)	-2.4(16)
Rh(2)-C(35)-C(36)-C(37)	-104.1(10)
C(42)-C(35)-C(36)-Rh(2)	101.7(10)
C(35)-C(36)-C(37)-C(38)	89.7(14)
Rh(2)-C(36)-C(37)-C(38)	8.3(14)
C(36)-C(37)-C(38)-C(39)	-33.9(18)
C(37)-C(38)-C(39)-C(40)	-36(2)
C(37)-C(38)-C(39)-Rh(2)	38.2(14)
C(38)-C(39)-C(40)-C(41)	-11.3(19)
Rh(2)-C(39)-C(40)-C(41)	-99.2(10)
C(38)-C(39)-C(40)-Rh(2)	87.9(13)
C(39)-C(40)-C(41)-C(42)	96.0(14)
Rh(2)-C(40)-C(41)-C(42)	18.6(14)
C(36)-C(35)-C(42)-C(41)	-46.4(16)
Rh(2)-C(35)-C(42)-C(41)	33.8(13)
C(40)-C(41)-C(42)-C(35)	-36.0(17)
C(9X)-B(1X)-C(1X)-C(6X)	40.3(13)
C(17X)-B(1X)-C(1X)-C(6X)	-82.8(12)
C(25X)-B(1X)-C(1X)-C(6X)	155.2(9)
C(9X)-B(1X)-C(1X)-C(2X)	-145.0(11)
C(17X)-B(1X)-C(1X)-C(2X)	91.9(12)
C(25X)-B(1X)-C(1X)-C(2X)	-30.1(14)
C(6X)-C(1X)-C(2X)-C(3X)	-2.7(16)
B(1X)-C(1X)-C(2X)-C(3X)	-177.6(10)
C(1X)-C(2X)-C(3X)-C(4X)	0.4(19)
C(1X)-C(2X)-C(3X)-C(7X)	-179.0(11)
C(2X)-C(3X)-C(4X)-C(5X)	1.9(19)
C(7X)-C(3X)-C(4X)-C(5X)	-178.7(11)
C(3X)-C(4X)-C(5X)-C(6X)	-1.8(18)
C(3X)-C(4X)-C(5X)-C(8X)	-179.7(11)
C(2X)-C(1X)-C(6X)-C(5X)	2.8(16)
B(1X)-C(1X)-C(6X)-C(5X)	177.9(10)
C(4X)-C(5X)-C(6X)-C(1X)	-0.6(17)
C(8X)-C(5X)-C(6X)-C(1X)	177.4(10)
C(4X)-C(3X)-C(7X)-F(7D)	47(3)
C(2X)-C(3X)-C(7X)-F(7D)	-134(3)
C(4X)-C(3X)-C(7X)-F(7E)	173(3)
C(2X)-C(3X)-C(7X)-F(7E)	-8(4)
C(4X)-C(3X)-C(7X)-F(7C)	-21(2)
C(2X)-C(3X)-C(7X)-F(7C)	158.4(16)

C(4X)-C(3X)-C(7X)-F(7A)	-138.8(13)
C(2X)-C(3X)-C(7X)-F(7A)	40.6(17)
C(4X)-C(3X)-C(7X)-F(7F)	-57(3)
C(2X)-C(3X)-C(7X)-F(7F)	122(3)
C(4X)-C(3X)-C(7X)-F(7B)	101.4(16)
C(2X)-C(3X)-C(7X)-F(7B)	-79.2(17)
C(4X)-C(5X)-C(8X)-F(8D)	-65(3)
C(6X)-C(5X)-C(8X)-F(8D)	117(3)
C(4X)-C(5X)-C(8X)-F(8C)	-4.9(19)
C(6X)-C(5X)-C(8X)-F(8C)	177.2(14)
C(4X)-C(5X)-C(8X)-F(8E)	162(3)
C(6X)-C(5X)-C(8X)-F(8E)	-15(3)
C(4X)-C(5X)-C(8X)-F(8A)	114.8(15)
C(6X)-C(5X)-C(8X)-F(8A)	-63.1(17)
C(4X)-C(5X)-C(8X)-F(8F)	45(3)
C(6X)-C(5X)-C(8X)-F(8F)	-133(3)
C(4X)-C(5X)-C(8X)-F(8B)	-123.1(15)
C(6X)-C(5X)-C(8X)-F(8B)	58.9(15)
C(17X)-B(1X)-C(9X)-C(10X)	146.3(10)
C(25X)-B(1X)-C(9X)-C(10X)	-92.5(11)
C(1X)-B(1X)-C(9X)-C(10X)	29.2(14)
C(17X)-B(1X)-C(9X)-C(14X)	-40.5(14)
C(25X)-B(1X)-C(9X)-C(14X)	80.7(11)
C(1X)-B(1X)-C(9X)-C(14X)	-157.6(10)
C(14X)-C(9X)-C(10X)-C(11X)	5.5(16)
B(1X)-C(9X)-C(10X)-C(11X)	179.0(10)
C(9X)-C(10X)-C(11X)-C(12X)	-3.7(17)
C(9X)-C(10X)-C(11X)-C(15X)	177.8(10)
C(10X)-C(11X)-C(12X)-C(13X)	-0.6(17)
C(15X)-C(11X)-C(12X)-C(13X)	178.0(10)
C(11X)-C(12X)-C(13X)-C(14X)	2.6(16)
C(11X)-C(12X)-C(13X)-C(16X)	-178.9(11)
C(10X)-C(9X)-C(14X)-C(13X)	-3.4(15)
B(1X)-C(9X)-C(14X)-C(13X)	-177.0(10)
C(12X)-C(13X)-C(14X)-C(9X)	-0.6(16)
C(16X)-C(13X)-C(14X)-C(9X)	-179.1(11)
C(10X)-C(11X)-C(15X)-F(15D)	-71(2)
C(12X)-C(11X)-C(15X)-F(15D)	110(2)
C(10X)-C(11X)-C(15X)-F(15B)	123.2(12)
C(12X)-C(11X)-C(15X)-F(15B)	-55.3(15)
C(10X)-C(11X)-C(15X)-F(15C)	-17(2)
C(12X)-C(11X)-C(15X)-F(15C)	164.5(19)
C(10X)-C(11X)-C(15X)-F(15A)	-131.9(12)
C(12X)-C(11X)-C(15X)-F(15A)	49.6(14)
C(10X)-C(11X)-C(15X)-F(15F)	37.4(19)
C(12X)-C(11X)-C(15X)-F(15F)	-141.2(18)
C(12X)-C(13X)-C(16X)-F(16B)	106.1(13)

C(14X)-C(13X)-C(16X)-F(16B)	-75.4(14)
C(12X)-C(13X)-C(16X)-F(16A)	-135.6(12)
C(14X)-C(13X)-C(16X)-F(16A)	43.0(17)
C(12X)-C(13X)-C(16X)-F(16C)	-11.2(18)
C(14X)-C(13X)-C(16X)-F(16C)	167.4(11)
C(9X)-B(1X)-C(17X)-C(18X)	-20.5(15)
C(25X)-B(1X)-C(17X)-C(18X)	-136.4(11)
C(1X)-B(1X)-C(17X)-C(18X)	100.4(12)
C(9X)-B(1X)-C(17X)-C(22X)	165.9(9)
C(25X)-B(1X)-C(17X)-C(22X)	50.0(13)
C(1X)-B(1X)-C(17X)-C(22X)	-73.2(12)
C(22X)-C(17X)-C(18X)-C(19X)	0.0(16)
B(1X)-C(17X)-C(18X)-C(19X)	-173.9(10)
C(17X)-C(18X)-C(19X)-C(20X)	-0.3(18)
C(17X)-C(18X)-C(19X)-C(23X)	-176.8(10)
C(18X)-C(19X)-C(20X)-C(21X)	0.5(18)
C(23X)-C(19X)-C(20X)-C(21X)	177.0(10)
C(19X)-C(20X)-C(21X)-C(22X)	-0.4(17)
C(19X)-C(20X)-C(21X)-C(24X)	179.1(11)
C(20X)-C(21X)-C(22X)-C(17X)	0.1(18)
C(24X)-C(21X)-C(22X)-C(17X)	-179.4(11)
C(18X)-C(17X)-C(22X)-C(21X)	0.1(16)
B(1X)-C(17X)-C(22X)-C(21X)	174.3(10)
C(18X)-C(19X)-C(23X)-F(23F)	41(2)
C(20X)-C(19X)-C(23X)-F(23F)	-136(2)
C(18X)-C(19X)-C(23X)-F(23B)	-141.5(14)
C(20X)-C(19X)-C(23X)-F(23B)	41.9(16)
C(18X)-C(19X)-C(23X)-F(23E)	-82(2)
C(20X)-C(19X)-C(23X)-F(23E)	102(2)
C(18X)-C(19X)-C(23X)-F(23C)	96.9(15)
C(20X)-C(19X)-C(23X)-F(23C)	-79.7(15)
C(18X)-C(19X)-C(23X)-F(23D)	161.8(18)
C(20X)-C(19X)-C(23X)-F(23D)	-14.8(19)
C(18X)-C(19X)-C(23X)-F(23A)	-20.6(16)
C(20X)-C(19X)-C(23X)-F(23A)	162.7(12)
C(22X)-C(21X)-C(24X)-F(24B)	-146.9(13)
C(20X)-C(21X)-C(24X)-F(24B)	33.6(18)
C(22X)-C(21X)-C(24X)-F(24A)	-22.0(19)
C(20X)-C(21X)-C(24X)-F(24A)	158.5(12)
C(22X)-C(21X)-C(24X)-F(24C)	93.6(14)
C(20X)-C(21X)-C(24X)-F(24C)	-85.9(14)
C(9X)-B(1X)-C(25X)-C(26X)	81.7(12)
C(17X)-B(1X)-C(25X)-C(26X)	-155.6(10)
C(1X)-B(1X)-C(25X)-C(26X)	-38.1(14)
C(9X)-B(1X)-C(25X)-C(30X)	-87.6(11)
C(17X)-B(1X)-C(25X)-C(30X)	35.1(14)
C(1X)-B(1X)-C(25X)-C(30X)	152.6(9)

C(30X)-C(25X)-C(26X)-C(27X)	-1.1(17)
B(1X)-C(25X)-C(26X)-C(27X)	-171.0(11)
C(25X)-C(26X)-C(27X)-C(28X)	0.4(19)
C(25X)-C(26X)-C(27X)-C(31X)	178.9(11)
C(26X)-C(27X)-C(28X)-C(29X)	0.9(18)
C(31X)-C(27X)-C(28X)-C(29X)	-177.6(11)
C(27X)-C(28X)-C(29X)-C(30X)	-1.3(17)
C(27X)-C(28X)-C(29X)-C(32X)	178.5(10)
C(26X)-C(25X)-C(30X)-C(29X)	0.6(16)
B(1X)-C(25X)-C(30X)-C(29X)	170.8(10)
C(28X)-C(29X)-C(30X)-C(25X)	0.6(17)
C(32X)-C(29X)-C(30X)-C(25X)	-179.3(10)
C(26X)-C(27X)-C(31X)-F(31E)	-11(2)
C(28X)-C(27X)-C(31X)-F(31E)	167(2)
C(26X)-C(27X)-C(31X)-F(31A)	173.5(17)
C(28X)-C(27X)-C(31X)-F(31A)	-8(2)
C(26X)-C(27X)-C(31X)-F(31C)	-50(2)
C(28X)-C(27X)-C(31X)-F(31C)	129(2)
C(26X)-C(27X)-C(31X)-F(31F)	122(3)
C(28X)-C(27X)-C(31X)-F(31F)	-60(3)
C(26X)-C(27X)-C(31X)-F(31B)	62.1(18)
C(28X)-C(27X)-C(31X)-F(31B)	-119.4(16)
C(26X)-C(27X)-C(31X)-F(31D)	-120.5(15)
C(28X)-C(27X)-C(31X)-F(31D)	58.0(17)
C(28X)-C(29X)-C(32X)-F(32F)	170(3)
C(30X)-C(29X)-C(32X)-F(32F)	-10(3)
C(28X)-C(29X)-C(32X)-F(32C)	-49.2(17)
C(30X)-C(29X)-C(32X)-F(32C)	130.7(15)
C(28X)-C(29X)-C(32X)-F(32E)	23(2)
C(30X)-C(29X)-C(32X)-F(32E)	-157(2)
C(28X)-C(29X)-C(32X)-F(32A)	-165.8(13)
C(30X)-C(29X)-C(32X)-F(32A)	14.1(17)
C(28X)-C(29X)-C(32X)-F(32D)	-79(3)
C(30X)-C(29X)-C(32X)-F(32D)	101(3)
C(28X)-C(29X)-C(32X)-F(32B)	75.0(14)
C(30X)-C(29X)-C(32X)-F(32B)	-105.1(14)
C(41X)-B(2X)-C(33X)-C(38X)	23.8(13)
C(49X)-B(2X)-C(33X)-C(38X)	-95.2(10)
C(57X)-B(2X)-C(33X)-C(38X)	139.1(9)
C(41X)-B(2X)-C(33X)-C(34X)	-161.3(9)
C(49X)-B(2X)-C(33X)-C(34X)	79.7(11)
C(57X)-B(2X)-C(33X)-C(34X)	-46.0(12)
C(38X)-C(33X)-C(34X)-C(35X)	-3.3(14)
B(2X)-C(33X)-C(34X)-C(35X)	-178.7(9)
C(33X)-C(34X)-C(35X)-C(36X)	2.7(15)
C(33X)-C(34X)-C(35X)-C(39X)	-178.8(9)
C(34X)-C(35X)-C(36X)-C(37X)	-0.5(15)

C(39X)-C(35X)-C(36X)-C(37X)	-179.0(9)
C(35X)-C(36X)-C(37X)-C(38X)	-0.7(14)
C(35X)-C(36X)-C(37X)-C(40X)	178.7(9)
C(36X)-C(37X)-C(38X)-C(33X)	-0.1(15)
C(40X)-C(37X)-C(38X)-C(33X)	-179.5(9)
C(34X)-C(33X)-C(38X)-C(37X)	2.0(14)
B(2X)-C(33X)-C(38X)-C(37X)	177.1(9)
C(36X)-C(35X)-C(39X)-F(39B)	-13.1(15)
C(34X)-C(35X)-C(39X)-F(39B)	168.4(10)
C(36X)-C(35X)-C(39X)-F(39C)	110.6(12)
C(34X)-C(35X)-C(39X)-F(39C)	-67.9(14)
C(36X)-C(35X)-C(39X)-F(39X)	-132.2(11)
C(34X)-C(35X)-C(39X)-F(39X)	49.3(13)
C(38X)-C(37X)-C(40X)-F(40A)	-37.6(13)
C(36X)-C(37X)-C(40X)-F(40A)	143.0(9)
C(38X)-C(37X)-C(40X)-F(40C)	83.0(12)
C(36X)-C(37X)-C(40X)-F(40C)	-96.5(11)
C(38X)-C(37X)-C(40X)-F(40B)	-159.1(9)
C(36X)-C(37X)-C(40X)-F(40B)	21.5(13)
C(33X)-B(2X)-C(41X)-C(46X)	-138.3(10)
C(49X)-B(2X)-C(41X)-C(46X)	-22.2(14)
C(57X)-B(2X)-C(41X)-C(46X)	99.9(11)
C(33X)-B(2X)-C(41X)-C(42X)	46.5(13)
C(49X)-B(2X)-C(41X)-C(42X)	162.7(9)
C(57X)-B(2X)-C(41X)-C(42X)	-75.3(11)
C(46X)-C(41X)-C(42X)-C(43X)	1.4(15)
B(2X)-C(41X)-C(42X)-C(43X)	177.1(10)
C(41X)-C(42X)-C(43X)-C(44X)	-1.6(17)
C(41X)-C(42X)-C(43X)-C(47X)	179.2(10)
C(42X)-C(43X)-C(44X)-C(45X)	0.2(17)
C(47X)-C(43X)-C(44X)-C(45X)	179.4(10)
C(43X)-C(44X)-C(45X)-C(46X)	1.3(17)
C(43X)-C(44X)-C(45X)-C(48X)	179.9(10)
C(44X)-C(45X)-C(46X)-C(41X)	-1.4(17)
C(48X)-C(45X)-C(46X)-C(41X)	-179.9(10)
C(42X)-C(41X)-C(46X)-C(45X)	0.0(15)
B(2X)-C(41X)-C(46X)-C(45X)	-175.3(10)
C(44X)-C(43X)-C(47X)-F(47A)	70.9(14)
C(42X)-C(43X)-C(47X)-F(47A)	-109.8(13)
C(44X)-C(43X)-C(47X)-F(47B)	-157.4(12)
C(42X)-C(43X)-C(47X)-F(47B)	21.8(16)
C(44X)-C(43X)-C(47X)-F(47F)	-101(2)
C(42X)-C(43X)-C(47X)-F(47F)	78(2)
C(44X)-C(43X)-C(47X)-F(47D)	35.6(17)
C(42X)-C(43X)-C(47X)-F(47D)	-145.2(15)
C(44X)-C(43X)-C(47X)-F(47C)	-43.7(13)
C(42X)-C(43X)-C(47X)-F(47C)	135.5(11)

C(44X)-C(43X)-C(47X)-F(47E)	142.9(14)
C(42X)-C(43X)-C(47X)-F(47E)	-37.8(16)
C(44X)-C(45X)-C(48X)-F(48D)	174(2)
C(46X)-C(45X)-C(48X)-F(48D)	-7(2)
C(44X)-C(45X)-C(48X)-F(48C)	95.1(16)
C(46X)-C(45X)-C(48X)-F(48C)	-86.3(16)
C(44X)-C(45X)-C(48X)-F(48F)	-58(2)
C(46X)-C(45X)-C(48X)-F(48F)	121(2)
C(44X)-C(45X)-C(48X)-F(48A)	-27.1(18)
C(46X)-C(45X)-C(48X)-F(48A)	151.5(15)
C(44X)-C(45X)-C(48X)-F(48B)	-147.6(14)
C(46X)-C(45X)-C(48X)-F(48B)	31.0(17)
C(44X)-C(45X)-C(48X)-F(48E)	53(2)
C(46X)-C(45X)-C(48X)-F(48E)	-129(2)
C(33X)-B(2X)-C(49X)-C(50X)	62.2(11)
C(41X)-B(2X)-C(49X)-C(50X)	-57.1(12)
C(57X)-B(2X)-C(49X)-C(50X)	-172.4(8)
C(33X)-B(2X)-C(49X)-C(54X)	-112.1(10)
C(41X)-B(2X)-C(49X)-C(54X)	128.5(10)
C(57X)-B(2X)-C(49X)-C(54X)	13.3(14)
C(54X)-C(49X)-C(50X)-C(51X)	-1.8(15)
B(2X)-C(49X)-C(50X)-C(51X)	-176.6(9)
C(49X)-C(50X)-C(51X)-C(52X)	0.0(16)
C(49X)-C(50X)-C(51X)-C(55X)	178.4(10)
C(50X)-C(51X)-C(52X)-C(53X)	-1.0(15)
C(55X)-C(51X)-C(52X)-C(53X)	-179.4(10)
C(51X)-C(52X)-C(53X)-C(54X)	3.9(15)
C(51X)-C(52X)-C(53X)-C(56X)	-178.8(9)
C(52X)-C(53X)-C(54X)-C(49X)	-5.9(15)
C(56X)-C(53X)-C(54X)-C(49X)	176.8(9)
C(50X)-C(49X)-C(54X)-C(53X)	4.6(14)
B(2X)-C(49X)-C(54X)-C(53X)	179.1(9)
C(50X)-C(51X)-C(55X)-F(55E)	-35(2)
C(52X)-C(51X)-C(55X)-F(55E)	143(2)
C(50X)-C(51X)-C(55X)-F(55B)	163.1(19)
C(52X)-C(51X)-C(55X)-F(55B)	-19(2)
C(50X)-C(51X)-C(55X)-F(55A)	33.6(18)
C(52X)-C(51X)-C(55X)-F(55A)	-148.0(13)
C(50X)-C(51X)-C(55X)-F(55D)	-154.0(16)
C(52X)-C(51X)-C(55X)-F(55D)	24(2)
C(50X)-C(51X)-C(55X)-F(55F)	94(2)
C(52X)-C(51X)-C(55X)-F(55F)	-87(2)
C(50X)-C(51X)-C(55X)-F(55C)	-81.2(19)
C(52X)-C(51X)-C(55X)-F(55C)	97.2(18)
C(54X)-C(53X)-C(56X)-F(56B)	176.4(11)
C(52X)-C(53X)-C(56X)-F(56B)	-0.9(16)
C(54X)-C(53X)-C(56X)-F(56A)	51.0(14)

C(52X)-C(53X)-C(56X)-F(56A)	-126.3(11)
C(54X)-C(53X)-C(56X)-F(56C)	-63.9(14)
C(52X)-C(53X)-C(56X)-F(56C)	118.7(12)
C(33X)-B(2X)-C(57X)-C(62X)	168.6(9)
C(41X)-B(2X)-C(57X)-C(62X)	-71.6(11)
C(49X)-B(2X)-C(57X)-C(62X)	47.7(12)
C(33X)-B(2X)-C(57X)-C(58X)	-17.8(13)
C(41X)-B(2X)-C(57X)-C(58X)	101.9(11)
C(49X)-B(2X)-C(57X)-C(58X)	-138.8(10)
C(62X)-C(57X)-C(58X)-C(59X)	0.3(15)
B(2X)-C(57X)-C(58X)-C(59X)	-173.6(10)
C(57X)-C(58X)-C(59X)-C(60X)	-1.6(18)
C(57X)-C(58X)-C(59X)-C(63X)	179.1(12)
C(58X)-C(59X)-C(60X)-C(61X)	2.2(18)
C(63X)-C(59X)-C(60X)-C(61X)	-178.5(12)
C(59X)-C(60X)-C(61X)-C(62X)	-1.6(17)
C(59X)-C(60X)-C(61X)-C(64X)	-178.7(11)
C(60X)-C(61X)-C(62X)-C(57X)	0.4(17)
C(64X)-C(61X)-C(62X)-C(57X)	177.4(10)
C(58X)-C(57X)-C(62X)-C(61X)	0.3(15)
B(2X)-C(57X)-C(62X)-C(61X)	174.3(10)
C(60X)-C(59X)-C(63X)-F(63F)	-145(4)
C(58X)-C(59X)-C(63X)-F(63F)	34(4)
C(60X)-C(59X)-C(63X)-F(63B)	141(2)
C(58X)-C(59X)-C(63X)-F(63B)	-40(2)
C(60X)-C(59X)-C(63X)-F(63E)	-25(4)
C(58X)-C(59X)-C(63X)-F(63E)	154(4)
C(60X)-C(59X)-C(63X)-F(63A)	15(3)
C(58X)-C(59X)-C(63X)-F(63A)	-165(2)
C(60X)-C(59X)-C(63X)-F(63C)	-104.1(19)
C(58X)-C(59X)-C(63X)-F(63C)	75(2)
C(60X)-C(59X)-C(63X)-F(63D)	106(3)
C(58X)-C(59X)-C(63X)-F(63D)	-75(3)
C(62X)-C(61X)-C(64X)-F(64C)	-86.9(16)
C(60X)-C(61X)-C(64X)-F(64C)	90.1(15)
C(62X)-C(61X)-C(64X)-F(64A)	147.6(11)
C(60X)-C(61X)-C(64X)-F(64A)	-35.4(16)
C(62X)-C(61X)-C(64X)-F(64B)	30.7(16)
C(60X)-C(61X)-C(64X)-F(64B)	-152.3(11)

Crystal data of **Rh9**(Synthesized from ligand containing bromide anion):

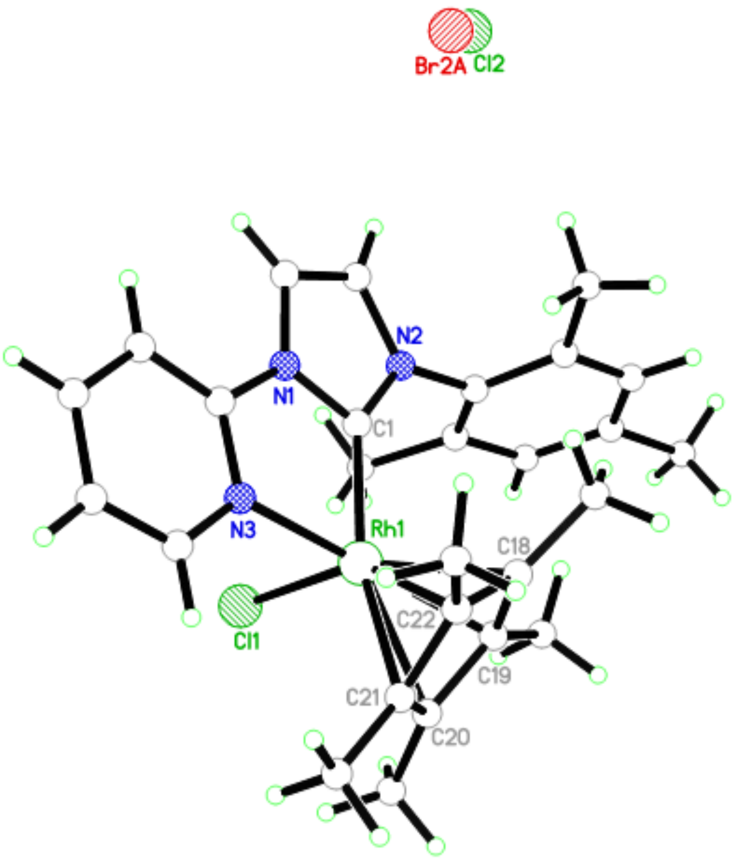

Figure S101. Single crystal X-ray structure of **Rh9** (Synthesized from ligand containing bromide anion). Co-crystallized solvent molecules were removed for clarity

Table S15. Crystal data and structure refinement for **Rh9**

Empirical formula	$C_{27}H_{32}Br_{0.25}Cl_{1.75}N_3Rh$
Formula weight	583.48
Temperature	140(2) K
Wavelength	0.71073 Å
Crystal system, space group	Triclinic, P-1
Unit cell dimensions	a = 8.1019(5) Å, α = 100.872(3)°
	b = 11.3477(7) Å, β = 97.198(3)°
	c = 18.5097(11) Å, γ = 98.071(3)°
Volume	634.36(17) Å^3
Z, Calculated density	2, 1.186 Mg/m^3
Absorption coefficient	0.987 mm^{-1}
F (000)	597
Crystal size	0.130 x 0.086 x 0.079 mm

Θ-range for data collection	2.269 to 26.403 °
Limiting indices	-10<=h<=10, -14<=k<=13, -23<=l<=23
Reflections collected / unique	28445 / 6635 [R(int) = 0.0487]
Completeness to Θ = 25.242	99.2 %
Absorption correction	Numerical
Max. and min. transmission	0.8334 and 0.7674
Refinement method	Full-matrix least-squares on F^2
Data / restraints / parameters	6635 / 0 / 315
Goodness-of-fit on F^2	1.050
Final R indices [I>2σ(I)]	R1 = 0.0742, wR2 = 0.1649
R indices (all data)	R1 = 0.1113, wR2 = 0.1751
Extinction coefficient	n/a
Largest diff. peak and hole	2.234 and -1.333 e.Å^{-3}

Table S16. Atomic coordinates ($x10^4$) and equivalent isotropic displacement parameters (Å^{2}x10^3) for **Rh9**. U(eq) is defined as one third of the trace of the orthogonalized U_{ij} tensor.

	x	y	z	U(eq)
Rh(1)	3934(1)	1427(1)	2631(1)	28(1)
Cl(1)	6686(2)	1833(2)	3383(1)	44(1)
N(1)	5363(8)	2890(5)	1677(4)	31(1)
C(1)	4498(9)	3003(7)	2268(4)	28(2)
Cl(2)	3217(5)	6656(4)	623(2)	37(1)
Br(2A)	3234(7)	6178(4)	393(2)	27(1)
N(2)	4327(8)	4198(6)	2400(4)	39(2)
C(2)	5749(12)	3988(7)	1458(5)	49(2)
N(3)	5188(7)	880(5)	1718(3)	27(1)
C(3)	5104(12)	4796(8)	1904(6)	51(2)
C(4)	5736(9)	1753(7)	1367(4)	28(2)
C(5)	5529(10)	-225(7)	1484(4)	35(2)
C(6)	6402(10)	-515(7)	907(4)	34(2)
C(7)	6949(10)	410(7)	552(4)	36(2)
C(8)	6616(9)	1562(7)	788(4)	29(2)
C(9)	3555(10)	4826(7)	2969(5)	37(2)
C(10)	1979(10)	5151(7)	2782(5)	36(2)
C(11)	1251(10)	5754(7)	3349(5)	39(2)
C(12)	2058(12)	6096(9)	4069(5)	48(2)
C(13)	3641(13)	5817(10)	4240(6)	63(3)
C(14)	4438(11)	5211(9)	3684(6)	58(3)
C(15)	1119(11)	4879(8)	1981(4)	39(2)

C(16)	1191(14)	6726(12)	4686(6)	75(4)
C(17)	6215(13)	4963(13)	3884(7)	88(5)
C(18)	1244(9)	1358(7)	2631(4)	34(2)
C(19)	2088(10)	1548(8)	3369(5)	39(2)
C(20)	2841(11)	496(9)	3466(5)	44(2)
C(21)	2569(11)	-309(8)	2757(5)	43(2)
C(22)	1590(10)	215(8)	2235(5)	39(2)
C(23)	90(10)	2099(8)	2310(5)	39(2)
C(24)	1943(13)	2556(9)	3999(5)	57(3)
C(25)	3753(16)	308(13)	4157(6)	81(4)
C(26)	2952(14)	-1610(10)	2616(6)	65(3)
C(27)	963(11)	-348(8)	1431(5)	44(2)

Table S17. Bond lengths [Å] and angles [°] for **Rh9**.

Rh(1)-C(1)	2.040(7)
Rh(1)-N(3)	2.119(6)
Rh(1)-C(22)	2.138(8)
Rh(1)-C(19)	2.149(8)
Rh(1)-C(18)	2.170(7)
Rh(1)-C(21)	2.187(8)
Rh(1)-C(20)	2.232(8)
Rh(1)-Cl(1)	2.411(2)
N(1)-C(1)	1.368(9)
N(1)-C(2)	1.388(10)
N(1)-C(4)	1.399(9)
C(1)-N(2)	1.362(10)
N(2)-C(3)	1.399(11)
N(2)-C(9)	1.420(9)
C(2)-C(3)	1.323(12)
C(2)-H(2)	0.9500
N(3)-C(5)	1.326(10)
N(3)-C(4)	1.338(9)
C(3)-H(3)	0.9500
C(4)-C(8)	1.362(10)
C(5)-C(6)	1.368(11)
C(5)-H(5)	0.9500
C(6)-C(7)	1.391(11)
C(6)-H(6)	0.9500
C(7)-C(8)	1.373(11)
C(7)-H(7)	0.9500

C(8)-H(8)	0.9500
C(9)-C(14)	1.382(13)
C(9)-C(10)	1.399(11)
C(10)-C(11)	1.384(11)
C(10)-C(15)	1.513(11)
C(11)-C(12)	1.366(12)
C(11)-H(11)	0.9500
C(12)-C(13)	1.378(13)
C(12)-C(16)	1.530(12)
C(13)-C(14)	1.404(13)
C(13)-H(13)	0.9500
C(14)-C(17)	1.520(13)
C(15)-H(15A)	0.9800
C(15)-H(15B)	0.9800
C(15)-H(15C)	0.9800
C(16)-H(16A)	0.9800
C(16)-H(16B)	0.9800
C(16)-H(16C)	0.9800
C(17)-H(17A)	0.9800
C(17)-H(17B)	0.9800
C(17)-H(17C)	0.9800
C(18)-C(19)	1.412(11)
C(18)-C(22)	1.446(12)
C(18)-C(23)	1.485(11)
C(19)-C(20)	1.446(12)
C(19)-C(24)	1.500(12)
C(20)-C(21)	1.422(13)
C(20)-C(25)	1.462(13)
C(21)-C(22)	1.440(12)
C(21)-C(26)	1.532(13)
C(22)-C(27)	1.498(12)
C(23)-H(23A)	0.9800
C(23)-H(23B)	0.9800
C(23)-H(23C)	0.9800
C(24)-H(24A)	0.9800
C(24)-H(24B)	0.9800
C(24)-H(24C)	0.9800
C(25)-H(25A)	0.9800
C(25)-H(25B)	0.9800
C(25)-H(25C)	0.9800
C(26)-H(26A)	0.9800

C(26)-H(26B)	0.9800
C(26)-H(26C)	0.9800
C(27)-H(27A)	0.9800
C(27)-H(27B)	0.9800
C(27)-H(27C)	0.9800

C(1)-Rh(1)-N(3)	77.3(3)
C(1)-Rh(1)-C(22)	122.1(3)
N(3)-Rh(1)-C(22)	98.1(3)
C(1)-Rh(1)-C(19)	111.9(3)
N(3)-Rh(1)-C(19)	162.9(3)
C(22)-Rh(1)-C(19)	64.8(3)
C(1)-Rh(1)-C(18)	100.1(3)
N(3)-Rh(1)-C(18)	128.0(3)
C(22)-Rh(1)-C(18)	39.2(3)
C(19)-Rh(1)-C(18)	38.2(3)
C(1)-Rh(1)-C(21)	160.9(3)
N(3)-Rh(1)-C(21)	102.1(3)
C(22)-Rh(1)-C(21)	38.9(3)
C(19)-Rh(1)-C(21)	64.2(3)
C(18)-Rh(1)-C(21)	64.9(3)
C(1)-Rh(1)-C(20)	148.0(3)
N(3)-Rh(1)-C(20)	134.6(3)
C(22)-Rh(1)-C(20)	64.0(3)
C(19)-Rh(1)-C(20)	38.5(3)
C(18)-Rh(1)-C(20)	64.0(3)
C(21)-Rh(1)-C(20)	37.5(3)
C(1)-Rh(1)-Cl(1)	89.8(2)
N(3)-Rh(1)-Cl(1)	85.94(16)
C(22)-Rh(1)-Cl(1)	148.0(2)
C(19)-Rh(1)-Cl(1)	107.9(2)
C(18)-Rh(1)-Cl(1)	145.9(2)
C(21)-Rh(1)-Cl(1)	109.2(2)
C(20)-Rh(1)-Cl(1)	90.6(2)
C(1)-N(1)-C(2)	112.0(6)
C(1)-N(1)-C(4)	119.7(6)
C(2)-N(1)-C(4)	128.3(6)
N(2)-C(1)-N(1)	103.3(6)
N(2)-C(1)-Rh(1)	142.7(6)
N(1)-C(1)-Rh(1)	113.9(5)

C(1)-N(2)-C(3)	110.6(6)
C(1)-N(2)-C(9)	127.5(7)
C(3)-N(2)-C(9)	121.9(7)
C(3)-C(2)-N(1)	106.2(7)
C(3)-C(2)-H(2)	126.9
N(1)-C(2)-H(2)	126.9
C(5)-N(3)-C(4)	117.7(6)
C(5)-N(3)-Rh(1)	126.2(5)
C(4)-N(3)-Rh(1)	116.1(5)
C(2)-C(3)-N(2)	107.9(8)
C(2)-C(3)-H(3)	126.1
N(2)-C(3)-H(3)	126.1
N(3)-C(4)-C(8)	123.4(7)
N(3)-C(4)-N(1)	112.9(6)
C(8)-C(4)-N(1)	123.6(7)
N(3)-C(5)-C(6)	123.6(7)
N(3)-C(5)-H(5)	118.2
C(6)-C(5)-H(5)	118.2
C(5)-C(6)-C(7)	117.4(7)
C(5)-C(6)-H(6)	121.3
C(7)-C(6)-H(6)	121.3
C(8)-C(7)-C(6)	119.9(7)
C(8)-C(7)-H(7)	120.0
C(6)-C(7)-H(7)	120.0
C(4)-C(8)-C(7)	118.0(7)
C(4)-C(8)-H(8)	121.0
C(7)-C(8)-H(8)	121.0
C(14)-C(9)-C(10)	120.8(7)
C(14)-C(9)-N(2)	119.6(8)
C(10)-C(9)-N(2)	119.3(7)
C(11)-C(10)-C(9)	118.0(8)
C(11)-C(10)-C(15)	121.1(7)
C(9)-C(10)-C(15)	120.9(7)
C(12)-C(11)-C(10)	122.3(8)
C(12)-C(11)-H(11)	118.8
C(10)-C(11)-H(11)	118.8
C(11)-C(12)-C(13)	119.1(8)
C(11)-C(12)-C(16)	120.9(8)
C(13)-C(12)-C(16)	120.0(9)
C(12)-C(13)-C(14)	120.8(9)
C(12)-C(13)-H(13)	119.6

C(14)-C(13)-H(13)	119.6
C(9)-C(14)-C(13)	118.6(8)
C(9)-C(14)-C(17)	121.7(8)
C(13)-C(14)-C(17)	119.7(9)
C(10)-C(15)-H(15A)	109.5
C(10)-C(15)-H(15B)	109.5
H(15A)-C(15)-H(15B)	109.5
C(10)-C(15)-H(15C)	109.5
H(15A)-C(15)-H(15C)	109.5
H(15B)-C(15)-H(15C)	109.5
C(12)-C(16)-H(16A)	109.5
C(12)-C(16)-H(16B)	109.5
H(16A)-C(16)-H(16B)	109.5
C(12)-C(16)-H(16C)	109.5
H(16A)-C(16)-H(16C)	109.5
H(16B)-C(16)-H(16C)	109.5
C(14)-C(17)-H(17A)	109.5
C(14)-C(17)-H(17B)	109.5
H(17A)-C(17)-H(17B)	109.5
C(14)-C(17)-H(17C)	109.5
H(17A)-C(17)-H(17C)	109.5
H(17B)-C(17)-H(17C)	109.5
C(19)-C(18)-C(22)	106.9(7)
C(19)-C(18)-C(23)	128.4(8)
C(22)-C(18)-C(23)	124.4(7)
C(19)-C(18)-Rh(1)	70.1(4)
C(22)-C(18)-Rh(1)	69.2(4)
C(23)-C(18)-Rh(1)	129.5(5)
C(18)-C(19)-C(20)	109.5(8)
C(18)-C(19)-C(24)	125.6(8)
C(20)-C(19)-C(24)	123.9(8)
C(18)-C(19)-Rh(1)	71.7(4)
C(20)-C(19)-Rh(1)	73.9(4)
C(24)-C(19)-Rh(1)	130.2(6)
C(21)-C(20)-C(19)	107.1(7)
C(21)-C(20)-C(25)	126.4(9)
C(19)-C(20)-C(25)	126.5(9)
C(21)-C(20)-Rh(1)	69.5(4)
C(19)-C(20)-Rh(1)	67.7(4)
C(25)-C(20)-Rh(1)	126.6(7)
C(20)-C(21)-C(22)	108.2(7)

C(20)-C(21)-C(26)	125.0(8)
C(22)-C(21)-C(26)	125.8(9)
C(20)-C(21)-Rh(1)	72.9(5)
C(22)-C(21)-Rh(1)	68.7(4)
C(26)-C(21)-Rh(1)	132.9(6)
C(21)-C(22)-C(18)	108.1(8)
C(21)-C(22)-C(27)	126.2(8)
C(18)-C(22)-C(27)	125.6(7)
C(21)-C(22)-Rh(1)	72.4(5)
C(18)-C(22)-Rh(1)	71.6(4)
C(27)-C(22)-Rh(1)	124.0(6)
C(18)-C(23)-H(23A)	109.5
C(18)-C(23)-H(23B)	109.5
H(23A)-C(23)-H(23B)	109.5
C(18)-C(23)-H(23C)	109.5
H(23A)-C(23)-H(23C)	109.5
H(23B)-C(23)-H(23C)	109.5
C(19)-C(24)-H(24A)	109.5
C(19)-C(24)-H(24B)	109.5
H(24A)-C(24)-H(24B)	109.5
C(19)-C(24)-H(24C)	109.5
H(24A)-C(24)-H(24C)	109.5
H(24B)-C(24)-H(24C)	109.5
C(20)-C(25)-H(25A)	109.5
C(20)-C(25)-H(25B)	109.5
H(25A)-C(25)-H(25B)	109.5
C(20)-C(25)-H(25C)	109.5
H(25A)-C(25)-H(25C)	109.5
H(25B)-C(25)-H(25C)	109.5
C(21)-C(26)-H(26A)	109.5
C(21)-C(26)-H(26B)	109.5
H(26A)-C(26)-H(26B)	109.5
C(21)-C(26)-H(26C)	109.5
H(26A)-C(26)-H(26C)	109.5
H(26B)-C(26)-H(26C)	109.5
C(22)-C(27)-H(27A)	109.5
C(22)-C(27)-H(27B)	109.5
H(27A)-C(27)-H(27B)	109.5
C(22)-C(27)-H(27C)	109.5
H(27A)-C(27)-H(27C)	109.5
H(27B)-C(27)-H(27C)	109.5

Table S18. Anisotropic displacement parameters ($Å^2x10^3$) for **Rh9**. The anisotropic displacement factor exponent takes the form: $-2 \Pi^2[h^2a^{*2}U_{11} + ... + 2hka^*b^*U_{12}]$.

	U11	U22	U33	U23	U13	U12
Rh(1)	27(1)	38(1)	22(1)	8(1)	6(1)	8(1)
Cl(1)	31(1)	72(2)	27(1)	0(1)	-3(1)	19(1)
N(1)	31(3)	25(3)	38(4)	5(3)	12(3)	5(3)
C(1)	23(3)	27(4)	31(4)	3(3)	7(3)	1(3)
Cl(2)	34(2)	43(3)	39(2)	29(2)	0(2)	-1(2)
Br(2A)	44(2)	25(2)	23(2)	10(2)	19(2)	24(2)
N(2)	37(4)	31(4)	46(4)	-5(3)	15(3)	3(3)
C(2)	60(6)	27(4)	67(6)	7(4)	43(5)	5(4)
N(3)	24(3)	31(3)	25(3)	4(3)	4(2)	3(3)
C(3)	56(6)	32(5)	70(7)	11(5)	30(5)	8(4)
C(4)	21(3)	30(4)	31(4)	2(3)	3(3)	6(3)
C(5)	39(4)	31(4)	38(5)	10(4)	8(4)	12(4)
C(6)	35(4)	35(4)	35(4)	6(4)	9(3)	11(3)
C(7)	41(4)	41(5)	30(4)	6(4)	9(3)	13(4)
C(8)	31(4)	29(4)	30(4)	10(3)	8(3)	7(3)
C(9)	43(5)	27(4)	38(5)	-9(3)	16(4)	5(4)
C(10)	37(4)	28(4)	42(5)	4(4)	9(4)	5(3)
C(11)	34(4)	37(5)	47(5)	3(4)	9(4)	9(4)
C(12)	46(5)	51(6)	40(5)	-10(4)	6(4)	10(4)
C(13)	60(6)	67(7)	52(6)	-19(5)	-2(5)	26(5)
C(14)	35(5)	56(6)	65(7)	-26(5)	1(5)	9(4)
C(15)	50(5)	38(5)	35(4)	18(4)	11(4)	13(4)
C(16)	60(7)	103(9)	56(7)	-21(6)	13(5)	42(7)
C(17)	47(6)	113(10)	78(8)	-44(7)	-15(6)	28(7)
C(18)	27(4)	42(5)	38(4)	22(4)	9(3)	2(3)
C(19)	35(4)	52(5)	36(5)	14(4)	15(4)	15(4)
C(20)	45(5)	61(6)	47(5)	34(5)	25(4)	29(4)
C(21)	44(5)	51(5)	50(5)	33(5)	24(4)	17(4)
C(22)	30(4)	39(5)	52(5)	19(4)	13(4)	0(4)
C(23)	30(4)	42(5)	51(5)	19(4)	6(4)	9(4)
C(24)	66(6)	70(7)	41(5)	11(5)	26(5)	20(5)
C(25)	90(9)	127(11)	54(7)	49(7)	23(6)	56(8)
C(26)	73(7)	65(7)	78(8)	38(6)	34(6)	26(6)
C(27)	43(5)	41(5)	49(5)	16(4)	4(4)	1(4)

Table S19. Hydrogen coordinates (x10^4) and isotropic displacement parameters (Å^{2}x10^3) for **Rh9**.

	x	y	z	U(eq)
H(2)	6355	4131	1067	59
H(3)	5159	5633	1890	61
H(5)	5147	-850	1731	42
H(6)	6625	-1317	754	41
H(7)	7553	246	146	44
H(8)	6989	2206	554	35
H(11)	152	5936	3234	47
H(13)	4201	6036	4741	76
H(15A)	1601	5498	1728	58
H(15B)	-92	4889	1967	58
H(15C)	1290	4075	1730	58
H(16A)	2016	7035	5141	112
H(16B)	278	6141	4783	112
H(16C)	724	7405	4528	112
H(17A)	6591	5250	4421	132
H(17B)	6980	5391	3612	132
H(17C)	6220	4086	3748	132
H(23A)	-1076	1779	2348	59
H(23B)	199	2061	1785	59
H(23C)	387	2946	2584	59
H(24A)	964	2311	4232	85
H(24B)	1800	3290	3808	85
H(24C)	2969	2726	4369	85
H(25A)	2952	-76	4434	122
H(25B)	4334	1094	4461	122
H(25C)	4583	-221	4040	122
H(26A)	4096	-1610	2862	98
H(26B)	2870	-1918	2079	98
H(26C)	2136	-2133	2816	98
H(27A)	1708	-906	1246	66
H(27B)	954	292	1143	66
H(27C)	-184	-800	1379	66

Table S20. Torsion angles [°] for **Rh9**.

C(2)-N(1)-C(1)-N(2)	1.3(9)
C(4)-N(1)-C(1)-N(2)	-178.3(6)

C(2)-N(1)-C(1)-Rh(1)	-178.8(6)
C(4)-N(1)-C(1)-Rh(1)	1.7(8)
N(1)-C(1)-N(2)-C(3)	-1.1(9)
Rh(1)-C(1)-N(2)-C(3)	179.0(8)
N(1)-C(1)-N(2)-C(9)	-178.4(7)
Rh(1)-C(1)-N(2)-C(9)	1.7(14)
C(1)-N(1)-C(2)-C(3)	-1.0(11)
C(4)-N(1)-C(2)-C(3)	178.5(8)
N(1)-C(2)-C(3)-N(2)	0.3(11)
C(1)-N(2)-C(3)-C(2)	0.5(11)
C(9)-N(2)-C(3)-C(2)	178.0(8)
C(5)-N(3)-C(4)-C(8)	0.0(11)
Rh(1)-N(3)-C(4)-C(8)	177.6(6)
C(5)-N(3)-C(4)-N(1)	-177.7(6)
Rh(1)-N(3)-C(4)-N(1)	-0.2(8)
C(1)-N(1)-C(4)-N(3)	-1.0(9)
C(2)-N(1)-C(4)-N(3)	179.6(8)
C(1)-N(1)-C(4)-C(8)	-178.8(7)
C(2)-N(1)-C(4)-C(8)	1.8(12)
C(4)-N(3)-C(5)-C(6)	-0.1(11)
Rh(1)-N(3)-C(5)-C(6)	-177.4(6)
N(3)-C(5)-C(6)-C(7)	-0.1(12)
C(5)-C(6)-C(7)-C(8)	0.4(12)
N(3)-C(4)-C(8)-C(7)	0.3(11)
N(1)-C(4)-C(8)-C(7)	177.8(7)
C(6)-C(7)-C(8)-C(4)	-0.5(11)
C(1)-N(2)-C(9)-C(14)	79.6(11)
C(3)-N(2)-C(9)-C(14)	-97.5(11)
C(1)-N(2)-C(9)-C(10)	-106.8(10)
C(3)-N(2)-C(9)-C(10)	76.1(10)
C(14)-C(9)-C(10)-C(11)	-6.9(13)
N(2)-C(9)-C(10)-C(11)	179.5(7)
C(14)-C(9)-C(10)-C(15)	171.5(8)
N(2)-C(9)-C(10)-C(15)	-2.0(12)
C(9)-C(10)-C(11)-C(12)	3.7(13)
C(15)-C(10)-C(11)-C(12)	-174.7(8)
C(10)-C(11)-C(12)-C(13)	-1.0(15)
C(10)-C(11)-C(12)-C(16)	-178.1(9)
C(11)-C(12)-C(13)-C(14)	1.3(17)
C(16)-C(12)-C(13)-C(14)	178.4(11)
C(10)-C(9)-C(14)-C(13)	7.3(15)

N(2)-C(9)-C(14)-C(13)	-179.2(9)
C(10)-C(9)-C(14)-C(17)	-174.6(10)
N(2)-C(9)-C(14)-C(17)	-1.1(16)
C(12)-C(13)-C(14)-C(9)	-4.5(17)
C(12)-C(13)-C(14)-C(17)	177.4(11)
C(22)-C(18)-C(19)-C(20)	-5.0(9)
C(23)-C(18)-C(19)-C(20)	170.1(7)
Rh(1)-C(18)-C(19)-C(20)	-64.7(6)
C(22)-C(18)-C(19)-C(24)	-173.4(8)
C(23)-C(18)-C(19)-C(24)	1.7(14)
Rh(1)-C(18)-C(19)-C(24)	126.9(9)
C(22)-C(18)-C(19)-Rh(1)	59.7(5)
C(23)-C(18)-C(19)-Rh(1)	-125.2(8)
C(18)-C(19)-C(20)-C(21)	4.8(9)
C(24)-C(19)-C(20)-C(21)	173.5(8)
Rh(1)-C(19)-C(20)-C(21)	-58.6(6)
C(18)-C(19)-C(20)-C(25)	-176.8(9)
C(24)-C(19)-C(20)-C(25)	-8.1(15)
Rh(1)-C(19)-C(20)-C(25)	119.9(10)
C(18)-C(19)-C(20)-Rh(1)	63.3(6)
C(24)-C(19)-C(20)-Rh(1)	-128.0(9)
C(19)-C(20)-C(21)-C(22)	-2.6(9)
C(25)-C(20)-C(21)-C(22)	178.9(9)
Rh(1)-C(20)-C(21)-C(22)	-60.0(5)
C(19)-C(20)-C(21)-C(26)	-171.7(8)
C(25)-C(20)-C(21)-C(26)	9.8(15)
Rh(1)-C(20)-C(21)-C(26)	130.9(9)
C(19)-C(20)-C(21)-Rh(1)	57.4(6)
C(25)-C(20)-C(21)-Rh(1)	-121.1(10)
C(20)-C(21)-C(22)-C(18)	-0.4(9)
C(26)-C(21)-C(22)-C(18)	168.6(8)
Rh(1)-C(21)-C(22)-C(18)	-63.1(5)
C(20)-C(21)-C(22)-C(27)	-177.7(8)
C(26)-C(21)-C(22)-C(27)	-8.7(13)
Rh(1)-C(21)-C(22)-C(27)	119.7(8)
C(20)-C(21)-C(22)-Rh(1)	62.7(6)
C(26)-C(21)-C(22)-Rh(1)	-128.3(8)
C(19)-C(18)-C(22)-C(21)	3.3(8)
C(23)-C(18)-C(22)-C(21)	-172.0(7)
Rh(1)-C(18)-C(22)-C(21)	63.6(5)
C(19)-C(18)-C(22)-C(27)	-179.4(7)

C(23)-C(18)-C(22)-C(27)	5.3(12)
Rh(1)-C(18)-C(22)-C(27)	-119.1(8)
C(19)-C(18)-C(22)-Rh(1)	-60.3(5)
C(23)-C(18)-C(22)-Rh(1)	124.3(7)

Crystal data of **Rh13**:

A - weak - disorder is found at the silyl radical. THF also cocrystallized.

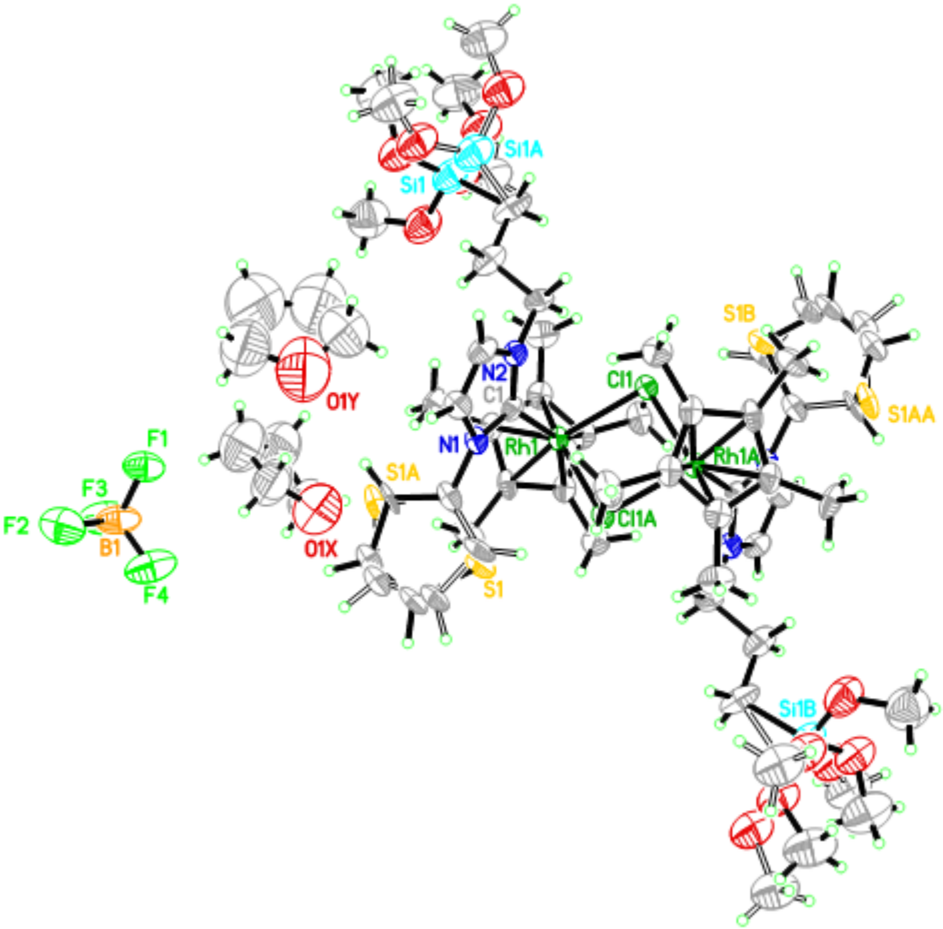

Figure S102. Single crystal X-ray structure of **Rh13**.

Table S21. Crystal data and structure refinement for **Rh13**.

Empirical formula	$C_{62}H_{102}B_2Cl_2F_8N_4O_{10}Rh_2S_2Si_2$
Formula weight	1634.11
Temperature	140(2) K
Wavelength	0.71073 Å
Crystal system, space group	Triclinic, P-1
Unit cell dimensions	a = 11.3444(14) Å, α = 76.950(6) °
	b = 12.2238(15) Å, β = 88.496(6) °
	c = 16.765(2) Å, γ = 63.650(5) °
Volume	2022.1(4) $Å^3$
Z, Calculated density	1, 1.342 Mg/m^3

Absorption coefficient	0.624 mm^{-1}
F (000)	848
Crystal size	0.490 x 0.262 x 0.214 mm
Θ range for data collection	1.915 to 28.315 °
Limiting indices	-14<=h<=15, -16<=k<=16, -22<=l<=13
Reflections collected / unique	37059 / 9585 [R(int) = 0.0523]
Completeness to Θ = 25.242	97.1 %
Absorption correction	Numerical
Max. and min. transmission	0.9513 and 0.7904
Refinement method	Full-matrix least-squares on F^2
Data / restraints / parameters	9585 / 469 / 531
Goodness-of-fit on F^2	1.061
Final R indices [I>2σ (I)]	R1 = 0.0743, wR2 = 0.1842
R indices (all data)	R1 = 0.1092, wR2 = 0.1961
Extinction coefficient	0.0072(9)
Largest diff. peak and hole	1.596 and -1.148 e.$Å^{-3}$

Table S22. Atomic coordinates (x 10^4) and equivalent isotropic displacement parameters ($Å^2$ x 10^3) for **Rh13**. U(eq) is defined as one third of the trace of the orthogonalized U_{ij} tensor.

	x	y	z	U(eq)
Rh(1)	10025(1)	9114(1)	4216(1)	24(1)
Cl(1)	10211(1)	11003(1)	4320(1)	27(1)
N(1)	7048(4)	9866(4)	4328(3)	28(1)
C(1)	8008(5)	10145(5)	3978(3)	25(1)
N(2)	7332(5)	11327(5)	3505(3)	31(1)
C(2)	5811(6)	10874(6)	4091(4)	36(1)
C(3)	5985(6)	11786(6)	3583(4)	37(1)
C(4)	7157(5)	8682(5)	4764(3)	33(1)
S(1)	7688(4)	7963(3)	5763(2)	38(1)
C(5)	7212(10)	6814(8)	5735(7)	36(2)
C(6)	6675(10)	6994(10)	4970(6)	39(2)
C(7)	6630(20)	8100(14)	4380(10)	35(3)
S(1A)	6526(11)	7884(9)	4348(6)	43(2)
C(5A)	6810(20)	6767(16)	5273(9)	38(3)
C(6A)	7360(20)	7078(18)	5855(12)	38(3)
C(7A)	7590(30)	8210(20)	5577(8)	39(4)
C(8)	7849(6)	12009(6)	2885(4)	37(1)
C(9)	7401(8)	12056(8)	2023(4)	52(2)
C(10)	7985(9)	12689(9)	1364(4)	64(2)

Si(1)	7771(4)	12299(4)	373(2)	68(1)
O(1)	8438(9)	10789(7)	501(5)	79(2)
O(2)	6330(9)	12737(9)	57(6)	81(2)
O(3)	8520(9)	12928(9)	-221(5)	80(2)
C(11)	8207(17)	10155(15)	-77(9)	95(4)
C(12)	5493(17)	13821(15)	-604(9)	111(4)
C(13)	8760(17)	12871(17)	-1077(6)	103(4)
Si(1A)	7766(12)	12955(13)	309(8)	78(2)
O(1A)	8940(19)	11707(19)	48(15)	79(2)
O(2A)	6201(19)	13240(20)	158(18)	80(2)
O(3A)	7990(30)	14090(20)	-318(14)	84(3)
C(11A)	10350(20)	11270(40)	-140(30)	98(7)
C(12A)	4840(20)	14260(30)	-80(30)	97(5)
C(13A)	7980(50)	14470(40)	-1210(30)	94(6)
C(14)	10311(6)	7991(6)	3339(4)	33(1)
C(15)	11062(6)	8687(6)	3159(4)	33(1)
C(16)	12058(5)	8215(6)	3838(4)	31(1)
C(17)	11871(5)	7310(5)	4430(4)	29(1)
C(18)	10750(5)	7206(5)	4135(4)	32(1)
C(19)	9269(6)	8054(7)	2778(4)	42(2)
C(20)	11015(7)	9542(7)	2381(4)	49(2)
C(21)	13116(6)	8622(6)	3869(5)	42(2)
C(22)	12687(6)	6570(6)	5223(4)	40(2)
C(23)	10295(7)	6254(6)	4565(5)	43(2)
B(1)	3833(11)	5509(9)	2952(6)	63(3)
F(1)	3703(5)	6688(4)	2560(3)	61(1)
F(2)	2553(6)	5573(6)	2928(4)	89(2)
F(3)	4643(6)	4647(5)	2536(3)	93(2)
F(4)	4250(6)	5183(5)	3741(3)	91(2)
O(1X)	8209(12)	5563(11)	3366(7)	142(4)
C(1X)	6999(15)	6049(15)	2933(9)	121(5)
C(2X)	7185(17)	5866(17)	2076(10)	141(6)
C(3X)	8629(17)	5345(16)	1997(10)	138(6)
C(4X)	9176(15)	4761(14)	2885(9)	116(4)
O(1Y)	5011(16)	9465(16)	2424(10)	197(6)
C(1Y)	4420(20)	9230(20)	1776(13)	158(7)
C(2Y)	5020(30)	9480(20)	1069(16)	207(10)
C(3Y)	6070(20)	9740(20)	1343(15)	197(9)
C(4Y)	5645(18)	10120(17)	2115(11)	138(6)

Table S23. Bond lengths [Å] and angles [°] for **Rh13**.

Rh(1)-C(1)	2.060(5)
Rh(1)-C(15)	2.134(6)
Rh(1)-C(18)	2.137(6)
Rh(1)-C(14)	2.153(5)
Rh(1)-C(16)	2.217(5)
Rh(1)-C(17)	2.227(5)
Rh(1)-Cl(1)#1	2.4412(15)
Rh(1)-Cl(1)	2.4548(14)
Cl(1)-Rh(1)#1	2.4412(15)
N(1)-C(1)	1.361(7)
N(1)-C(2)	1.385(7)
N(1)-C(4)	1.418(7)
C(1)-N(2)	1.355(7)
N(2)-C(3)	1.389(7)
N(2)-C(8)	1.459(8)
C(2)-C(3)	1.328(9)
C(2)-H(2)	0.9500
C(3)-H(3)	0.9500
C(4)-C(7A)	1.364(10)
C(4)-C(7)	1.370(9)
C(4)-S(1)	1.691(6)
C(4)-S(1A)	1.704(8)
S(1)-C(5)	1.723(7)
C(5)-C(6)	1.363(9)
C(5)-H(5)	0.9500
C(6)-C(7)	1.466(9)
C(6)-H(6)	0.9500
C(7)-H(7)	0.9500
S(1A)-C(5A)	1.747(10)
C(5A)-C(6A)	1.378(10)
C(5A)-H(5A)	0.9500
C(6A)-C(7A)	1.485(10)
C(6A)-H(6A)	0.9500
C(7A)-H(7A)	0.9500
C(8)-C(9)	1.524(9)
C(8)-H(8A)	0.9900
C(8)-H(8B)	0.9900
C(9)-C(10)	1.514(12)
C(9)-H(9A)	0.9900

C(9)-H(9B)	0.9900
C(10)-Si(1A)	1.729(14)
C(10)-Si(1)	1.878(9)
C(10)-H(10A)	0.9900
C(10)-H(10B)	0.9900
Si(1)-O(2)	1.543(9)
Si(1)-O(3)	1.579(9)
Si(1)-O(1)	1.617(7)
O(1)-C(11)	1.464(9)
O(2)-C(12)	1.470(9)
O(3)-C(13)	1.465(7)
C(11)-H(11A)	0.9800
C(11)-H(11B)	0.9800
C(11)-H(11C)	0.9800
C(12)-H(12A)	0.9800
C(12)-H(12B)	0.9800
C(12)-H(12C)	0.9800
C(13)-H(13A)	0.9800
C(13)-H(13B)	0.9800
C(13)-H(13C)	0.9800
Si(1A)-O(2A)	1.665(17)
Si(1A)-O(3A)	1.657(17)
Si(1A)-O(1A)	1.661(9)
O(1A)-C(11A)	1.491(10)
O(2A)-C(12A)	1.484(10)
O(3A)-C(13A)	1.46(4)
C(11A)-H(11D)	0.9800
C(11A)-H(11E)	0.9800
C(11A)-H(11F)	0.9800
C(12A)-H(12D)	0.9800
C(12A)-H(12E)	0.9800
C(12A)-H(12F)	0.9800
C(13A)-H(13D)	0.9800
C(13A)-H(13E)	0.9800
C(13A)-H(13F)	0.9800
C(14)-C(18)	1.407(9)
C(14)-C(15)	1.433(9)
C(14)-C(19)	1.494(8)
C(15)-C(16)	1.447(8)
C(15)-C(20)	1.461(9)
C(16)-C(17)	1.399(9)

C(16)-C(21)	1.495(9)
C(17)-C(18)	1.444(8)
C(17)-C(22)	1.490(9)
C(18)-C(23)	1.508(9)
C(19)-H(19A)	0.9800
C(19)-H(19B)	0.9800
C(19)-H(19C)	0.9800
C(20)-H(20A)	0.9800
C(20)-H(20B)	0.9800
C(20)-H(20C)	0.9800
C(21)-H(21A)	0.9800
C(21)-H(21B)	0.9800
C(21)-H(21C)	0.9800
C(22)-H(22A)	0.9800
C(22)-H(22B)	0.9800
C(22)-H(22C)	0.9800
C(23)-H(23A)	0.9800
C(23)-H(23B)	0.9800
C(23)-H(23C)	0.9800
B(1)-F(4)	1.326(11)
B(1)-F(3)	1.371(10)
B(1)-F(1)	1.385(11)
B(1)-F(2)	1.421(13)
O(1X)-C(1X)	1.377(15)
O(1X)-C(4X)	1.473(17)
C(1X)-C(2X)	1.498(19)
C(1X)-H(1X1)	0.9900
C(1X)-H(1X2)	0.9900
C(2X)-C(3X)	1.49(2)
C(2X)-H(2X1)	0.9900
C(2X)-H(2X2)	0.9900
C(3X)-C(4X)	1.512(19)
C(3X)-H(3X1)	0.9900
C(3X)-H(3X2)	0.9900
C(4X)-H(4X1)	0.9900
C(4X)-H(4X2)	0.9900
O(1Y)-C(4Y)	1.316(19)
O(1Y)-C(1Y)	1.44(2)
C(1Y)-C(2Y)	1.39(3)
C(1Y)-H(1Y1)	0.9900
C(1Y)-H(1Y2)	0.9900

C(2Y)-C(3Y)	1.47(3)
C(2Y)-H(2Y1)	0.9900
C(2Y)-H(2Y2)	0.9900
C(3Y)-C(4Y)	1.47(2)
C(3Y)-H(3Y1)	0.9900
C(3Y)-H(3Y2)	0.9900
C(4Y)-H(4Y1)	0.9900
C(4Y)-H(4Y2)	0.9900

C(1)-Rh(1)-C(15)	114.5(2)
C(1)-Rh(1)-C(18)	113.7(2)
C(15)-Rh(1)-C(18)	65.1(2)
C(1)-Rh(1)-C(14)	96.8(2)
C(15)-Rh(1)-C(14)	39.1(2)
C(18)-Rh(1)-C(14)	38.3(2)
C(1)-Rh(1)-C(16)	153.1(2)
C(15)-Rh(1)-C(16)	38.8(2)
C(18)-Rh(1)-C(16)	63.7(2)
C(14)-Rh(1)-C(16)	64.0(2)
C(1)-Rh(1)-C(17)	151.9(2)
C(15)-Rh(1)-C(17)	63.9(2)
C(18)-Rh(1)-C(17)	38.6(2)
C(14)-Rh(1)-C(17)	63.7(2)
C(16)-Rh(1)-C(17)	36.7(2)
C(1)-Rh(1)-Cl(1)#1	89.79(15)
C(15)-Rh(1)-Cl(1)#1	155.58(17)
C(18)-Rh(1)-Cl(1)#1	103.63(18)
C(14)-Rh(1)-Cl(1)#1	140.17(18)
C(16)-Rh(1)-Cl(1)#1	117.13(16)
C(17)-Rh(1)-Cl(1)#1	93.54(16)
C(1)-Rh(1)-Cl(1)	90.44(16)
C(15)-Rh(1)-Cl(1)	101.65(17)
C(18)-Rh(1)-Cl(1)	155.42(16)
C(14)-Rh(1)-Cl(1)	138.86(18)
C(16)-Rh(1)-Cl(1)	92.82(16)
C(17)-Rh(1)-Cl(1)	117.62(15)
Cl(1)#1-Rh(1)-Cl(1)	79.98(5)
Rh(1)#1-Cl(1)-Rh(1)	100.02(5)
C(1)-N(1)-C(2)	111.0(5)
C(1)-N(1)-C(4)	128.5(4)

C(2)-N(1)-C(4)	119.7(5)
N(2)-C(1)-N(1)	103.9(4)
N(2)-C(1)-Rh(1)	127.4(4)
N(1)-C(1)-Rh(1)	128.4(4)
C(1)-N(2)-C(3)	111.2(5)
C(1)-N(2)-C(8)	127.1(5)
C(3)-N(2)-C(8)	121.1(5)
C(3)-C(2)-N(1)	107.1(5)
C(3)-C(2)-H(2)	126.4
N(1)-C(2)-H(2)	126.4
C(2)-C(3)-N(2)	106.8(5)
C(2)-C(3)-H(3)	126.6
N(2)-C(3)-H(3)	126.6
C(7A)-C(4)-N(1)	122.0(10)
C(7)-C(4)-N(1)	117.8(7)
C(7)-C(4)-S(1)	114.1(7)
N(1)-C(4)-S(1)	127.5(4)
C(7A)-C(4)-S(1A)	115.6(10)
N(1)-C(4)-S(1A)	121.7(5)
C(4)-S(1)-C(5)	92.4(4)
C(6)-C(5)-S(1)	110.7(8)
C(6)-C(5)-H(5)	124.7
S(1)-C(5)-H(5)	124.7
C(5)-C(6)-C(7)	113.7(10)
C(5)-C(6)-H(6)	123.2
C(7)-C(6)-H(6)	123.2
C(4)-C(7)-C(6)	109.2(11)
C(4)-C(7)-H(7)	125.4
C(6)-C(7)-H(7)	125.4
C(4)-S(1A)-C(5A)	93.0(9)
C(6A)-C(5A)-S(1A)	107.8(12)
C(6A)-C(5A)-H(5A)	126.1
S(1A)-C(5A)-H(5A)	126.1
C(5A)-C(6A)-C(7A)	116.8(15)
C(5A)-C(6A)-H(6A)	121.6
C(7A)-C(6A)-H(6A)	121.6
C(4)-C(7A)-C(6A)	106.9(13)
C(4)-C(7A)-H(7A)	126.5
C(6A)-C(7A)-H(7A)	126.5
N(2)-C(8)-C(9)	110.7(6)
N(2)-C(8)-H(8A)	109.5

C(9)-C(8)-H(8A)	109.5
N(2)-C(8)-H(8B)	109.5
C(9)-C(8)-H(8B)	109.5
H(8A)-C(8)-H(8B)	108.1
C(10)-C(9)-C(8)	112.2(7)
C(10)-C(9)-H(9A)	109.2
C(8)-C(9)-H(9A)	109.2
C(10)-C(9)-H(9B)	109.2
C(8)-C(9)-H(9B)	109.2
H(9A)-C(9)-H(9B)	107.9
C(9)-C(10)-Si(1A)	128.8(8)
C(9)-C(10)-Si(1)	108.2(6)
C(9)-C(10)-H(10A)	110.1
Si(1)-C(10)-H(10A)	110.1
C(9)-C(10)-H(10B)	110.1
Si(1)-C(10)-H(10B)	110.1
H(10A)-C(10)-H(10B)	108.4
O(2)-Si(1)-O(3)	113.8(6)
O(2)-Si(1)-O(1)	103.7(6)
O(3)-Si(1)-O(1)	112.8(6)
O(2)-Si(1)-C(10)	115.4(5)
O(3)-Si(1)-C(10)	101.8(5)
O(1)-Si(1)-C(10)	109.5(5)
C(11)-O(1)-Si(1)	124.1(9)
C(12)-O(2)-Si(1)	129.1(11)
C(13)-O(3)-Si(1)	127.3(10)
O(1)-C(11)-H(11A)	109.5
O(1)-C(11)-H(11B)	109.5
H(11A)-C(11)-H(11B)	109.5
O(1)-C(11)-H(11C)	109.5
H(11A)-C(11)-H(11C)	109.5
H(11B)-C(11)-H(11C)	109.5
O(2)-C(12)-H(12A)	109.5
O(2)-C(12)-H(12B)	109.5
H(12A)-C(12)-H(12B)	109.5
O(2)-C(12)-H(12C)	109.5
H(12A)-C(12)-H(12C)	109.5
H(12B)-C(12)-H(12C)	109.5
O(3)-C(13)-H(13A)	109.5
O(3)-C(13)-H(13B)	109.5
H(13A)-C(13)-H(13B)	109.5

O(3)-C(13)-H(13C)	109.5
H(13A)-C(13)-H(13C)	109.5
H(13B)-C(13)-H(13C)	109.5
O(2A)-Si(1A)-O(3A)	108.6(15)
O(2A)-Si(1A)-O(1A)	118.3(14)
O(3A)-Si(1A)-O(1A)	100.2(15)
O(2A)-Si(1A)-C(10)	102.7(12)
O(3A)-Si(1A)-C(10)	121.4(13)
O(1A)-Si(1A)-C(10)	106.7(11)
C(11A)-O(1A)-Si(1A)	137.3(18)
C(12A)-O(2A)-Si(1A)	143(2)
C(13A)-O(3A)-Si(1A)	133(3)
O(1A)-C(11A)-H(11D)	109.5
O(1A)-C(11A)-H(11E)	109.5
H(11D)-C(11A)-H(11E)	109.5
O(1A)-C(11A)-H(11F)	109.5
H(11D)-C(11A)-H(11F)	109.5
H(11E)-C(11A)-H(11F)	109.5
O(2A)-C(12A)-H(12D)	109.5
O(2A)-C(12A)-H(12E)	109.5
H(12D)-C(12A)-H(12E)	109.5
O(2A)-C(12A)-H(12F)	109.5
H(12D)-C(12A)-H(12F)	109.5
H(12E)-C(12A)-H(12F)	109.5
O(3A)-C(13A)-H(13D)	109.5
O(3A)-C(13A)-H(13E)	109.5
H(13D)-C(13A)-H(13E)	109.5
O(3A)-C(13A)-H(13F)	109.5
H(13D)-C(13A)-H(13F)	109.5
H(13E)-C(13A)-H(13F)	109.5
C(18)-C(14)-C(15)	108.0(5)
C(18)-C(14)-C(19)	125.6(6)
C(15)-C(14)-C(19)	126.4(6)
C(18)-C(14)-Rh(1)	70.2(3)
C(15)-C(14)-Rh(1)	69.8(3)
C(19)-C(14)-Rh(1)	126.8(4)
C(14)-C(15)-C(16)	107.1(6)
C(14)-C(15)-C(20)	127.1(6)
C(16)-C(15)-C(20)	124.9(6)
C(14)-C(15)-Rh(1)	71.2(3)
C(16)-C(15)-Rh(1)	73.7(3)

C(20)-C(15)-Rh(1)	128.9(4)
C(17)-C(16)-C(15)	108.4(5)
C(17)-C(16)-C(21)	127.0(6)
C(15)-C(16)-C(21)	124.6(6)
C(17)-C(16)-Rh(1)	72.0(3)
C(15)-C(16)-Rh(1)	67.5(3)
C(21)-C(16)-Rh(1)	127.3(4)
C(16)-C(17)-C(18)	107.9(5)
C(16)-C(17)-C(22)	126.4(5)
C(18)-C(17)-C(22)	125.7(6)
C(16)-C(17)-Rh(1)	71.3(3)
C(18)-C(17)-Rh(1)	67.4(3)
C(22)-C(17)-Rh(1)	127.3(4)
C(14)-C(18)-C(17)	108.4(5)
C(14)-C(18)-C(23)	126.7(6)
C(17)-C(18)-C(23)	124.2(6)
C(14)-C(18)-Rh(1)	71.5(3)
C(17)-C(18)-Rh(1)	74.1(3)
C(23)-C(18)-Rh(1)	127.7(4)
C(14)-C(19)-H(19A)	109.5
C(14)-C(19)-H(19B)	109.5
H(19A)-C(19)-H(19B)	109.5
C(14)-C(19)-H(19C)	109.5
H(19A)-C(19)-H(19C)	109.5
H(19B)-C(19)-H(19C)	109.5
C(15)-C(20)-H(20A)	109.5
C(15)-C(20)-H(20B)	109.5
H(20A)-C(20)-H(20B)	109.5
C(15)-C(20)-H(20C)	109.5
H(20A)-C(20)-H(20C)	109.5
H(20B)-C(20)-H(20C)	109.5
C(16)-C(21)-H(21A)	109.5
C(16)-C(21)-H(21B)	109.5
H(21A)-C(21)-H(21B)	109.5
C(16)-C(21)-H(21C)	109.5
H(21A)-C(21)-H(21C)	109.5
H(21B)-C(21)-H(21C)	109.5
C(17)-C(22)-H(22A)	109.5
C(17)-C(22)-H(22B)	109.5
H(22A)-C(22)-H(22B)	109.5
C(17)-C(22)-H(22C)	109.5

H(22A)-C(22)-H(22C)	109.5
H(22B)-C(22)-H(22C)	109.5
C(18)-C(23)-H(23A)	109.5
C(18)-C(23)-H(23B)	109.5
H(23A)-C(23)-H(23B)	109.5
C(18)-C(23)-H(23C)	109.5
H(23A)-C(23)-H(23C)	109.5
H(23B)-C(23)-H(23C)	109.5
F(4)-B(1)-F(3)	111.9(8)
F(4)-B(1)-F(1)	112.7(9)
F(3)-B(1)-F(1)	109.2(8)
F(4)-B(1)-F(2)	106.1(8)
F(3)-B(1)-F(2)	109.6(9)
F(1)-B(1)-F(2)	107.1(7)
C(1X)-O(1X)-C(4X)	105.9(12)
O(1X)-C(1X)-C(2X)	110.0(14)
O(1X)-C(1X)-H(1X1)	109.7
C(2X)-C(1X)-H(1X1)	109.7
O(1X)-C(1X)-H(1X2)	109.7
C(2X)-C(1X)-H(1X2)	109.7
H(1X1)-C(1X)-H(1X2)	108.2
C(3X)-C(2X)-C(1X)	105.8(13)
C(3X)-C(2X)-H(2X1)	110.6
C(1X)-C(2X)-H(2X1)	110.6
C(3X)-C(2X)-H(2X2)	110.6
C(1X)-C(2X)-H(2X2)	110.6
H(2X1)-C(2X)-H(2X2)	108.7
C(2X)-C(3X)-C(4X)	101.5(14)
C(2X)-C(3X)-H(3X1)	111.5
C(4X)-C(3X)-H(3X1)	111.5
C(2X)-C(3X)-H(3X2)	111.5
C(4X)-C(3X)-H(3X2)	111.5
H(3X1)-C(3X)-H(3X2)	109.3
O(1X)-C(4X)-C(3X)	105.8(12)
O(1X)-C(4X)-H(4X1)	110.6
C(3X)-C(4X)-H(4X1)	110.6
O(1X)-C(4X)-H(4X2)	110.6
C(3X)-C(4X)-H(4X2)	110.6
H(4X1)-C(4X)-H(4X2)	108.7
C(4Y)-O(1Y)-C(1Y)	110.1(18)
C(2Y)-C(1Y)-O(1Y)	106.6(19)

C(2Y)-C(1Y)-H(1Y1)	110.4
O(1Y)-C(1Y)-H(1Y1)	110.4
C(2Y)-C(1Y)-H(1Y2)	110.4
O(1Y)-C(1Y)-H(1Y2)	110.4
H(1Y1)-C(1Y)-H(1Y2)	108.6
C(1Y)-C(2Y)-C(3Y)	105(2)
C(1Y)-C(2Y)-H(2Y1)	110.7
C(3Y)-C(2Y)-H(2Y1)	110.7
C(1Y)-C(2Y)-H(2Y2)	110.7
C(3Y)-C(2Y)-H(2Y2)	110.7
H(2Y1)-C(2Y)-H(2Y2)	108.8
C(2Y)-C(3Y)-C(4Y)	104(2)
C(2Y)-C(3Y)-H(3Y1)	111.0
C(4Y)-C(3Y)-H(3Y1)	111.0
C(2Y)-C(3Y)-H(3Y2)	111.0
C(4Y)-C(3Y)-H(3Y2)	111.0
H(3Y1)-C(3Y)-H(3Y2)	109.0
O(1Y)-C(4Y)-C(3Y)	103.8(18)
O(1Y)-C(4Y)-H(4Y1)	111.0
C(3Y)-C(4Y)-H(4Y1)	111.0
O(1Y)-C(4Y)-H(4Y2)	111.0
C(3Y)-C(4Y)-H(4Y2)	111.0
H(4Y1)-C(4Y)-H(4Y2)	109.0

Table S24. Anisotropic displacement parameters (Å^2 x 10^3) for **Rh13**. The anisotropic displacement factor exponent takes the form: $-2 \Pi^2[h^2 a^{*2} U_{11} + ... + 2 h k a^* b^* U_{12}]$.

	U11	U22	U33	U23	U13	U12
Rh(1)	16(1)	21(1)	33(1)	-11(1)	1(1)	-4(1)
Cl(1)	23(1)	25(1)	35(1)	-8(1)	3(1)	-10(1)
N(1)	14(2)	22(2)	46(3)	-11(2)	1(2)	-4(2)
C(1)	23(3)	25(3)	25(3)	-11(2)	0(2)	-7(2)
N(2)	22(2)	27(3)	40(3)	-6(2)	-3(2)	-9(2)
C(2)	18(3)	28(3)	53(4)	-5(3)	1(2)	-5(2)
C(3)	23(3)	36(3)	39(4)	-7(3)	-4(2)	-2(3)
C(4)	19(3)	22(3)	57(4)	-13(3)	9(2)	-9(2)
S(1)	24(1)	25(2)	57(2)	3(1)	4(1)	-11(1)
C(5)	27(3)	20(3)	63(4)	-4(3)	11(3)	-15(3)
C(6)	31(3)	26(3)	63(4)	-14(3)	11(3)	-16(3)
C(7)	31(4)	22(4)	68(4)	-19(4)	12(3)	-22(3)
S(1A)	30(3)	35(3)	79(3)	-26(3)	8(2)	-22(2)

C(5A)	31(4)	27(4)	61(5)	-11(4)	11(4)	-17(4)
C(6A)	26(4)	27(4)	59(5)	-2(4)	11(4)	-16(4)
C(7A)	27(5)	26(5)	61(6)	4(5)	13(5)	-16(4)
C(8)	38(3)	30(3)	32(3)	0(2)	-3(3)	-8(3)
C(9)	46(4)	55(5)	39(4)	-13(3)	-7(3)	-8(4)
C(10)	59(5)	84(6)	29(4)	-3(4)	-6(3)	-19(5)
Si(1)	79(2)	80(2)	41(2)	-20(2)	1(1)	-32(2)
O(1)	90(3)	85(3)	60(3)	-26(3)	0(3)	-32(3)
O(2)	87(3)	89(3)	59(3)	-15(3)	-3(2)	-32(3)
O(3)	89(3)	86(3)	57(3)	-17(3)	4(2)	-32(3)
C(11)	103(7)	96(7)	81(7)	-28(6)	13(6)	-39(6)
C(12)	101(6)	114(6)	81(6)	-9(5)	-3(5)	-22(5)
C(13)	108(7)	108(7)	69(6)	-10(6)	17(6)	-34(6)
Si(1A)	87(3)	86(3)	56(2)	-18(3)	0(2)	-32(3)
O(1A)	87(3)	85(3)	57(3)	-19(3)	4(3)	-30(3)
O(2A)	87(3)	88(4)	56(3)	-17(3)	-1(3)	-30(3)
O(3A)	90(4)	88(4)	61(4)	-16(4)	4(4)	-31(4)
C(11A)	99(9)	99(9)	75(9)	-15(9)	6(9)	-29(8)
C(12A)	98(6)	101(6)	69(6)	-13(6)	-1(6)	-27(6)
C(13A)	99(8)	98(8)	69(7)	-11(7)	11(7)	-34(7)
C(14)	30(3)	29(3)	41(4)	-21(3)	3(2)	-8(2)
C(15)	26(3)	33(3)	38(3)	-19(3)	10(2)	-7(2)
C(16)	17(3)	34(3)	41(3)	-20(3)	10(2)	-6(2)
C(17)	16(2)	23(3)	44(3)	-19(2)	4(2)	0(2)
C(18)	22(3)	26(3)	53(4)	-21(3)	4(2)	-10(2)
C(19)	36(3)	48(4)	41(4)	-29(3)	-5(3)	-8(3)
C(20)	44(4)	47(4)	44(4)	-10(3)	11(3)	-12(3)
C(21)	28(3)	38(4)	64(5)	-25(3)	11(3)	-12(3)
C(22)	31(3)	30(3)	48(4)	-12(3)	2(3)	-4(3)
C(23)	36(3)	18(3)	65(5)	-8(3)	11(3)	-6(3)
B(1)	72(7)	37(5)	50(6)	-3(4)	12(5)	-1(4)
F(1)	73(3)	37(2)	58(3)	-9(2)	12(2)	-12(2)
F(2)	87(4)	77(4)	90(4)	-5(3)	17(3)	-34(3)
F(3)	116(5)	44(3)	69(4)	-13(2)	27(3)	6(3)
F(4)	106(5)	79(4)	49(3)	-5(3)	4(3)	-13(3)
O(1X)	137(7)	155(7)	130(7)	-36(6)	0(6)	-61(6)
C(1X)	117(8)	134(9)	108(8)	-25(6)	-25(6)	-55(7)
C(2X)	134(9)	154(9)	134(9)	-57(7)	6(7)	-53(7)
C(3X)	144(9)	141(9)	134(9)	-51(7)	-4(7)	-59(7)
C(4X)	110(8)	113(8)	121(8)	-33(6)	-3(6)	-44(6)
O(1Y)	195(9)	221(10)	205(10)	-58(8)	2(7)	-115(8)

C(1Y)	160(10)	173(10)	153(10)	-45(8)	-3(8)	-81(8)
C(2Y)	203(13)	223(13)	204(13)	-37(9)	14(9)	-110(9)
C(3Y)	198(12)	213(12)	188(12)	-47(9)	5(9)	-101(9)
C(4Y)	138(9)	138(9)	148(10)	-34(7)	20(7)	-70(8)

Table S25. Hydrogen coordinates (x 10^4) and isotropic displacement parameters (Å^2 x 10^3) for **Rh13**.

	x	y	z	U(eq)
H(2)	4992	10908	4260	43
H(3)	5316	12597	3322	44
H(5)	7311	6147	6190	43
H(6)	6357	6455	4833	46
H(7)	6296	8370	3820	42
H(5A)	6615	6070	5360	46
H(6A)	7589	6592	6406	45
H(7A)	7956	8538	5904	47
H(8A)	8826	11588	2960	45
H(8B)	7534	12875	2954	45
H(9A)	7667	11189	1970	62
H(9B)	6426	12518	1941	62
H(10A)	8935	12386	1517	77
H(10B)	7530	13611	1301	77
H(11A)	8714	9243	129	142
H(11B)	7265	10379	-133	142
H(11C)	8488	10418	-614	142
H(12A)	4623	13846	-658	167
H(12B)	5393	14598	-473	167
H(12C)	5904	13743	-1123	167
H(13A)	9248	13348	-1294	154
H(13B)	9278	11992	-1103	154
H(13C)	7914	13233	-1405	154
H(11D)	10674	10442	-269	146
H(11E)	10413	11870	-613	146
H(11F)	10878	11196	337	146
H(12D)	4225	13893	-74	146
H(12E)	4595	14802	304	146
H(12F)	4799	14757	-638	146
H(13D)	8157	15202	-1357	141
H(13E)	8656	13775	-1409	141
H(13F)	7109	14690	-1465	141

H(19A)	9645	7338	2522	64
H(19B)	8941	8839	2350	64
H(19C)	8539	8027	3093	64
H(20A)	11767	9110	2080	73
H(20B)	11055	10280	2490	73
H(20C)	10190	9811	2052	73
H(21A)	13368	8539	4442	63
H(21B)	12785	9502	3566	63
H(21C)	13887	8094	3619	63
H(22A)	13458	5830	5132	60
H(22B)	12158	6299	5617	60
H(22C)	12980	7094	5444	60
H(23A)	9372	6539	4377	64
H(23B)	10372	6163	5160	64
H(23C)	10846	5442	4437	64
H(1X1)	6501	6957	2914	145
H(1X2)	6483	5623	3212	145
H(2X1)	6854	5274	1988	169
H(2X2)	6705	6677	1669	169
H(3X1)	8884	6015	1747	165
H(3X2)	8927	4706	1667	165
H(4X1)	9279	3891	3052	140
H(4X2)	10047	4741	2967	140
H(1Y1)	4571	8345	1908	190
H(1Y2)	3457	9784	1697	190
H(2Y1)	4373	10222	661	249
H(2Y2)	5390	8752	818	249
H(3Y1)	6149	10423	932	236
H(3Y2)	6934	8978	1438	236
H(4Y1)	6413	9911	2491	166
H(4Y2)	5055	11034	2009	166

Table S26. Torsion angles [°] for **Rh13**.

C(2)-N(1)-C(1)-N(2)	-1.8(6)
C(4)-N(1)-C(1)-N(2)	167.7(5)
C(2)-N(1)-C(1)-Rh(1)	171.5(4)
C(4)-N(1)-C(1)-Rh(1)	-19.0(8)
N(1)-C(1)-N(2)-C(3)	2.1(6)
Rh(1)-C(1)-N(2)-C(3)	-171.3(4)
N(1)-C(1)-N(2)-C(8)	-168.5(5)

Rh(1)-C(1)-N(2)-C(8)	18.1(8)
C(1)-N(1)-C(2)-C(3)	0.9(7)
C(4)-N(1)-C(2)-C(3)	-169.7(5)
N(1)-C(2)-C(3)-N(2)	0.5(7)
C(1)-N(2)-C(3)-C(2)	-1.7(7)
C(8)-N(2)-C(3)-C(2)	169.6(6)
C(1)-N(1)-C(4)-C(7A)	82.9(19)
C(2)-N(1)-C(4)-C(7A)	-108.4(18)
C(1)-N(1)-C(4)-C(7)	-106.7(12)
C(2)-N(1)-C(4)-C(7)	62.0(13)
C(1)-N(1)-C(4)-S(1)	82.0(7)
C(2)-N(1)-C(4)-S(1)	-109.2(6)
C(1)-N(1)-C(4)-S(1A)	-107.4(8)
C(2)-N(1)-C(4)-S(1A)	61.3(8)
C(7)-C(4)-S(1)-C(5)	-0.7(12)
N(1)-C(4)-S(1)-C(5)	170.8(6)
C(4)-S(1)-C(5)-C(6)	0.5(6)
S(1)-C(5)-C(6)-C(7)	-0.3(11)
N(1)-C(4)-C(7)-C(6)	-171.8(9)
S(1)-C(4)-C(7)-C(6)	0.6(17)
C(5)-C(6)-C(7)-C(4)	-0.2(17)
C(7A)-C(4)-S(1A)-C(5A)	-0.7(19)
N(1)-C(4)-S(1A)-C(5A)	-171.0(8)
C(4)-S(1A)-C(5A)-C(6A)	0.6(10)
S(1A)-C(5A)-C(6A)-C(7A)	-0.5(15)
N(1)-C(4)-C(7A)-C(6A)	170.8(11)
S(1A)-C(4)-C(7A)-C(6A)	0(3)
C(5A)-C(6A)-C(7A)-C(4)	0(2)
C(1)-N(2)-C(8)-C(9)	104.8(7)
C(3)-N(2)-C(8)-C(9)	-65.1(7)
N(2)-C(8)-C(9)-C(10)	-176.6(6)
C(8)-C(9)-C(10)-Si(1A)	179.9(8)
C(8)-C(9)-C(10)-Si(1)	164.3(5)
C(9)-C(10)-Si(1)-O(2)	60.5(8)
C(9)-C(10)-Si(1)-O(3)	-175.7(6)
C(9)-C(10)-Si(1)-O(1)	-56.1(7)
O(2)-Si(1)-O(1)-C(11)	42.0(12)
O(3)-Si(1)-O(1)-C(11)	-81.6(12)
C(10)-Si(1)-O(1)-C(11)	165.8(11)
O(3)-Si(1)-O(2)-C(12)	-14.7(15)
O(1)-Si(1)-O(2)-C(12)	-137.6(13)

C(10)-Si(1)-O(2)-C(12)	102.5(14)
O(2)-Si(1)-O(3)-C(13)	-59.3(13)
O(1)-Si(1)-O(3)-C(13)	58.6(12)
C(10)-Si(1)-O(3)-C(13)	175.8(11)
C(9)-C(10)-Si(1A)-O(2A)	35.1(15)
C(9)-C(10)-Si(1A)-O(3A)	156.5(14)
C(9)-C(10)-Si(1A)-O(1A)	-90.0(14)
O(2A)-Si(1A)-O(1A)-C(11A)	160(3)
O(3A)-Si(1A)-O(1A)-C(11A)	42(4)
C(10)-Si(1A)-O(1A)-C(11A)	-85(4)
O(3A)-Si(1A)-O(2A)-C(12A)	-31(5)
O(1A)-Si(1A)-O(2A)-C(12A)	-144(4)
C(10)-Si(1A)-O(2A)-C(12A)	99(4)
O(2A)-Si(1A)-O(3A)-C(13A)	-67(4)
O(1A)-Si(1A)-O(3A)-C(13A)	58(4)
C(10)-Si(1A)-O(3A)-C(13A)	175(3)
C(18)-C(14)-C(15)-C(16)	5.4(6)
C(19)-C(14)-C(15)-C(16)	-173.1(5)
Rh(1)-C(14)-C(15)-C(16)	65.5(4)
C(18)-C(14)-C(15)-C(20)	174.9(6)
C(19)-C(14)-C(15)-C(20)	-3.6(10)
Rh(1)-C(14)-C(15)-C(20)	-125.0(6)
C(18)-C(14)-C(15)-Rh(1)	-60.1(4)
C(19)-C(14)-C(15)-Rh(1)	121.4(6)
C(14)-C(15)-C(16)-C(17)	-3.1(6)
C(20)-C(15)-C(16)-C(17)	-172.9(6)
Rh(1)-C(15)-C(16)-C(17)	60.7(4)
C(14)-C(15)-C(16)-C(21)	175.4(5)
C(20)-C(15)-C(16)-C(21)	5.6(9)
Rh(1)-C(15)-C(16)-C(21)	-120.8(5)
C(14)-C(15)-C(16)-Rh(1)	-63.8(4)
C(20)-C(15)-C(16)-Rh(1)	126.4(6)
C(15)-C(16)-C(17)-C(18)	-0.3(6)
C(21)-C(16)-C(17)-C(18)	-178.8(5)
Rh(1)-C(16)-C(17)-C(18)	57.6(4)
C(15)-C(16)-C(17)-C(22)	179.1(5)
C(21)-C(16)-C(17)-C(22)	0.6(9)
Rh(1)-C(16)-C(17)-C(22)	-123.0(5)
C(15)-C(16)-C(17)-Rh(1)	-57.9(4)
C(21)-C(16)-C(17)-Rh(1)	123.6(6)
C(15)-C(14)-C(18)-C(17)	-5.6(6)

C(19)-C(14)-C(18)-C(17)	172.9(5)
Rh(1)-C(14)-C(18)-C(17)	-65.4(4)
C(15)-C(14)-C(18)-C(23)	-176.5(5)
C(19)-C(14)-C(18)-C(23)	2.0(9)
Rh(1)-C(14)-C(18)-C(23)	123.7(6)
C(15)-C(14)-C(18)-Rh(1)	59.8(4)
C(19)-C(14)-C(18)-Rh(1)	-121.7(6)
C(16)-C(17)-C(18)-C(14)	3.7(6)
C(22)-C(17)-C(18)-C(14)	-175.7(5)
Rh(1)-C(17)-C(18)-C(14)	63.7(4)
C(16)-C(17)-C(18)-C(23)	174.8(5)
C(22)-C(17)-C(18)-C(23)	-4.6(9)
Rh(1)-C(17)-C(18)-C(23)	-125.2(6)
C(16)-C(17)-C(18)-Rh(1)	-60.0(4)
C(22)-C(17)-C(18)-Rh(1)	120.6(5)
C(4X)-O(1X)-C(1X)-C(2X)	-15.0(17)
O(1X)-C(1X)-C(2X)-C(3X)	-6(2)
C(1X)-C(2X)-C(3X)-C(4X)	23.5(18)
C(1X)-O(1X)-C(4X)-C(3X)	30.4(16)
C(2X)-C(3X)-C(4X)-O(1X)	-32.9(16)
C(4Y)-O(1Y)-C(1Y)-C(2Y)	16(3)
O(1Y)-C(1Y)-C(2Y)-C(3Y)	6(3)
C(1Y)-C(2Y)-C(3Y)-C(4Y)	-23(3)
C(1Y)-O(1Y)-C(4Y)-C(3Y)	-30(2)
C(2Y)-C(3Y)-C(4Y)-O(1Y)	32(2)
